# Annual economic impacts of seasonal influenza on US counties: Spatial heterogeneity and patterns

**DOI:** 10.1186/1476-072X-11-16

**Published:** 2012-05-17

**Authors:** Liang Mao, Yang Yang, Youliang Qiu, Yan Yang

**Affiliations:** 1 Department of Geography, University of Florida, Gainesville, FL, 32611, USA; 2 Department of Geography, University at Buffalo, State University of New York, Amherst, NY, 14261, USA

**Keywords:** Influenza, Economic costs, US Counties, Vaccination, Spatial heterogeneity

## Abstract

Economic impacts of seasonal influenza vary across US counties, but little estimation has been conducted at the county level. This research computed annual economic costs of seasonal influenza for 3143 US counties based on Census 2010, identified inherent spatial patterns, and investigated cost-benefits of vaccination strategies. The computing model modified existing methods for national level estimation, and further emphasized spatial variations between counties, in terms of population size, age structure, influenza activity, and income level. Upon such a model, four vaccination strategies that prioritize different types of counties were simulated and their net returns were examined. The results indicate that the annual economic costs of influenza varied from $13.9 thousand to $957.5 million across US counties, with a median of $2.47 million. Prioritizing vaccines to counties with high influenza attack rates produces the lowest influenza cases and highest net returns. This research fills the current knowledge gap by downscaling the estimation to a county level, and adds spatial variability into studies of influenza economics and interventions. Compared to the national estimates, the presented statistics and maps will offer detailed guidance for local health agencies to fight against influenza.

## Introduction

Every year in the US, influenza viruses pose remarkable impacts on socio-economy, such as costs of medical care, loss of productivity, and deaths [1]. Since economic considerations are essential for influenza control, decision makers often need to examine following questions for health interventions. How much will an influenza season cost the US? Which states or counties bear high costs? Where to distribute vaccines to achieve the maximum returns? To date, only a small number of studies have estimated the economic impacts of influenza in the US. The Office of Technology Assessment reported that the influenza accounts for $1 ~ 3 billion per year in medical costs [2]. Meltzer, et al. argued that the annual economic burden of pandemic influenza could range from $71.3 ~ 166.5 billion [3]. The latest estimation by Molinari et al. indicated that the short-term costs and long-term burden of seasonal influenza can be amounted to $26.8 ~ $87.1 billion a year [4]. These studies have established systematic methods to analyze influenza economics and offered valuable guidance for interventions.

Previous studies, however, have focused on the national-level estimation, while few have drilled down to a county level and taken into account spatial heterogeneity between counties. Many factors were assumed to be homogenous across counties but in fact vary remarkably, such as the influenza activity, population size, age structure, income level, and so on. Current estimates for the entire US fail to differentiate influenza impacts between counties, and thus offer little information for state/county-level health planning. For instance, the national estimates cannot inform the design of county-based vaccination strategies, i.e., where (or which counties) should receive vaccines first for a best cost-effectiveness. In addition, the lack of county-level knowledge may cloud the identification of contributing factors to influenza costs, due to the modifiable areal unit problem (MAUP). That is, different levels of aggregation, such as the county-, state-, and national levels, may produce variation in statistical associations [5]. Although the Centers for Disease Control and Prevention (CDC) have offered a FluAid software to help estimate local economic impacts [6], this tool still employs nationwide homogeneous parameters, and cannot characterize any inter-county variations except for demographics.

This article aims to estimate annual economic impact of seasonal influenza for 3143 US counties based on US Census 2010, characterize the inter-county variation of such impact, and investigate cost-effectiveness of vaccination strategies. The estimation modified existing methods in the literature, and further emphasized spatial variations among counties, in terms of population size, age structure, influenza activity, and income level. Spatial and statistical analyses were conducted to identify spatial patterns of influenza impacts across counties. Futhermore, four county-based vaccination strategies were simulated and their cost-effectiveness was compared to identify the optimal.

## Materials and methods

For a specific county, the economic impacts of influenza are the sum of monetary costs to each influenza case in this county (Equation 1).

(1) $$\begin{array}{l} Totaleconomic\;impacts_{i}=\sum_{j}^{n}Montery\;costs_{i,j}=\sum_{j}^{n}\left(DirectCosts_{i,j}+IndirectCosts_{i,j}\right)\\ =\sum_{j}^{n}\left(MedicalCosts_{i,j}+LossofProductivity_{i,j}\right) \end{array}$$

(where *i* represents a county, *n* denotes all influenza cases in county *i*, and *j* indicates an influenza case in county *i)*.

The monetary costs of an influenza case can be further divided into direct and indirect costs. The direct costs result from expenses of healthcare resources, e.g., hospitalization and antiviral treatment, while the indirect costs come from the loss of productivity from school/work absenteeism and death [3,7]. According to Equation 1, following three sub-sections describe the datasets and methods to estimate the county-level influenza cases, direct and indirect costs, respectively.

### County-level influenza cases and health outcomes

#### *County population by age group*

The population of a county is a basis for calculating the number of influenza cases. Since influenza infections vary by age, the county population was divided into five age groups: under 5, 5–17, 18–49, 50–64, and ≥65 years. The county population by age group was extracted from the US Census 2010 Summary File 1 [8], the most recent demographic data available. For mapping purposes, the county population was further geo-referenced to its administrative boundary from the US Census Topologically Integrated Geographic Encoding and Referencing system (TIGER) Products [9].

### County influenza attack rate by age group

For each age group in a county, the number of influenza cases is a product of the age-group population and the age-specific attack rate. Since no data has been published on the influenza attack rates by county, three pieces of information were used for estimation: the national attack rates by age group, the national Influenza Like Illness (ILI) rates (ILI visits per 100,000), and the ILI rates of major US cities. As shown in Equation 2, the influenza attack rate in county *i* at age group *g*$\left(\hat{Attack\;rate_{i,g}}\right)$ is equal to the national attack rate at the age group $\left(Nation\;attack\;rate_{g}\right)$*g* adjusted by a ratio between the county ILI rate $\left(\hat{County}ILI_{i}\right)$ and the national ILI rate (*National ILI)*. Simiarly, the standard devation of $\hat{Attack\;rate_{i,g}}$ is the adjusted standard devation of $Nation\;attack\;rate_{g}$.

(2) $$\left\{ \begin{array}{l} \;\hat{Attack\;rate_{i,g}}=\frac{\hat{CountyILI_{i}}}{NationILI}\times Nation\;attack\;rate_{g}\\ Std\;dev\left(\hat{Attack\;rate_{i,g}}\right)=\frac{\hat{CountyILI_{i}}}{NationILI}\times Std\;dev(Nation\;attack\;rate_{g}) \end{array}\right.$$

The national attack rates by age group $\left(Nation\;attack\;rate_{g}\right)$ and the associated standard deviations were adopted from the surveillance data and established literature [4], with details listed in Supplementary file 1: Table S1. The national ILI rate $\left(NationILI\right)$ was the average of weekly naitonal ILI rates from 2003 to 2010, published by the Google Flu Trends [10]. To gain a representative ILI rate for seasonal influenza, the data of Season 2009–10 was eliminated before averaging, due to the H1N1 flu pandemic. The Flu Trends data could be a reasonable proxy to influenza activities because recent literature has reported its capability of predicting influenza activity [11,12]. The correlation between the data from the Google Flu Trends and CDC Virus Surveillance can achieve up to 82% [13].

**Figure 1:**
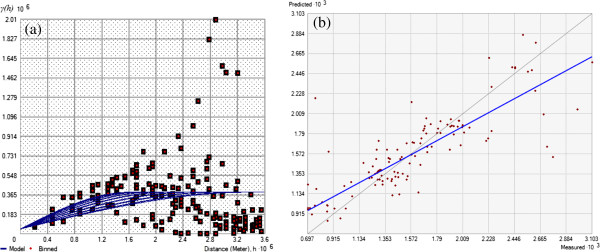
The kriging model to estimate county ILI rates: (a) Sample semivariogram of city ILI rates (red squares) and the fitted spherical model (blue curves): 46692*Nugget+342120*Spherical(3405500, 1599800, 328.8°); (b) Scatterplot of reported ILI rates vs. kriging predicted ILI rates.The blue straightline summarizes the trend as: *y=0.698x+465.73* (Figures were created with ESRI ArcGIS 10.0).

The county specific ILI rate $\hat{County}ILI_{i}$ (in Equation 2) was interpolated using the ILI rates of major cities near to the county centroid. The ILI rates of 117 major US cities were also averaged from the weekly ILI rates from Google Flu Trends. Similar to the national ILI rate, the data of Season 2009–10 was removed before averaging. The ordinary kriging, a sophisticated geostatistical method, was employed for interpolation, because it has been commonly used for predicting ILI rates [14,15]. In this research, a major advantage of kriging lies in its ability to minimize the standard error of an estimate and quantify this standard error [16,17]. The kriging interploation was accomplished by four steps. First, the 117 major cities were georeferenced to their geographic locations. Their ILI rates were used to compute semivariances between cities at different separations, and consitute a sample semivariogram (squares in Figure 1a). Second, a spherical model was fitted to the sample semivariogram (curves in Figure 1a), using a least square method. The sill, nugget, and major range of the spherical model were estimated to be 34.21 km, 46,692, and 3405.5 km respectively. Third, the searching neighbor was set to the nearest 12 to 20 cities around the centriod of each county. The fitted spehrical model was used to assign weights to neigbhor cities, and the kriged county ILI was the weighted sum of ILI rates at neighbor cities. Lastly, a cross-validation was conducted and gave a mean error of −0.05 (close to zero) and an average standard error of 310.1 (Figure 1b). 

**Figure 2:**
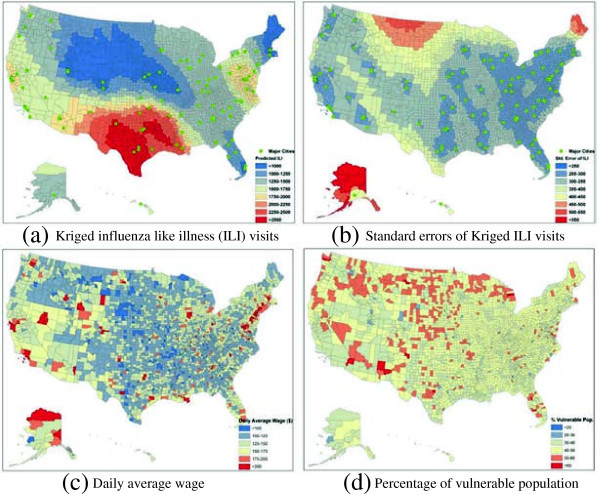
Spatial heterogeneity between US counties, in terms of (a) Kriging estimated influenza like illness (ILI) visits (per 100,000 persons), (b) Standard errors of ILI estimation, (c)Daily average wage as an indicator of income level, (d)Percentage of vulnerable population (age <5 and >50 years).

XThe kriged county ILI rates (Figure 2a) and associated standard errors (Figure 2b) were used together to simulate the $\hat{County}ILI_{i}$. Based upon the estimates of $\left(Nation\;attack\;rate_{g}\right)$, $\left(NationILI\right)$, and $\hat{County}ILI_{i}$, the county attack rates $\hat{Attack\;rate_{i,g}}$ were cacluated for each age group, and then multiplied by the age-group populations to obtain the number of influenza cases by age group.

### Risks and health outcomes by age group

Influenza cases may progress to different outcomes, which costs distinctly. To refine the cost estimation, this research classified influenza cases of each age group into two types of risks, and then four health outcomes. The two types of risks refer to non-high and high risks of developing serious complications. An influenza case was defined to be high risk if one or more medical conditions were consistent with high-risk conditions identified by the Advisory Committee on Immunization Practices (ACIP), thus more likely to develop severe outcomes [18]. For each age-risk group, influenza cases were further separated into four health outcomes, including: self-care (not medically attended), outpatient visit, hospitalization, and death. The likelihoods of becoming a high-risk case and the probabilities of developing each health outcome were estimated by Molinari et al. [4] based on the literature, medical records, and reports. Mean values and statistical distributions of these parameters are shown in Additional file 1: Table S1 by age and by risk group. Finally, each influenza case in a county was assigned one of 40 categories (5 age groups × 2 types of risks × 4 health outcomes). Each category was associated with a direct cost and an indirect cost discussed below.

### Direct costs by county

The direct costs come from the medical expenditure in response to influenza (e.g., hospitalizations, outpatient visits, and drug purchases), and vary over the 40 age-risk-outcome categories. Molinari et al. [4] had estimated the national average medical cost (and distribution) for each of the 40 categories according to a proprietary database that contains health insurance claims data from 4 million insured persons [19]. Details about the costs per category are given in Additional file 1: Table S2, and these estimates were used to parameterize the county model after an inflation adjustment. Since the work of Molinari et al. was done in 2003, this research inflated the medical costs from 2003 to 2010 (the year of census data) based on the consumer price index (CPI) of these two years. The CPI ratio between year 2010 and 2003 was set to 1.185 as reported by the Bureau of Labor Statistics [20]. Therefore, this research estimated the economic impacts if a typical seasonal influenza hit US in 2010 or years later on. By simulating the inflated direct costs for each case, the direct costs of influenza to a county were the sum of costs from all cases.

### Indirect costs by county

The indirect costs include the loss of productivity due to work/school absenteeism and death. The loss of productivity due to work/school absenteeism was calculated by multiplying the length of days absent from work by the monetary value lost per day [21]. The national average length of work/school absenteeism, along with its distribution, had been previously estimated by Molinari et al. [4] for the 40 age-risk-outcome categories (Additional file 1: Table S2). This research assumed that the length of absenteeism in a county follows the nationwide distribution, while the monetary value lost per day varies between counties. For each county, the monetary value lost per day (Figure 2c) was equal to the average wage per job [22] divided by the total working days per year (260 days).

For influenza cases ending with deaths, the productivity loss was estimated as the present value of lost earnings (PVLE), the projected earnings until retirement based on a person’s current salary [23]. The national average of PVLE had been previously extrapolated by age group in the work of Molinari et al. [4], and were inflated to 2010 in this research (Additional file 1: Table S2). In such a way, the indirect cost of each influenza case was evaluated and the sum of all cases gave the indirect costs of influenza in a county.

### Total economic costs by county

**Figure 3:**
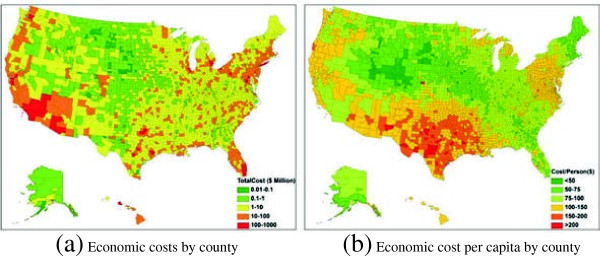
**Spatial heterogeneity between US counties, in terms of (a) Total economic costs of influenza, and (b) Economic cost per capita.** The detailed county estimates and associated 95% confidence intervals were available at http://heep.geog.ufl.edu/flucost/ and Additional file 2.

Upon the evaluation of influenza case number, associated direct and indirect costs, the total economic costs for each county were valued using Equation 1. To quantify the uncertainties in estimation, this research employed an individual-based stochastic approach. Each individual in a county is a discrete modeling unit with properties, such as the age group, attack rate, health outcome, direct and indirect costs. Using Monte-Carlo simulation, these properties were assigned random values from their adjusted probability distributions according to (Additional file 1: Table S1 and S2) and kriging estimates. The simulation was run by 1,000 realizations to establish 95% confidence intervals for estimates of interest. Averaged from 1,000 realizations, the total economic costs for each county were mapped in Figure 3a, and the economic costs per capita were also computed by prorating the total costs to the county population (Figure 3b). Due to the word limits, the county estimates and associated 95% confidence intervals were presented in Additional file 2 and published on an interactive online mapping system ( http://heep.geog.ufl.edu/flucost/). To capture the spatial clusters of economic impacts, a Moran’s *I* statistic was applied the county cost map.

### Net returns of vaccination against influenza

Vaccination is widely suggested to be a major strategy for reducing the impacts of influenza. The use of vaccine was assumed to avert outcomes of influenza cases, and thus led to savings of costs that need to treat these outcomes. The net return from vaccination is an important economic measure in cost-benefit analysis of such intervention. Following the method of Meltzer, et al. [3], the total net return was calculated by Equation 3:

(3) $$\begin{array}{l} Total\;Net\;Return=\sum^{\;}Net\;Return_{age,risk\;group}\\ \;=\sum^{\;}(Savings\;from\;outcomes\;averted\;in\;population_{age.risk\;group}\\ \;-Vaccinated\;popoulation_{age,risk\;group}\times Cost\;of\;vaccine\;per\;person) \end{array}$$

The savings from averted outcomes in a population was determined by the effectiveness of vaccines (reported in Additional file 1: Table S3). To consider the match between vaccine and influenza virus, a probability of good match 80% [24] was incorporated into the simulation model. The vaccine effectiveness of a good match was assumed to be twice as high as that of a poor match. The vaccinated population was a function of vaccination coverage, i.e., the proportion of people being inoculated. The cost of vaccine per person was estimated to be $21 by Meltzer et al. in 1999 [3] and inflated to $27.50 in 2010. This cost includes the vaccine price, its distribution and administration fees (health-care worker time, supplies), patient travel, time lost from work and other activities; and cost of side effects.

**Figure 4:**
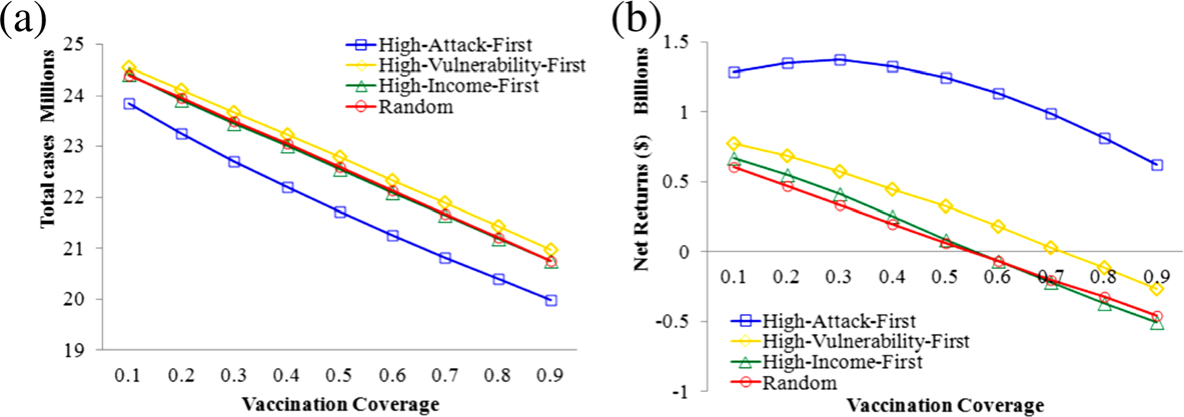
Comparison of cost-benefits between four county-based strategies: (a) Total influenza cases and (b) Net returns ($) as a function of vaccination coverage from 10% to 90% of the population.

By explicitly considering the county differences, this research was capable of investigating four county-based vaccination strategies. The first strategy, referred to as the ‘random strategy’, randomly vaccinated US population to a predefined coverage (in percentage), regardless of the age group and the county they live in. The second strategy vaccinated the same amount of people, but prioritized those who live in the counties of high influenza attack rates, thus called ‘High-Attack-First’ strategy. The third strategy is similar to the second, but prioritizing those who live in the counties with high proportion of vulnerable population, named as a ‘High-Vulnerability-First’ strategy. According to the recommendation by CDC [25], the population under 5 years and over 50 years was defined as the most vulnerable population (Figure 2d). The last strategy aims to vaccinate people who live in the counties with high income level, and thus called ‘High-Income-First’ strategy. Of the four strategies, each was simulated based on Equation 3 at a range of vaccination coverage from 10% to 90% with a 10% increment. The average influenza case number and the net return ($) were estimated for each strategy-coverage combination after 1,000 simulation runs (Figure 4). The four strategies were compared to identify the optimal one that produces the fewest influenza cases and highest net return. 

## Results and discussion

At the national level, the seasonal influenza resulted in 25.34 million cases a year (95%CI: 24.83 – 25.86 million), 8.1% of the total population in 2010 (Table 1). The annual economic costs were estimated to be $29.12 billion (95% CI: $28.44 – $29.87 billion), approximately 0.2% of the gross domestic product of US in 2010. About 65% of the economic costs comes from the indirect cost, i.e., loss of productivity due to work absenteeism and death, while the rest of 35% is from the direct medical cost.

**Table 1:** Estimated annual influenza impacts on the entire US using population and costs of 2010

	**Total**	**Lower 95%CI**	**Upper 95%CI**
**Number of cases (Million)**	25.34	24.83	25.86
**Direct costs (Millions $)**	10,262.98	10,046.60	10,482.80
**Indirect costs (Millions $)**	18,853.66	18,321.81	19,439.80
**Total economic costs (Millions $)**	29,116.65	28,441.24	29,870.84

The economic impacts of influenza varied dramatically across US counties (Table 2). Kalawao County, Hawaii, had the smallest number of influenza cases (7 cases, 95% CI: 2–13) and the lowest economic costs ($13,883, 95% CI: $281– $97,037), because its population was only 90 by 2010. On the other hand, Los Angeles County, California, is the most populous county in US (9,818,605 persons in 2010). Not surprisingly, this county possessed the largest number of cases (868,587, 95% CI: 610,091 – 1,118,763) and the highest economic costs ($957.5 million, 95%CI: $664.7–1248.2 million). Due to the wide variability among counties, the median value would be a more reasonable statistic to indicate the centrality than the mean value. On average, a US county would have 2009 influenza cases per year based on US census 2010. The economic costs for a county averaged $2.47 million, and a quarter of counties (above the 75% percentile) may experience an economic loss greater than $6.42 million.

**Table 2:** Annual influenza impacts on US counties using population and costs of 2010

	**Minimum**	**Maximum**	**Mean**	**Median**	**Standard deviation**	**25% percentile**	**75% percentile**
**Number of cases**	7	868,588	8,061	2009	27,950	829	5530
**Direct costs (Millions $)**	0.004	327.11	3.26	0.89	10.56	0.38	2.35
**Indirect costs (Millions $)**	0.008	630.38	6.00	1.57	20.17	0.65	4.10
**Total costs (Millions $)**	0.014	957.49	9.26	2.47	30.72	1.02	6.42
**Costs/Capita ($)**	32.51	272.36	97.50	89.27	32.77	75.84	113.16

*Statistics and 95% confidence intervals by county are available at: http://heep.geog.ufl.edu/flucost/.

The geographic distribution of economic costs is of interest (Figure 3a). Note that each color shade represents differences orders of magnitude. The distribution of economic costs strongly coincides with the population distribution, in that a large population often has more influenza cases and requires more money to alleviate the disease. In general, counties with high costs were concentrated in the coastal areas, such as the Pacific region (Washington, Oregon, California) and the Middle Atlantic region (New York, Pennsylvania, Connecticut, New Jersey). The inland areas in the Mid-West and Mountain regions, such as Montana, Idaho, Minnesota, and Oklahoma, had relatively lower costs. The Moran’s *I*, indicated that the county economic costs were significantly clustered over space (Moran’s *I* = 0.28, *Z* score = 27.95, *p*-value < 0.05). Two spatial clusters of high costs can be easily identified: one was centered at Los Angeles County, California along the West Coast, while the other encompassed the Boston-New York City metropolitan areas at the East Coast. Other scattered metropolitan areas also showed high levels of costs, such as Miami (Florida), Seattle (Washington), and Houston (Texas), etc.

It is also interesting to examine what if the economic costs were prorated to every person in a county (Figure 3b). The economic cost per capita by county exhibited a distinct spatial pattern. The annual cost per capita ranged from $32.5 (Ziebach County, South Dakota) to $272.4 (Llano County, Texas). One spatial cluster of high cost per capita was the South Central region (Texas, Oklahoma, Arkansas, and Louisiana), where every resident needed to spend more than $150 to combat influenza. The other cluster can be found in the mid-Atlantic and south Atlantic regions, with a personal cost between $100 ~ 150 per capita. Residents in the Mountain region bore the lightest burden (under $50 per capita) except for southern counties in Arizona and New Mexico. This spatial pattern can be explained by the fact that the economic cost per capita is independent of county population size. Counties with high cost per capita were associated with high levels of influenza attack rates. A person in a high attack-rate county is more likely to be infected and develop high-risk complications, and thus costs more than a person in a low attack rate county.

With regarding to the vaccination strategies, the ‘High-Attack-First’ strategy significantly outperforms any other strategy due to the lowest influenza cases and highest net return (Figure 4). In other words, it would be an optimal strategy to first vaccinating people living in the West-South-Central region, including Texas, Oklahoma, Arkansas, and New Mexico (dark red regions in Figure 2a). A possible reason is that this strategy directly prohibited the transmission of influenza, while other strategies address influenza indirectly through population vulnerability and income level. The effect of ‘High-Attack-First’ vaccination is, thus, more straightforward than other strategies. The random, ‘High-Vulnerability-First’ and ‘High-Income-First’ strategies reduce the influenza cases to a similar extent, but the ‘High-Vulnerability-First’ strategy returns more monetary benefits, and is therefore the second cost-effective strategy. This is because the ‘High-Vulnerability-First’ strategy greatly decreases the people who may otherwise develop severe outcomes, such as hospitalizations and death, and thus curtails the major source of influenza costs. In this sense, it is also a wise strategy to prioritize vaccines to the North West region and North Midwest region (including Idaho, Wyoming, North Dakota, South Dakota, Minnesota and Wisconsin), where the proportion of vulnerable population is pretty high (dark red regions in Figure 2c).

There are several issues that require special attention as a result of this study. First, although many parameters were estimated for individual counties, a few parameters were still assumed to be nationwide homogeneous, such as the length of absenteeism and the present value of lost earnings. The major reason is the lack of relevant data for each of US counties. These homogenous parameters may reduce the variability of costs between counties and lead to a smoother map than the reality. This may also mislead our understandings on counties with extremely low and high costs. Second, the use of Google Flu Trends data would introduce biases into the estimation, even though its correlation to the viral surveillance can reach 82%. A finer scale influenza ILI data at the county level would be helpful to improve estimation. The authors had tried to directly obtain county flu infection data, instead of using Google flu data. Unfortunately, among the 51 US states, only 6 have explicitly published their county-level flu attack rates. The US CDC only releases regional flu data, with each region covering several states. Therefore, the Google flu data could be the best data that is publicly available and contains sufficient details. Third, the influenza attack rates, direct and indirect costs were assumed to be homogeneous within a county, regardless of urban and rural areas. But in reality, these parameters may vary between urban and rural areas because of different population density, land use patterns, accessibility to healthcare, etc. It remains unclear whether or not the modeling of urban–rural difference would significantly change the current estimation, but this question warrants a future study.

## Conclusions

The contributions of this research have two folds. First, the current estimation of influenza impacts uses nationwide homogenous parameters, which flatten the spatial variations among US counties. This research is the first attempt to calculate and map the county-level economic impacts of seasonal influenza in the US, thereby filling the current gap that only national estimates are available. The economic costs of influenza range from $13.9 thousand to $957.5 million among US counties, varying over demographics, economy, and epidemics. There are two spatial clusters of high costs: one centered at Los Angeles County, California along the West Coast, while the other embracing the Boston-New York City metropolitan areas on the East Coast. Secondly, before this research, most studies investigate vaccination strategies only at a national level, but few have considered the county differences, i.e., distributing vaccine resources based on county characteristics. This research has explored four county-based strategies and suggested that vaccination prioritizing counties with high attack rates would produce the greatest cost-benefits. This research adds a county/spatial perspective into the design of health interventions, and sheds insight on new cost-effective health policies.

It is argued that any estimation model can only produce crude approximations to reality. The key of this research is not to look for the absolute numeric predictions, but for differences in outcomes between different counties or between different scenarios. In this sense, this research lays a foundation for county-level study of influenza economics and interventions, and can be easily expanded to other infectious diseases. The numerical estimates and maps presented here not only inform general health planning for the entire US, but also offer detailed guidance for state or county level interventions to fight future influenza outbreaks.

## Competing interests

The authors declare that they have no competing interests.

## Authors’ contributions

LM designed the work, performed all coding and simulation, and drafted the manuscript. YY carried out part of data collection and analyses. YLQ and YY established the GIS website. All authors read and approved the final manuscript.

## Authors’ information

LM and YLQ are Assistant professors of Geography in the University of Florida. YY is a PhD candiate of Geography in the University of Florida. The other YY is a PhD candiate of Geography in the University at Buffalo, State University of New York.

## Supplementary Material

Additional file 1 **Supplementary file 1.** Annual Economic Impacts of Seasonal Influenza and Vaccination on US Counties: Spatial Heterogeneity and Patterns [3,4]. Click here for file

Additional file 2 **Supplementary file 2.** Annual Economic Impacts of Seasonal Influenza and Vaccination on US Counties: Spatial Heterogeneity and Patterns. Click here for file
