# Supplementary material for: Annual economic impacts of seasonal influenza on US counties: Spatial heterogeneity and patterns
Source: Int J Health Geogr. 2012 May 17;11:16. doi: 10.1186/1476-072X-11-16 (PMC3479051; doi:10.1186/1476-072X-11-16)
Supplement: Additional file 1 — Supplementary file 1. Annual Economic Impacts of Seasonal Influenza and Vaccination on US Counties: Spatial Heterogeneity and Patterns [3,4]. [file 1476-072X-11-16-S1.doc]

**Supporting Information-1**

**Annual Economic Impacts of Seasonal Influenza and Vaccination on US Counties: Spatial Heterogeneity and Patterns**

Liang Mao^a^, Yang Yang^a^, Youliang Qiu^a^, Yan Yang^b^

^a^Department of Geography, University of Florida, Gainesville, FL, 32611, USA

^b^Department of Geography, University at Buffalo, State University of New York at Buffalo, Amherst, NY, 14261, USA

**Table 1** Nationwide influenza parameters and distributions by age group, by risk and by health outcomes, all adopted from Molinari et al.

| **Influenza Parameters** | **Age Group** | **Mean** | **Standard Deviation** |
| --- | --- | --- | --- |
| **Attack rates** | | | |
|  | 0-4 | 0.203 | 0.062 |
|  | 5-17 | 0.102 | 0.032 |
|  | 18-49 | 0.066 | 0.017 |
|  | 50-64 | 0.066 | 0.017 |
|  | 65+ | 0.090 | 0.024 |
| **Likelihoods of high-risk influenza cases** | | | |
|  | 0-4 | 0.052 | 0.890 |
|  | 5-17 | 0.106 | 0.360 |
|  | 18-49 | 0.149 | 0.340 |
|  | 50-64 | 0.330 | 0.700 |
|  | 65+ | 0.512 | 0.730 |
| **Pr (Outpatient visit| flu infection)** | | | |
| Non-High risk cases | 0-4 | 0.455 | 0.098 |
|  | 5-17 | 0.318 | 0.061 |
|  | 18-49 | 0.313 | 0.014 |
|  | 50-64 | 0.313 | 0.014 |
|  | 65+ | 0.620 | 0.027 |
| High risk cases | 0-4 | 0.910 | 0.250 |
|  | 5-17 | 0.635 | 0.167 |
|  | 18-49 | 0.625 | 0.118 |
|  | 50-64 | 0.625 | 0.118 |
|  | 65+ | 0.820 | 0.093 |
| **Pr (Hospitalization| flu infection)** | | | |
| All risks | 0-4 | 0.0141 | 0.0047 |
|  | 5-17 | 0.0006 | 0.0002 |
|  | 18-49 | 0.0042 | 0.0014 |
|  | 50-64 | 0.0193 | 0.0064 |
|  | 65+ | 0.0421 | 0.0140 |
| **Pr (Death| flu infection)** | | | |
| All risks | 0-4 | 0.00004 | 0.00001 |
|  | 5-17 | 0.00001 | 0.00000 |
|  | 18-49 | 0.00009 | 0.00003 |
|  | 50-64 | 0.00134 | 0.00045 |
|  | 65+ | 0.01170 | 0.00390 |
| **Pr (Self-care| flu infection)** | | | |
| Non-High risk | 0-4 | Pr (Self-care| flu infection)  =1- Pr (Outpatient visit| flu infection)- Pr (Hospitalization| flu infection)- Pr (Death| flu infection) | |
|  | 5-17 |  |  |
|  | 18-49 |  |  |
|  | 50-64 |  |  |
|  | 65+ |  |  |
| High risk | 0-4 | Pr (Self-care| flu infection)  =1- Pr (Outpatient visit| flu infection)- Pr (Hospitalization| flu infection)- Pr (Death| flu infection) | |
|  | 5-17 |  |  |
|  | 18-49 |  |  |
|  | 50-64 |  |  |
|  | 65+ |  |  |

**Table 2** Nationwide influenza-related costs and distributions by age group, by risk and by health outcome, all adopted from Molinari et al.

| **Cost per health outcome by age and risk group** | **Medical cost** | | | |  | **Lost productivity (days)*** | | | | |  |
| --- | --- | --- | --- | --- | --- | --- | --- | --- | --- | --- | --- |
|  | Mean | S.D. | Distribution | |  | Mean | | Distribution | | |  |
| **Self-care** |  |  |  | |  |  | |  | | |  |
| All risks |  |  |  | |  |  | |  | | |  |
| 0-4 | 3 | 2 | Log normal | |  | 1.0 | | Poisson | | |  |
| 5-17 | 3 | 2 | Log normal | |  | 0.5 | | Poisson | | |  |
| 18-49 | 3 | 2 | Log normal | |  | 0.5 | | Poisson | | |  |
| 50-64 | 3 | 2 | Log normal | |  | 0.5 | | Poisson | | |  |
| 65+ | 2 | 2 | Log normal | |  | 1.0 | | Poisson | | |  |
| **Outpatient visit** |  |  |  | |  |  | |  | | |  |
| Non-High risk |  |  |  | |  |  | |  | | |  |
| 0-4 | 167 | 307 | Log normal | |  | 1 | | Poisson | | |  |
| 5-17 | 95 | 258 | Log normal | |  | 1 | | Poisson | | |  |
| 18-49 | 125 | 438 | Log normal | |  | 1 | | Poisson | | |  |
| 50-64 | 150 | 766 | Log normal | |  | 2 | | Poisson | | |  |
| 65+ | 242 | 1,544 | Log normal | |  | 3 | | Poisson | | |  |
| High risk |  |  |  | |  |  | |  | | |  |
| 0-4 | 574 | 1,266 | Log normal | |  | 6 | | Poisson | | |  |
| 5-17 | 649 | 1,492 | Log normal | |  | 4 | | Poisson | | |  |
| 18-49 | 725 | 1,717 | Log normal | |  | 2 | | Poisson | | |  |
| 50-64 | 733 | 1,307 | Log normal | |  | 4 | | Poisson | | |  |
| 65+ | 476 | 1,131 | Log normal | |  | 7 | | Poisson | | |  |
| **Hospitalization** |  |  |  | |  |  | |  | | |  |
| Non-High risk |  |  |  | |  |  | |  | | |  |
| 0-4 | 10,880 | 36,189 | Log normal | |  | 8 | | Poisson | | |  |
| 5-17 | 15,014 | 86,804 | Log normal | |  | 9 | | Poisson | | |  |
| 18-49 | 19,012 | 44,636 | Log normal | |  | 12 | | Poisson | | |  |
| 50-64 | 22,304 | 95,727 | Log normal | |  | 13 | | Poisson | | |  |
| 65+ | 11,451 | 23,128 | Log normal | |  | 13 | | Poisson | | |  |
| High risk |  |  |  | |  |  | |  | | |  |
| 0-4 | 81,596 | 123,626 | Log normal | |  | 31 | | Poisson | | |  |
| 5-17 | 41,918 | 50,393 | Log normal | |  | 23 | | Poisson | | |  |
| 18-49 | 47,722 | 85,644 | Log normal | |  | 21 | | Poisson | | |  |
| 50-64 | 41,309 | 74,798 | Log normal | |  | 24 | | Poisson | | |  |
| 65+ | 16,750 | 32,091 | Log normal | |  | 18 | | Poisson | | |  |
| **Death** |  |  |  | | **Present value of lost earnings ($)** | | | | | |  |
| Non-High risk | Mean | S.D. | Distribution | Mean | | | S.D. | | | Distribution |  |
| 0-4 | 28,818 | 24,483 | Log normal | 1,074,866 | | | 222,803 | | Log normal | | |
| 5-17 | 28,818 | 24,483 | Log normal | 1,276,012 | | | 900,934 | | Log normal | | |
| 18-49 | 76,336 | 91,654 | Log normal | 1,374,115 | | | 2,754,332 | | Log normal | | |
| 50-64 | 118,575 | 333,879 | Log normal | 521,083 | | | 1,588,835 | | Log normal | | |
| 65+ | 41,948 | 96,467 | Log normal | 185,846 | | | 597,639 | | Log normal | | |
| High risk |  | | | | | | | | | | |
| 0-4 | 267,954 | 221,130 | Log normal | 1,074,866 | | | 222,803 | | Log normal | | |
| 5-17 | 267,954 | 221,130 | Log normal | 1,276,012 | | | 900,934 | | Log normal | | |
| 18-49 | 75,890 | 65,267 | Log normal | 1,374,115 | | | 2,754,332 | | Log normal | | |
| 50-64 | 118,842 | 345,973 | Log normal | 521,083 | | | 1,588,835 | | Log normal | | |
| 65+ | 33,011 | 61,904 | Log normal | 185,846 | | | 597,639 | | Log normal | | |

*Value of a lost productivity day for all risk and age groups has a log normal distribution with a S.D. of $13.

**Table 3 Vaccine effectiveness by health outcome and age group, adopted from Meltzer et al.**

| **Health Outcome**  **Age group** | **Vaccine effectiveness*** | | | |
| --- | --- | --- | --- | --- |
|  | Self-care | Outpatient visits | Hospitalization | Death |
| All risk |  |  |  |  |
| 0-4 | 0.4 | 0.4 | 0.5 | 0.7 |
| 5-17 | 0.4 | 0.4 | 0.5 | 0.7 |
| 18-49 | 0.4 | 0.4 | 0.5 | 0.7 |
| 50-64 | 0.4 | 0.4 | 0.5 | 0.7 |
| 65+ | 0.4 | 0.4 | 0.55 | 0.6 |

* Vaccine effectiveness is defined as the reduction in the number of cases in each of the age and disease categories

References

Meltzer, M.I., Cox, N.J., Fukuda, K., 1999. The economic impact of pandemic influenza in the United States: priorities for intervention. Emerging Infectious Diseases 5, 659-671.

Molinari, N.A.M., Ortega-Sanchez, I.R., Messonnier, M.L., Thompson, W.W., Wortley, P.M., Weintraub, E., Bridges, C.B., 2007. The annual impact of seasonal influenza in the US: measuring disease burden and costs. Vaccine 25, 5086-5096.
