# Supplementary material for: Annual economic impacts of seasonal influenza on US counties: Spatial heterogeneity and patterns
Source: Int J Health Geogr. 2012 May 17;11:16. doi: 10.1186/1476-072X-11-16 (PMC3479051; doi:10.1186/1476-072X-11-16)
Supplement: Additional file 2 — Supplementary file 2. Annual Economic Impacts of Seasonal Influenza and Vaccination on US Counties: Spatial Heterogeneity and Patterns. [file 1476-072X-11-16-S2.pdf]

FIPS	County NAME	State Name	CountyPop	TotalCases	Lower 95%CI	Upper 95% CI	Direct Cost $	Lower 95%CI	Upper 95% CI	Indirect Cost $	Lower 95%CI	Upper 95% CI	Total Cost $	Lower 95%CI	Upper 95% CI
01001	Autauga	Alabama	54,571	4,304	2,465.50	6,134.00	1,645,530.79	754,209.78	2,917,398.28	2,867,580.06	665,581.02	10,013,347.54	4,513,110.85	1,687,184.27	12,277,175.60
01003	Baldwin	Alabama	182,265	16,139	9,311.50	22,444.50	7,326,525.99	3,902,010.27	11,131,566.10	12,609,015.80	4,931,587.45	25,913,934.24	19,935,541.78	9,218,021.84	34,955,449.30
01005	Barbour	Alabama	27,457	2,007	1,154.50	2,833.50	865,875.76	297,161.94	1,905,065.37	1,428,569.09	268,625.69	4,720,351.98	2,294,444.85	673,620.38	5,992,273.71
01007	Bibb	Alabama	22,915	1,756	988.50	2,488.50	716,883.21	273,974.05	1,557,750.00	1,195,330.50	207,219.13	5,012,401.33	1,912,213.71	525,537.63	5,959,085.16
01009	Blount	Alabama	57,322	4,238	2,564.00	5,864.00	1,803,457.50	802,799.59	3,346,440.37	2,960,084.52	685,150.94	9,395,366.17	4,763,542.02	1,695,127.58	12,110,700.45
01011	Bullock	Alabama	10,914	812	465.00	1,172.50	345,733.11	101,413.14	927,794.31	502,001.77	78,522.50	2,155,784.17	847,734.88	195,052.68	2,747,611.35
01013	Butler	Alabama	20,947	1,692	918.50	2,395.00	755,546.97	268,106.00	1,681,036.25	1,269,794.06	212,664.21	5,687,821.02	2,025,341.04	577,562.69	6,447,765.63
01015	Calhoun	Alabama	118,572	8,515	4,874.00	12,099.00	3,617,956.94	1,847,167.44	6,207,392.11	6,417,210.78	1,957,801.58	17,392,854.37	10,035,167.72	4,202,063.87	21,564,915.76
01017	Chambers	Alabama	34,215	2,455	1,334.50	3,484.00	1,118,915.72	492,655.22	2,194,509.69	2,050,389.60	349,698.43	6,984,632.66	3,169,305.31	972,967.05	8,378,214.86
01019	Cherokee	Alabama	25,989	1,819	1,036.50	2,612.50	855,580.31	342,356.73	1,817,435.18	1,637,453.08	254,501.41	5,971,780.62	2,493,033.38	694,266.91	7,285,828.07
01021	Chilton	Alabama	43,643	3,439	2,113.00	4,883.00	1,427,647.12	638,902.28	2,721,414.83	2,228,693.54	501,960.77	8,459,623.13	3,656,340.66	1,292,958.15	10,318,581.28
01023	Choctaw	Alabama	13,859	1,188	730.50	1,634.50	574,830.16	224,046.19	1,286,443.36	1,135,511.33	234,315.10	4,768,070.01	1,710,341.49	502,767.56	5,440,392.09
01025	Clarke	Alabama	25,833	2,203	1,251.50	3,109.00	967,888.97	343,015.20	2,008,665.69	1,555,881.26	333,178.27	5,478,330.28	2,523,770.24	807,159.70	6,769,743.03
01027	Clay	Alabama	13,932	1,033	605.50	1,449.00	473,824.14	160,070.88	1,130,263.75	842,862.31	117,195.94	3,961,595.59	1,316,686.45	312,854.41	4,613,429.34
01029	Cleburne	Alabama	14,972	1,081	630.50	1,523.00	488,312.35	151,963.05	1,163,148.78	887,602.56	129,047.03	3,856,695.69	1,375,914.91	324,417.30	4,730,951.99
01031	Coffee	Alabama	49,948	3,884	2,231.00	5,615.50	1,641,721.94	729,980.49	3,189,544.81	2,812,851.81	620,300.16	9,880,303.57	4,454,573.76	1,558,400.57	11,880,728.31
01033	Colbert	Alabama	54,428	3,970	2,215.50	5,623.50	1,846,824.23	844,911.50	3,282,895.05	3,449,980.78	806,036.57	11,023,924.78	5,296,805.01	1,783,377.98	14,014,847.49
01035	Conecuh	Alabama	13,228	1,091	600.00	1,563.00	510,165.43	160,012.35	1,232,456.85	894,867.27	132,921.37	4,480,936.61	1,405,032.70	309,178.28	5,054,268.97
01037	Coosa	Alabama	11,539	867	531.00	1,192.50	420,850.25	145,785.06	1,074,279.81	685,605.92	115,972.48	3,349,102.41	1,106,456.17	279,731.73	3,988,433.29
01039	Covington	Alabama	37,765	2,989	1,583.50	4,339.00	1,386,154.92	541,129.02	2,521,256.56	2,635,330.27	485,805.94	9,573,781.70	4,021,485.19	1,185,270.56	11,158,876.01
01041	Crenshaw	Alabama	13,906	1,065	585.50	1,525.50	469,607.58	152,768.90	1,168,916.65	863,195.69	121,258.50	3,975,995.84	1,332,803.27	303,583.55	4,528,691.91
01043	Cullman	Alabama	80,406	5,999	3,643.00	8,503.50	2,650,140.12	1,281,015.08	4,466,429.68	4,792,106.21	1,280,652.84	14,466,476.46	7,442,246.33	2,812,369.73	17,388,107.25
01045	Dale	Alabama	50,251	3,840	2,265.00	5,406.00	1,562,098.66	711,877.38	2,993,121.59	3,174,410.94	873,325.10	12,964,432.90	4,736,509.60	1,703,722.05	14,815,438.85
01047	Dallas	Alabama	43,820	3,558	2,034.50	5,102.50	1,476,057.89	677,999.03	2,778,127.39	2,491,596.08	520,943.92	7,938,685.69	3,967,653.97	1,310,090.62	9,651,102.36
01049	De Kalb	Alabama	71,109	5,126	2,925.00	7,294.50	2,112,943.80	1,030,633.83	3,876,246.71	3,567,485.40	878,280.55	10,546,049.87	5,680,429.20	2,043,333.89	13,454,993.18
01051	Elmore	Alabama	79,303	5,965	3,456.50	8,466.00	2,385,834.64	1,151,089.00	4,364,360.23	3,690,199.56	995,881.66	10,452,177.07	6,076,034.19	2,365,203.55	13,219,669.30
01053	Escambia	Alabama	38,319	3,156	1,919.50	4,467.00	1,380,254.36	628,151.62	2,825,482.14	2,465,201.52	532,095.61	8,527,081.67	3,845,455.88	1,265,829.03	10,400,454.43
01055	Etowah	Alabama	104,430	7,512	4,339.50	10,868.50	3,367,249.30	1,668,759.07	5,590,121.86	5,760,994.20	1,660,463.44	16,478,085.21	9,128,243.50	3,677,994.23	20,612,302.31
01057	Fayette	Alabama	17,241	1,320	766.00	1,862.50	619,413.41	213,341.27	1,406,975.73	1,105,596.93	155,696.75	5,647,841.83	1,725,010.34	401,779.86	6,504,626.05
01059	Franklin	Alabama	31,704	2,371	1,318.50	3,436.00	992,281.22	402,999.22	2,077,385.24	1,719,184.93	319,382.23	7,305,001.51	2,711,466.15	822,212.25	9,098,356.39
01061	Geneva	Alabama	26,790	2,064	1,199.50	2,930.00	970,188.99	378,766.09	2,036,814.29	1,630,094.89	298,493.91	7,084,238.79	2,600,283.87	750,540.22	8,409,126.66
01063	Greene	Alabama	9,045	725	415.50	1,051.00	337,629.82	96,234.42	980,078.31	557,312.68	89,727.49	3,023,915.47	894,942.50	219,360.19	3,552,610.51
01065	Hale	Alabama	15,760	1,250	728.00	1,780.50	535,623.70	187,912.63	1,219,132.46	898,159.94	144,181.38	3,897,100.59	1,433,783.64	347,924.63	4,601,244.12
01067	Henry	Alabama	17,302	1,248	724.00	1,822.00	604,211.30	201,259.12	1,506,275.62	1,158,913.55	173,573.06	5,231,440.88	1,763,124.84	415,833.15	5,917,227.94
01069	Houston	Alabama	101,547	7,660	4,616.50	10,806.00	3,212,559.33	1,632,417.83	5,489,491.48	5,876,596.93	1,792,069.62	15,562,049.02	9,089,156.27	3,753,124.34	19,689,151.74
01071	Jackson	Alabama	53,227	3,759	2,265.00	5,371.00	1,739,806.68	766,220.52	3,446,824.04	2,987,940.04	712,519.42	9,712,814.15	4,727,746.73	1,648,454.12	11,895,344.45
01073	Jefferson	Alabama	658,466	50,614	31,275.00	69,868.50	20,931,493.49	12,978,603.16	30,428,104.11	39,609,547.24	20,302,812.78	70,204,618.65	60,541,040.73	33,509,745.12	97,163,417.80
01075	Lamar	Alabama	14,564	1,116	648.50	1,559.00	536,193.79	176,851.19	1,194,361.82	933,643.37	143,964.15	4,239,074.94	1,469,837.16	366,239.56	4,964,400.59
01077	Lauderdale	Alabama	92,709	6,526	3,606.00	9,390.50	2,980,713.62	1,359,074.90	5,431,793.21	5,248,187.43	1,390,970.02	14,253,488.29	8,228,901.05	3,173,240.20	18,496,314.84
01079	Lawrence	Alabama	34,339	2,562	1,571.00	3,594.50	1,119,020.87	466,198.62	2,195,767.17	1,999,143.75	449,663.32	7,198,639.89	3,118,164.62	1,055,051.40	8,389,464.16
01081	Lee	Alabama	140,247	10,024	5,764.00	14,195.00	3,578,364.34	1,655,402.44	5,950,320.29	5,715,316.57	1,618,020.36	15,457,031.16	9,293,680.91	3,652,171.98	20,027,428.15
01083	Limestone	Alabama	82,782	5,907	3,365.50	8,420.50	2,389,732.94	1,112,786.85	4,144,340.86	4,032,783.36	1,122,442.48	11,386,434.90	6,422,516.31	2,481,977.75	14,895,127.55
01085	Lowndes	Alabama	11,299	890	502.50	1,305.00	393,738.42	120,151.36	1,091,746.28	661,409.44	110,199.85	2,724,366.21	1,055,147.87	249,994.17	3,575,358.54
01087	Macon	Alabama	21,452	1,572	902.00	2,240.50	683,457.04	240,464.17	1,599,322.80	1,104,562.22	212,897.67	4,052,134.34	1,788,019.27	503,530.63	4,947,540.45
01089	Madison	Alabama	334,811	23,945	13,885.00	33,449.50	9,614,559.02	5,200,862.90	14,448,562.36	18,326,750.42	7,852,151.79	35,510,963.79	27,941,309.44	13,902,372.88	47,352,113.52
01091	Marengo	Alabama	21,027	1,729	1,027.00	2,465.00	768,290.79	282,790.87	1,744,876.62	1,357,859.28	233,230.84	5,278,432.45	2,126,150.07	587,122.29	6,307,185.42
01093	Marion	Alabama	30,776	2,287	1,237.50	3,319.50	1,058,306.51	384,936.37	2,134,952.40	1,922,207.17	310,536.06	7,942,091.33	2,980,513.68	758,054.90	9,272,447.00
01095	Marshall	Alabama	93,019	6,860	3,849.00	9,931.00	2,877,487.59	1,363,894.78	4,905,687.08	4,858,792.42	1,275,148.08	14,422,522.47	7,736,280.01	2,936,843.00	18,083,125.92
01097	Mobile	Alabama	412,992	38,462	22,830.00	53,846.50	15,705,189.93	8,960,182.30	23,208,896.46	27,822,196.39	13,260,873.85	51,170,536.09	43,527,386.32	23,357,153.94	68,899,792.77
01099	Monroe	Alabama	23,068	1,946	1,122.00	2,727.50	887,396.66	341,877.23	1,779,021.22	1,563,800.15	315,462.78	6,803,979.74	2,451,196.81	751,683.39	8,158,887.03
01101	Montgomery	Alabama	229,363	17,645	10,103.00	25,092.00	6,901,181.17	3,494,538.12	10,737,067.83	12,367,785.61	4,672,890.60	26,432,089.50	19,268,966.78	8,785,218.96	35,384,921.02
01103	Morgan	Alabama	119,490	8,804	5,108.50	12,533.50	3,725,444.01	1,784,406.43	6,189,870.31	6,814,166.25	2,288,498.55	19,034,897.41	10,539,610.26	4,436,721.62	23,320,832.85
01105	Perry	Alabama	10,591	848	489.50	1,211.00	378,168.05	109,807.21	905,893.04	624,925.99	84,479.92	3,337,182.36	1,003,094.04	213,120.50	3,761,716.33
01107	Pickens	Alabama	19,746	1,553	851.00	2,198.50	701,460.32	252,952.88	1,494,264.47	1,230,377.50	190,477.24	5,224,306.70	1,931,837.82	504,246.15	5,968,131.51
01109	Pike	Alabama	32,899	2,412	1,415.00	3,410.50	962,001.34	387,743.47	1,937,842.77	1,683,975.84	329,876.04	6,186,159.06	2,645,977.18	796,178.27	7,502,614.50
01111	Randolph	Alabama	22,913	1,676	1,004.00	2,395.50	776,859.59	294,565.47	1,688,447.82	1,325,688.97	211,753.46	5,558,878.88	2,102,548.57	570,690.86	6,792,704.54
01113	Russell	Alabama	52,947	3,957	2,362.00	5,590.50	1,596,957.38	663,178.97	2,927,902.20	2,627,679.11	601,418.31	8,203,731.64	4,224,636.49	1,476,262.88	10,602,833.06
01115	St. Clair	Alabama	83,593	6,231	3,588.50	8,569.00	2,585,186.52	1,240,214.35	4,495,815.46	4,335,267.60	1,096,361.60	12,133,618.05	6,920,454.12	2,615,998.26	14,794,231.19
01117	Shelby	Alabama	195,085	15,081	9,124.50	21,028.00	5,801,348.68	3,069,508.14	9,363,687.41	10,479,265.80	3,940,354.56	26,646,398.27	16,280,614.48	7,595,463.40	32,659,428.77
01119	Sumter	Alabama	13,763	1,118	665.00	1,602.50	492,664.25	157,935.66	1,286,664.09	815,352.22	143,638.09	3,638,397.97	1,308,016.47	339,198.60	4,200,822.86
01121	Talladega	Alabama	82,291	6,163	3,710.50	8,707.50	2,615,869.69	1,281,105.78	4,716,214.57	4,520,231.72	1,466,044.77	12,357,530.86	7,136,101.40	2,975,434.52	15,832,069.91
01123	Tallapoosa	Alabama	41,616	3,085	1,832.50	4,496.00	1,418,030.59	572,247.59	2,727,581.30	2,500,284.80	509,910.75	7,765,559.12	3,918,315.39	1,306,326.74	9,359,205.94
01125	Tuscaloosa	Alabama	194,656	14,635	9,176.00	20,807.50	5,596,972.55	3,066,907.82	8,979,543.61	9,621,577.91	3,336,514.64	22,811,956.47	15,218,550.46	6,921,620.00	29,551,291.61
01127	Walker	Alabama	67,023	5,153	3,049.50	7,289.50	2,343,218.75	1,125,976.76	4,060,742.10	4,104,915.88	1,138,187.56	11,722,577.63	6,448,134.63	2,593,649.94	15,175,130.10
01129	Washington	Alabama	17,581	1,577	972.00	2,262.50	680,556.19	258,192.83	1,598,276.56	1,263,516.82	312,042.97	4,861,440.52	1,944,073.01	641,792.74	5,804,686.45

01131	Wilcox	Alabama	11,670	958	569.50	1,333.00	413,032.07	134,160.04	997,447.13	716,287.50	135,761.26	3,519,661.75	1,129,319.58	298,878.50	4,075,684.88
01133	Winston	Alabama	24,484	1,815	1,065.00	2,586.50	843,617.15	331,246.78	1,730,307.96	1,532,636.76	262,510.01	6,488,748.63	2,376,253.92	682,263.73	7,282,547.61
02013	Aleutians East	Alaska	3,141	221	42.50	415.00	84,855.38	4,385.06	412,111.57	110,496.52	6,061.20	849,310.77	195,351.91	12,794.76	1,090,811.34
02016	Aleutians West	Alaska	5,561	386	39.50	739.50	129,972.45	3,742.62	497,041.57	195,804.61	7,891.39	1,365,302.95	325,777.06	13,445.87	1,644,357.94
02020	Anchorage	Alaska	291,826	21,943	11,776.50	32,933.00	7,654,536.74	3,541,363.76	12,479,326.62	13,598,035.86	5,149,165.05	31,290,413.84	21,252,572.60	9,173,559.32	42,894,471.92
02050	Bethel	Alaska	17,013	1,392	353.50	2,343.50	435,576.68	61,224.87	1,079,868.62	611,381.23	60,126.10	3,082,667.29	1,046,957.91	132,515.17	3,745,830.75
02060	Bristol Bay	Alaska	997	73	16.50	128.00	31,388.90	1,047.63	145,193.90	54,606.67	2,518.77	381,558.80	85,995.57	3,803.97	557,323.93
02070	Dillingham	Alaska	4,847	389	91.50	664.00	118,082.07	16,290.88	416,836.88	179,321.27	16,892.27	1,184,619.74	297,403.34	37,415.56	1,365,738.47
02090	Fairbanks North Star	Alaska	97,581	7,243	1,412.00	13,028.50	2,506,866.36	365,512.03	5,252,849.83	4,115,242.34	452,987.15	13,068,160.07	6,622,108.70	815,409.18	16,580,811.10
02100	Haines	Alaska	2,508	188	52.00	329.50	89,500.15	6,196.51	392,181.35	184,260.39	9,993.80	1,141,778.18	273,760.54	19,180.35	1,370,752.12
02110	Juneau	Alaska	31,275	2,406	781.00	4,054.50	919,500.36	212,521.18	2,101,216.95	1,541,422.74	205,403.24	6,751,013.18	2,460,923.10	445,030.36	7,934,104.87
02122	Kenai Peninsula	Alaska	55,400	4,100	1,774.50	6,409.50	1,715,930.75	612,984.90	3,356,862.73	3,040,031.41	630,506.69	9,749,101.41	4,755,962.16	1,274,733.43	12,435,022.64
02130	Ketchikan Gateway	Alaska	13,477	1,015	253.50	1,731.50	403,276.26	49,294.63	1,154,702.97	626,298.97	65,590.92	2,537,932.58	1,029,575.23	114,351.39	3,120,419.98
02150	Kodiak Island	Alaska	13,592	1,076	284.00	1,878.50	363,516.26	46,719.50	970,912.12	570,092.04	66,911.36	3,024,465.63	933,608.30	126,383.02	3,706,731.48
02164	Lake and Peninsula	Alaska	1,631	129	26.00	231.50	43,961.72	1,814.97	186,649.43	53,091.32	3,829.51	369,796.97	97,053.04	5,980.77	542,778.39
02170	Matanuska-Susitna	Alaska	88,995	6,799	2,906.50	10,730.50	2,394,911.06	862,031.13	4,359,491.96	3,820,596.08	871,851.38	11,396,056.91	6,215,507.14	1,986,775.31	14,815,722.95
02180	Nome	Alaska	9,492	808	223.50	1,375.50	253,606.11	36,928.56	759,148.50	409,016.58	44,529.02	2,270,271.39	662,622.69	90,969.88	2,691,745.03
02185	North Slope	Alaska	9,430	748	99.00	1,423.00	258,565.35	14,742.74	807,324.18	526,133.18	40,374.66	2,274,502.47	784,698.53	60,472.62	2,740,136.36
02188	Northwest Arctic	Alaska	7,523	646	125.50	1,209.00	200,837.68	18,308.41	746,753.37	364,340.37	28,272.49	1,937,814.25	565,178.05	58,252.29	2,243,218.57
02198	e of Wales-Outer Ketch	Alaska	5,559	424	119.50	723.50	167,886.12	18,729.34	583,465.35	315,048.41	23,912.56	1,741,164.48	482,934.53	46,754.15	2,140,882.38
02220	Sitka	Alaska	8,881	672	194.50	1,148.00	290,896.05	43,104.16	903,229.63	525,279.09	43,859.57	2,875,686.23	816,175.14	96,793.39	3,538,238.32
02230	kagway-Yakutat-Angoo	Alaska	968	71	17.50	128.00	33,774.85	1,107.10	201,947.53	69,755.53	3,331.43	522,362.33	103,530.37	4,876.30	618,954.54
02240	Southeast Fairbanks	Alaska	7,029	522	101.00	925.50	203,635.84	15,993.36	644,797.10	368,037.07	31,947.34	1,734,892.43	571,672.91	55,177.31	2,054,524.71
02261	Valdez-Cordova	Alaska	9,636	716	228.50	1,136.50	274,470.67	41,539.56	810,880.57	438,673.10	58,517.17	2,176,874.03	713,143.77	110,375.42	2,881,736.10
02270	Wade Hampton	Alaska	7,459	636	174.00	1,105.50	171,491.67	26,630.16	545,393.82	226,700.91	20,410.10	1,657,404.95	398,192.58	49,288.31	1,957,119.31
02275	Wrangell-Petersburg	Alaska	2,369	178	46.00	300.50	80,973.20	7,318.62	311,447.56	136,084.37	9,402.76	921,613.33	217,057.57	18,770.91	1,097,362.39
02290	Yukon-Koyukuk	Alaska	5,588	446	99.00	782.50	176,146.06	14,343.92	574,512.75	290,575.08	18,253.10	1,854,761.29	466,721.14	33,844.53	2,082,740.84
04001	Apache	Arizona	71,518	7,232	4,407.00	10,001.50	2,658,322.14	1,358,655.50	4,594,135.76	4,709,376.76	1,391,184.29	13,279,488.40	7,367,698.90	3,061,010.08	16,060,705.38
04003	Cochise	Arizona	131,346	13,727	9,204.00	18,242.00	6,240,959.67	3,695,021.98	9,464,812.26	12,084,165.63	5,265,234.76	24,709,354.73	18,325,125.29	9,533,703.20	31,795,067.12
04005	Coconino	Arizona	134,421	11,691	6,661.00	16,655.00	4,277,509.52	2,191,251.73	6,923,668.20	7,013,302.17	2,315,179.73	16,947,526.70	11,290,811.70	4,791,913.63	22,371,189.31
04007	Gila	Arizona	53,597	5,268	3,468.00	6,915.50	2,830,516.48	1,524,256.67	4,785,534.28	5,446,903.55	1,748,174.24	13,522,925.27	8,277,420.03	3,643,550.53	17,001,685.56
04009	Graham	Arizona	37,220	3,994	2,642.00	5,329.00	1,510,764.09	737,705.42	2,903,791.43	2,613,037.56	642,635.15	8,218,921.44	4,123,801.65	1,546,513.53	10,358,698.00
04011	Greenlee	Arizona	8,437	912	582.50	1,259.00	358,602.35	110,420.89	903,183.28	748,386.26	196,007.85	3,293,188.46	1,106,988.61	330,105.26	3,822,803.85
04012	La Paz	Arizona	20,489	1,872	1,168.50	2,622.50	1,169,081.10	482,815.08	2,349,940.28	2,238,821.93	482,803.79	7,542,068.29	3,407,903.04	1,169,932.20	8,795,587.48
04013	Maricopa	Arizona	3,817,117	362,695	257,244.00	476,996.00	139,170,723.60	97,742,761.87	184,478,689.50	260,227,867.00	174,281,886.70	365,372,570.30	399,398,590.70	274,597,036.50	542,778,700.50
04015	Mohave	Arizona	200,186	17,331	10,863.00	23,845.00	9,208,565.32	5,219,614.05	14,350,759.76	17,435,690.13	7,835,727.97	34,306,458.38	26,644,255.45	13,649,418.09	45,510,323.35
04017	Navajo	Arizona	107,449	10,588	6,401.00	15,068.00	4,153,216.98	2,263,540.88	6,834,756.63	7,219,984.60	2,614,207.04	18,622,820.89	11,373,201.58	5,233,187.06	22,777,729.06
04019	Pima	Arizona	980,263	93,441	61,062.00	124,781.00	40,552,616.83	26,145,493.45	56,674,471.51	75,680,717.92	43,905,212.61	116,940,989.50	116,233,334.80	72,399,600.35	167,183,120.40
04021	Pinal	Arizona	375,770	37,968	26,683.50	49,152.50	15,237,116.00	10,328,933.75	21,072,541.51	27,628,040.88	14,611,026.79	51,157,651.85	42,865,156.89	25,965,734.17	69,613,207.67
04023	Santa Cruz	Arizona	47,420	5,018	3,340.50	6,636.00	1,974,104.60	1,062,967.79	3,479,501.72	3,397,492.04	1,063,956.53	9,442,821.13	5,371,596.63	2,364,290.71	12,040,056.78
04025	Yavapai	Arizona	211,033	19,334	12,707.50	25,193.50	10,759,952.00	6,534,495.38	15,782,123.47	19,967,311.17	9,723,476.58	36,519,196.02	30,727,263.17	17,333,109.13	49,002,736.22
04027	Yuma	Arizona	195,751	18,878	11,697.50	26,322.50	7,700,943.06	4,386,813.38	11,621,797.00	13,631,149.56	5,425,664.29	28,762,201.73	21,332,092.62	10,287,882.16	36,465,375.81
05001	Arkansas	Arkansas	19,019	2,046	1,509.50	2,605.00	930,152.76	436,479.67	1,772,795.42	1,653,689.85	359,482.74	6,340,478.14	2,583,842.62	897,174.72	7,289,181.96
05003	Ashley	Arkansas	21,853	2,633	1,983.50	3,343.00	1,159,249.80	574,719.75	2,170,335.67	1,941,296.60	545,063.59	6,365,065.46	3,100,546.40	1,284,534.01	7,783,262.04
05005	Baxter	Arkansas	41,513	3,347	2,076.50	4,635.00	1,924,012.41	972,919.34	3,311,237.02	3,926,565.29	1,046,224.82	11,510,084.71	5,850,577.70	2,338,149.60	13,763,062.55
05007	Benton	Arkansas	221,339	22,444	14,998.50	30,008.50	8,447,637.11	5,073,113.49	12,562,484.92	15,742,521.82	7,279,055.75	33,103,503.70	24,190,158.93	12,907,737.65	42,117,265.25
05009	Boone	Arkansas	36,903	3,248	2,105.50	4,374.50	1,507,012.26	739,055.81	2,914,385.93	2,561,624.75	677,571.09	8,261,868.83	4,068,637.01	1,633,376.63	10,315,309.49
05011	Bradley	Arkansas	11,508	1,420	1,029.50	1,788.00	662,633.87	284,801.83	1,423,576.20	1,054,672.69	209,862.50	4,310,933.53	1,717,306.57	561,724.45	5,451,756.26
05013	Calhoun	Arkansas	5,368	654	483.00	834.00	315,867.79	100,611.15	836,328.77	631,698.86	136,207.08	3,292,144.28	947,566.64	257,675.82	3,924,134.35
05015	Carroll	Arkansas	27,446	2,521	1,686.00	3,357.00	1,220,866.52	590,222.85	2,464,894.18	2,004,949.92	430,260.17	6,967,393.88	3,225,796.44	1,144,760.63	8,268,130.31
05017	Chicot	Arkansas	11,800	1,363	975.00	1,763.50	626,036.19	265,593.77	1,322,923.99	1,048,412.00	190,269.44	4,688,560.31	1,674,448.19	529,792.24	5,417,728.96
05019	Clark	Arkansas	22,995	2,783	2,054.00	3,536.00	1,203,024.01	613,176.53	2,269,468.63	2,033,272.41	471,904.49	8,622,705.57	3,236,296.42	1,212,682.84	10,521,155.68
05021	Clay	Arkansas	16,083	1,025	511.50	1,549.00	487,678.29	146,228.63	1,147,620.96	977,776.15	106,539.70	5,239,073.65	1,465,454.45	284,153.96	5,650,417.14
05023	Cleburne	Arkansas	25,970	2,528	1,769.00	3,278.50	1,319,018.06	674,740.69	2,381,547.20	2,287,928.87	579,868.24	6,783,001.56	3,606,946.93	1,462,392.66	8,389,360.45
05025	Cleveland	Arkansas	8,689	1,069	779.50	1,328.00	470,846.07	175,561.46	1,171,169.82	733,195.33	125,430.30	3,477,428.00	1,204,041.40	339,692.44	4,248,251.02
05027	Columbia	Arkansas	24,552	3,207	2,295.00	4,047.50	1,367,166.83	719,965.85	2,483,852.46	2,463,786.59	664,494.36	7,982,758.17	3,830,953.42	1,544,586.54	9,667,163.85
05029	Conway	Arkansas	21,273	2,404	1,775.00	3,123.00	1,084,668.70	529,705.77	2,060,078.88	1,939,756.20	439,508.49	7,662,080.99	3,024,424.90	1,083,403.90	8,687,060.39
05031	Craighead	Arkansas	96,443	7,068	3,817.00	10,070.50	2,783,391.42	1,231,729.69	4,870,996.27	4,614,845.17	1,316,587.22	13,516,167.08	7,398,236.58	2,815,748.64	17,335,021.41
05033	Crawford	Arkansas	61,948	7,070	4,845.00	9,070.50	2,875,875.66	1,602,059.70	4,743,836.05	4,726,041.75	1,474,757.73	13,588,312.17	7,601,917.41	3,456,578.81	16,970,413.87
05035	Crittenden	Arkansas	50,902	3,989	2,389.00	5,500.50	1,481,359.79	589,554.87	2,789,988.47	2,336,889.76	577,130.13	8,747,746.57	3,818,249.55	1,353,851.64	10,284,965.19
05037	Cross	Arkansas	17,870	1,471	940.50	2,027.00	651,159.08	242,812.75	1,484,351.37	1,033,843.55	185,246.75	4,321,514.03	1,685,002.64	484,791.26	5,990,868.91
05039	Dallas	Arkansas	8,116	1,000	704.50	1,282.50	472,811.29	180,744.51	1,119,243.80	803,430.72	149,809.11	3,510,220.69	1,276,242.01	365,150.74	4,231,101.62
05041	Desha	Arkansas	13,008	1,465	1,056.00	1,913.00	637,796.93	265,909.44	1,362,897.12	1,089,933.95	212,099.64	4,823,168.39	1,727,730.88	534,391.14	5,819,038.80
05043	Drew	Arkansas	18,509	2,160	1,577.50	2,727.00	897,230.94	427,667.54	1,768,923.15	1,560,838.25	310,066.47	6,669,935.53	2,458,069.19	845,452.76	7,786,416.62
05045	Faulkner	Arkansas	113,237	12,861	9,628.00	16,165.00	4,681,293.16	2,981,238.93	7,197,460.33	7,806,827.35	3,037,719.52	19,928,801.09	12,488,120.52	6,602,217.96	26,284,041.11
05047	Franklin	Arkansas	18,125	2,054	1,448.50	2,649.00	918,290.28	412,668.20	1,836,288.29	1,633,677.28	343,606.91	6,432,293.63	2,551,967.56	842,948.29	7,742,588.80

05049	Fulton	Arkansas	12,245	918	524.00	1,299.00	485,583.52	158,379.75	1,215,647.69	820,316.05	101,188.42	3,683,007.33	1,305,899.57	293,094.33	4,695,275.21
05051	Garland	Arkansas	96,024	11,556	8,473.00	14,574.00	5,722,603.54	3,639,704.47	8,506,462.15	10,730,855.27	4,651,060.84	22,678,815.31	16,453,458.81	8,728,115.84	29,904,576.74
05053	Grant	Arkansas	17,853	2,134	1,577.00	2,712.50	922,283.03	419,005.25	2,028,083.90	1,466,855.01	329,197.45	5,849,454.57	2,389,138.05	803,290.09	7,265,413.10
05055	Greene	Arkansas	42,090	2,876	1,601.50	4,207.50	1,196,035.58	467,073.83	2,376,688.77	1,960,787.50	397,239.07	6,875,785.91	3,156,823.08	991,831.47	8,224,127.71
05057	Hempstead	Arkansas	22,609	3,034	2,240.00	3,807.50	1,289,227.96	664,329.57	2,456,881.28	2,031,457.82	557,366.65	6,800,917.41	3,320,685.78	1,332,439.19	8,373,643.10
05059	Hot Spring	Arkansas	32,923	4,006	2,976.00	5,094.00	1,784,393.58	940,133.47	2,960,752.42	3,233,310.17	833,679.15	10,234,173.49	5,017,703.75	1,988,788.06	12,653,451.46
05061	Howard	Arkansas	13,789	1,818	1,339.50	2,313.50	783,902.11	355,765.55	1,663,460.45	1,309,490.15	265,754.40	5,388,847.57	2,093,392.25	694,852.33	6,534,284.80
05063	Independence	Arkansas	36,647	3,096	1,986.00	4,233.00	1,347,864.77	636,460.12	2,607,139.58	2,329,493.05	523,386.38	8,789,868.59	3,677,357.82	1,340,432.95	10,783,622.68
05065	Izard	Arkansas	13,696	1,103	695.50	1,518.00	609,281.41	224,184.23	1,490,722.42	1,126,629.66	152,794.38	4,634,164.48	1,735,911.06	420,790.55	5,658,629.48
05067	Jackson	Arkansas	17,997	1,457	896.50	1,992.50	656,430.17	266,103.15	1,468,485.15	1,067,357.71	213,984.13	4,153,267.69	1,723,787.88	535,017.69	5,208,768.75
05069	Jefferson	Arkansas	77,435	9,058	6,779.50	11,377.00	3,753,452.72	2,323,972.81	6,112,942.55	6,751,011.30	2,565,282.12	18,908,236.21	10,504,464.02	5,373,102.89	22,642,374.70
05071	Johnson	Arkansas	25,540	2,805	1,998.50	3,702.00	1,186,267.79	550,046.31	2,285,288.49	1,925,485.31	431,351.19	8,112,252.72	3,111,753.11	1,166,061.99	9,493,220.37
05073	Lafayette	Arkansas	7,645	1,020	744.00	1,281.50	468,132.17	193,102.48	1,004,359.92	811,092.04	145,194.03	3,437,479.99	1,279,224.21	388,166.23	3,983,984.04
05075	Lawrence	Arkansas	17,415	1,287	765.50	1,835.00	594,598.79	207,273.93	1,341,016.06	987,188.32	150,008.38	4,265,700.34	1,581,787.11	395,668.31	5,335,411.32
05077	Lee	Arkansas	10,424	894	574.00	1,224.50	395,473.17	129,930.54	985,734.77	678,181.00	103,790.98	3,293,509.36	1,073,654.16	269,080.16	3,920,982.33
05079	Lincoln	Arkansas	14,134	1,577	1,147.50	1,983.00	644,735.68	265,268.97	1,513,659.11	1,003,378.08	197,851.56	4,519,230.53	1,648,113.77	520,498.05	5,396,436.90
05081	Little River	Arkansas	13,171	1,783	1,304.50	2,236.00	825,365.34	373,574.11	1,711,930.14	1,613,112.86	452,664.79	5,746,271.79	2,438,478.20	899,168.46	6,973,820.81
05083	Logan	Arkansas	22,353	2,598	1,882.00	3,341.00	1,167,776.80	579,488.97	2,222,751.39	1,950,455.74	471,968.36	6,622,927.44	3,118,232.54	1,245,824.24	7,973,027.66
05085	Lonoke	Arkansas	68,356	7,929	5,868.00	9,990.50	2,977,563.90	1,754,567.74	4,841,594.82	4,743,719.28	1,536,652.73	12,386,432.88	7,721,283.18	3,665,784.17	15,787,649.73
05087	Madison	Arkansas	15,717	1,587	1,089.50	2,072.50	704,447.66	288,139.45	1,583,731.83	1,263,260.32	208,287.89	4,566,073.81	1,967,707.98	564,193.26	5,637,226.95
05089	Marion	Arkansas	16,653	1,394	894.50	1,885.00	792,968.84	312,624.29	1,751,548.36	1,474,278.58	227,356.22	6,620,483.33	2,267,247.43	614,403.73	7,452,206.48
05091	Miller	Arkansas	43,462	5,879	4,296.00	7,351.50	2,469,653.04	1,373,256.25	4,373,096.55	4,140,330.53	1,335,663.59	11,293,873.35	6,609,983.57	3,058,406.26	14,153,185.75
05093	Mississippi	Arkansas	46,480	3,293	1,788.00	4,773.50	1,311,736.17	545,983.76	2,698,497.52	2,181,936.73	473,282.64	8,211,745.46	3,493,672.90	1,114,155.58	9,802,461.59
05095	Monroe	Arkansas	8,149	803	533.50	1,078.00	383,081.04	128,668.83	962,457.27	681,842.95	93,768.10	3,230,890.12	1,064,923.99	250,114.64	3,549,207.16
05097	Montgomery	Arkansas	9,487	1,171	835.50	1,481.00	615,053.38	254,076.95	1,446,000.94	1,053,796.10	170,170.77	4,698,600.78	1,668,849.48	510,958.15	5,543,099.56
05099	Nevada	Arkansas	8,997	1,171	846.00	1,459.00	532,401.85	222,294.42	1,210,123.49	960,173.95	173,428.56	4,811,059.12	1,492,575.80	455,178.12	5,341,644.73
05101	Newton	Arkansas	8,330	820	543.00	1,082.00	413,533.11	151,328.55	1,077,626.45	774,207.66	99,676.48	3,827,508.31	1,187,740.77	291,038.93	4,609,589.51
05103	Ouachita	Arkansas	26,120	3,348	2,506.50	4,210.00	1,557,951.59	811,626.44	2,907,609.67	2,662,704.89	664,747.68	9,001,855.27	4,220,656.48	1,670,451.14	10,891,829.27
05105	Perry	Arkansas	10,445	1,204	869.50	1,528.00	541,884.97	210,679.81	1,220,194.83	905,630.88	177,187.07	4,103,698.03	1,447,515.85	438,932.12	4,707,378.08
05107	Phillips	Arkansas	21,757	2,135	1,445.00	2,816.50	885,719.50	396,202.83	1,820,315.95	1,445,462.39	302,044.97	5,696,853.83	2,331,181.89	779,729.50	7,102,263.38
05109	Pike	Arkansas	11,291	1,456	1,066.00	1,838.00	649,784.42	276,370.46	1,555,794.05	1,094,924.25	199,335.19	4,671,821.33	1,744,708.67	530,469.75	5,599,600.99
05111	Poinsett	Arkansas	24,583	1,867	1,087.50	2,557.50	812,461.16	308,573.07	1,782,884.15	1,435,498.53	251,508.65	6,043,951.19	2,247,959.70	642,533.77	6,949,689.35
05113	Polk	Arkansas	20,662	2,686	2,005.50	3,387.00	1,308,640.41	656,852.33	2,371,284.88	2,296,221.02	481,497.50	8,860,460.80	3,604,861.43	1,311,138.24	10,721,801.83
05115	Pope	Arkansas	61,754	6,725	4,842.50	8,609.50	2,719,867.05	1,532,656.59	4,666,703.18	4,450,602.58	1,471,840.77	13,240,972.24	7,170,469.63	3,459,049.59	16,193,697.69
05117	Prairie	Arkansas	8,715	897	642.50	1,179.50	434,280.68	166,526.63	936,552.89	906,718.10	122,324.85	3,799,962.63	1,340,998.78	321,865.29	4,772,938.77
05119	Pulaski	Arkansas	382,748	45,071	33,892.00	56,683.50	17,989,112.91	12,349,580.69	24,697,448.17	32,946,628.57	19,297,404.92	60,821,811.39	50,935,741.48	33,470,170.30	80,695,295.33
05121	Randolph	Arkansas	17,969	1,244	621.50	1,829.00	579,552.97	182,124.27	1,394,305.13	945,592.99	118,685.33	4,199,631.00	1,525,145.96	345,486.99	5,050,631.16
05123	St. Francis	Arkansas	28,258	2,372	1,533.00	3,276.00	958,782.99	414,971.67	1,948,105.81	1,580,874.30	334,405.30	6,236,322.95	2,539,657.29	836,108.09	7,547,310.43
05125	Saline	Arkansas	107,118	12,683	9,396.50	16,369.50	5,408,804.33	3,479,619.02	8,147,099.02	9,352,575.31	3,634,485.22	23,363,406.48	14,761,379.63	7,839,410.73	30,121,956.28
05127	Scott	Arkansas	11,233	1,405	1,038.00	1,791.00	643,252.57	267,285.74	1,438,042.76	1,026,328.11	182,377.47	5,051,315.36	1,669,580.67	517,515.46	6,026,070.77
05129	Searcy	Arkansas	8,195	775	546.50	1,012.50	400,452.88	144,994.82	1,009,297.31	737,257.59	94,786.27	3,411,353.60	1,137,710.47	269,639.58	3,891,643.27
05131	Sebastian	Arkansas	125,744	15,178	10,989.50	19,472.50	6,133,031.59	3,791,373.53	9,140,361.36	10,547,710.15	4,473,437.22	23,761,846.35	16,680,741.73	8,786,008.96	30,952,462.08
05133	Sevier	Arkansas	17,058	2,360	1,767.50	2,996.50	878,545.63	446,443.21	1,732,044.74	1,425,234.03	312,846.93	6,586,646.33	2,303,779.67	836,074.38	7,491,654.00
05135	Sharp	Arkansas	17,264	1,309	736.00	1,900.50	699,313.51	254,676.55	1,464,314.51	1,221,249.22	168,711.81	5,360,042.02	1,920,562.74	472,926.95	6,315,804.29
05137	Stone	Arkansas	12,394	1,132	725.50	1,537.50	611,636.96	215,228.52	1,552,176.05	1,094,871.14	152,543.90	5,296,659.29	1,706,508.10	420,952.39	6,239,254.30
05139	Union	Arkansas	41,639	5,310	3,872.50	6,770.00	2,335,426.42	1,283,591.48	4,014,577.85	4,192,240.86	1,434,919.32	11,356,153.73	6,527,667.28	2,902,527.44	14,664,932.65
05141	Van Buren	Arkansas	17,295	1,766	1,231.50	2,276.00	928,787.81	421,391.69	2,032,374.45	1,702,153.57	313,613.99	6,301,686.96	2,630,941.38	829,252.09	7,218,908.62
05143	Washington	Arkansas	203,065	21,493	14,855.00	28,224.00	7,670,291.08	4,734,345.82	11,491,346.01	12,900,668.60	5,620,862.82	29,448,719.72	20,570,959.69	10,834,588.97	39,034,490.30
05145	White	Arkansas	77,076	7,655	5,305.50	9,866.00	3,136,170.95	1,795,597.03	5,071,943.23	5,495,429.79	1,910,516.02	14,492,312.31	8,631,600.74	4,089,667.32	18,167,104.61
05147	Woodruff	Arkansas	7,260	656	440.00	888.50	301,375.04	91,033.18	801,857.71	563,663.54	82,092.90	3,187,200.03	865,038.58	195,111.37	3,653,181.77
05149	Yell	Arkansas	22,185	2,643	1,918.00	3,346.00	1,125,344.06	563,515.24	2,165,181.49	1,883,981.45	423,621.26	6,791,929.83	3,009,325.51	1,093,858.47	8,100,373.57
06001	Alameda	California	1,510,271	148,345	111,610.00	188,830.00	58,158,698.11	42,685,812.73	75,259,790.38	117,720,368.00	79,197,590.51	171,328,337.40	175,879,066.10	123,138,538.20	240,040,268.80
06003	Alpine	California	1,175	105	63.00	150.50	49,411.38	5,212.99	204,133.93	96,372.83	11,364.83	557,780.40	145,784.21	17,762.11	774,414.55
06005	Amador	California	38,091	3,349	2,265.50	4,526.50	1,787,495.09	887,452.01	3,144,203.74	3,397,638.69	953,582.84	10,165,399.67	5,185,133.78	2,069,408.80	12,306,322.42
06007	Butte	California	220,000	20,068	13,292.00	26,506.50	8,764,631.06	5,156,550.50	12,984,440.64	16,333,885.63	7,636,368.76	34,537,480.10	25,098,516.69	13,699,323.21	44,638,115.46
06009	Calaveras	California	45,578	4,048	2,671.00	5,449.50	2,190,131.72	1,067,695.38	3,899,960.81	3,918,362.64	1,179,171.38	11,179,678.06	6,108,494.36	2,497,933.17	13,704,029.89
06011	Colusa	California	21,419	2,101	1,398.00	2,759.50	803,392.45	350,891.17	1,769,283.82	1,362,434.56	337,342.06	4,994,627.30	2,165,827.01	731,473.28	6,033,814.93
06013	Contra Costa	California	1,049,025	103,316	74,859.00	130,632.00	41,763,100.93	29,643,149.62	54,596,841.06	84,395,148.07	54,613,580.41	122,314,704.60	126,158,249.00	86,246,328.61	173,470,027.30
06015	Del Norte	California	28,610	2,559	1,485.00	3,561.00	1,103,476.12	439,470.49	2,279,774.28	1,886,579.77	409,699.66	7,147,328.38	2,990,055.89	980,085.47	8,684,935.81
06017	El Dorado	California	181,058	16,184	10,348.50	21,572.00	7,309,605.96	4,352,305.30	11,014,403.88	13,234,543.14	5,605,187.20	27,515,348.46	20,544,149.09	10,573,165.10	36,468,310.40
06019	Fresno	California	930,450	87,815	60,798.50	114,944.00	30,948,400.43	20,934,412.48	41,969,564.36	52,319,005.03	30,411,798.55	86,023,553.70	83,267,405.45	52,868,866.19	122,968,658.90
06021	Glenn	California	28,122	2,695	1,765.00	3,678.50	1,076,096.37	489,691.99	2,166,237.77	1,900,546.05	461,502.10	6,210,450.09	2,976,642.43	1,004,724.13	7,663,181.07
06023	Humboldt	California	134,623	12,133	7,375.50	16,690.50	5,276,911.04	2,742,264.23	8,272,235.03	8,825,439.52	3,077,008.27	21,502,018.12	14,102,350.56	6,375,920.19	28,033,587.57
06025	Imperial	California	174,528	15,971	9,496.50	22,341.00	5,701,393.84	3,168,399.65	9,015,496.85	9,936,744.06	3,449,031.23	22,668,799.32	15,638,137.89	6,994,473.26	28,996,487.59
06027	Inyo	California	18,546	1,635	1,032.00	2,261.00	826,559.28	328,903.69	1,825,669.02	1,642,988.42	314,943.98	6,874,101.81	2,469,547.70	717,771.50	8,214,819.29
06029	Kern	California	839,631	78,410	50,062.50	104,075.00	26,808,745.68	16,812,831.06	36,928,642.58	46,141,903.07	24,844,889.98	75,038,954.43	72,950,648.75	43,539,901.12	108,615,326.60

06031	Kings	California	152,982	14,380	9,419.50	19,183.50	4,763,681.17	2,808,981.07	7,492,473.68	7,852,410.27	3,255,197.48	18,882,721.59	12,616,091.44	6,580,275.27	24,561,982.41
06033	Lake	California	64,665	6,118	4,099.00	8,327.50	2,935,287.94	1,589,694.93	4,853,733.44	5,610,361.52	1,644,953.55	16,291,595.54	8,545,649.46	3,654,520.86	19,771,720.61
06035	Lassen	California	34,895	2,816	1,698.00	3,890.00	1,118,367.72	439,543.77	2,345,933.36	1,767,176.79	466,304.70	6,517,768.12	2,885,544.51	1,034,529.68	8,736,190.79
06037	Los Angeles	California	9,818,605	868,588	610,092.00	1,118,760.00	327,106,978.80	228,009,968.90	421,057,992.30	630,381,859.30	436,425,296.80	835,611,329.80	957,488,838.10	664,735,330.00	1,248,181,845.00
06039	Madera	California	150,865	14,157	9,609.50	18,617.50	5,264,408.72	3,137,471.82	7,852,487.97	8,815,498.85	3,539,346.54	20,195,586.29	14,079,907.57	7,302,613.65	26,148,948.42
06041	Marin	California	252,409	24,829	18,019.50	31,824.00	11,850,453.25	7,899,321.28	16,912,317.39	24,228,343.76	12,632,370.95	41,411,562.53	36,078,797.01	21,601,580.04	55,404,588.96
06043	Mariposa	California	18,251	1,620	1,096.00	2,144.50	892,429.03	370,213.86	1,903,731.02	1,574,483.87	326,366.38	6,048,376.32	2,466,912.90	834,839.14	7,047,063.20
06045	Mendocino	California	87,841	8,393	5,420.00	11,218.50	3,831,341.01	2,220,833.34	6,210,942.81	6,775,064.04	2,385,568.02	18,023,831.59	10,606,405.05	5,028,175.14	22,464,585.95
06047	Merced	California	255,793	25,329	18,020.50	33,140.00	8,529,202.82	5,595,574.29	12,100,786.09	14,205,552.84	6,675,616.65	30,385,232.25	22,734,755.66	12,732,382.75	39,422,266.93
06049	Modoc	California	9,686	809	483.50	1,169.50	394,085.04	121,923.11	1,000,551.19	737,120.24	115,011.82	3,784,261.86	1,131,205.28	275,923.85	4,263,013.55
06051	Mono	California	14,202	1,231	793.00	1,678.00	498,515.80	179,166.17	1,167,511.40	898,133.93	163,139.12	3,914,467.93	1,396,649.73	371,414.82	4,772,335.61
06053	Monterey	California	415,057	40,292	26,932.00	53,717.50	14,952,349.86	9,422,713.62	20,941,205.21	26,674,574.68	13,356,571.41	48,378,492.13	41,626,924.54	23,644,704.19	65,751,149.16
06055	Napa	California	136,484	13,029	9,125.00	17,082.00	5,665,591.32	3,479,785.51	8,760,967.58	10,602,211.63	4,807,571.12	21,025,214.65	16,267,802.95	8,950,492.12	28,538,327.02
06057	Nevada	California	98,764	8,766	5,629.50	11,759.50	4,496,449.04	2,524,672.68	6,877,444.37	8,481,249.54	3,432,099.76	20,071,081.67	12,977,698.58	6,680,197.47	25,142,377.78
06059	Orange	California	3,010,232	265,693	187,347.00	346,883.00	102,933,964.80	72,301,523.79	135,290,329.90	199,705,618.10	132,641,050.70	276,981,881.00	302,639,582.90	206,151,906.30	403,500,659.20
06061	Placer	California	348,432	31,540	20,841.50	41,924.50	13,769,781.99	8,464,633.62	20,097,729.27	26,653,727.41	13,860,662.02	48,586,372.02	40,423,509.40	23,453,926.19	64,840,524.13
06063	Plumas	California	20,007	1,714	1,097.00	2,348.50	954,347.61	396,499.67	2,084,681.33	1,910,140.09	387,383.92	6,380,588.70	2,864,487.70	909,675.69	7,669,756.78
06065	Riverside	California	2,189,641	199,069	128,019.00	277,305.00	74,302,309.82	46,718,486.19	103,536,516.20	132,431,014.10	78,046,193.94	199,874,387.90	206,733,323.90	128,424,232.20	299,538,104.90
06067	Sacramento	California	1,418,788	133,126	92,495.00	175,090.00	50,962,950.86	34,859,277.21	68,328,434.63	96,892,933.10	58,715,201.60	147,627,670.00	147,855,884.00	96,050,381.21	208,401,943.00
06069	San Benito	California	55,269	5,324	3,725.00	6,911.50	1,919,331.30	1,023,224.78	3,325,922.85	3,259,969.84	1,041,791.05	9,982,362.05	5,179,301.14	2,241,155.45	12,003,340.40
06071	San Bernardino	California	2,035,210	185,165	115,737.00	255,951.00	64,017,009.37	39,266,145.17	90,232,377.74	109,195,054.80	62,130,256.12	163,879,039.50	173,212,064.20	105,031,601.80	245,054,864.90
06073	San Diego	California	3,095,313	271,334	176,571.00	360,267.00	104,854,331.10	68,034,423.90	139,575,149.00	198,767,206.50	120,687,796.00	283,097,511.40	303,621,537.70	191,097,101.40	411,534,014.80
06075	San Francisco	California	805,235	74,834	55,004.00	95,527.50	32,792,097.38	22,931,352.86	43,386,699.55	74,799,539.62	48,196,379.34	110,217,231.00	107,591,637.00	71,867,098.52	152,601,790.10
06077	San Joaquin	California	685,306	67,278	46,778.00	87,742.50	24,309,694.27	16,148,005.14	33,227,360.12	41,882,188.51	24,215,578.66	68,362,555.26	66,191,882.78	41,130,811.23	97,993,557.29
06079	San Luis Obispo	California	269,637	24,013	15,730.00	32,151.00	10,981,676.62	6,672,127.65	16,048,343.23	20,416,980.17	9,098,026.94	38,888,489.35	31,398,656.80	17,007,575.61	52,906,111.40
06081	San Mateo	California	718,451	69,367	50,646.50	88,473.50	29,148,329.58	20,075,422.10	38,224,074.76	66,864,377.72	41,696,677.35	103,970,276.00	96,012,707.30	62,565,855.20	139,121,890.90
06083	Santa Barbara	California	423,895	38,564	23,920.00	53,836.50	15,381,926.62	9,121,021.90	22,101,036.50	28,735,027.05	14,199,099.79	51,340,476.27	44,116,953.67	24,066,782.74	71,264,575.60
06085	Santa Clara	California	1,781,642	171,315	124,470.00	218,624.00	65,518,281.32	46,590,673.46	86,945,955.25	150,825,135.40	101,128,735.10	207,237,184.30	216,343,416.70	151,803,171.50	287,353,853.60
06087	Santa Cruz	California	262,382	24,740	17,318.50	31,671.00	10,029,246.06	6,637,666.60	14,301,084.41	17,866,899.75	9,137,318.39	36,770,538.98	27,896,145.80	16,748,009.93	47,086,340.65
06089	Shasta	California	177,223	15,773	10,150.50	22,250.50	7,307,966.65	4,120,721.60	11,386,471.09	13,113,663.62	5,296,172.55	26,231,221.45	20,421,630.27	10,509,108.12	35,205,697.77
06091	Sierra	California	3,240	281	182.00	382.00	149,739.98	29,124.65	516,624.70	325,619.64	39,586.14	1,876,582.85	475,359.62	75,330.83	2,145,550.61
06093	Siskiyou	California	44,900	3,893	2,366.00	5,583.50	1,993,019.16	916,503.20	3,800,113.99	3,623,052.45	924,814.89	10,319,786.57	5,616,071.61	2,095,786.59	13,130,774.51
06095	Solano	California	413,344	39,738	28,651.00	50,884.00	15,775,590.38	10,703,011.43	21,876,027.89	29,050,902.96	16,749,607.09	52,072,755.07	44,826,493.33	28,181,064.06	70,191,815.91
06097	Sonoma	California	483,878	46,894	32,706.50	60,324.50	20,362,384.91	13,254,788.35	28,052,342.77	37,394,384.62	20,183,296.71	61,978,489.55	57,756,769.53	34,585,406.05	86,298,153.23
06099	Stanislaus	California	514,453	49,472	33,738.00	63,299.00	18,123,888.02	11,601,436.74	25,628,160.33	31,251,687.34	16,407,924.00	55,596,465.49	49,375,575.36	29,448,459.68	76,051,217.94
06101	Sutter	California	94,737	9,117	6,146.00	12,054.50	3,556,750.29	2,043,651.69	5,631,007.54	6,225,721.19	2,250,564.21	16,536,225.64	9,782,471.48	4,751,645.07	20,955,034.46
06103	Tehama	California	63,463	5,871	3,637.00	8,011.00	2,559,351.61	1,293,992.31	4,382,540.80	4,590,817.69	1,450,191.04	12,545,767.33	7,150,169.30	3,199,778.95	15,297,220.20
06105	Trinity	California	13,786	1,210	713.50	1,712.50	630,641.49	241,387.33	1,285,877.99	1,166,881.68	203,426.02	4,675,009.67	1,797,523.16	528,160.04	5,518,518.87
06107	Tulare	California	442,179	42,596	27,906.00	57,652.50	14,397,186.38	9,185,463.14	20,709,174.01	23,283,314.54	10,890,840.09	45,547,302.38	37,680,500.92	21,269,229.64	63,248,261.00
06109	Tuolumne	California	55,365	4,880	3,139.00	6,643.00	2,597,165.82	1,330,313.30	4,450,363.67	4,980,220.71	1,379,793.91	14,838,231.11	7,577,386.53	3,073,699.39	18,726,530.97
06111	Ventura	California	823,318	74,110	48,803.50	100,084.00	28,880,203.16	18,330,566.39	40,241,456.73	54,506,026.77	30,456,363.24	88,661,385.52	83,386,229.93	51,750,436.77	124,368,527.00
06113	Yolo	California	200,849	18,600	13,127.50	24,411.50	6,814,625.52	4,252,897.03	10,187,754.16	12,319,619.64	5,604,469.24	25,577,072.72	19,134,245.15	10,646,693.67	33,791,159.63
06115	Yuba	California	72,155	6,807	4,521.00	9,228.00	2,438,460.93	1,321,947.78	3,962,431.96	4,372,609.97	1,572,247.27	12,743,267.01	6,811,070.90	3,199,661.35	15,339,508.43
08001	Adams	Colorado	441,603	21,002	5,576.50	36,851.00	7,166,095.96	1,730,684.77	13,495,126.74	12,164,986.71	2,368,172.49	29,151,126.24	19,331,082.67	4,413,977.81	39,611,039.38
08003	Alamosa	Colorado	15,445	1,197	621.00	1,720.00	464,583.17	140,949.10	1,094,572.12	871,296.71	123,410.06	4,302,182.87	1,335,879.88	290,499.16	4,948,966.93
08005	Arapahoe	Colorado	572,003	27,187	9,603.00	44,964.00	10,345,918.44	3,473,948.97	18,186,705.02	19,562,314.10	5,587,399.89	38,563,547.53	29,908,232.53	9,175,152.33	53,717,139.04
08007	Archuleta	Colorado	12,084	987	582.50	1,405.50	516,145.72	155,642.99	1,326,522.14	931,798.37	130,972.12	3,582,800.76	1,447,944.10	324,385.52	4,361,780.79
08009	Baca	Colorado	3,788	305	167.00	442.00	168,718.14	30,215.91	628,853.66	256,266.75	30,120.76	1,547,031.67	424,984.89	68,386.29	1,877,956.30
08011	Bent	Colorado	6,499	417	203.50	609.50	174,963.18	34,569.35	553,791.27	297,638.89	40,521.41	1,935,023.73	472,602.07	85,963.92	2,208,933.61
08013	Boulder	Colorado	294,567	12,216	3,637.00	21,015.00	4,782,823.40	1,204,409.97	8,875,091.79	8,965,953.53	1,814,330.47	21,776,429.36	13,748,776.93	3,467,474.43	28,859,910.23
08015	Chaffee	Colorado	17,809	1,027	482.00	1,609.00	529,859.40	155,718.00	1,222,910.31	974,834.55	118,308.51	4,441,466.09	1,504,693.95	309,193.06	5,318,517.64
08017	Cheyenne	Colorado	1,836	118	49.00	185.50	50,269.12	4,471.15	213,479.02	102,229.94	9,846.69	676,684.15	152,499.07	15,755.29	843,328.88
08019	Clear Creek	Colorado	9,088	396	135.50	653.00	186,394.76	26,390.58	628,648.55	358,440.47	29,917.42	2,279,608.27	544,835.23	67,064.74	2,569,796.47
08021	Conejos	Colorado	8,256	701	394.00	1,033.00	301,192.02	79,057.93	838,203.30	478,969.85	67,422.91	2,414,016.85	780,161.87	158,543.37	2,841,725.76
08023	Costilla	Colorado	3,524	285	161.00	418.50	152,130.44	26,225.43	502,372.24	279,902.10	32,121.75	1,693,540.43	432,032.54	64,458.50	1,991,120.59
08025	Crowley	Colorado	5,823	324	160.00	495.50	140,990.31	22,522.91	518,690.59	216,430.68	29,368.01	1,611,156.78	357,420.99	57,764.40	1,837,096.10
08027	Custer	Colorado	4,255	270	130.00	423.00	168,958.47	22,329.30	615,107.29	281,655.43	28,230.56	1,930,548.63	450,613.89	55,246.00	2,454,709.00
08029	Delta	Colorado	30,952	1,970	929.50	3,075.50	993,876.96	320,185.38	2,084,985.06	1,804,950.64	275,148.79	6,174,851.17	2,798,827.60	679,001.15	7,195,238.43
08031	Denver	Colorado	600,158	26,147	9,443.00	42,906.50	9,818,532.71	3,164,344.06	17,097,340.48	19,224,794.68	5,818,301.93	41,210,354.81	29,043,327.39	9,353,209.08	55,005,971.84
08033	Dolores	Colorado	2,064	158	75.00	242.00	82,986.34	9,050.56	348,755.08	122,308.72	12,576.61	805,983.10	205,295.06	24,681.19	1,048,836.71
08035	Douglas	Colorado	285,465	13,485	5,658.50	21,835.50	4,523,708.15	1,699,491.42	8,059,227.28	8,310,293.98	2,418,014.18	19,173,526.99	12,834,002.13	4,590,009.08	26,302,314.66
08037	Eagle	Colorado	52,197	2,603	802.50	4,348.00	857,638.52	193,168.18	1,998,025.98	1,450,834.90	192,554.06	5,191,705.87	2,308,473.42	463,562.14	6,449,812.75
08039	Elbert	Colorado	23,086	1,131	412.00	1,901.00	467,641.40	99,179.04	1,294,803.42	756,667.30	90,106.30	4,330,651.08	1,224,308.70	215,137.83	4,856,864.50
08041	El Paso	Colorado	622,263	32,498	14,815.00	50,476.00	12,053,162.90	5,282,856.81	19,610,629.80	21,398,324.94	7,151,043.51	41,887,223.35	33,451,487.84	12,626,592.84	57,509,344.53
08043	Fremont	Colorado	46,824	2,744	1,253.00	4,147.00	1,318,380.74	434,670.28	2,677,775.67	2,286,125.15	434,631.63	7,004,515.36	3,604,505.89	1,081,185.48	9,166,010.67
08045	Garfield	Colorado	56,389	3,147	1,046.00	5,245.50	1,128,955.53	252,998.66	2,508,400.75	1,840,059.37	306,296.95	6,983,697.93	2,969,014.89	585,183.24	8,504,464.54

08047	Gilpin	Colorado	5,441	228	65.00	374.50	104,456.66	6,733.66	474,904.74	163,804.36	14,288.03	1,046,290.11	268,261.02	22,802.92	1,240,123.84
08049	Grand	Colorado	14,843	641	187.00	1,113.00	280,035.60	34,013.13	892,373.01	513,543.37	33,651.76	2,678,103.05	793,578.97	70,562.75	3,288,581.46
08051	Gunnison	Colorado	15,324	930	414.50	1,436.50	355,659.95	81,285.78	923,778.33	591,567.60	78,239.02	3,252,831.30	947,227.56	174,915.09	3,593,255.24
08053	Hinsdale	Colorado	843	65	31.00	101.00	31,014.43	2,466.58	192,056.10	54,887.44	3,964.17	439,161.04	85,901.87	7,132.12	540,140.67
08055	Huerfano	Colorado	6,711	471	256.00	695.00	277,162.71	60,888.93	824,730.21	488,354.68	61,911.40	2,474,350.89	765,517.38	148,812.04	2,890,795.63
08057	Jackson	Colorado	1,394	57	8.50	108.00	24,887.86	468.27	150,856.23	32,639.44	1,473.86	241,804.73	57,527.30	2,180.61	316,749.81
08059	Jefferson	Colorado	534,543	23,388	8,262.50	38,547.50	9,897,335.16	3,072,839.51	17,198,460.29	18,682,650.90	5,343,954.92	38,057,694.94	28,579,986.05	8,905,760.52	54,811,540.17
08061	Kiowa	Colorado	1,398	92	43.00	145.00	45,449.39	4,028.02	194,885.86	80,722.24	7,746.57	535,334.96	126,171.64	12,999.28	654,818.67
08063	Kit Carson	Colorado	8,270	490	179.50	806.50	215,830.57	32,478.56	696,898.58	360,510.08	35,262.06	2,044,908.02	576,340.65	75,504.84	2,266,693.15
08065	Lake	Colorado	7,310	392	138.00	643.00	146,999.81	18,581.45	473,179.29	230,080.01	21,217.98	1,890,071.61	377,079.83	44,646.16	2,106,567.75
08067	La Plata	Colorado	51,334	4,053	2,273.50	5,924.00	1,723,873.16	712,331.46	3,384,735.61	2,948,378.65	726,770.44	10,709,870.31	4,672,251.81	1,675,799.00	12,804,420.10
08069	Larimer	Colorado	299,630	11,936	2,090.00	21,821.50	4,775,280.55	627,680.65	9,487,150.90	8,655,475.18	881,407.75	24,864,920.09	13,430,755.72	1,823,721.35	31,774,372.09
08071	Las Animas	Colorado	15,507	1,208	714.00	1,711.00	580,040.61	205,429.67	1,359,554.14	1,155,276.76	172,083.03	5,632,933.38	1,735,317.37	392,889.38	6,494,059.93
08073	Lincoln	Colorado	5,467	305	126.50	493.00	139,450.30	20,020.07	503,315.71	247,937.99	26,320.25	1,648,714.95	387,388.30	54,114.71	1,839,939.94
08075	Logan	Colorado	22,709	1,124	196.50	1,954.00	488,677.98	56,574.66	1,322,700.58	814,856.93	52,771.74	4,059,111.54	1,303,534.91	111,601.82	4,840,997.57
08077	Mesa	Colorado	146,723	8,996	3,975.00	14,130.00	3,885,179.65	1,503,727.60	6,713,732.51	7,070,150.59	1,773,742.90	17,215,375.37	10,955,330.23	3,598,586.39	22,668,922.78
08079	Mineral	Colorado	712	52	26.00	80.50	29,214.74	2,005.98	153,107.91	59,854.67	4,314.43	384,489.75	89,069.41	6,884.63	572,642.30
08081	Moffat	Colorado	13,795	675	150.50	1,165.00	268,158.66	31,892.86	769,822.88	419,230.62	34,025.29	2,175,227.61	687,389.28	73,203.36	2,778,589.59
08083	Montezuma	Colorado	25,535	2,049	1,169.50	2,947.00	943,030.37	371,608.36	1,862,274.27	1,675,524.52	301,097.07	6,197,702.91	2,618,554.89	805,172.33	7,671,838.77
08085	Montrose	Colorado	41,276	2,837	1,353.00	4,334.00	1,328,827.24	434,613.70	2,581,347.49	2,452,917.60	433,719.66	8,652,337.48	3,781,744.84	1,019,887.43	10,690,996.36
08087	Morgan	Colorado	28,159	1,434	388.50	2,532.00	576,450.50	94,565.12	1,416,875.64	987,913.06	87,933.28	4,600,473.99	1,564,363.56	197,722.97	5,493,522.70
08089	Otero	Colorado	18,831	1,300	664.50	1,906.00	575,538.64	189,546.68	1,313,894.16	1,050,830.94	150,281.93	4,309,921.72	1,626,369.58	365,408.31	5,021,820.35
08091	Ouray	Colorado	4,436	302	148.50	469.50	175,884.94	26,924.25	645,656.16	298,729.64	32,357.24	2,017,903.43	474,614.58	64,015.49	2,400,588.22
08093	Park	Colorado	16,206	803	303.00	1,308.50	383,567.24	63,373.40	1,063,278.81	615,946.89	53,692.82	3,612,057.53	999,514.14	131,755.56	4,152,580.87
08095	Phillips	Colorado	4,442	244	57.50	425.50	114,092.42	8,851.35	391,918.47	210,517.46	11,209.82	1,519,053.25	324,609.88	21,635.82	1,729,005.87
08097	Pitkin	Colorado	17,148	901	331.50	1,475.50	388,743.23	66,466.26	1,092,127.88	813,794.40	94,299.03	3,666,301.66	1,202,537.63	167,408.27	4,285,727.13
08099	Prowers	Colorado	12,551	947	486.50	1,398.00	390,526.70	111,194.76	937,966.23	737,128.72	99,258.96	3,789,082.87	1,127,655.42	228,583.82	4,492,589.03
08101	Pueblo	Colorado	159,063	10,076	5,248.00	15,160.50	4,401,553.22	2,057,242.81	7,560,513.48	7,831,741.27	2,629,041.05	20,858,225.26	12,233,294.48	5,057,578.48	26,041,765.57
08103	Rio Blanco	Colorado	6,666	348	107.00	602.50	139,527.80	16,097.52	529,049.82	275,776.25	31,736.82	1,784,041.30	415,304.05	52,974.82	1,973,501.39
08105	Rio Grande	Colorado	11,982	946	524.00	1,386.00	447,588.96	122,811.05	1,158,689.29	723,820.88	110,668.58	3,280,837.75	1,171,409.84	264,206.61	4,133,353.63
08107	Routt	Colorado	23,509	991	191.50	1,790.00	393,924.83	46,231.22	1,194,180.99	674,532.58	48,917.16	3,409,730.40	1,068,457.41	114,799.19	4,103,826.69
08109	Saguache	Colorado	6,108	430	218.00	649.50	189,709.85	39,153.04	633,009.03	352,939.89	40,170.12	1,783,004.26	542,649.73	85,134.91	2,104,516.09
08111	San Juan	Colorado	699	51	25.00	78.00	23,788.49	1,453.81	141,987.62	32,160.98	2,790.62	237,577.18	55,949.47	4,905.97	356,891.78
08113	San Miguel	Colorado	7,359	511	250.00	757.00	204,008.43	38,542.34	710,490.79	326,269.45	48,879.82	1,692,684.20	530,277.87	97,777.32	2,096,726.76
08115	Sedgwick	Colorado	2,379	126	27.50	224.50	75,780.08	3,811.84	311,331.24	166,690.31	5,638.09	1,104,003.57	242,470.39	10,664.34	1,301,076.10
08117	Summit	Colorado	27,994	1,269	368.50	2,173.00	490,146.32	87,755.21	1,222,408.30	782,456.76	71,119.77	4,094,280.17	1,272,603.08	181,636.98	4,715,549.74
08119	Teller	Colorado	23,350	1,186	480.00	1,865.00	586,587.47	147,509.79	1,521,871.79	938,124.49	138,694.57	4,312,486.37	1,524,711.96	323,355.72	5,120,096.36
08121	Washington	Colorado	4,814	259	76.50	448.00	126,936.84	10,824.66	541,056.61	191,230.28	15,109.50	1,197,351.33	318,167.12	29,664.26	1,512,124.50
08123	Weld	Colorado	252,825	12,118	3,179.00	21,462.00	4,407,782.70	1,090,356.40	8,712,831.17	7,186,029.26	1,090,089.53	18,256,098.29	11,593,811.96	2,321,865.18	26,237,105.81
08125	Yuma	Colorado	10,043	583	179.50	978.50	245,258.50	39,512.64	724,404.59	439,348.40	39,722.67	2,038,364.01	684,606.90	91,295.24	2,361,175.65
09001	Fairfield	Connecticut	916,829	63,178	34,815.00	91,133.00	26,124,467.53	13,711,014.95	39,120,446.17	59,009,596.26	28,670,249.00	96,282,080.59	85,134,063.78	43,382,411.72	130,380,798.20
09003	Hartford	Connecticut	894,014	56,327	29,056.50	82,671.50	24,238,157.30	11,799,393.33	36,768,985.71	49,852,899.80	22,894,705.87	84,839,486.09	74,091,057.09	35,658,980.50	119,396,081.80
09005	Litchfield	Connecticut	189,927	12,637	7,230.00	18,324.00	5,871,915.31	3,015,962.36	9,427,440.80	11,012,668.86	4,137,006.98	23,454,876.14	16,884,584.16	7,669,001.09	30,802,837.09
09007	Middlesex	Connecticut	165,676	9,806	4,656.00	14,759.00	4,534,976.87	1,880,347.74	7,713,632.35	8,515,527.05	2,830,130.52	19,419,047.06	13,050,503.91	5,024,931.65	24,868,662.59
09009	New Haven	Connecticut	862,477	55,171	28,881.50	82,103.50	23,620,905.80	11,553,167.02	36,812,779.93	45,935,713.81	20,923,955.37	81,033,929.15	69,556,619.61	33,748,601.93	115,445,368.00
09011	New London	Connecticut	274,055	15,104	6,989.50	23,353.00	6,537,005.54	2,720,238.43	11,035,503.10	12,366,856.17	4,178,115.40	25,902,490.99	18,903,861.72	7,366,720.06	35,558,320.31
09013	Tolland	Connecticut	152,691	8,568	4,280.50	12,578.00	3,544,441.95	1,650,589.73	6,101,480.27	6,580,672.30	1,824,758.04	18,127,605.30	10,125,114.25	3,694,247.67	22,317,310.40
09015	Windham	Connecticut	118,428	6,248	2,737.50	9,542.00	2,617,391.05	959,282.12	4,632,210.92	4,534,453.90	1,085,002.11	13,008,166.46	7,151,844.95	2,108,707.00	16,355,010.27
10001	Kent	Delaware	162,310	15,775	10,816.00	20,517.50	6,403,312.62	3,915,012.31	9,385,452.92	11,390,633.98	4,901,835.26	24,349,647.58	17,793,946.60	9,464,588.33	32,159,421.73
10003	New Castle	Delaware	538,479	51,096	35,695.50	66,229.50	20,644,861.34	13,609,160.85	28,491,737.45	39,873,279.25	22,812,482.74	66,345,887.86	60,518,101.30	38,282,647.45	91,341,840.61
10005	Sussex	Delaware	197,145	18,858	12,704.50	24,760.50	9,571,401.71	5,710,582.03	14,326,550.14	17,722,203.20	7,970,150.58	35,792,380.00	27,293,604.91	14,853,231.81	46,188,970.47
11001	Washington	strict of Columb	601,723	57,940	44,245.50	72,064.50	23,091,546.09	16,947,152.59	30,626,995.02	51,764,479.50	33,241,239.82	79,637,828.96	74,856,025.59	51,287,952.87	106,644,812.90
12001	Alachua	Florida	247,336	15,676	9,541.00	21,915.00	6,106,765.38	3,226,567.15	9,288,559.58	10,572,546.98	3,843,166.08	24,593,489.17	16,679,312.35	7,619,418.49	32,746,237.58
12003	Baker	Florida	27,115	1,826	1,061.00	2,653.00	695,474.35	272,843.74	1,572,577.95	1,167,105.20	209,858.88	5,500,925.59	1,862,579.54	569,495.90	6,414,816.63
12005	Bay	Florida	168,852	12,947	7,216.00	19,163.50	5,579,588.32	2,783,046.30	9,138,626.06	9,895,363.91	3,423,775.09	22,478,854.50	15,474,952.23	6,471,927.61	29,072,078.96
12007	Bradford	Florida	28,520	1,839	1,005.00	2,617.50	791,116.86	289,272.34	1,697,597.27	1,443,314.00	260,010.70	5,632,999.88	2,234,430.86	617,861.38	7,000,369.58
12009	Brevard	Florida	543,376	31,308	16,052.00	46,774.50	15,850,104.84	7,729,249.35	25,189,324.37	31,317,515.15	12,549,057.40	57,874,274.70	47,167,619.99	21,035,832.39	78,904,316.55
12011	Broward	Florida	1,748,066	89,861	39,468.00	137,440.00	38,307,467.41	16,233,160.71	59,503,422.34	72,106,322.95	28,210,847.40	122,535,065.70	110,414,290.40	44,420,179.79	179,730,251.20
12013	Calhoun	Florida	14,625	1,070	581.50	1,555.00	476,947.24	142,770.67	1,189,360.64	802,454.15	117,483.80	4,118,921.61	1,279,401.39	286,991.42	4,905,818.48
12015	Charlotte	Florida	159,978	8,976	4,155.50	13,649.00	5,868,158.18	2,480,632.50	9,499,252.56	11,932,045.38	3,703,337.42	26,007,300.30	17,800,203.56	6,267,808.33	33,785,972.74
12017	Citrus	Florida	141,236	8,820	4,728.50	12,788.50	5,579,762.01	2,680,400.96	9,199,962.88	11,517,903.15	3,940,570.64	25,724,239.97	17,097,665.17	7,250,781.85	33,084,017.10
12019	Clay	Florida	190,865	12,232	6,795.00	17,310.50	4,767,754.62	2,130,530.44	7,737,964.99	8,196,631.09	2,442,927.08	21,542,969.01	12,964,385.71	5,114,714.07	27,243,916.29
12021	Collier	Florida	321,520	17,906	7,452.00	28,400.00	9,709,263.82	3,858,131.58	16,129,544.57	20,401,069.87	6,133,223.60	41,601,563.03	30,110,333.69	10,387,617.68	54,470,078.70
12023	Columbia	Florida	67,531	4,435	2,434.50	6,426.00	1,960,638.23	843,356.51	3,604,659.52	3,540,337.55	884,096.56	10,126,451.51	5,500,975.78	1,991,831.90	12,279,551.87
12027	De Soto	Florida	34,862	1,993	965.50	3,110.50	906,922.71	299,301.48	1,913,892.20	1,610,863.87	276,497.90	6,075,517.15	2,517,786.58	639,129.13	7,382,876.79
12029	Dixie	Florida	16,422	1,080	571.50	1,575.50	535,521.94	161,351.30	1,318,811.79	1,010,201.38	138,590.92	5,234,350.00	1,545,723.31	316,751.22	5,762,202.06

12031	Duval	Florida	864,263	53,276	27,939.00	78,410.00	20,849,428.16	10,955,415.51	32,729,306.76	37,913,336.30	16,792,919.66	69,004,155.78	58,762,764.46	28,963,230.97	95,864,465.47
12033	Escambia	Florida	297,619	25,689	14,926.50	36,809.50	11,046,298.33	6,119,547.01	16,626,408.45	20,268,118.80	8,596,352.85	39,637,671.35	31,314,417.13	15,455,887.40	54,993,412.26
12035	Flagler	Florida	95,696	5,767	3,123.50	8,424.00	3,128,540.12	1,390,765.10	5,645,077.76	5,960,960.47	1,597,370.44	15,202,412.78	9,089,500.59	3,346,194.35	19,773,282.40
12037	Franklin	Florida	11,549	832	468.00	1,189.00	401,146.93	127,768.07	983,770.56	702,921.01	102,327.17	4,086,783.25	1,104,067.94	254,109.60	4,678,737.72
12039	Gadsden	Florida	46,389	3,379	2,037.50	4,714.00	1,415,835.10	636,385.88	2,719,875.96	2,560,456.67	592,686.59	8,771,186.75	3,976,291.77	1,344,820.74	10,073,018.20
12041	Gilchrist	Florida	16,939	1,105	608.00	1,575.00	503,322.42	163,922.62	1,140,303.99	966,538.58	140,361.69	4,010,884.59	1,469,861.00	337,341.38	4,851,813.78
12043	Glades	Florida	12,884	705	312.50	1,078.50	340,764.02	79,763.18	880,814.29	747,888.76	76,592.53	3,270,260.41	1,088,652.78	179,704.34	3,915,194.15
12045	Gulf	Florida	15,863	1,122	589.00	1,674.00	519,713.00	177,204.00	1,208,197.47	941,886.71	158,290.43	4,438,182.14	1,461,599.71	370,766.36	5,000,199.64
12047	Hamilton	Florida	14,799	972	516.50	1,420.50	412,156.54	125,952.77	1,037,135.60	846,173.71	156,168.61	3,738,211.32	1,258,330.26	317,643.83	4,168,334.86
12049	Hardee	Florida	27,731	1,652	823.00	2,519.00	646,392.07	195,501.64	1,486,061.12	1,035,564.78	169,826.27	4,878,964.05	1,681,956.85	397,413.73	5,612,919.37
12051	Hendry	Florida	39,140	2,209	973.00	3,467.50	821,289.75	221,462.57	1,812,126.69	1,325,867.67	180,270.58	5,390,544.58	2,147,157.42	465,635.59	6,473,245.89
12053	Hernando	Florida	172,778	10,576	5,180.00	15,627.00	5,797,988.11	2,511,038.63	9,655,821.43	11,278,050.90	3,570,741.72	24,473,650.94	17,076,039.00	6,563,017.65	32,093,749.13
12055	Highlands	Florida	98,786	5,531	2,383.00	8,694.00	3,333,788.01	1,255,305.24	6,120,400.98	6,679,064.98	1,534,677.23	17,111,775.17	10,012,852.99	2,960,564.49	20,811,097.46
12057	Hillsborough	Florida	1,229,226	71,409	37,055.00	105,825.00	27,905,625.46	13,880,427.37	41,661,274.65	51,462,417.54	22,304,406.69	91,109,325.16	79,368,043.00	37,186,204.61	129,071,593.00
12059	Holmes	Florida	19,927	1,527	846.50	2,219.00	688,217.22	242,466.27	1,551,039.44	1,174,580.42	174,592.01	4,217,381.70	1,862,797.64	486,033.76	5,334,839.55
12061	Indian River	Florida	138,028	7,667	3,292.00	11,772.00	4,348,179.78	1,648,625.70	7,353,258.39	8,779,395.28	2,304,192.60	21,545,734.47	13,127,575.05	4,150,693.94	26,349,652.33
12063	Jackson	Florida	49,746	3,626	2,044.50	5,194.50	1,636,565.84	714,257.03	2,985,103.60	2,869,433.87	655,242.77	9,707,646.03	4,505,999.71	1,575,871.92	11,604,518.79
12065	Jefferson	Florida	14,761	989	552.00	1,422.00	484,458.93	147,593.61	1,223,739.08	856,414.08	128,851.75	4,454,144.43	1,340,873.01	315,317.18	5,100,465.55
12067	Lafayette	Florida	8,870	571	303.50	829.00	219,596.04	55,089.71	627,423.55	363,764.58	55,208.64	2,226,241.14	583,360.62	117,685.87	2,516,858.38
12069	Lake	Florida	297,052	17,941	9,280.00	26,771.00	9,404,315.23	4,489,333.70	15,057,304.75	17,799,278.74	6,565,183.57	34,559,624.10	27,203,593.97	11,861,660.58	47,851,332.18
12071	Lee	Florida	618,754	35,873	15,745.50	54,612.50	18,754,326.96	7,522,765.31	29,533,268.59	37,757,692.05	13,281,810.11	67,828,532.35	56,512,019.01	21,420,842.25	95,568,625.37
12073	Leon	Florida	275,487	18,720	11,288.00	26,024.50	6,942,189.16	3,751,711.10	10,414,662.53	11,898,037.64	4,902,599.13	24,152,414.37	18,840,226.80	9,272,454.73	32,412,950.56
12075	Levy	Florida	40,801	2,617	1,428.50	3,792.00	1,288,363.30	492,720.20	2,554,483.11	2,435,231.30	414,123.63	8,561,917.36	3,723,594.60	1,009,673.35	9,879,605.79
12077	Liberty	Florida	8,365	592	336.00	868.00	238,182.40	57,002.51	733,178.40	427,231.07	69,475.50	2,384,878.75	665,413.47	137,064.27	2,700,936.47
12079	Madison	Florida	19,224	1,284	712.00	1,849.00	563,418.68	177,117.49	1,317,761.80	925,094.12	157,902.62	3,999,357.29	1,488,512.80	378,897.80	4,804,765.42
12081	Manatee	Florida	322,833	18,932	9,357.00	28,762.50	9,863,609.84	4,407,058.00	15,857,136.79	19,356,114.81	7,213,250.26	35,966,524.77	29,219,724.65	12,165,727.66	49,336,437.37
12083	Marion	Florida	331,298	21,069	11,866.50	30,063.50	11,449,165.15	6,445,185.68	17,453,792.66	22,371,761.24	9,205,926.01	45,266,446.39	33,820,926.39	16,131,384.84	59,588,445.13
12085	Martin	Florida	146,318	7,842	3,599.00	12,309.50	4,480,380.37	1,781,187.39	7,837,955.66	9,196,164.73	2,496,251.68	20,340,629.68	13,676,545.10	4,708,850.42	26,219,187.35
12086	Dade	Florida	2,496,435	126,833	58,751.00	199,065.00	52,524,021.61	22,876,630.26	84,400,631.04	101,423,007.10	43,218,668.33	171,853,572.00	153,947,028.80	67,292,114.22	252,740,937.50
12087	Monroe	Florida	73,090	3,912	1,311.50	6,253.50	2,000,042.94	538,802.22	3,780,890.17	3,706,081.46	517,624.53	11,338,453.60	5,706,124.39	1,194,799.10	13,829,007.92
12089	Nassau	Florida	73,314	4,555	2,422.50	6,770.00	2,098,690.50	921,790.23	3,849,953.47	3,751,270.35	937,973.87	11,888,835.31	5,849,960.85	2,015,540.89	14,121,764.24
12091	Okaloosa	Florida	180,822	14,487	8,070.00	20,807.50	6,131,413.09	3,008,875.05	9,671,771.23	11,417,442.98	4,014,664.03	26,119,188.55	17,548,856.07	7,489,394.48	33,046,636.73
12093	Okeechobee	Florida	39,996	2,250	1,050.00	3,378.00	990,421.71	318,004.70	2,124,942.47	1,705,911.29	305,718.06	6,161,646.79	2,696,333.01	684,628.37	7,440,837.38
12095	Orange	Florida	1,145,956	66,104	35,974.50	97,601.50	24,138,052.87	12,965,010.14	36,110,689.45	42,599,903.37	19,786,079.28	74,349,874.15	66,737,956.24	33,329,115.83	107,023,298.20
12097	Osceola	Florida	268,685	15,378	8,151.00	22,858.00	5,813,376.97	2,910,376.61	9,560,542.97	9,786,314.42	3,047,952.39	22,600,205.43	15,599,691.39	6,206,952.72	30,558,792.63
12099	Palm Beach	Florida	1,320,134	69,781	32,404.50	107,367.00	34,720,704.05	16,539,731.16	54,700,099.46	71,105,188.05	29,769,001.22	122,134,247.70	105,825,892.10	47,827,032.32	169,446,932.50
12101	Pasco	Florida	464,697	27,615	13,993.50	40,918.00	13,534,400.75	6,824,277.67	21,167,368.18	25,359,249.26	9,680,094.08	47,834,031.30	38,893,650.01	16,784,384.92	65,016,490.46
12103	Pinellas	Florida	916,542	54,019	28,594.50	80,292.00	28,060,677.38	14,887,198.78	42,904,730.32	55,755,731.12	24,498,166.07	97,243,092.44	83,816,408.50	41,004,236.37	136,804,944.60
12105	Polk	Florida	602,095	35,355	18,052.00	53,283.00	16,026,408.20	7,689,295.60	24,926,213.05	29,885,481.98	12,439,496.93	56,537,630.02	45,911,890.18	20,598,356.06	78,967,107.98
12107	Putnam	Florida	74,364	4,749	2,529.50	7,006.00	2,301,170.08	1,047,807.05	4,239,386.78	4,250,622.79	1,100,897.30	13,013,094.92	6,551,792.87	2,498,031.81	15,718,894.42
12109	St. Johns	Florida	190,039	11,537	6,373.00	16,633.00	5,170,245.91	2,419,390.95	8,366,548.58	9,419,013.94	3,239,833.67	20,732,526.13	14,589,259.85	6,247,617.80	27,842,767.87
12111	St. Lucie	Florida	277,789	15,312	6,921.50	23,703.50	7,313,319.80	3,267,615.89	12,143,255.97	14,244,305.21	4,468,001.18	30,443,214.79	21,557,625.02	8,338,796.92	39,527,336.54
12113	Santa Rosa	Florida	151,372	12,687	6,965.00	18,104.00	5,212,302.73	2,477,793.27	8,446,806.07	9,113,718.53	3,050,472.20	21,755,421.02	14,326,021.26	5,929,433.07	27,910,528.02
12115	Sarasota	Florida	379,448	22,173	10,182.50	34,717.00	13,720,043.17	6,177,737.89	22,076,898.43	29,069,173.49	11,559,342.68	54,135,999.69	42,789,216.66	18,444,697.24	74,935,663.82
12117	Seminole	Florida	422,718	24,170	12,185.50	37,007.00	9,708,206.88	4,895,731.99	15,538,477.22	17,729,759.37	6,763,081.96	39,853,639.59	27,437,966.24	11,928,444.32	52,868,187.43
12119	Sumter	Florida	93,420	5,562	3,029.00	8,078.00	4,214,148.57	1,949,531.53	6,999,798.71	9,012,011.44	3,037,752.67	20,119,979.18	13,226,160.01	5,349,719.00	25,303,330.74
12121	Suwannee	Florida	41,551	2,785	1,446.50	3,951.00	1,311,150.81	529,924.31	2,564,609.93	2,330,787.68	409,965.28	7,378,414.56	3,641,938.49	1,112,547.58	9,060,376.94
12123	Taylor	Florida	22,570	1,501	805.50	2,171.50	683,392.76	241,903.15	1,646,018.60	1,136,266.34	221,760.49	4,348,292.83	1,819,659.10	506,561.58	5,323,280.11
12125	Union	Florida	15,535	987	527.50	1,407.00	409,185.24	121,158.12	1,069,964.42	673,451.08	112,640.02	3,038,169.31	1,082,636.33	264,934.05	3,857,300.84
12127	Volusia	Florida	494,593	29,388	14,004.50	44,480.00	15,021,722.12	6,777,165.89	24,131,434.55	28,769,642.59	10,956,443.89	58,670,671.84	43,791,364.72	18,388,350.11	78,240,517.56
12129	Wakulla	Florida	30,776	2,143	1,273.00	3,038.00	880,055.14	316,572.57	1,961,017.76	1,507,577.24	297,929.69	5,959,292.77	2,387,632.38	662,483.30	6,878,250.46
12131	Walton	Florida	55,043	4,272	2,328.50	6,227.50	1,981,193.83	854,182.68	3,595,093.00	3,626,054.04	806,953.98	10,890,390.96	5,607,247.87	1,841,682.34	14,432,761.80
12133	Washington	Florida	24,896	1,921	1,091.00	2,729.50	829,370.23	308,751.67	1,645,700.80	1,372,802.21	279,421.70	4,911,376.21	2,202,172.44	687,562.20	5,889,029.51
13001	Appling	Georgia	18,236	1,223	615.00	1,804.00	503,451.37	149,200.48	1,229,486.39	931,334.40	156,228.75	4,560,487.57	1,434,785.77	343,327.06	5,217,526.04
13003	Atkinson	Georgia	8,375	584	319.50	863.00	221,468.39	51,099.59	731,492.62	283,897.91	49,773.79	1,697,575.34	505,366.30	109,591.97	2,144,994.95
13005	Bacon	Georgia	11,096	743	359.50	1,096.00	298,311.72	77,243.12	764,633.14	498,016.19	59,815.82	2,634,912.65	796,327.90	143,852.28	3,146,174.02
13007	Baker	Georgia	3,451	242	136.00	353.00	116,551.63	17,058.73	389,760.38	189,892.70	22,265.60	1,313,667.71	306,444.34	43,669.01	1,605,084.59
13009	Baldwin	Georgia	45,720	3,087	1,800.00	4,415.50	1,242,192.34	513,033.56	2,490,543.53	1,938,213.59	407,998.95	7,241,098.09	3,180,405.93	1,064,134.96	8,670,505.20
13011	Banks	Georgia	18,395	1,307	757.00	1,847.50	522,886.29	178,226.50	1,281,067.78	876,049.84	134,352.99	4,430,732.43	1,398,936.13	342,422.87	4,984,053.07
13013	Barrow	Georgia	69,367	4,990	3,056.00	6,990.50	1,757,842.48	803,790.53	3,133,368.49	2,681,551.95	690,467.09	8,562,886.83	4,439,394.43	1,601,755.90	10,942,936.04
13015	Bartow	Georgia	100,157	6,955	4,173.00	9,654.50	2,666,922.18	1,359,249.43	4,664,957.50	4,392,072.99	1,408,088.29	11,480,992.51	7,058,995.18	3,049,128.73	14,779,730.36
13017	Ben Hill	Georgia	17,634	1,219	654.00	1,817.50	515,768.96	163,914.36	1,252,510.67	837,575.70	126,120.30	3,299,332.69	1,353,344.66	316,546.94	4,416,974.99
13019	Berrien	Georgia	19,286	1,321	719.50	1,909.50	538,363.53	184,398.86	1,291,853.58	976,638.54	145,654.20	4,232,207.03	1,515,002.07	377,526.77	4,980,054.52
13021	Bibb	Georgia	155,547	10,750	6,107.00	15,416.50	4,304,548.22	2,135,950.32	7,010,610.95	7,539,712.28	2,704,927.14	18,394,605.07	11,844,260.50	5,310,822.51	23,850,862.92
13023	Bleckley	Georgia	13,063	874	474.00	1,262.50	370,601.66	113,922.94	948,813.34	576,263.32	80,448.07	3,001,562.73	946,864.98	213,808.00	3,600,745.60
13025	Brantley	Georgia	18,411	1,230	647.00	1,784.50	491,446.49	149,945.65	1,172,129.70	802,272.02	121,166.09	3,969,823.84	1,293,718.51	304,836.24	4,469,006.91

13027	Brooks	Georgia	16,243	1,118	615.00	1,639.00	519,777.08	161,157.83	1,206,664.25	857,184.01	120,002.46	3,642,922.14	1,376,961.09	304,831.15	4,427,312.19
13029	Bryan	Georgia	30,233	2,101	1,120.00	3,119.50	757,871.09	245,341.00	1,618,398.51	1,248,272.21	219,757.69	6,291,058.43	2,006,143.29	515,587.37	7,327,445.22
13031	Bulloch	Georgia	70,217	4,681	2,400.00	6,954.50	1,647,550.72	699,871.20	2,974,462.62	2,639,733.06	575,223.09	9,122,147.25	4,287,283.78	1,370,690.26	11,085,801.12
13033	Burke	Georgia	23,316	1,701	970.50	2,466.00	658,232.75	233,199.88	1,505,023.39	1,142,836.81	254,524.09	4,516,035.53	1,801,069.57	544,308.51	5,509,024.20
13035	Butts	Georgia	23,655	1,566	906.50	2,184.50	638,378.63	227,139.15	1,506,360.35	1,021,892.99	186,265.36	4,434,687.64	1,660,271.62	451,285.96	5,095,914.99
13037	Calhoun	Georgia	6,694	467	274.00	662.50	182,730.14	41,373.59	582,260.10	380,616.66	40,642.58	2,446,952.87	563,346.80	88,782.20	2,910,109.56
13039	Camden	Georgia	50,513	3,280	1,767.50	4,774.00	1,168,165.68	457,377.86	2,399,863.95	1,983,157.44	476,680.74	7,303,333.79	3,151,323.12	1,035,999.30	8,969,815.95
13043	Candler	Georgia	10,998	763	388.00	1,132.50	324,055.18	86,053.62	831,141.03	541,040.42	74,747.03	2,870,841.06	865,095.60	172,762.34	3,234,684.60
13045	Carroll	Georgia	110,527	7,854	4,529.50	11,266.50	2,967,757.16	1,442,373.55	5,105,354.03	4,983,905.05	1,413,502.14	13,573,981.80	7,951,662.21	3,200,252.42	17,379,442.02
13047	Catoosa	Georgia	63,942	4,504	2,677.50	6,388.50	1,864,925.22	880,614.16	3,357,265.65	3,224,723.13	768,345.24	9,950,553.49	5,089,648.35	1,793,279.63	12,522,356.58
13049	Charlton	Georgia	12,171	772	419.50	1,123.00	312,598.29	86,814.59	888,647.46	527,824.87	78,533.06	3,055,226.60	840,423.16	174,822.80	3,493,606.96
13051	Chatham	Georgia	265,128	17,817	9,027.00	26,241.00	7,063,973.84	3,320,497.76	11,267,510.12	12,904,396.20	4,711,214.64	29,233,058.28	19,968,370.04	8,491,369.98	38,019,022.28
13053	Chattahoochee	Georgia	11,267	816	458.00	1,182.00	219,654.14	60,353.21	598,777.32	326,474.61	101,691.94	1,587,036.81	546,128.75	176,571.10	1,888,884.80
13055	Chattooga	Georgia	26,015	1,826	1,041.00	2,549.50	788,035.39	303,579.56	1,658,491.33	1,295,377.43	243,032.62	4,935,778.11	2,083,412.82	641,199.60	5,838,946.06
13057	Cherokee	Georgia	214,346	14,973	9,395.00	20,965.00	5,403,534.31	3,003,798.62	8,596,499.83	8,812,138.70	3,283,171.20	21,532,027.79	14,215,673.00	6,899,844.69	27,563,064.10
13059	Clarke	Georgia	116,714	7,856	4,601.50	11,335.00	2,664,735.34	1,308,950.09	4,664,936.99	4,651,478.99	1,321,539.62	11,912,940.72	7,316,214.33	2,851,479.00	15,223,305.63
13061	Clay	Georgia	3,183	235	132.00	336.00	122,038.48	19,076.24	406,409.71	216,983.42	24,148.65	1,585,727.59	339,021.89	46,806.51	1,840,006.29
13063	Clayton	Georgia	259,424	18,053	10,790.00	25,064.00	5,854,609.89	3,111,612.24	9,050,756.57	9,391,957.55	3,576,495.29	22,368,291.03	15,246,567.45	7,324,366.66	29,214,004.77
13065	Clinch	Georgia	6,798	462	240.00	687.00	175,107.28	40,351.18	504,284.09	312,823.41	42,310.20	2,079,612.54	487,930.70	90,755.65	2,313,212.65
13067	Cobb	Georgia	688,078	48,017	30,259.50	66,826.50	17,176,005.88	10,345,610.60	25,227,478.66	31,746,398.98	15,582,006.99	59,415,821.22	48,922,404.86	26,740,602.24	78,886,599.42
13069	Coffee	Georgia	42,356	2,864	1,557.00	4,161.50	1,104,628.82	432,679.48	2,239,584.76	1,797,010.10	352,942.31	6,822,997.07	2,901,638.92	882,186.76	8,171,423.27
13071	Colquitt	Georgia	45,498	3,213	1,682.50	4,777.00	1,320,719.95	476,479.91	2,595,684.17	2,144,349.97	383,555.96	7,447,236.93	3,465,069.93	981,240.02	9,523,137.26
13073	Columbia	Georgia	124,053	8,958	5,130.00	12,827.00	3,384,618.29	1,681,821.45	5,670,753.10	5,678,587.15	1,705,666.11	15,448,233.65	9,063,205.44	3,671,591.93	18,808,974.15
13075	Cook	Georgia	17,212	1,179	618.00	1,749.00	474,097.77	144,531.96	1,099,157.29	821,106.39	117,390.14	3,968,169.10	1,295,204.16	289,405.96	4,603,877.86
13077	Coweta	Georgia	127,317	8,973	5,110.50	12,640.00	3,374,949.95	1,582,003.66	5,978,245.91	5,422,696.62	1,730,767.61	14,094,942.34	8,797,646.57	3,750,800.60	18,685,318.61
13079	Crawford	Georgia	12,630	841	474.00	1,200.50	351,678.22	111,194.93	855,110.76	545,719.89	83,698.04	2,730,512.09	897,398.11	216,026.71	3,644,680.82
13081	Crisp	Georgia	23,439	1,661	966.00	2,385.50	690,407.21	245,320.44	1,658,803.08	1,197,819.75	208,116.83	5,375,015.78	1,888,226.96	510,569.98	6,234,753.96
13083	Dade	Georgia	16,633	1,152	641.50	1,646.50	496,434.68	159,200.14	1,161,466.85	749,135.68	132,390.65	3,252,096.65	1,245,570.35	331,969.40	4,034,935.29
13085	Dawson	Georgia	22,330	1,557	973.50	2,178.50	651,249.61	242,338.23	1,412,181.19	1,037,684.35	184,239.48	3,933,680.66	1,688,933.95	481,311.75	4,653,067.73
13087	Decatur	Georgia	27,842	2,018	1,135.50	2,885.00	829,533.67	315,110.50	1,679,394.25	1,400,972.51	259,023.88	5,947,758.85	2,230,506.18	641,549.99	6,985,834.28
13089	De Kalb	Georgia	691,893	47,436	29,455.00	65,009.50	17,225,872.57	10,205,642.30	25,089,430.17	31,437,654.09	15,913,085.95	58,685,293.07	48,663,526.66	27,117,404.07	79,004,057.64
13091	Dodge	Georgia	21,796	1,473	792.50	2,144.50	603,014.69	201,714.29	1,504,890.06	1,021,736.59	155,796.55	4,505,132.17	1,624,751.28	365,952.13	5,251,149.38
13093	Dooly	Georgia	14,918	1,003	560.00	1,473.50	446,406.02	139,689.12	1,164,273.34	710,191.67	107,034.05	3,254,408.72	1,156,597.69	262,020.28	3,909,928.08
13095	Dougherty	Georgia	94,565	6,733	3,728.50	9,848.50	2,639,967.56	1,252,889.84	4,519,722.09	4,738,528.22	1,292,246.35	13,000,171.97	7,378,495.78	2,795,050.99	16,398,350.13
13097	Douglas	Georgia	132,403	9,377	5,761.00	12,893.00	3,251,918.33	1,674,377.14	5,477,931.83	4,901,356.00	1,543,940.28	12,794,881.41	8,153,274.33	3,620,963.58	16,676,555.60
13099	Early	Georgia	11,008	825	491.50	1,184.50	366,028.40	103,296.42	1,027,544.65	635,089.30	120,317.47	2,937,745.98	1,001,117.70	265,463.36	3,438,248.70
13101	Echols	Georgia	4,034	283	152.00	427.50	99,331.09	16,344.79	391,564.06	142,456.35	20,579.69	1,118,220.40	241,787.44	40,434.06	1,319,648.14
13103	Effingham	Georgia	52,250	3,630	1,882.50	5,459.00	1,285,906.32	524,517.64	2,720,523.58	2,114,069.24	442,597.08	8,179,745.63	3,399,975.56	1,072,631.01	9,522,918.59
13105	Elbert	Georgia	20,166	1,462	852.00	2,071.00	646,183.63	228,159.34	1,457,897.90	1,158,883.93	205,901.84	5,027,522.47	1,805,067.56	507,245.68	5,921,522.80
13107	Emanuel	Georgia	22,598	1,593	891.00	2,348.50	678,010.74	227,291.50	1,591,362.97	1,063,005.45	181,725.42	4,872,809.86	1,741,016.19	464,164.26	5,441,905.45
13109	Evans	Georgia	11,000	761	412.50	1,135.00	304,850.70	78,665.79	795,265.95	472,578.81	73,312.54	2,467,315.80	777,429.51	162,449.68	3,022,417.73
13111	Fannin	Georgia	23,682	1,637	971.50	2,278.50	861,738.16	340,670.44	1,751,629.43	1,502,513.14	282,706.81	5,404,295.14	2,364,251.29	734,203.02	6,500,201.30
13113	Fayette	Georgia	106,567	7,197	4,296.00	9,850.50	2,990,617.03	1,452,599.82	4,977,346.08	5,439,542.26	1,685,321.24	16,269,229.69	8,430,159.28	3,499,225.05	19,709,536.94
13115	Floyd	Georgia	96,317	6,846	3,816.50	9,685.00	2,849,551.09	1,419,801.74	4,852,317.32	5,189,309.64	1,534,373.39	14,546,093.80	8,038,860.73	3,347,878.98	18,563,639.55
13117	Forsyth	Georgia	175,511	12,432	7,848.00	17,491.00	4,308,355.32	2,440,833.58	7,126,964.03	7,638,347.75	2,935,976.95	17,555,110.84	11,946,703.08	5,604,479.05	22,390,686.70
13119	Franklin	Georgia	22,084	1,573	884.00	2,212.50	711,353.55	248,593.16	1,785,571.45	1,237,572.52	216,677.72	4,690,337.36	1,948,926.07	546,801.90	5,778,852.11
13121	Fulton	Georgia	920,581	61,815	38,722.50	87,280.00	22,409,118.98	13,667,047.42	32,359,197.64	44,212,174.30	23,982,116.42	72,393,134.47	66,621,293.28	38,925,064.25	101,102,624.50
13123	Gilmer	Georgia	28,292	1,983	1,196.50	2,829.50	964,683.63	366,608.54	2,063,835.04	1,728,270.55	308,076.46	6,791,265.90	2,692,954.18	748,589.01	8,158,862.67
13125	Glascock	Georgia	3,082	220	115.00	326.00	105,103.67	12,325.92	400,674.49	144,395.74	14,735.54	1,008,142.48	249,499.41	30,683.67	1,286,346.45
13127	Glynn	Georgia	79,626	5,267	2,765.50	7,842.00	2,278,155.03	992,284.97	4,250,015.61	4,012,663.14	1,090,789.30	11,924,853.28	6,290,818.17	2,343,093.39	14,831,409.62
13129	Gordon	Georgia	55,186	3,946	2,405.50	5,467.50	1,540,898.22	691,260.55	2,893,396.44	2,480,861.49	652,993.53	8,305,779.71	4,021,759.71	1,548,302.06	10,178,384.90
13131	Grady	Georgia	25,011	1,805	1,029.00	2,582.00	742,039.28	271,470.07	1,612,283.25	1,290,638.25	215,144.34	5,455,844.50	2,032,677.53	547,479.12	6,288,491.18
13133	Greene	Georgia	15,994	1,129	653.50	1,611.50	601,722.07	187,785.25	1,439,690.88	1,071,916.72	178,227.50	4,484,978.54	1,673,638.80	424,860.41	5,442,586.36
13135	Gwinnett	Georgia	805,321	57,728	34,207.50	79,074.50	18,891,797.85	11,169,456.54	27,181,729.27	32,486,658.75	16,660,150.74	60,631,221.76	51,378,456.61	28,479,489.28	82,861,415.44
13137	Habersham	Georgia	43,041	3,148	1,831.50	4,595.00	1,363,993.77	568,829.61	2,540,644.62	2,402,821.57	495,016.10	8,621,409.25	3,766,815.34	1,221,624.85	10,177,445.74
13139	Hall	Georgia	179,684	13,086	7,664.50	18,293.00	4,876,944.41	2,588,833.53	7,803,760.82	8,475,928.51	3,089,343.27	20,897,188.26	13,352,872.91	6,036,324.61	27,424,955.29
13141	Hancock	Georgia	9,429	632	340.50	919.00	296,266.89	77,624.39	876,537.42	490,017.06	60,086.66	2,511,626.17	786,283.95	149,563.62	3,022,990.55
13143	Haralson	Georgia	28,780	2,056	1,170.50	2,908.00	852,033.12	315,461.28	1,754,043.12	1,466,506.69	277,803.22	5,043,501.09	2,318,540.07	696,053.51	6,201,463.29
13145	Harris	Georgia	32,024	2,264	1,314.00	3,204.00	977,502.22	397,242.31	1,967,935.01	1,528,761.99	284,774.64	6,031,737.48	2,506,264.21	767,765.56	7,476,660.98
13147	Hart	Georgia	25,213	1,829	1,069.00	2,569.50	859,710.02	337,656.17	1,793,113.35	1,505,882.20	267,541.89	6,392,097.61	2,365,592.22	655,297.79	7,320,605.64
13149	Heard	Georgia	11,834	839	490.50	1,197.00	344,972.48	92,914.78	875,194.26	627,408.78	142,931.18	2,766,611.16	972,381.25	258,913.82	3,292,185.55
13151	Henry	Georgia	203,922	13,833	8,034.00	19,155.00	4,752,322.90	2,448,540.90	7,685,630.96	7,557,097.67	2,590,292.45	19,146,140.27	12,309,420.57	5,558,871.77	24,721,016.76
13153	Houston	Georgia	139,900	9,687	5,231.00	14,061.50	3,564,071.28	1,716,084.71	5,906,011.10	6,338,680.68	2,140,665.75	16,749,868.49	9,902,751.96	4,296,073.45	20,904,427.22
13155	Irwin	Georgia	9,538	649	380.50	941.50	269,935.23	77,454.30	756,296.31	445,843.85	67,575.31	2,430,223.22	715,779.08	153,690.94	2,949,425.57
13157	Jackson	Georgia	60,485	4,343	2,657.50	6,084.50	1,674,566.96	775,970.73	3,104,951.78	2,943,692.11	714,674.42	9,757,341.48	4,618,259.07	1,614,287.72	12,413,569.89
13159	Jasper	Georgia	13,900	950	560.50	1,358.50	404,021.45	127,200.30	1,099,214.32	592,257.90	101,891.13	3,224,343.55	996,279.35	259,713.73	3,780,872.59

13161	Jeff Davis	Georgia	15,068	1,016	534.50	1,512.00	395,288.63	119,056.73	933,156.43	619,061.04	97,050.09	3,368,857.48	1,014,349.67	228,783.55	3,872,304.37
13163	Jefferson	Georgia	16,930	1,193	657.00	1,765.00	501,090.68	151,687.26	1,224,902.00	877,968.28	143,795.42	4,353,880.09	1,379,058.96	326,262.77	4,928,478.38
13165	Jenkins	Georgia	8,340	596	352.00	849.00	250,616.23	66,155.14	661,809.51	487,535.70	57,003.05	2,281,286.76	738,151.92	133,517.76	2,642,246.48
13167	Johnson	Georgia	9,980	671	309.50	994.50	281,092.66	71,591.94	745,380.72	497,560.95	57,152.39	2,772,292.67	778,653.61	148,756.75	3,260,155.41
13169	Jones	Georgia	28,669	1,991	1,184.50	2,896.00	806,613.71	311,491.03	1,723,832.15	1,314,135.08	244,144.61	5,178,071.45	2,120,748.80	610,653.04	6,551,058.64
13171	Lamar	Georgia	18,317	1,227	696.50	1,752.00	510,791.66	174,237.73	1,226,411.24	884,886.06	139,891.91	3,641,513.76	1,395,677.72	351,613.36	4,513,220.57
13173	Lanier	Georgia	10,078	700	365.00	1,019.50	261,283.23	60,397.64	724,614.00	446,364.01	54,928.31	2,925,137.90	707,647.24	128,626.02	3,285,228.05
13175	Laurens	Georgia	48,434	3,296	1,800.50	4,824.50	1,397,312.56	532,944.18	2,841,250.90	2,572,573.38	512,270.48	8,896,030.68	3,969,885.94	1,150,933.08	10,521,712.55
13177	Lee	Georgia	28,298	1,962	1,131.50	2,824.50	707,450.65	260,790.50	1,569,046.50	1,054,444.26	205,113.06	4,724,704.60	1,761,894.91	536,860.55	5,917,459.96
13179	Liberty	Georgia	63,453	4,482	2,383.50	6,627.50	1,406,803.70	553,423.36	2,697,961.22	2,268,196.74	626,446.93	6,944,455.66	3,675,000.44	1,372,194.43	8,630,420.66
13181	Lincoln	Georgia	7,996	566	331.50	813.00	272,132.07	64,301.24	727,525.02	493,052.48	61,597.84	2,523,369.46	765,184.55	144,862.27	3,206,405.77
13183	Long	Georgia	14,464	1,003	525.50	1,484.00	321,917.09	87,495.09	877,173.66	452,117.02	74,848.08	2,854,933.83	774,034.12	177,132.05	3,146,574.03
13185	Lowndes	Georgia	109,233	7,542	4,135.00	10,896.50	2,713,612.78	1,211,435.15	4,768,394.40	4,140,363.76	1,160,746.73	11,710,660.41	6,853,976.54	2,692,963.75	15,429,521.39
13187	Lumpkin	Georgia	29,966	2,070	1,222.50	2,984.00	847,191.27	354,906.14	1,804,740.01	1,387,358.09	284,556.09	4,918,897.46	2,234,549.35	694,901.29	6,311,549.24
13189	McDuffie	Georgia	21,875	1,578	868.00	2,307.00	651,635.59	207,142.39	1,505,186.66	1,150,636.71	178,019.71	4,381,504.26	1,802,272.30	423,821.06	5,185,853.09
13191	McIntosh	Georgia	14,333	941	504.00	1,401.50	445,297.38	131,462.58	1,069,762.17	864,102.06	98,222.14	3,957,475.03	1,309,399.44	248,693.10	4,395,971.80
13193	Macon	Georgia	14,740	991	585.50	1,420.00	419,757.73	131,374.05	1,060,853.54	763,466.29	113,171.18	3,729,200.20	1,183,224.02	268,510.28	4,214,622.11
13195	Madison	Georgia	28,120	2,014	1,186.50	2,817.50	832,736.24	328,367.94	1,724,380.64	1,354,667.10	234,603.44	5,214,549.58	2,187,403.34	634,332.72	6,353,543.36
13197	Marion	Georgia	8,742	606	322.00	855.00	264,139.97	63,886.92	790,653.64	379,547.42	54,077.50	2,322,884.95	643,687.38	127,355.16	2,820,508.73
13199	Meriwether	Georgia	21,992	1,536	891.50	2,232.00	713,112.71	259,550.72	1,614,378.03	1,238,869.93	209,517.41	5,990,899.04	1,951,982.65	524,991.09	6,709,733.49
13201	Miller	Georgia	6,125	450	259.00	645.00	207,571.45	47,163.39	629,280.88	384,030.17	48,622.08	1,786,831.12	591,601.62	100,908.09	2,328,315.53
13205	Mitchell	Georgia	23,498	1,668	877.00	2,404.50	678,375.30	226,956.19	1,647,637.29	1,047,926.99	158,604.73	4,983,913.74	1,726,302.28	458,933.58	5,915,098.65
13207	Monroe	Georgia	26,424	1,776	1,049.00	2,502.50	805,155.04	289,660.21	1,857,338.25	1,380,142.02	253,852.69	5,608,094.21	2,185,297.06	661,655.59	7,025,708.88
13209	Montgomery	Georgia	9,123	604	331.00	886.50	238,676.63	56,168.95	701,908.98	458,677.70	53,333.63	2,298,893.40	697,354.33	121,672.98	2,672,622.14
13211	Morgan	Georgia	17,868	1,246	691.50	1,801.50	536,881.37	183,708.36	1,270,298.37	955,324.31	149,041.66	4,391,292.93	1,492,205.67	371,945.03	5,046,956.62
13213	Murray	Georgia	39,628	2,797	1,605.00	3,947.00	1,051,011.10	451,192.39	2,112,535.96	1,730,406.42	368,616.01	6,809,714.44	2,781,417.52	931,578.98	7,915,188.52
13215	Muscogee	Georgia	189,885	13,750	7,747.00	19,610.50	5,283,336.44	2,696,528.38	8,376,222.42	9,405,188.18	3,154,866.37	22,043,045.71	14,688,524.62	6,333,705.70	28,516,357.62
13217	Newton	Georgia	99,958	6,939	3,988.00	9,910.50	2,478,947.63	1,208,092.18	4,450,736.98	4,204,814.51	1,253,150.23	12,428,476.22	6,683,762.14	2,693,827.76	15,183,499.89
13219	Oconee	Georgia	32,808	2,325	1,390.50	3,280.00	908,665.47	366,429.10	1,976,277.86	1,462,960.34	332,475.61	6,009,232.35	2,371,625.81	768,198.38	6,818,859.87
13221	Oglethorpe	Georgia	14,899	1,048	583.00	1,501.50	446,240.19	134,752.06	1,119,922.52	710,431.46	102,527.03	3,444,565.43	1,156,671.65	250,953.89	4,044,651.55
13223	Paulding	Georgia	142,324	10,083	5,881.00	13,990.50	3,254,070.37	1,663,178.91	5,474,542.08	4,770,127.38	1,411,063.23	13,612,517.70	8,024,197.75	3,333,904.09	17,237,464.05
13225	Peach	Georgia	27,695	1,858	1,028.00	2,646.00	732,004.19	253,257.09	1,593,526.12	1,243,963.79	230,708.77	5,436,624.54	1,975,967.98	552,195.91	6,354,908.90
13227	Pickens	Georgia	29,431	2,037	1,206.00	2,851.50	942,837.76	381,813.47	1,922,958.59	1,729,954.06	317,024.07	7,290,448.42	2,672,791.82	753,714.39	8,218,119.45
13229	Pierce	Georgia	18,758	1,263	642.50	1,871.50	516,475.74	148,433.86	1,296,475.64	843,709.61	130,985.03	3,632,613.70	1,360,185.35	298,551.93	4,254,288.74
13231	Pike	Georgia	17,869	1,230	720.00	1,730.00	475,295.77	155,811.89	1,133,920.58	759,267.83	124,553.20	3,797,609.34	1,234,563.60	305,956.23	4,339,234.60
13233	Polk	Georgia	41,475	3,051	1,797.00	4,369.50	1,216,313.89	542,397.72	2,339,423.25	2,200,960.14	478,128.08	6,832,037.88	3,417,274.03	1,175,664.51	8,156,747.84
13235	Pulaski	Georgia	12,010	796	432.00	1,145.50	350,672.19	94,884.99	985,622.95	635,387.58	98,691.14	3,271,922.12	986,059.78	213,640.29	3,891,105.28
13237	Putnam	Georgia	21,218	1,476	878.50	2,129.50	690,785.66	261,325.34	1,621,810.66	1,200,167.48	204,454.76	4,638,676.77	1,890,953.14	548,985.68	5,709,750.66
13239	Quitman	Georgia	2,513	185	106.00	267.00	103,632.93	15,134.21	359,190.08	167,315.13	19,404.90	1,319,260.89	270,948.06	37,330.84	1,643,386.29
13241	Rabun	Georgia	16,276	1,200	711.50	1,714.50	623,723.05	187,146.26	1,521,919.10	1,081,322.34	166,760.65	5,100,655.74	1,705,045.38	402,970.57	5,767,188.26
13243	Randolph	Georgia	7,719	557	309.00	791.50	273,076.79	67,128.06	778,169.62	487,911.68	56,204.26	2,848,313.90	760,988.47	134,452.96	3,169,349.13
13245	Richmond	Georgia	200,549	14,441	8,298.50	20,822.00	5,646,049.22	2,938,431.71	9,045,410.03	9,842,353.18	3,708,444.37	22,098,500.59	15,488,402.40	7,134,258.64	29,161,383.73
13247	Rockdale	Georgia	85,215	5,894	3,607.00	8,106.00	2,230,582.60	1,144,708.90	3,968,387.27	3,969,818.64	1,151,275.09	12,685,610.32	6,200,401.25	2,440,387.21	15,154,913.84
13249	Schley	Georgia	5,010	356	208.50	523.50	136,931.74	30,387.01	447,875.03	203,966.12	38,600.52	1,170,248.19	340,897.86	74,945.73	1,441,945.08
13251	Screven	Georgia	14,593	1,043	584.00	1,517.00	441,063.60	135,724.06	1,081,434.66	742,304.92	112,067.05	3,715,790.48	1,183,368.51	266,332.49	4,201,714.92
13253	Seminole	Georgia	8,729	633	366.50	897.50	303,142.64	79,335.99	845,039.81	464,257.15	75,661.93	2,352,870.93	767,399.79	171,232.44	2,843,263.79
13255	Spalding	Georgia	64,073	4,462	2,616.00	6,383.00	1,810,028.79	830,880.25	3,482,436.55	3,124,636.58	751,136.76	9,974,513.90	4,934,665.36	1,857,889.75	12,099,455.67
13257	Stephens	Georgia	26,175	1,888	1,169.50	2,628.00	873,818.23	365,790.42	1,769,354.90	1,519,396.31	305,110.46	6,198,526.62	2,393,214.54	733,529.12	7,155,413.94
13259	Stewart	Georgia	6,058	411	228.50	593.00	177,149.68	39,678.02	589,324.58	268,170.49	40,238.35	1,339,481.58	445,320.16	85,288.29	1,800,907.31
13261	Sumter	Georgia	32,819	2,336	1,230.00	3,386.50	921,463.31	355,818.93	1,924,399.06	1,578,810.05	291,071.03	6,757,816.91	2,500,273.36	766,668.88	7,831,654.36
13263	Talbot	Georgia	6,865	467	266.00	674.00	217,931.12	53,150.16	639,398.75	370,298.78	52,207.48	2,381,138.96	588,229.90	112,020.60	2,713,302.43
13265	Taliaferro	Georgia	1,717	119	65.00	175.00	62,928.05	6,915.33	281,574.32	133,854.89	9,810.22	855,695.49	196,782.94	18,326.48	1,029,864.26
13267	Tattnall	Georgia	25,520	1,676	908.00	2,450.00	666,148.11	236,273.25	1,498,750.97	1,143,538.08	161,691.97	5,171,985.44	1,809,686.19	461,366.01	6,001,254.62
13269	Taylor	Georgia	8,906	614	342.00	883.00	271,438.44	68,566.03	751,165.47	472,545.47	69,557.30	2,481,724.58	743,983.92	151,751.11	2,949,675.53
13271	Telfair	Georgia	16,500	1,079	569.00	1,580.00	476,093.63	143,698.23	1,195,492.36	746,297.86	99,875.41	3,657,509.11	1,222,391.49	274,016.53	4,370,070.52
13273	Terrell	Georgia	9,315	672	368.50	980.50	295,475.73	75,014.31	932,615.94	484,381.23	69,059.67	2,606,529.22	779,856.96	160,812.84	3,044,251.56
13275	Thomas	Georgia	44,720	3,178	1,805.50	4,545.00	1,369,906.83	567,816.01	2,562,591.55	2,489,242.62	551,833.64	8,292,905.95	3,859,149.46	1,264,821.89	9,744,027.04
13277	Tift	Georgia	40,118	2,750	1,445.50	4,041.00	1,070,476.70	391,598.23	2,243,872.58	1,793,603.84	352,068.94	6,275,161.28	2,864,080.54	835,797.15	7,176,066.91
13279	Toombs	Georgia	27,223	1,907	990.50	2,792.50	779,071.54	286,271.65	1,734,204.74	1,305,611.43	229,905.66	5,271,836.16	2,084,682.97	581,432.19	6,224,674.77
13281	Towns	Georgia	10,471	738	440.00	1,023.00	444,090.93	137,307.06	997,948.29	891,812.01	121,094.61	3,776,342.88	1,335,902.94	284,988.10	4,467,306.63
13283	Treutlen	Georgia	6,885	473	236.00	702.00	197,826.53	43,161.77	584,591.75	290,001.18	36,172.19	1,764,362.79	487,827.71	88,784.68	2,062,343.86
13285	Troup	Georgia	67,044	4,817	2,695.00	6,956.00	1,909,389.38	903,162.55	3,583,582.50	3,263,023.11	898,157.92	10,103,253.29	5,172,412.49	1,917,568.21	12,725,193.79
13287	Turner	Georgia	8,930	610	327.00	894.00	266,533.12	66,284.91	808,192.19	451,271.84	56,109.42	2,492,011.22	717,804.97	135,428.07	3,149,377.72
13289	Twiggs	Georgia	9,023	597	338.00	849.50	279,035.34	70,225.58	797,721.61	445,529.29	74,782.12	2,561,060.70	724,564.62	157,632.30	2,946,464.38
13291	Union	Georgia	21,356	1,523	905.00	2,140.50	868,879.00	364,791.09	1,752,958.30	1,558,339.40	307,041.69	6,327,380.91	2,427,218.40	781,825.92	7,506,286.79
13293	Upson	Georgia	27,153	1,859	1,045.00	2,649.50	814,372.35	308,995.63	1,664,174.51	1,397,239.31	246,596.07	6,140,513.06	2,211,611.66	623,669.37	7,048,159.06

13295	Walker	Georgia	68,756	4,792	2,846.00	6,916.50	2,066,282.55	889,718.70	3,799,001.11	3,621,491.60	839,904.26	10,834,960.73	5,687,774.15	1,991,974.76	13,192,050.01
13297	Walton	Georgia	83,768	5,861	3,614.00	8,284.50	2,307,257.54	1,096,955.01	4,143,517.19	3,896,312.05	1,093,126.18	11,672,202.13	6,203,569.60	2,417,237.79	14,070,445.51
13299	Ware	Georgia	36,312	2,406	1,267.50	3,511.50	1,045,752.54	386,602.53	2,279,139.18	1,935,559.26	362,873.45	8,037,665.63	2,981,311.80	844,294.64	9,460,754.89
13301	Warren	Georgia	5,834	417	233.50	614.50	200,538.67	40,714.68	652,455.42	318,051.64	41,883.77	1,718,144.87	518,590.31	93,262.12	2,158,991.73
13303	Washington	Georgia	21,187	1,470	791.50	2,171.50	613,826.02	199,191.70	1,361,843.46	1,087,146.72	175,618.73	4,758,493.48	1,700,972.74	442,613.98	5,487,091.12
13305	Wayne	Georgia	30,099	2,003	1,061.50	2,907.50	812,639.95	311,656.87	1,782,452.75	1,466,532.23	272,953.00	6,041,982.97	2,279,172.18	623,974.68	7,225,220.43
13307	Webster	Georgia	2,799	195	106.50	285.50	82,806.87	12,946.00	326,783.27	147,066.58	16,663.48	913,557.22	229,873.46	33,216.84	1,138,308.08
13309	Wheeler	Georgia	7,421	480	259.00	699.00	203,820.36	43,063.15	669,981.60	297,839.69	42,681.65	1,830,391.55	501,660.05	91,737.77	2,293,725.65
13311	White	Georgia	27,144	1,965	1,136.50	2,769.50	896,611.51	365,669.69	1,709,178.43	1,545,974.18	282,649.68	5,754,933.54	2,442,585.68	706,306.43	6,933,079.08
13313	Whitfield	Georgia	102,599	7,404	4,536.50	10,343.50	2,714,434.01	1,344,172.70	4,607,738.60	4,612,631.53	1,394,147.83	13,623,752.35	7,327,065.55	3,164,439.58	17,353,903.45
13315	Wilcox	Georgia	9,255	606	328.50	879.50	260,596.99	63,609.24	712,674.31	376,942.46	53,999.96	2,142,130.85	637,539.45	131,820.56	2,392,065.02
13317	Wilkes	Georgia	10,593	766	439.50	1,097.00	355,929.91	105,887.66	941,539.37	607,453.33	85,057.36	2,731,299.29	963,383.23	208,824.53	3,151,620.30
13319	Wilkinson	Georgia	9,563	656	359.50	951.00	278,451.52	77,755.53	760,333.44	590,797.24	95,314.92	2,963,977.46	869,248.76	186,463.66	3,306,752.53
13321	Worth	Georgia	21,679	1,527	843.00	2,200.50	680,540.01	231,526.31	1,618,710.42	1,005,328.55	172,153.33	4,018,387.90	1,685,868.56	458,848.03	5,046,207.59
15001	Hawaii	Hawaii	185,079	16,837	8,341.50	25,821.00	7,521,470.73	3,420,841.92	12,102,771.83	13,178,224.65	4,508,563.42	28,464,866.52	20,699,695.38	8,709,416.18	38,950,889.50
15003	Honolulu	Hawaii	953,207	87,212	38,922.00	137,895.00	36,952,264.38	15,661,490.33	60,092,430.43	70,652,302.37	28,111,988.55	126,146,295.70	107,604,566.80	44,695,980.23	178,577,539.20
15007	Kauai	Hawaii	67,091	6,085	3,215.00	8,893.00	2,753,543.78	1,250,979.08	4,746,510.68	4,866,082.07	1,347,172.09	13,720,150.25	7,619,625.85	2,791,633.60	17,458,862.07
15009	Maui	Hawaii	154,834	13,976	8,219.50	19,540.50	5,888,292.20	3,067,421.48	9,323,086.40	10,497,761.47	3,862,477.48	24,704,095.71	16,386,053.67	7,538,792.40	32,934,578.69
16001	Ada	Idaho	392,365	28,050	16,978.00	39,819.50	10,455,830.99	5,821,775.62	15,832,966.12	18,161,408.12	8,042,379.73	36,347,976.64	28,617,239.11	15,200,227.05	48,402,836.61
16003	Adams	Idaho	3,976	276	150.50	408.00	151,915.51	26,685.12	578,617.55	281,481.90	29,282.15	1,580,322.25	433,397.40	60,410.36	1,718,489.92
16005	Bannock	Idaho	82,839	4,287	1,193.50	7,373.00	1,583,200.77	359,350.46	3,244,417.51	2,550,131.58	270,616.52	8,363,548.71	4,133,332.34	717,797.54	10,539,617.02
16007	Bear Lake	Idaho	5,986	295	71.50	524.00	143,709.48	9,202.06	515,765.89	220,189.37	11,718.57	1,495,980.84	363,898.86	20,906.47	1,833,493.00
16009	Benewah	Idaho	9,285	666	337.00	968.00	324,665.36	86,575.14	894,050.09	543,782.42	78,449.73	2,658,176.83	868,447.78	177,121.30	3,378,110.64
16011	Bingham	Idaho	45,607	2,567	725.00	4,280.00	921,161.26	235,305.51	1,995,466.06	1,530,715.71	173,325.96	5,885,912.14	2,451,876.96	460,041.34	7,028,069.49
16013	Blaine	Idaho	21,376	1,246	488.00	1,989.50	521,124.69	126,032.67	1,226,968.27	799,147.24	111,773.62	3,549,146.35	1,320,271.93	251,688.98	4,239,946.32
16015	Boise	Idaho	7,028	458	235.00	677.50	222,959.83	45,621.10	717,069.38	346,736.10	36,879.75	2,073,723.60	569,695.93	91,403.98	2,236,739.96
16017	Bonner	Idaho	40,877	2,785	1,232.50	4,459.00	1,388,014.35	433,559.96	2,817,859.08	2,395,908.85	399,580.42	8,143,966.78	3,783,923.20	1,037,244.61	9,953,548.16
16019	Bonneville	Idaho	104,234	5,405	1,104.50	9,519.00	1,966,577.31	351,568.98	4,005,014.86	3,254,436.56	292,848.70	11,036,222.15	5,221,013.87	742,873.67	13,792,059.14
16021	Boundary	Idaho	10,972	763	324.00	1,247.00	347,788.95	76,558.67	945,734.91	632,207.28	72,092.27	3,150,209.83	979,996.23	162,487.91	3,629,996.89
16023	Butte	Idaho	2,891	169	48.00	283.00	78,898.27	6,797.13	307,627.31	175,157.55	24,764.27	1,048,643.74	254,055.82	33,264.74	1,245,080.17
16025	Camas	Idaho	1,117	68	28.50	108.00	30,543.94	1,988.50	182,832.21	45,696.58	4,366.44	422,607.54	76,240.52	7,301.11	543,810.22
16027	Canyon	Idaho	188,923	14,481	8,564.50	20,456.50	5,132,780.56	2,762,696.20	8,149,635.67	8,536,971.98	2,894,207.38	21,953,188.49	13,669,752.53	5,882,187.25	28,986,649.00
16029	Caribou	Idaho	6,963	347	75.00	615.00	150,201.16	11,742.89	544,878.08	287,000.05	21,049.32	1,740,999.01	437,201.21	38,786.97	2,078,807.56
16031	Cassia	Idaho	22,952	1,349	545.50	2,147.50	497,181.43	140,149.57	1,224,663.99	876,144.30	124,261.28	4,189,919.98	1,373,325.74	286,642.88	4,896,159.69
16033	Clark	Idaho	982	55	12.00	99.50	23,640.77	624.34	140,475.92	31,099.63	2,074.11	245,327.16	54,740.40	2,804.67	432,585.66
16035	Clearwater	Idaho	8,761	572	280.00	907.50	333,506.07	72,264.48	944,182.48	656,432.52	67,610.52	3,351,965.72	989,938.59	161,329.02	4,001,896.06
16037	Custer	Idaho	4,368	256	95.50	419.00	129,379.30	15,701.26	502,447.07	223,787.40	20,247.03	1,376,562.22	353,166.70	41,269.59	1,638,016.22
16039	Elmore	Idaho	27,038	1,883	968.00	2,744.00	659,272.26	232,577.41	1,496,577.27	1,090,082.74	231,386.17	4,841,182.37	1,749,355.00	505,467.05	5,705,114.15
16041	Franklin	Idaho	12,786	658	165.50	1,178.00	236,563.14	34,317.95	761,859.84	311,053.18	29,255.34	1,570,122.53	547,616.32	74,834.25	2,091,203.76
16043	Fremont	Idaho	13,242	718	131.00	1,328.50	276,071.41	26,074.15	779,318.39	445,455.40	23,193.83	2,230,617.60	721,526.81	51,945.99	3,019,106.72
16045	Gem	Idaho	16,719	1,187	639.00	1,713.50	569,588.23	173,132.61	1,415,920.94	989,037.88	137,051.48	4,737,523.48	1,558,626.11	347,796.06	5,768,728.00
16047	Gooding	Idaho	15,464	1,010	512.50	1,554.00	405,045.47	108,958.15	1,078,438.84	671,885.07	106,959.09	3,371,997.89	1,076,930.54	233,547.87	4,038,975.45
16049	Idaho	Idaho	16,267	1,068	462.50	1,635.00	562,534.95	149,986.90	1,403,675.32	1,097,589.93	126,767.87	5,359,555.67	1,660,124.88	323,733.73	5,857,270.46
16051	Jefferson	Idaho	26,140	1,426	362.00	2,458.00	493,282.67	68,305.12	1,239,971.86	723,185.55	57,879.80	3,782,889.38	1,216,468.22	141,619.61	4,571,955.66
16053	Jerome	Idaho	22,374	1,376	592.50	2,131.50	502,153.93	135,800.69	1,231,265.89	805,019.29	130,624.72	3,902,969.33	1,307,173.22	303,300.50	4,615,531.50
16055	Kootenai	Idaho	138,494	9,831	5,039.50	14,288.50	4,206,397.72	1,969,170.30	6,905,556.56	7,095,340.37	2,178,242.84	17,326,462.65	11,301,738.09	4,627,041.09	23,494,768.74
16057	Latah	Idaho	37,244	2,529	1,329.50	3,772.50	984,507.52	354,034.31	2,205,548.14	1,602,296.15	259,964.11	6,849,389.47	2,586,803.67	679,475.60	8,052,448.65
16059	Lemhi	Idaho	7,936	481	176.50	776.50	256,870.36	39,263.73	825,583.17	469,456.62	38,641.50	2,515,640.59	726,326.98	84,030.71	3,271,075.44
16061	Lewis	Idaho	3,821	271	134.00	413.00	139,565.72	20,585.08	517,004.42	228,286.63	22,647.40	1,560,294.72	367,852.35	49,398.15	1,827,761.75
16063	Lincoln	Idaho	5,208	317	130.50	503.50	113,547.65	19,143.70	416,642.50	180,800.08	22,949.27	1,301,131.07	294,347.73	45,031.38	1,557,441.84
16065	Madison	Idaho	37,536	1,943	411.50	3,453.50	542,799.44	67,365.20	1,235,740.25	756,645.09	55,554.97	4,173,866.17	1,299,444.53	136,827.68	4,790,579.61
16067	Minidoka	Idaho	20,069	1,148	454.50	1,841.50	477,158.48	106,844.05	1,191,660.28	787,619.81	98,171.63	3,658,928.64	1,264,778.30	231,483.33	4,499,055.60
16069	Nez Perce	Idaho	39,265	2,777	1,380.50	4,088.00	1,288,875.45	491,250.96	2,442,198.04	2,331,778.83	477,226.58	7,855,741.88	3,620,654.27	1,131,002.35	9,418,473.92
16071	Oneida	Idaho	4,286	225	70.00	373.50	103,828.70	8,412.92	403,690.26	173,197.93	11,015.81	1,407,547.47	277,026.62	21,216.52	1,658,442.59
16073	Owyhee	Idaho	11,526	838	460.00	1,273.50	339,441.72	88,835.22	846,070.64	539,226.01	85,202.86	2,909,629.02	878,667.73	188,880.86	3,527,735.19
16075	Payette	Idaho	22,623	1,724	949.50	2,465.50	713,624.40	254,913.73	1,793,066.43	1,231,281.22	236,771.67	5,357,109.67	1,944,905.61	556,319.59	6,377,913.76
16077	Power	Idaho	7,817	436	135.00	716.50	179,387.99	21,381.34	656,203.23	297,387.86	27,700.28	1,681,034.50	476,775.86	50,687.89	2,150,903.10
16079	Shoshone	Idaho	12,765	844	386.00	1,308.00	431,556.94	97,305.24	1,117,556.62	784,131.76	98,609.04	3,791,940.20	1,215,688.70	217,161.12	4,733,576.95
16081	Teton	Idaho	10,170	530	106.00	963.50	160,960.34	14,702.86	533,010.54	280,545.50	16,909.33	1,555,822.26	441,505.84	34,061.18	1,818,142.99
16083	Twin Falls	Idaho	77,230	4,843	2,203.00	7,454.50	1,929,137.96	754,034.35	3,657,269.87	3,245,239.51	672,496.26	10,395,967.71	5,174,377.47	1,666,335.48	13,100,692.79
16085	Valley	Idaho	9,862	642	312.50	979.00	337,871.56	74,799.61	1,042,053.95	574,151.63	68,233.68	2,807,623.03	912,023.20	159,751.93	3,203,987.93
16087	Washington	Idaho	10,198	754	414.50	1,066.50	362,902.18	106,486.76	939,610.95	618,719.33	84,488.68	3,091,308.37	981,621.51	206,589.91	3,671,534.98
17001	Adams	Illinois	67,103	3,413	1,275.00	5,566.50	1,584,053.06	436,090.75	3,315,972.10	2,814,588.04	413,691.26	9,066,169.22	4,398,641.10	1,004,364.33	11,351,800.87
17003	Alexander	Illinois	8,238	496	241.00	792.50	221,433.69	47,406.94	660,477.47	402,156.87	53,671.49	2,280,221.14	623,590.56	105,925.36	2,599,311.02
17005	Bond	Illinois	17,768	979	374.00	1,572.00	424,625.01	95,363.85	1,085,689.51	800,141.73	96,156.98	4,109,222.41	1,224,766.75	212,546.63	4,835,776.48
17007	Boone	Illinois	54,165	3,889	2,311.00	5,504.50	1,484,456.71	671,151.86	2,868,825.20	2,605,478.81	694,948.96	9,390,286.38	4,089,935.52	1,545,157.63	10,990,886.95

17009	Brown	Illinois	6,937	343	130.50	559.00	135,544.54	19,292.89	453,116.56	284,691.80	30,755.39	1,667,866.80	420,236.34	55,500.34	1,937,983.64
17011	Bureau	Illinois	34,978	2,261	1,130.00	3,482.50	1,050,886.81	367,053.02	2,111,465.64	1,954,685.43	373,690.70	7,111,179.81	3,005,572.24	824,172.20	8,417,255.87
17013	Calhoun	Illinois	5,089	254	107.50	416.50	118,588.57	14,442.57	383,014.83	204,951.05	16,822.13	1,665,250.04	323,539.61	32,736.25	1,940,723.55
17015	Carroll	Illinois	15,387	1,019	516.00	1,481.50	513,032.02	160,931.46	1,237,738.75	884,198.15	127,698.91	3,788,363.38	1,397,230.17	327,374.08	4,388,229.24
17017	Cass	Illinois	13,642	772	303.50	1,242.00	336,265.94	67,175.22	923,424.78	571,123.48	64,076.66	2,938,710.43	907,389.41	150,178.78	3,443,651.47
17019	Champaign	Illinois	201,081	13,089	7,067.50	19,057.50	4,882,129.82	2,403,957.38	7,919,024.79	8,247,445.41	2,706,008.62	19,424,665.28	13,129,575.23	5,277,740.47	25,983,439.00
17021	Christian	Illinois	34,800	2,060	905.50	3,277.50	957,031.90	303,541.71	1,953,989.76	1,713,753.42	249,938.74	6,236,677.61	2,670,785.32	599,783.88	7,651,236.89
17023	Clark	Illinois	16,335	1,102	594.00	1,625.00	513,859.55	145,885.63	1,218,431.01	846,023.31	141,566.76	3,777,289.67	1,359,882.86	321,361.44	4,422,162.48
17025	Clay	Illinois	13,815	873	410.50	1,345.00	388,784.19	108,653.16	942,362.49	680,820.41	92,434.05	3,502,425.34	1,069,604.59	210,233.67	4,094,753.10
17027	Clinton	Illinois	37,762	2,141	950.00	3,365.00	916,198.90	291,921.91	1,960,324.66	1,624,513.83	233,181.00	6,504,747.77	2,540,712.73	592,502.96	7,416,322.92
17029	Coles	Illinois	53,873	3,405	1,599.50	5,074.50	1,388,707.66	534,069.56	2,663,634.80	2,552,559.47	442,519.34	9,373,818.71	3,941,267.13	1,075,094.32	11,044,599.25
17031	Cook	Illinois	5,194,675	363,035	229,818.00	499,314.00	142,635,185.00	89,352,964.56	197,501,273.30	281,621,917.60	173,340,047.80	408,646,432.00	424,257,102.60	266,442,982.70	604,629,355.20
17033	Crawford	Illinois	19,817	1,298	705.00	1,916.00	576,961.61	177,877.79	1,402,093.71	1,203,563.57	208,029.73	5,851,987.86	1,780,525.17	405,143.04	6,484,072.62
17035	Cumberland	Illinois	11,048	712	346.00	1,086.50	329,139.95	65,580.85	892,427.02	588,806.43	63,338.79	3,097,147.24	917,946.37	142,867.40	3,570,885.66
17037	De Kalb	Illinois	105,160	7,117	3,974.50	10,395.50	2,585,432.43	1,214,402.30	4,640,030.35	4,237,814.31	1,115,153.21	14,005,562.22	6,823,246.74	2,617,068.95	16,951,910.49
17039	De Witt	Illinois	16,561	1,064	493.00	1,601.00	495,989.10	132,259.73	1,317,285.01	849,453.59	171,583.50	3,281,397.53	1,345,442.69	348,798.61	4,126,250.86
17041	Douglas	Illinois	19,980	1,378	689.50	2,044.50	601,331.71	185,905.31	1,413,779.21	1,121,999.96	169,820.95	4,858,548.23	1,723,331.67	387,411.73	5,962,288.66
17043	Du Page	Illinois	916,924	64,787	39,847.50	90,407.00	25,949,684.88	15,090,528.11	37,787,496.01	50,077,079.85	26,884,444.80	85,582,018.36	76,026,764.73	43,501,253.19	119,558,847.30
17045	Edgar	Illinois	18,576	1,280	694.00	1,883.50	624,549.27	204,865.44	1,470,443.96	1,103,987.54	183,221.64	4,559,968.69	1,728,536.82	431,464.13	5,355,821.92
17047	Edwards	Illinois	6,721	432	187.00	654.00	207,215.36	36,175.76	650,391.09	366,081.33	40,300.30	2,237,182.86	573,296.69	88,715.42	2,715,833.93
17049	Effingham	Illinois	34,242	2,160	1,027.00	3,468.00	931,575.00	284,464.38	1,982,668.26	1,652,826.79	265,760.49	6,789,938.66	2,584,401.79	645,092.12	8,144,460.97
17051	Fayette	Illinois	22,140	1,319	581.50	2,107.00	606,853.28	170,380.53	1,424,980.22	912,493.33	127,629.62	3,801,230.94	1,519,346.61	328,977.82	4,652,483.24
17053	Ford	Illinois	14,081	968	507.50	1,414.00	459,761.82	129,567.30	1,141,438.60	829,560.22	118,429.73	3,813,632.21	1,289,322.04	271,693.49	4,515,992.59
17055	Franklin	Illinois	39,561	2,321	1,079.50	3,613.00	1,119,026.49	368,392.89	2,268,413.23	2,251,672.69	322,271.16	9,499,489.81	3,370,699.18	771,246.91	11,158,125.25
17057	Fulton	Illinois	37,069	2,148	1,001.50	3,349.50	1,008,890.06	326,531.62	2,140,730.89	1,782,836.98	263,485.47	7,376,783.65	2,791,727.04	726,874.58	8,530,126.03
17059	Gallatin	Illinois	5,589	345	164.00	539.00	167,578.38	29,457.21	599,347.26	296,097.37	35,059.92	1,797,131.86	463,675.76	73,462.35	2,093,767.83
17061	Greene	Illinois	13,886	720	295.50	1,154.50	318,119.75	69,735.14	861,955.73	555,110.63	61,216.27	2,811,444.75	873,230.39	148,628.70	3,238,614.61
17063	Grundy	Illinois	50,063	3,497	1,821.00	5,060.00	1,309,366.80	557,991.53	2,613,135.54	2,431,378.83	664,550.59	7,853,794.93	3,740,745.62	1,353,837.38	9,430,418.25
17065	Hamilton	Illinois	8,457	522	233.00	801.50	260,966.44	50,846.82	818,562.38	400,252.45	48,742.33	2,089,166.77	661,218.89	117,948.66	2,515,997.78
17067	Hancock	Illinois	19,104	1,016	357.00	1,648.00	484,267.83	117,759.31	1,116,146.82	868,005.44	89,713.79	4,298,949.74	1,352,273.27	241,391.98	4,926,952.05
17069	Hardin	Illinois	4,320	264	114.00	406.50	141,778.28	18,020.59	461,767.29	260,448.73	19,301.06	1,814,885.03	402,227.02	43,082.08	2,177,133.48
17071	Henderson	Illinois	7,331	406	168.50	652.50	213,790.58	35,071.75	784,997.05	363,281.62	33,147.72	1,828,331.55	577,072.20	76,969.32	2,196,690.69
17073	Henry	Illinois	50,486	3,138	1,467.00	4,919.50	1,430,720.97	475,657.41	2,755,289.00	2,550,829.29	507,503.58	8,429,471.62	3,981,550.26	1,201,900.37	10,325,246.10
17075	Iroquois	Illinois	29,718	2,073	1,112.50	3,061.50	989,538.27	381,423.95	2,049,985.68	1,735,011.83	336,881.76	6,216,883.67	2,724,550.10	832,059.61	7,790,604.59
17077	Jackson	Illinois	60,218	3,269	1,461.00	5,088.00	1,307,497.26	396,362.57	2,838,579.61	2,245,950.38	386,976.61	9,147,539.33	3,553,447.64	898,061.84	10,971,238.52
17079	Jasper	Illinois	9,698	625	287.00	955.00	291,203.00	60,978.61	866,132.27	543,729.97	67,193.01	2,971,064.81	834,932.97	137,549.19	3,481,884.26
17081	Jefferson	Illinois	38,827	2,236	1,010.50	3,399.00	1,004,738.31	310,369.18	2,146,017.58	1,836,383.68	298,231.92	6,893,623.74	2,841,121.99	672,875.79	8,206,769.29
17083	Jersey	Illinois	22,985	1,173	506.00	1,840.50	533,982.81	128,906.90	1,355,975.56	868,236.29	120,164.45	4,077,450.41	1,402,219.10	276,902.29	4,833,591.47
17085	Jo Daviess	Illinois	22,678	1,488	809.00	2,228.50	774,317.79	243,077.35	1,766,283.22	1,384,233.12	211,004.09	5,147,246.92	2,158,550.91	555,941.22	6,543,276.00
17087	Johnson	Illinois	12,582	734	341.00	1,124.00	357,610.88	73,532.66	1,087,399.91	631,927.03	80,889.54	3,210,610.66	989,537.91	168,375.47	3,563,487.92
17089	Kane	Illinois	515,269	37,181	22,076.50	52,056.00	13,307,545.43	7,227,387.02	19,988,148.16	22,861,680.69	10,280,998.09	43,558,319.91	36,169,226.12	18,096,783.34	60,481,971.95
17091	Kankakee	Illinois	113,449	8,094	4,744.50	11,440.50	3,325,951.14	1,698,725.49	5,351,611.33	5,839,208.37	1,852,463.13	15,733,759.43	9,165,159.51	3,990,690.90	19,402,920.22
17093	Kendall	Illinois	114,736	8,341	4,637.50	12,051.50	2,719,975.53	1,256,851.03	4,836,742.54	4,249,609.66	1,261,744.10	12,643,017.75	6,969,585.19	2,708,907.06	15,936,722.91
17095	Knox	Illinois	52,919	3,163	1,405.50	5,011.00	1,483,665.03	540,451.25	2,979,314.16	2,738,505.68	532,014.43	10,103,873.35	4,222,170.71	1,208,109.60	12,301,900.47
17097	Lake	Illinois	703,462	49,852	30,034.50	68,322.50	18,779,087.75	10,835,514.83	27,554,190.00	36,775,627.52	18,553,774.78	62,381,150.38	55,554,715.27	29,934,543.55	86,527,575.76
17099	La Salle	Illinois	113,924	7,756	3,958.00	11,499.50	3,496,880.13	1,458,982.07	5,924,407.15	6,377,292.60	1,757,743.07	16,317,087.64	9,874,172.73	3,600,220.18	20,852,190.25
17101	Lawrence	Illinois	16,833	1,083	552.50	1,630.00	481,767.99	152,171.06	1,194,759.03	891,721.88	133,479.08	4,457,801.63	1,373,489.87	306,219.80	4,991,592.54
17103	Lee	Illinois	36,031	2,388	1,203.50	3,586.50	1,076,532.38	382,806.81	2,338,017.42	2,168,240.20	389,721.40	8,782,749.04	3,244,772.58	870,655.88	10,047,746.78
17105	Livingston	Illinois	38,950	2,607	1,425.50	3,871.00	1,157,034.59	455,119.42	2,406,314.08	2,108,956.87	441,139.00	6,824,269.86	3,265,991.46	1,024,366.45	8,423,995.74
17107	Logan	Illinois	30,305	1,801	786.50	2,742.50	808,557.91	250,003.16	1,848,899.10	1,436,379.49	224,999.50	5,078,327.44	2,244,937.40	542,694.68	6,304,590.30
17109	McDonough	Illinois	32,612	1,744	719.50	2,756.00	741,193.62	222,805.82	1,719,643.06	1,286,701.26	166,993.59	5,656,760.25	2,027,894.88	426,299.13	6,670,232.27
17111	McHenry	Illinois	308,760	22,050	13,450.00	30,608.50	8,336,228.87	4,758,750.75	12,652,067.21	14,369,386.17	5,922,054.81	31,704,322.71	22,705,615.03	11,217,931.06	40,772,373.02
17113	McLean	Illinois	169,572	10,958	5,291.50	16,483.00	4,130,275.72	1,726,971.14	7,294,693.94	7,485,947.05	2,174,604.66	18,942,956.68	11,616,222.77	4,131,166.71	25,321,274.16
17115	Macon	Illinois	110,768	7,096	3,250.50	10,968.50	3,196,797.77	1,257,796.97	5,615,219.14	6,093,060.21	1,695,111.53	15,184,438.45	9,289,857.98	3,241,829.98	19,377,589.36
17117	Macoupin	Illinois	47,765	2,589	985.00	4,059.50	1,183,323.65	353,922.50	2,446,642.62	2,171,145.29	325,112.71	8,495,535.33	3,354,468.94	825,677.72	10,050,315.52
17119	Madison	Illinois	269,282	14,207	6,008.00	21,918.00	6,034,075.07	2,347,940.95	10,153,754.23	10,884,659.13	2,852,076.02	24,992,663.82	16,918,734.19	5,737,920.15	33,885,529.38
17121	Marion	Illinois	39,437	2,391	1,197.00	3,734.50	1,093,964.27	359,311.82	2,530,404.63	1,958,073.57	362,170.02	7,255,916.32	3,052,037.83	888,777.07	8,835,933.43
17123	Marshall	Illinois	12,640	802	359.00	1,188.00	387,489.29	101,041.58	1,001,349.46	711,745.51	88,936.20	3,322,424.35	1,099,234.80	205,786.22	3,801,060.36
17125	Mason	Illinois	14,666	853	367.50	1,358.00	418,304.25	95,379.69	1,137,443.44	719,914.94	80,346.38	3,724,953.04	1,138,219.19	185,600.96	4,432,280.38
17127	Massac	Illinois	15,429	951	444.00	1,477.00	444,135.80	111,856.62	1,131,381.37	845,617.55	124,067.18	4,311,560.87	1,289,753.35	251,940.86	4,828,534.89
17129	Menard	Illinois	12,705	746	295.00	1,192.50	338,970.93	71,355.18	949,117.55	530,927.12	54,129.72	2,939,936.39	869,898.05	144,541.89	3,421,920.62
17131	Mercer	Illinois	16,434	982	431.00	1,540.50	480,839.63	117,389.46	1,259,033.86	914,330.22	97,529.98	4,320,324.44	1,395,169.85	233,649.57	5,231,537.73
17133	Monroe	Illinois	32,957	1,698	762.50	2,670.50	710,353.68	217,108.03	1,599,043.39	1,313,313.55	172,088.70	5,839,613.88	2,023,667.23	423,547.48	6,530,150.34
17135	Montgomery	Illinois	30,104	1,725	756.50	2,707.00	790,516.45	230,536.06	1,818,324.82	1,474,355.01	190,428.02	6,107,229.76	2,264,871.45	465,403.98	7,106,348.66
17137	Morgan	Illinois	35,547	1,941	764.50	3,104.50	892,694.71	236,929.88	1,980,187.80	1,672,302.83	230,111.56	7,146,744.27	2,564,997.54	540,360.63	8,264,290.83
17139	Moultrie	Illinois	14,846	964	470.50	1,472.00	429,621.00	107,708.91	1,131,631.16	767,545.90	96,665.47	3,567,894.37	1,197,166.90	233,215.92	4,104,694.64

17141	Ogle	Illinois	53,497	3,703	2,059.50	5,433.50	1,646,952.44	704,470.38	3,115,356.32	2,898,271.03	763,072.87	8,939,793.03	4,545,223.47	1,676,831.35	11,006,457.61
17143	Peoria	Illinois	186,494	11,685	5,706.50	18,466.00	4,830,700.01	2,109,201.53	8,186,498.13	9,014,640.40	2,860,338.09	20,171,748.33	13,845,340.41	5,446,780.64	26,938,381.22
17145	Perry	Illinois	22,350	1,229	471.50	1,921.50	544,701.37	138,344.67	1,310,389.96	938,930.13	114,033.12	4,131,830.76	1,483,631.49	278,848.62	4,928,367.26
17147	Piatt	Illinois	16,729	1,090	511.50	1,628.00	494,595.27	144,828.74	1,256,863.54	851,063.76	124,615.47	3,475,134.14	1,345,659.03	305,151.76	4,177,608.87
17149	Pike	Illinois	16,430	828	300.00	1,331.50	403,536.16	79,105.82	1,098,896.51	688,002.29	61,529.17	3,383,547.75	1,091,538.44	154,718.23	4,083,082.97
17151	Pope	Illinois	4,470	267	128.50	411.50	139,405.02	21,257.10	480,820.80	273,127.52	26,534.83	1,898,900.36	412,532.54	51,581.83	2,025,624.33
17153	Pulaski	Illinois	6,161	363	162.00	561.00	178,098.88	32,749.88	576,211.85	260,745.34	35,174.35	1,532,595.96	438,844.22	79,209.64	1,834,347.45
17155	Putnam	Illinois	6,006	392	201.00	593.00	191,251.55	37,552.56	607,137.51	386,224.49	52,911.58	2,538,650.26	577,476.04	97,874.41	2,941,849.45
17157	Randolph	Illinois	33,476	1,769	761.50	2,808.50	800,396.56	241,964.76	1,715,004.78	1,426,679.45	202,634.48	6,092,382.97	2,227,076.00	460,365.66	7,015,196.56
17159	Richland	Illinois	16,233	1,049	465.00	1,602.00	498,844.69	135,596.23	1,165,859.65	877,538.87	126,437.81	4,059,440.62	1,376,383.56	315,044.90	4,844,063.19
17161	Rock Island	Illinois	147,546	9,167	4,447.00	14,384.00	4,167,822.39	1,858,988.23	7,185,551.80	8,065,486.18	2,516,933.49	19,400,762.30	12,233,308.57	4,962,764.48	24,180,064.18
17163	St. Clair	Illinois	270,056	14,276	5,858.00	22,350.50	5,720,440.84	2,219,426.99	9,963,894.45	9,901,385.74	2,663,473.88	21,327,969.01	15,621,826.58	5,101,166.43	29,227,039.60
17165	Saline	Illinois	24,913	1,511	715.50	2,351.50	702,990.11	208,102.35	1,613,214.95	1,392,725.57	184,420.80	5,404,086.23	2,095,715.69	409,579.60	6,679,032.28
17167	Sangamon	Illinois	197,465	11,417	5,101.00	17,897.50	4,905,481.99	1,902,876.33	8,575,331.84	9,196,599.24	2,376,579.82	23,730,415.05	14,102,081.23	4,617,403.55	30,432,498.67
17169	Schuyler	Illinois	7,544	409	155.00	649.00	203,424.10	31,770.10	640,221.00	454,475.07	37,226.31	2,087,679.54	657,899.16	75,610.26	2,362,846.73
17171	Scott	Illinois	5,355	280	114.00	461.00	133,496.70	13,733.76	493,596.60	217,509.87	22,294.91	1,306,367.96	351,006.57	40,829.76	1,499,796.59
17173	Shelby	Illinois	22,363	1,430	573.50	2,208.00	689,038.92	192,965.49	1,552,414.24	1,282,491.23	147,936.06	5,758,657.22	1,971,530.15	408,082.26	6,730,836.75
17175	Stark	Illinois	5,994	381	179.50	597.50	179,330.82	31,645.44	554,678.53	380,667.05	37,415.88	2,202,346.52	559,997.87	79,108.39	2,505,048.41
17177	Stephenson	Illinois	47,711	3,264	1,707.50	4,866.00	1,552,972.23	577,558.08	2,923,582.99	2,912,213.65	659,156.98	8,674,348.91	4,465,185.88	1,419,188.38	10,715,857.16
17179	Tazewell	Illinois	135,394	8,427	3,713.00	13,172.50	3,661,316.82	1,387,734.54	6,513,424.81	7,334,025.38	2,030,489.62	18,343,079.90	10,995,342.20	3,937,659.26	23,220,302.40
17181	Union	Illinois	17,808	1,043	417.00	1,641.50	495,966.06	135,941.07	1,194,520.99	873,550.79	105,881.03	4,328,922.90	1,369,516.85	288,116.77	5,044,901.20
17183	Vermilion	Illinois	81,625	5,700	3,261.00	8,100.50	2,510,528.00	1,135,364.79	4,492,780.06	4,614,085.09	1,346,112.68	12,200,670.99	7,124,613.09	2,830,031.00	15,387,030.48
17185	Wabash	Illinois	11,947	766	377.00	1,164.50	346,158.43	94,660.17	846,204.22	545,674.57	82,322.70	2,531,045.38	891,833.00	197,627.25	3,058,396.70
17187	Warren	Illinois	17,707	1,016	418.00	1,612.50	448,943.39	116,236.87	1,033,196.69	783,478.71	91,813.68	3,365,437.76	1,232,422.10	223,996.21	4,092,324.22
17189	Washington	Illinois	14,716	814	321.50	1,255.00	376,972.92	89,636.53	980,143.69	661,707.74	85,168.02	3,316,198.86	1,038,680.65	208,128.58	4,017,511.47
17191	Wayne	Illinois	16,760	1,028	473.50	1,587.00	495,007.24	132,917.75	1,209,080.76	868,119.53	104,686.68	3,885,145.80	1,363,126.76	280,681.28	4,435,594.36
17193	White	Illinois	14,665	921	419.50	1,412.00	455,526.79	122,354.01	1,132,371.47	814,728.93	119,271.52	3,977,398.74	1,270,255.73	290,804.79	4,760,524.57
17195	Whiteside	Illinois	58,498	3,809	1,892.50	5,712.50	1,749,824.75	648,449.80	3,202,375.60	3,178,397.76	597,481.35	8,902,483.92	4,928,222.51	1,418,426.98	11,592,247.76
17197	Will	Illinois	677,560	48,715	29,936.50	68,611.00	17,183,804.78	9,817,296.50	25,600,343.78	29,689,663.92	14,317,389.58	55,625,491.95	46,873,468.70	24,855,856.56	76,249,303.32
17199	Williamson	Illinois	66,357	3,904	1,751.00	5,857.00	1,763,878.95	648,692.77	3,294,572.71	3,147,615.39	692,120.84	11,746,031.42	4,911,494.34	1,470,719.84	13,879,927.85
17201	Winnebago	Illinois	295,266	20,750	11,796.50	29,633.50	8,629,007.05	4,498,866.78	13,356,067.79	15,895,485.87	6,475,695.70	34,459,289.55	24,524,492.91	11,720,839.79	45,542,930.35
17203	Woodford	Illinois	38,664	2,519	1,174.00	3,751.50	1,078,181.04	355,030.08	2,147,530.53	1,889,188.56	274,889.40	6,368,395.74	2,967,369.60	664,034.74	7,776,853.29
18001	Adams	Indiana	34,387	2,574	1,495.00	3,657.50	1,036,925.66	435,252.54	2,154,369.41	1,617,148.18	348,157.26	5,804,899.79	2,654,073.84	892,094.82	7,036,601.81
18003	Allen	Indiana	355,329	25,485	14,634.00	35,795.00	9,813,716.68	5,183,631.62	15,062,536.85	17,376,170.38	7,083,949.09	34,355,907.32	27,189,887.06	13,118,502.27	46,597,569.67
18005	Bartholomew	Indiana	76,794	5,710	3,375.00	7,928.50	2,392,550.42	1,117,353.47	4,326,444.68	4,320,489.96	1,301,404.69	11,844,165.64	6,713,040.38	2,699,457.29	14,868,298.47
18007	Benton	Indiana	8,854	644	379.50	906.50	293,823.56	75,803.69	835,706.93	521,895.76	75,977.04	2,618,281.50	815,719.32	172,354.64	3,321,944.64
18009	Blackford	Indiana	12,766	908	525.00	1,321.00	405,292.34	135,477.96	997,612.90	705,278.31	112,377.10	2,997,687.04	1,110,570.65	266,472.89	3,613,455.84
18011	Boone	Indiana	56,640	4,228	2,618.00	5,811.50	1,654,051.45	799,774.73	3,039,428.51	2,740,247.50	706,504.66	9,504,564.37	4,394,298.94	1,656,969.53	11,185,549.93
18013	Brown	Indiana	15,242	1,078	608.00	1,509.00	552,486.91	179,236.64	1,319,405.28	971,712.01	117,188.07	4,501,703.18	1,524,198.93	337,712.34	5,489,333.34
18015	Carroll	Indiana	20,155	1,459	877.50	2,063.00	656,275.49	228,104.94	1,487,962.27	1,177,038.81	186,204.99	5,194,884.06	1,833,314.30	452,963.06	5,997,301.51
18017	Cass	Indiana	38,966	2,831	1,606.00	4,000.00	1,203,287.82	478,708.46	2,404,368.20	2,030,707.93	438,122.83	6,556,644.56	3,233,995.75	1,039,018.97	8,004,128.94
18019	Clark	Indiana	110,232	8,021	4,986.50	11,184.50	3,296,131.62	1,787,085.47	5,669,854.59	5,647,639.74	1,716,797.50	15,058,433.15	8,943,770.97	3,904,896.05	19,331,249.36
18021	Clay	Indiana	26,890	1,897	1,075.00	2,776.50	835,477.51	317,854.91	1,834,538.62	1,405,987.41	224,242.79	5,711,742.84	2,241,464.92	627,416.26	7,112,387.68
18023	Clinton	Indiana	33,224	2,454	1,428.00	3,373.50	1,015,452.71	417,977.64	2,249,631.47	1,791,199.68	386,462.74	7,105,760.30	2,806,652.39	912,600.57	8,277,406.55
18025	Crawford	Indiana	10,713	760	431.00	1,088.00	332,820.79	104,092.68	852,375.98	572,868.22	75,540.85	2,925,905.80	905,689.01	196,757.01	3,640,426.33
18027	Daviess	Indiana	31,648	2,325	1,259.50	3,385.50	935,172.25	330,251.86	1,934,635.81	1,556,815.53	293,037.45	6,337,494.68	2,491,987.78	713,163.41	7,462,770.51
18029	Dearborn	Indiana	50,047	3,616	2,209.00	5,034.00	1,522,972.59	693,010.54	2,940,414.46	2,640,701.87	613,877.77	8,286,635.50	4,163,674.46	1,408,983.34	10,345,136.30
18031	Decatur	Indiana	25,740	1,908	1,193.00	2,695.50	796,502.29	304,707.86	1,586,302.86	1,435,985.54	303,838.48	5,479,096.97	2,232,487.83	654,430.92	6,568,043.93
18033	De Kalb	Indiana	42,223	3,058	1,715.50	4,307.50	1,243,830.50	483,959.17	2,617,539.83	2,207,988.87	479,202.65	7,208,709.30	3,451,819.38	1,042,296.99	8,587,939.48
18035	Delaware	Indiana	117,671	8,263	5,041.00	11,459.50	3,538,424.15	1,784,021.22	5,931,658.11	6,360,171.49	1,939,490.62	16,932,666.65	9,898,595.64	4,335,000.78	21,392,798.54
18037	Dubois	Indiana	41,889	2,959	1,586.00	4,347.50	1,245,390.95	506,142.09	2,436,707.63	2,111,316.25	484,489.87	7,522,186.44	3,356,707.20	1,076,565.55	9,114,211.65
18039	Elkhart	Indiana	197,559	14,460	8,364.00	20,831.50	5,558,143.39	2,842,498.18	9,231,281.94	9,388,826.67	3,684,638.71	22,481,605.89	14,946,970.06	7,052,736.19	29,651,475.78
18041	Fayette	Indiana	24,277	1,776	1,071.50	2,498.50	803,216.40	305,614.91	1,860,206.14	1,395,968.55	248,876.20	6,016,887.61	2,199,184.95	616,625.11	6,988,957.29
18043	Floyd	Indiana	74,578	5,311	3,052.00	7,438.00	2,193,356.15	1,034,498.27	3,866,592.61	3,687,460.29	1,122,175.07	10,538,579.26	5,880,816.44	2,406,481.79	13,302,777.24
18045	Fountain	Indiana	17,240	1,216	707.50	1,737.50	554,478.56	179,794.22	1,252,792.75	1,061,778.44	163,587.39	4,549,092.26	1,616,257.00	379,643.84	5,219,819.22
18047	Franklin	Indiana	23,087	1,691	1,071.50	2,311.50	713,025.29	275,953.75	1,576,283.95	1,204,138.00	215,470.30	4,950,618.65	1,917,163.29	571,280.18	5,862,764.89
18049	Fulton	Indiana	20,836	1,513	871.50	2,212.50	683,186.78	249,239.52	1,476,314.91	1,215,104.43	216,789.91	5,360,084.46	1,898,291.20	517,299.75	6,058,136.29
18051	Gibson	Indiana	33,503	2,252	1,156.50	3,400.00	990,118.61	337,689.14	2,123,839.62	1,884,166.31	411,215.08	7,131,702.24	2,874,284.92	844,474.74	8,416,413.62
18053	Grant	Indiana	70,061	5,002	3,145.50	7,146.50	2,263,169.70	1,173,033.79	3,903,499.08	4,236,173.62	1,130,128.92	12,499,320.11	6,499,343.32	2,619,587.60	15,501,879.34
18055	Greene	Indiana	33,165	2,312	1,250.50	3,303.50	1,031,207.93	383,600.08	2,091,368.47	1,785,381.49	360,043.81	7,355,724.24	2,816,589.41	845,743.59	8,310,369.81
18057	Hamilton	Indiana	274,569	20,478	12,257.50	28,825.00	7,126,121.51	3,832,339.94	11,210,458.10	12,226,037.95	4,987,459.17	25,051,599.73	19,352,159.47	9,462,579.99	34,699,034.37
18059	Hancock	Indiana	70,002	5,060	3,077.00	7,049.50	2,044,636.40	949,226.96	4,023,416.42	3,714,749.49	969,205.71	11,590,023.16	5,759,385.89	2,156,626.28	13,907,230.79
18061	Harrison	Indiana	39,364	2,773	1,648.50	3,957.50	1,188,842.34	489,558.10	2,382,951.07	2,052,808.77	423,921.82	8,105,243.73	3,241,651.10	1,043,821.72	9,420,301.90
18063	Hendricks	Indiana	145,448	10,654	6,509.50	14,891.00	4,021,075.65	2,112,249.06	6,732,736.63	6,692,414.43	2,128,103.00	17,598,760.42	10,713,490.08	4,584,609.85	22,750,393.46
18065	Henry	Indiana	49,462	3,555	2,087.00	4,996.50	1,581,672.84	701,730.73	2,982,156.76	2,833,885.84	636,619.51	9,169,183.97	4,415,558.68	1,536,857.34	10,884,224.27
18067	Howard	Indiana	82,752	5,974	3,536.00	8,427.00	2,667,751.00	1,265,980.93	4,795,513.13	5,060,504.92	1,639,774.31	12,885,243.73	7,728,255.92	3,144,760.25	16,421,721.71

18069	Huntington	Indiana	37,124	2,598	1,476.00	3,662.50	1,117,423.30	464,968.99	2,282,842.20	1,843,656.07	391,276.24	7,580,533.66	2,961,079.37	945,199.38	8,912,493.85
18071	Jackson	Indiana	42,376	3,114	1,903.00	4,346.50	1,283,810.10	599,920.83	2,527,839.85	2,338,755.62	567,851.52	9,127,236.35	3,622,565.72	1,336,600.30	10,525,104.01
18073	Jasper	Indiana	33,478	2,395	1,358.50	3,377.00	999,242.16	367,684.05	2,107,767.85	1,633,259.61	368,955.26	6,067,195.21	2,632,501.77	831,648.48	7,425,535.20
18075	Jay	Indiana	21,253	1,537	899.50	2,167.00	640,867.95	235,401.69	1,546,989.83	1,143,683.98	195,556.06	5,106,806.34	1,784,551.92	473,264.84	5,983,840.02
18077	Jefferson	Indiana	32,428	2,357	1,450.50	3,299.00	1,011,186.36	442,706.25	1,974,790.15	1,793,777.00	360,935.12	6,787,503.27	2,804,963.36	868,008.78	7,723,615.46
18079	Jennings	Indiana	28,525	2,090	1,273.50	2,866.00	831,771.01	347,954.21	1,632,906.35	1,443,548.06	307,780.37	5,265,804.22	2,275,319.07	746,646.11	6,401,420.10
18081	Johnson	Indiana	139,654	10,279	6,499.50	14,093.50	4,023,979.12	2,261,174.28	6,314,988.63	6,730,816.07	2,328,125.31	17,214,292.40	10,754,795.19	5,113,034.44	22,345,482.20
18083	Knox	Indiana	38,440	2,558	1,356.50	3,746.50	1,125,788.17	451,686.34	2,162,311.86	1,938,896.42	385,235.95	7,180,027.59	3,064,684.59	903,353.23	8,281,464.22
18085	Kosciusko	Indiana	77,358	5,552	3,196.50	7,932.50	2,279,105.16	1,045,478.52	4,137,371.49	3,969,520.95	1,228,164.70	12,487,895.29	6,248,626.11	2,621,868.88	15,357,216.12
18087	Lagrange	Indiana	37,128	2,884	1,711.50	4,162.00	1,030,707.28	411,873.02	2,121,879.94	1,721,145.97	381,576.37	6,761,156.69	2,751,853.25	889,937.79	7,857,791.28
18089	Lake	Indiana	496,005	35,635	21,011.00	49,203.50	14,567,704.91	7,846,549.87	21,530,974.27	25,862,175.68	11,828,485.02	46,492,773.91	40,429,880.59	20,537,636.17	65,598,101.97
18091	La Porte	Indiana	111,467	7,796	4,543.00	11,143.00	3,379,203.42	1,696,447.37	5,655,785.18	5,683,472.80	1,767,356.00	14,995,419.19	9,062,676.22	3,749,601.22	19,370,135.77
18093	Lawrence	Indiana	46,134	3,301	1,853.50	4,720.00	1,495,446.26	647,840.68	2,783,669.07	2,673,765.01	581,294.86	8,835,094.43	4,169,211.27	1,376,179.46	10,701,896.90
18095	Madison	Indiana	131,636	9,584	5,867.00	13,404.00	4,187,993.20	2,196,829.06	6,855,629.40	7,087,337.48	2,395,636.37	18,557,705.93	11,275,330.68	5,123,896.58	23,589,375.08
18097	Marion	Indiana	903,393	66,282	40,595.50	91,278.00	24,888,005.42	14,729,513.18	35,461,919.94	45,997,029.70	23,744,023.98	78,767,619.42	70,885,035.12	39,973,466.87	109,240,013.30
18099	Marshall	Indiana	47,051	3,378	1,930.50	4,910.00	1,448,910.87	551,025.97	2,619,774.17	2,482,304.83	568,171.26	9,552,548.87	3,931,215.70	1,282,920.47	10,879,587.24
18101	Martin	Indiana	10,334	726	380.00	1,072.50	324,966.76	86,360.07	924,826.01	601,461.77	152,091.64	2,622,871.75	926,428.53	255,185.61	3,134,860.09
18103	Miami	Indiana	36,903	2,600	1,470.50	3,746.00	1,109,700.97	448,981.49	2,230,633.18	1,918,579.83	383,968.77	6,779,851.62	3,028,280.81	946,481.62	7,899,385.73
18105	Monroe	Indiana	137,974	9,274	5,482.00	12,906.50	3,509,937.77	1,741,360.04	5,970,603.08	5,633,826.75	1,713,102.76	14,835,420.54	9,143,764.52	3,907,727.62	19,205,519.20
18107	Montgomery	Indiana	38,124	2,728	1,671.00	3,881.00	1,187,897.18	515,833.21	2,353,467.65	2,217,260.86	538,411.31	7,339,525.52	3,405,158.04	1,177,760.15	8,974,645.98
18109	Morgan	Indiana	68,894	4,971	3,081.00	6,875.50	2,102,266.91	1,005,561.00	3,890,811.20	3,573,666.27	947,151.80	11,683,522.15	5,675,933.18	2,083,980.03	14,475,737.68
18111	Newton	Indiana	14,244	1,008	575.50	1,435.50	444,991.15	158,975.55	1,097,278.55	842,359.51	133,482.97	4,874,781.33	1,287,350.66	330,365.51	5,409,205.33
18113	Noble	Indiana	47,536	3,371	1,906.00	4,874.00	1,362,937.69	557,818.72	2,644,160.86	2,216,148.67	487,849.23	7,664,346.96	3,579,086.35	1,188,026.77	9,276,921.59
18115	Ohio	Indiana	6,128	445	266.00	609.00	205,426.00	48,039.60	683,022.58	316,079.20	55,942.23	1,999,758.94	521,505.21	113,467.75	2,382,763.94
18117	Orange	Indiana	19,840	1,426	846.00	2,014.00	631,155.71	232,187.56	1,540,951.41	1,165,904.80	189,821.91	5,415,118.49	1,797,060.51	470,487.61	6,063,759.36
18119	Owen	Indiana	21,575	1,500	843.00	2,159.00	671,325.71	249,953.05	1,569,085.36	1,130,164.78	199,352.26	4,717,802.10	1,801,490.49	513,966.52	5,436,369.40
18121	Parke	Indiana	17,339	1,197	652.00	1,736.50	558,516.62	172,051.42	1,538,051.56	862,456.87	126,868.26	3,879,423.12	1,420,973.49	338,745.81	4,859,940.29
18123	Perry	Indiana	19,338	1,332	670.50	1,951.50	590,956.39	182,480.14	1,487,552.98	1,009,288.02	174,951.63	4,266,068.04	1,600,244.42	386,324.00	4,882,168.12
18125	Pike	Indiana	12,845	873	466.00	1,316.00	394,802.67	102,399.97	1,040,574.39	809,499.28	138,027.06	4,027,010.87	1,204,301.95	273,586.49	4,648,418.28
18127	Porter	Indiana	164,343	11,718	7,228.00	16,225.00	4,826,364.64	2,577,689.81	7,687,934.19	8,405,481.22	3,206,180.31	19,726,371.74	13,231,845.87	6,181,705.72	25,574,485.30
18129	Posey	Indiana	25,910	1,667	815.50	2,540.50	736,522.07	222,425.42	1,694,557.21	1,391,574.44	271,725.86	5,364,758.84	2,128,096.51	546,028.89	6,515,321.32
18131	Pulaski	Indiana	13,402	965	539.50	1,357.00	448,049.44	136,730.91	1,140,614.52	790,285.93	127,357.09	3,631,947.70	1,238,335.37	291,894.02	4,227,979.82
18133	Putnam	Indiana	37,963	2,665	1,589.00	3,833.00	1,117,521.26	458,692.78	2,181,259.36	1,940,710.83	361,428.04	8,468,751.26	3,058,232.09	933,139.04	9,404,892.12
18135	Randolph	Indiana	26,171	1,899	1,168.50	2,675.00	867,795.09	332,046.04	1,783,885.62	1,525,926.99	290,538.50	6,260,468.61	2,393,722.08	736,577.18	7,453,564.76
18137	Ripley	Indiana	28,818	2,141	1,284.50	2,992.50	894,603.96	359,267.78	1,762,312.67	1,560,606.38	365,222.63	4,888,786.12	2,455,210.35	848,886.52	6,067,696.74
18139	Rush	Indiana	17,392	1,279	778.50	1,799.00	543,879.30	206,050.60	1,208,932.15	1,027,736.44	183,045.69	4,709,965.41	1,571,615.74	429,136.33	5,308,077.91
18141	St. Joseph	Indiana	266,931	18,994	10,572.50	26,732.50	7,688,480.58	4,112,954.31	12,273,469.68	13,685,790.64	5,614,776.70	29,810,863.58	21,374,271.23	10,263,228.06	39,088,749.69
18143	Scott	Indiana	24,181	1,780	1,154.00	2,402.00	773,705.75	279,632.25	1,815,212.16	1,269,277.69	227,887.83	4,793,016.69	2,042,983.44	547,736.90	6,253,588.64
18145	Shelby	Indiana	44,436	3,238	2,003.50	4,522.50	1,387,367.67	612,798.29	2,618,891.27	2,438,951.67	596,479.63	8,717,940.66	3,826,319.34	1,303,762.15	10,931,269.98
18147	Spencer	Indiana	20,952	1,429	735.50	2,066.00	636,547.82	196,044.44	1,476,002.74	1,143,739.52	176,175.33	5,931,722.74	1,780,287.34	424,566.16	6,874,756.52
18149	Starke	Indiana	23,363	1,674	1,002.50	2,380.50	739,127.04	289,291.16	1,637,643.70	1,186,540.55	222,231.10	4,638,573.60	1,925,667.60	579,216.96	5,816,381.56
18151	Steuben	Indiana	34,185	2,396	1,364.50	3,405.00	1,061,107.10	418,886.26	2,154,184.95	1,699,392.29	324,301.72	6,646,386.03	2,760,499.38	841,611.82	7,832,656.15
18153	Sullivan	Indiana	21,475	1,408	706.50	2,057.50	607,869.99	192,955.49	1,402,680.58	1,028,384.49	163,063.18	4,514,587.54	1,636,254.48	386,913.96	5,709,420.94
18155	Switzerland	Indiana	10,613	793	506.00	1,096.00	345,400.01	98,456.75	981,349.98	575,700.39	97,443.25	3,447,624.55	921,100.39	221,523.32	3,895,234.47
18157	Tippecanoe	Indiana	172,780	12,101	7,104.50	16,988.00	4,383,078.67	2,133,090.29	7,250,541.90	7,491,324.24	2,495,009.60	19,255,221.42	11,874,402.91	5,059,906.07	24,373,531.98
18159	Tipton	Indiana	15,936	1,147	692.00	1,621.00	538,849.21	182,258.37	1,431,793.65	994,560.90	174,700.12	4,193,597.41	1,533,410.10	394,339.52	4,848,214.14
18161	Union	Indiana	7,516	538	325.50	775.00	222,198.02	59,834.69	659,985.47	424,304.51	51,342.92	1,902,690.30	646,502.54	120,987.41	2,347,352.14
18163	Vanderburgh	Indiana	179,703	11,803	6,176.00	17,633.50	4,995,824.60	2,313,622.40	8,256,499.16	9,159,252.20	3,086,349.26	22,417,469.81	14,155,076.80	5,811,815.23	28,113,871.80
18165	Vermillion	Indiana	16,212	1,130	631.00	1,629.50	507,711.67	173,579.26	1,213,572.00	1,007,419.53	202,506.63	4,080,274.99	1,515,131.19	390,702.01	4,736,974.55
18167	Vigo	Indiana	107,848	7,323	4,054.50	10,622.00	3,042,092.06	1,370,426.93	5,364,963.77	5,091,843.52	1,470,024.87	13,611,448.57	8,133,935.58	3,184,131.34	17,660,148.11
18169	Wabash	Indiana	32,888	2,344	1,381.50	3,373.00	1,086,139.07	453,421.94	2,140,297.89	1,893,343.04	375,918.47	6,718,731.98	2,979,482.11	1,014,956.62	8,200,949.64
18171	Warren	Indiana	8,508	596	335.00	855.50	272,413.02	69,640.63	779,340.83	543,761.17	68,095.34	3,292,920.38	816,174.19	155,461.40	3,778,831.02
18173	Warrick	Indiana	59,689	4,038	2,065.00	5,977.00	1,723,509.07	685,467.84	3,383,925.62	3,051,887.42	737,932.36	9,396,152.20	4,775,396.49	1,611,454.62	11,574,788.30
18175	Washington	Indiana	28,262	2,044	1,256.50	2,854.50	839,401.86	354,214.49	1,962,815.71	1,343,783.07	257,746.78	5,133,108.58	2,183,184.93	661,940.37	6,344,194.04
18177	Wayne	Indiana	68,917	4,951	2,778.00	6,992.00	2,173,541.09	969,493.42	3,718,232.72	3,944,088.35	1,023,303.96	11,157,054.13	6,117,629.43	2,215,790.83	13,598,932.04
18179	Wells	Indiana	27,636	1,984	1,152.50	2,851.50	891,327.38	348,925.26	1,967,171.07	1,630,143.56	290,645.05	6,326,842.07	2,521,470.93	748,648.12	7,638,224.16
18181	White	Indiana	24,643	1,782	1,028.00	2,544.50	815,885.53	313,957.21	1,799,190.80	1,403,103.15	269,029.05	5,071,813.79	2,218,988.68	657,057.46	6,177,121.21
18183	Whitley	Indiana	33,292	2,342	1,405.00	3,305.00	996,217.45	412,212.53	1,949,449.96	1,766,203.44	359,376.96	8,210,235.67	2,762,420.89	872,903.93	9,480,149.69
19001	Adair	Iowa	7,682	370	122.50	622.50	178,331.91	22,191.04	618,553.21	313,530.21	26,176.23	2,072,196.83	491,862.12	51,087.73	2,351,606.21
19003	Adams	Iowa	4,029	194	72.00	321.50	99,966.76	11,479.28	376,660.52	156,560.04	12,741.29	1,147,155.56	256,526.80	27,133.70	1,316,456.30
19005	Allamakee	Iowa	14,330	966	467.00	1,459.50	494,752.58	124,915.41	1,233,558.78	841,810.16	109,288.01	3,985,195.77	1,336,562.73	276,710.09	4,562,408.30
19007	Appanoose	Iowa	12,887	621	235.50	1,010.50	324,247.35	56,935.68	994,043.44	521,548.08	49,213.10	2,752,264.40	845,795.44	122,711.82	3,538,870.76
19009	Audubon	Iowa	6,119	292	90.00	491.50	155,215.91	14,793.35	565,911.69	299,286.62	19,561.60	1,811,210.55	454,502.53	36,361.36	2,224,271.71
19011	Benton	Iowa	26,076	1,498	576.00	2,341.00	637,976.50	163,886.37	1,393,357.23	1,142,067.32	130,812.32	4,502,683.01	1,780,043.82	319,609.79	5,309,942.25
19013	Black Hawk	Iowa	131,090	7,623	3,454.00	11,961.00	3,193,807.44	1,166,150.68	5,849,551.27	5,682,883.11	1,346,329.76	15,128,722.50	8,876,690.55	2,985,303.91	19,285,962.17
19015	Boone	Iowa	26,306	1,285	500.50	2,038.00	571,461.24	127,373.43	1,426,839.79	1,016,980.11	119,552.44	4,821,435.92	1,588,441.35	276,269.88	5,361,223.61

19017	Bremer	Iowa	24,276	1,450	657.00	2,220.00	673,489.90	200,173.58	1,598,606.33	1,177,141.43	170,164.64	4,603,180.16	1,850,631.34	416,554.93	5,771,305.85
19019	Buchanan	Iowa	20,958	1,294	576.00	2,022.00	546,221.61	135,366.09	1,298,180.17	997,395.63	124,460.61	4,452,486.81	1,543,617.24	300,177.14	5,127,378.11
19021	Buena Vista	Iowa	20,260	1,061	390.50	1,737.00	446,760.74	93,102.49	1,131,138.75	812,049.37	82,666.90	3,787,763.55	1,258,810.11	192,416.97	4,706,095.44
19023	Butler	Iowa	14,867	895	394.00	1,384.00	441,279.13	102,243.27	1,260,974.54	768,285.05	89,034.61	4,005,503.21	1,209,564.19	217,139.12	4,605,688.55
19025	Calhoun	Iowa	9,670	480	143.00	805.00	251,638.72	37,994.53	803,961.88	494,849.81	33,501.81	2,605,472.01	746,488.53	76,981.71	2,954,306.33
19027	Carroll	Iowa	20,816	1,031	366.50	1,755.00	472,775.00	99,929.15	1,239,854.80	849,088.90	88,035.34	4,159,104.62	1,321,863.90	204,442.68	5,212,493.50
19029	Cass	Iowa	13,956	668	230.00	1,109.50	341,753.03	56,993.33	955,313.74	613,050.86	50,370.83	2,914,307.98	954,803.89	112,834.36	3,466,947.26
19031	Cedar	Iowa	18,499	1,121	457.50	1,737.00	499,541.10	123,864.01	1,191,477.66	942,339.16	93,257.64	4,431,302.08	1,441,880.25	250,830.76	5,022,915.89
19033	Cerro Gordo	Iowa	44,151	2,539	1,209.00	3,900.00	1,214,539.09	402,078.15	2,515,900.12	2,276,213.11	358,938.80	7,628,335.74	3,490,752.20	832,856.02	9,047,425.78
19035	Cherokee	Iowa	12,072	600	169.50	978.00	315,083.86	46,354.42	840,791.17	597,412.74	44,621.56	3,214,350.96	912,496.61	109,781.56	3,690,908.39
19037	Chickasaw	Iowa	12,439	784	373.50	1,215.00	384,690.34	95,048.89	1,154,887.99	673,119.32	84,661.41	3,321,983.93	1,057,809.66	195,582.01	3,735,519.27
19039	Clarke	Iowa	9,286	457	172.00	743.50	199,869.84	29,885.27	592,515.01	405,187.89	30,737.98	2,172,973.72	605,057.73	68,889.41	2,842,720.06
19041	Clay	Iowa	16,667	853	267.50	1,434.50	402,729.07	64,336.81	1,066,160.55	689,478.16	64,341.85	3,237,342.35	1,092,207.22	153,978.27	3,859,888.43
19043	Clayton	Iowa	18,129	1,167	587.50	1,805.50	569,126.03	159,926.40	1,359,360.04	954,441.52	132,138.56	3,903,541.50	1,523,567.55	330,285.24	4,924,201.36
19045	Clinton	Iowa	49,116	3,124	1,420.50	4,865.50	1,406,625.85	520,032.46	2,892,300.85	2,408,558.27	457,538.45	8,464,458.83	3,815,184.11	1,058,255.18	10,324,108.34
19047	Crawford	Iowa	17,096	844	272.00	1,391.00	360,304.65	58,107.41	961,463.69	661,115.04	56,594.94	3,644,367.57	1,021,419.69	128,917.42	4,121,682.49
19049	Dallas	Iowa	66,135	3,277	1,360.50	5,393.00	1,164,945.64	390,846.52	2,552,128.61	2,139,670.47	375,248.09	8,484,411.12	3,304,616.11	798,331.54	9,787,592.71
19051	Davis	Iowa	8,753	459	176.00	770.00	203,119.96	33,775.95	656,932.72	350,342.28	29,657.77	1,865,365.42	553,462.24	72,721.45	2,227,901.16
19053	Decatur	Iowa	8,457	403	129.50	669.50	183,711.43	24,999.13	607,602.51	293,562.87	22,091.50	1,740,077.47	477,274.30	51,748.28	2,060,391.33
19055	Delaware	Iowa	17,764	1,108	501.50	1,714.50	490,068.63	135,637.03	1,167,296.76	828,335.95	116,180.25	3,837,643.34	1,318,404.58	304,951.65	4,473,397.49
19057	Des Moines	Iowa	40,325	2,262	789.00	3,687.00	1,030,706.63	263,261.48	2,319,977.90	1,866,900.86	221,752.21	7,132,674.58	2,897,607.50	530,922.01	8,131,029.15
19059	Dickinson	Iowa	16,667	846	258.00	1,396.00	460,725.01	67,921.82	1,242,507.44	883,372.84	59,359.84	4,230,930.15	1,344,097.85	137,574.84	5,009,274.07
19061	Dubuque	Iowa	93,653	6,090	2,959.00	9,204.50	2,635,835.54	1,057,225.93	4,568,524.71	4,708,303.27	1,064,279.88	13,593,810.42	7,344,138.81	2,417,707.34	16,698,151.61
19063	Emmet	Iowa	10,302	547	206.00	909.50	263,393.43	43,511.33	837,945.32	481,088.39	43,664.32	2,764,669.56	744,481.82	95,704.64	3,057,108.12
19065	Fayette	Iowa	20,880	1,299	580.00	2,015.50	624,384.95	189,995.59	1,479,121.73	1,130,887.60	152,000.55	5,027,598.14	1,755,272.55	361,585.89	6,132,470.29
19067	Floyd	Iowa	16,303	998	470.50	1,553.50	482,509.87	132,706.96	1,150,782.15	954,518.00	108,222.51	4,242,799.77	1,437,027.86	277,093.73	5,090,404.35
19069	Franklin	Iowa	10,680	595	231.50	948.00	272,852.24	47,188.29	815,440.26	516,862.38	52,198.49	2,570,541.15	789,714.62	109,913.29	2,945,668.43
19071	Fremont	Iowa	7,441	367	145.00	589.00	172,524.40	28,022.48	530,791.44	304,922.24	31,066.38	1,541,511.69	477,446.64	63,383.49	1,941,996.81
19073	Greene	Iowa	9,336	455	157.50	721.00	231,394.31	37,923.34	740,651.93	456,887.48	35,995.56	2,623,113.99	688,281.80	86,199.61	3,177,711.55
19075	Grundy	Iowa	12,453	690	280.50	1,103.00	331,872.51	74,802.30	960,894.90	589,566.85	69,299.59	2,982,313.28	921,439.36	161,909.21	3,608,173.93
19077	Guthrie	Iowa	10,954	516	168.00	881.50	260,889.80	35,233.20	816,094.34	496,104.81	36,703.75	2,884,137.11	756,994.61	80,940.28	3,241,666.82
19079	Hamilton	Iowa	15,673	819	317.00	1,343.50	382,600.04	75,711.62	1,022,803.43	660,233.14	67,579.12	3,732,846.67	1,042,833.18	160,090.70	4,372,646.30
19081	Hancock	Iowa	11,341	631	237.00	997.50	307,411.59	57,092.15	921,177.66	563,607.76	58,047.91	2,462,257.91	871,019.35	126,154.51	3,031,183.73
19083	Hardin	Iowa	17,534	960	389.50	1,573.50	470,599.37	104,775.79	1,152,802.30	933,292.46	96,104.14	4,458,230.18	1,403,891.83	215,889.44	5,260,165.41
19085	Harrison	Iowa	14,928	737	261.50	1,209.50	336,406.47	52,356.68	913,198.21	620,751.49	49,883.73	3,226,481.55	957,157.96	115,905.34	3,855,214.41
19087	Henry	Iowa	20,145	1,096	441.00	1,783.00	489,982.21	107,300.02	1,221,133.68	832,814.74	108,145.22	3,726,135.83	1,322,796.95	253,139.44	4,553,385.69
19089	Howard	Iowa	9,566	620	294.50	931.00	297,455.21	69,253.70	789,527.77	487,156.95	60,832.64	2,370,747.51	784,612.16	141,370.45	2,840,095.19
19091	Humboldt	Iowa	9,815	517	186.50	830.00	245,843.63	36,883.23	747,229.69	438,725.91	43,675.57	2,708,938.99	684,569.54	88,117.76	3,157,915.81
19093	Ida	Iowa	7,089	348	107.00	590.00	167,318.39	17,680.67	502,381.92	311,322.93	23,801.69	2,192,975.49	478,641.32	47,022.10	2,467,806.90
19095	Iowa	Iowa	16,355	921	397.50	1,446.00	407,520.21	106,190.41	1,139,901.44	693,682.90	88,282.57	3,774,758.28	1,101,203.11	216,140.32	4,257,327.85
19097	Jackson	Iowa	19,848	1,271	642.50	1,928.00	630,146.59	179,971.26	1,424,288.65	1,226,320.75	148,924.67	5,236,350.08	1,856,467.33	369,513.98	6,097,991.02
19099	Jasper	Iowa	36,842	1,897	776.50	3,009.00	847,806.13	225,021.64	1,833,433.16	1,465,481.63	211,884.78	5,852,118.83	2,313,287.76	486,811.98	6,887,047.06
19101	Jefferson	Iowa	16,843	858	354.00	1,348.00	424,499.89	83,913.35	1,140,383.11	676,744.98	77,161.59	3,571,566.55	1,101,244.87	181,917.43	4,219,358.58
19103	Johnson	Iowa	130,882	7,179	2,933.00	11,270.00	2,623,047.10	923,528.81	4,638,559.38	4,267,578.59	890,236.21	12,710,921.09	6,890,625.69	1,941,651.91	16,752,837.88
19105	Jones	Iowa	20,638	1,273	593.00	1,965.50	574,733.51	180,279.98	1,336,808.74	980,207.96	135,876.34	4,040,333.75	1,554,941.47	337,127.07	4,727,858.87
19107	Keokuk	Iowa	10,511	560	200.00	915.50	269,767.88	46,751.96	777,962.38	461,848.78	40,974.31	2,636,733.47	731,616.66	91,384.59	3,038,163.93
19109	Kossuth	Iowa	15,543	854	316.00	1,385.50	436,856.26	97,266.83	1,097,171.11	783,811.05	86,728.55	4,013,279.43	1,220,667.31	198,147.68	4,880,573.74
19111	Lee	Iowa	35,862	1,899	801.00	3,108.00	870,540.21	267,498.42	1,927,799.20	1,719,350.16	223,743.74	6,260,937.24	2,589,890.37	558,129.01	7,341,638.57
19113	Linn	Iowa	211,226	12,665	5,779.50	19,911.00	5,131,870.86	1,939,644.61	8,463,154.72	9,556,441.93	2,742,494.57	22,061,292.40	14,688,312.79	5,189,554.54	29,638,247.60
19115	Louisa	Iowa	11,387	655	286.00	1,050.00	274,738.96	56,224.73	832,926.31	462,753.43	56,649.58	2,147,878.92	737,492.39	127,375.25	2,594,718.08
19117	Lucas	Iowa	8,898	433	162.00	699.50	203,227.16	31,549.34	592,762.90	356,852.76	35,284.82	1,876,728.23	560,079.91	71,671.20	2,196,913.58
19119	Lyon	Iowa	11,581	633	187.00	1,078.50	273,892.69	38,631.22	828,237.74	549,694.62	38,576.75	3,046,775.44	823,587.32	82,262.94	3,645,396.47
19121	Madison	Iowa	15,679	773	288.00	1,272.50	326,620.19	68,655.43	966,928.37	535,612.67	50,265.33	3,076,919.84	862,232.86	131,271.20	3,609,845.37
19123	Mahaska	Iowa	22,381	1,178	434.50	1,925.50	510,800.97	118,342.32	1,201,414.05	920,720.48	103,620.35	4,359,938.14	1,431,521.45	234,984.73	5,150,286.75
19125	Marion	Iowa	33,309	1,648	539.00	2,627.00	716,917.71	140,320.72	1,844,329.65	1,215,346.04	144,431.91	4,739,438.04	1,932,263.75	307,830.11	5,867,311.42
19127	Marshall	Iowa	40,648	2,177	789.00	3,499.00	971,156.78	239,406.81	2,071,246.96	1,694,805.47	243,655.10	6,229,113.23	2,665,962.25	620,008.71	7,375,521.14
19129	Mills	Iowa	15,059	733	309.00	1,168.50	334,414.79	61,891.76	1,014,865.06	544,634.31	62,472.84	3,098,160.59	879,049.10	139,652.48	3,897,651.64
19131	Mitchell	Iowa	10,776	676	315.50	1,035.50	333,267.40	78,383.59	970,026.53	575,497.56	74,491.98	2,638,451.31	908,764.96	172,720.46	3,236,252.32
19133	Monona	Iowa	9,243	467	178.00	750.50	233,392.48	38,688.93	646,167.35	442,315.89	34,722.54	2,512,693.78	675,708.38	82,979.26	2,873,879.55
19135	Monroe	Iowa	7,970	398	145.00	657.50	182,108.33	27,437.48	657,258.29	332,742.57	36,936.36	2,293,684.85	514,850.90	68,823.86	2,542,938.95
19137	Montgomery	Iowa	10,740	513	192.50	842.00	254,043.35	37,889.47	795,234.63	500,008.81	42,611.58	2,748,686.91	754,052.16	94,248.83	3,057,503.89
19139	Muscatine	Iowa	42,745	2,552	1,005.50	4,083.00	1,051,247.22	341,309.22	2,158,954.15	1,770,343.26	317,063.97	5,877,941.09	2,821,590.48	738,387.84	7,323,256.36
19141	O'Brien	Iowa	14,398	749	244.50	1,328.00	352,480.98	60,724.36	923,421.98	649,376.60	50,954.50	3,078,890.25	1,001,857.58	112,157.94	3,532,249.34
19143	Osceola	Iowa	6,462	341	124.50	576.00	168,305.44	22,729.82	578,956.44	296,696.06	23,802.69	2,061,639.67	465,001.50	54,318.26	2,407,549.54
19145	Page	Iowa	15,932	757	298.50	1,220.00	363,467.85	72,161.39	1,011,765.91	670,974.36	66,620.35	3,142,703.11	1,034,442.21	164,345.66	3,622,353.34
19147	Palo Alto	Iowa	9,421	475	153.50	801.00	226,218.88	30,176.24	683,943.72	420,981.21	34,342.91	2,392,857.63	647,200.09	80,794.40	2,695,008.80

19149	Plymouth	Iowa	24,986	1,233	413.50	2,080.00	544,018.41	105,723.21	1,325,485.66	989,197.15	112,453.25	4,475,541.64	1,533,215.55	249,249.76	5,326,544.15
19151	Pocahontas	Iowa	7,310	368	121.00	609.50	192,815.25	27,070.42	611,039.63	300,305.59	26,586.26	1,992,699.92	493,120.83	58,167.38	2,405,326.02
19153	Polk	Iowa	430,640	21,206	8,137.50	34,730.50	8,057,805.38	2,759,062.07	13,841,899.60	14,532,644.13	4,044,971.36	33,763,082.87	22,590,449.50	7,295,845.81	45,071,118.60
19155	Pottawattamie	Iowa	93,158	4,475	1,671.50	7,406.00	1,914,673.01	562,935.78	3,699,384.41	3,259,995.29	471,387.69	10,467,425.92	5,174,668.30	1,226,129.44	12,692,045.45
19157	Poweshiek	Iowa	18,914	998	400.50	1,585.00	460,013.80	119,199.39	1,106,680.36	905,000.55	109,392.57	4,025,370.13	1,365,014.35	251,815.42	4,747,593.89
19159	Ringgold	Iowa	5,131	253	92.00	413.50	132,347.12	17,166.71	464,637.47	235,690.08	21,225.30	1,194,082.36	368,037.20	42,239.50	1,469,613.75
19161	Sac	Iowa	10,350	525	190.50	865.50	271,714.68	50,489.85	824,620.42	481,954.41	41,517.28	2,587,386.98	753,669.09	103,526.90	2,971,114.07
19163	Scott	Iowa	165,224	10,142	4,866.50	15,414.50	4,197,937.42	1,576,857.88	7,438,292.61	7,138,025.48	1,763,668.15	20,689,853.51	11,335,962.91	3,820,103.94	25,936,835.34
19165	Shelby	Iowa	12,167	591	199.50	961.00	280,888.26	55,472.30	770,288.21	487,700.36	48,365.63	2,419,103.07	768,588.62	109,318.26	2,826,368.98
19167	Sioux	Iowa	33,704	1,783	587.50	3,025.50	708,277.17	145,646.48	1,645,920.16	1,159,415.26	120,536.01	4,654,840.60	1,867,692.43	285,495.84	5,887,833.11
19169	Story	Iowa	89,542	4,411	1,711.00	7,295.50	1,624,290.00	448,757.10	3,202,139.07	2,703,884.63	424,690.73	9,249,650.51	4,328,174.63	982,638.19	11,042,017.93
19171	Tama	Iowa	17,767	998	377.50	1,607.00	461,626.08	107,350.62	1,183,949.50	802,988.12	80,686.19	3,475,552.34	1,264,614.20	205,619.64	4,189,002.26
19173	Taylor	Iowa	6,317	303	112.00	504.00	146,838.23	18,598.26	529,033.53	281,021.95	21,678.62	1,743,254.79	427,860.18	45,755.56	2,052,764.49
19175	Union	Iowa	12,534	592	177.00	987.50	279,871.60	40,271.31	830,298.54	530,583.12	34,434.14	2,972,828.32	810,454.71	85,475.83	3,467,697.83
19177	Van Buren	Iowa	7,570	398	140.00	648.50	195,910.71	20,945.98	611,805.96	310,206.92	25,445.77	1,961,259.35	506,117.63	48,901.60	2,316,938.75
19179	Wapello	Iowa	35,625	1,826	606.50	3,036.00	821,906.63	172,197.50	1,795,801.04	1,399,746.93	174,177.18	5,636,901.35	2,221,653.56	386,422.64	6,497,079.86
19181	Warren	Iowa	46,225	2,217	817.00	3,559.00	896,330.22	218,124.92	1,944,848.69	1,590,575.13	205,470.07	6,805,009.86	2,486,905.35	542,934.05	7,988,887.25
19183	Washington	Iowa	21,704	1,212	498.00	1,968.50	562,170.34	129,678.81	1,410,278.46	927,712.24	97,187.08	4,667,250.85	1,489,882.58	274,448.01	5,759,185.05
19185	Wayne	Iowa	6,403	302	94.00	507.00	147,780.79	17,449.95	476,264.24	295,984.98	18,247.78	2,235,196.91	443,765.77	40,188.20	2,451,091.01
19187	Webster	Iowa	38,013	1,908	752.50	3,057.00	872,120.43	226,457.97	1,968,831.05	1,443,507.53	216,915.81	5,215,168.34	2,315,627.96	546,296.58	6,262,449.77
19189	Winnebago	Iowa	10,866	610	249.50	971.00	297,538.01	59,968.31	807,848.82	499,471.29	53,707.59	2,625,022.84	797,009.30	118,573.93	3,317,719.67
19191	Winneshiek	Iowa	21,056	1,334	659.00	1,992.50	600,477.63	188,299.35	1,406,435.80	1,015,311.38	152,655.32	4,556,438.57	1,615,789.00	379,501.30	5,350,457.18
19193	Woodbury	Iowa	102,172	5,117	1,691.00	8,503.00	2,011,189.76	539,677.03	3,737,758.58	3,502,545.23	463,556.69	11,107,278.31	5,513,734.99	1,155,453.81	13,589,970.47
19195	Worth	Iowa	7,598	448	196.50	700.00	214,478.09	41,112.96	632,378.91	358,490.20	40,322.83	1,932,796.94	572,968.29	93,822.50	2,234,144.37
19197	Wright	Iowa	13,229	698	279.00	1,117.00	345,028.42	64,986.59	980,693.27	645,344.04	69,939.83	3,223,074.86	990,372.46	147,790.30	3,666,436.54
20001	Allen	Kansas	13,371	1,010	586.00	1,444.50	454,325.28	139,331.40	1,081,738.15	841,954.91	129,653.09	3,480,256.51	1,296,280.18	304,031.53	4,283,824.45
20003	Anderson	Kansas	8,102	574	314.00	837.00	270,008.31	71,936.20	781,561.47	467,846.56	61,408.15	2,545,406.29	737,854.86	152,197.68	2,848,764.85
20005	Atchison	Kansas	16,924	887	413.50	1,399.50	366,547.14	87,000.81	949,148.67	591,171.34	82,108.11	2,634,770.64	957,718.48	188,638.84	3,363,090.41
20007	Barber	Kansas	4,861	448	275.50	604.00	221,491.25	54,888.99	583,695.02	368,227.28	52,905.50	2,498,035.35	589,718.52	115,973.51	2,852,152.62
20009	Barton	Kansas	27,674	1,889	1,009.00	2,816.50	847,103.61	269,452.11	1,853,533.84	1,504,597.58	258,730.18	6,359,521.66	2,351,701.19	619,696.04	7,451,519.51
20011	Bourbon	Kansas	15,173	1,122	641.50	1,581.50	493,117.38	154,249.76	1,182,100.45	930,631.54	129,780.14	4,540,077.57	1,423,748.92	325,616.49	4,966,540.01
20013	Brown	Kansas	9,984	526	237.50	841.50	248,343.38	52,125.69	781,804.52	455,759.79	48,387.53	2,519,018.37	704,103.17	114,147.29	3,065,603.27
20015	Butler	Kansas	65,880	5,265	3,108.00	7,526.00	2,132,278.42	949,208.99	3,906,171.27	3,647,427.41	963,206.65	11,950,706.30	5,779,705.82	2,212,825.93	14,956,044.17
20017	Chase	Kansas	2,790	201	98.00	297.00	95,825.34	14,818.40	363,342.75	170,011.92	19,041.29	1,291,583.63	265,837.26	35,517.84	1,479,606.96
20019	Chautauqua	Kansas	3,669	342	223.00	459.50	184,843.15	42,248.37	606,057.21	310,277.41	39,882.06	2,097,861.71	495,120.56	90,531.71	2,448,949.85
20021	Cherokee	Kansas	21,603	1,872	1,205.00	2,598.00	809,055.24	329,880.70	1,712,166.73	1,390,412.70	281,737.65	5,586,452.18	2,199,467.94	690,259.20	6,627,424.10
20023	Cheyenne	Kansas	2,726	157	58.50	273.00	86,002.50	6,926.31	385,431.60	151,163.36	11,053.78	1,096,121.65	237,165.86	19,307.93	1,229,241.74
20025	Clark	Kansas	2,215	213	132.00	300.00	101,327.86	19,532.09	350,053.43	188,380.85	24,609.82	1,225,030.11	289,708.72	46,504.25	1,364,534.36
20027	Clay	Kansas	8,535	547	263.00	828.00	266,243.24	56,079.71	768,398.05	438,394.63	48,369.79	2,184,048.73	704,637.87	112,245.70	2,501,719.23
20029	Cloud	Kansas	9,533	607	280.00	933.00	295,104.55	64,272.45	807,193.39	499,225.68	49,162.70	2,799,820.73	794,330.23	132,208.41	3,270,807.72
20031	Coffey	Kansas	8,601	624	321.00	903.50	290,178.69	70,676.27	812,605.04	556,730.72	97,924.28	2,606,671.92	846,909.41	190,860.30	3,102,958.88
20033	Comanche	Kansas	1,891	179	111.50	248.50	96,218.31	15,387.91	366,399.61	172,055.09	18,028.63	1,077,183.19	268,273.40	36,186.68	1,368,644.74
20035	Cowley	Kansas	36,311	3,213	2,187.50	4,201.50	1,383,384.48	649,663.15	2,607,689.20	2,284,345.04	588,046.85	7,796,236.36	3,667,729.52	1,396,519.84	9,943,940.50
20037	Crawford	Kansas	39,134	3,028	1,862.00	4,284.00	1,234,342.66	530,812.01	2,416,694.86	2,158,279.92	431,041.81	8,357,186.86	3,392,622.57	1,118,577.59	9,878,277.25
20039	Decatur	Kansas	2,961	177	66.50	292.50	101,337.47	8,728.56	402,549.00	151,244.23	13,025.55	1,009,093.84	252,581.71	23,623.02	1,315,059.25
20041	Dickinson	Kansas	19,754	1,340	648.00	2,004.50	596,295.69	185,237.77	1,308,203.25	1,095,960.13	149,331.92	4,941,443.91	1,692,255.83	402,119.16	5,739,351.86
20043	Doniphan	Kansas	7,945	390	148.50	627.00	177,950.08	26,242.56	586,838.48	296,090.70	29,107.52	2,012,695.97	474,040.78	60,612.70	2,232,575.66
20045	Douglas	Kansas	110,826	6,299	2,873.50	9,434.00	2,256,210.26	832,302.40	4,100,337.23	3,502,240.42	755,020.83	10,801,096.29	5,758,450.68	1,858,164.17	13,681,774.53
20047	Edwards	Kansas	3,037	252	147.00	360.50	117,012.12	22,044.60	448,565.72	222,058.97	29,152.56	1,775,991.22	339,071.09	53,161.12	1,929,981.95
20049	Elk	Kansas	2,882	254	160.00	348.00	135,779.83	24,743.55	499,578.07	249,415.03	24,059.72	1,845,200.59	385,194.86	54,416.49	2,159,900.79
20051	Ellis	Kansas	28,452	1,866	904.00	2,786.00	766,580.19	251,711.94	1,733,019.44	1,321,810.98	198,084.17	5,870,111.02	2,088,391.18	509,473.41	6,653,591.75
20053	Ellsworth	Kansas	6,497	405	187.00	633.50	207,921.63	34,046.88	688,337.00	382,733.37	37,990.06	2,294,953.06	590,655.00	81,256.95	2,683,915.68
20055	Finney	Kansas	36,776	3,059	1,600.50	4,480.50	1,026,225.42	350,820.63	2,095,410.71	1,618,495.81	342,185.65	6,727,060.55	2,644,721.22	762,906.52	8,016,735.37
20057	Ford	Kansas	33,848	3,013	1,769.00	4,154.50	1,074,429.36	429,687.98	2,273,919.72	1,657,837.65	355,738.80	5,710,436.21	2,732,267.01	877,709.43	7,284,953.96
20059	Franklin	Kansas	25,992	1,659	847.00	2,501.00	710,174.75	235,323.68	1,615,087.35	1,218,881.81	200,632.36	5,380,576.97	1,929,056.56	478,547.41	6,199,281.33
20061	Geary	Kansas	34,362	2,344	1,085.50	3,495.50	747,515.00	235,638.19	1,564,021.86	1,263,853.28	294,481.52	5,008,893.04	2,011,368.29	595,477.38	6,228,557.16
20063	Gove	Kansas	2,695	194	86.50	305.00	101,051.67	13,037.45	396,215.44	185,953.46	15,010.82	1,287,553.39	287,005.13	30,899.45	1,438,697.74
20065	Graham	Kansas	2,597	164	65.00	262.00	93,611.01	7,984.25	401,525.87	156,820.02	13,326.74	1,081,171.29	250,431.04	23,132.16	1,291,707.89
20067	Grant	Kansas	7,829	691	397.00	984.00	250,354.50	67,913.12	685,951.98	377,961.65	85,380.61	1,762,893.69	628,316.14	161,564.82	2,129,300.68
20069	Gray	Kansas	6,006	522	285.50	759.50	196,026.14	47,253.27	568,437.19	309,756.15	48,277.81	1,838,001.47	505,782.29	107,919.12	2,045,692.37
20071	Greeley	Kansas	1,247	89	42.00	142.00	45,548.60	3,984.84	225,761.74	75,857.92	7,543.42	643,255.10	121,406.52	12,880.28	743,504.67
20073	Greenwood	Kansas	6,689	534	315.00	749.50	274,541.84	69,926.99	834,542.13	462,129.34	62,787.56	2,456,215.18	736,671.18	162,890.78	2,923,581.04
20075	Hamilton	Kansas	2,690	216	112.00	327.00	88,039.06	13,167.23	342,623.86	193,826.45	19,690.23	1,176,607.09	281,865.51	35,771.10	1,414,691.41
20077	Harper	Kansas	6,034	549	354.50	749.00	263,037.10	74,029.25	785,987.80	474,373.70	75,724.09	2,581,009.21	737,410.79	166,396.26	2,912,568.55
20079	Harvey	Kansas	34,684	2,550	1,503.50	3,558.00	1,128,155.61	467,740.93	2,208,818.00	2,211,678.59	407,846.63	8,781,510.18	3,339,834.20	981,838.45	9,822,859.45
20081	Haskell	Kansas	4,256	377	215.50	547.00	136,017.30	26,766.08	469,763.50	243,678.15	39,775.71	1,636,070.74	379,695.45	74,663.31	1,888,213.86

20083	Hodgeman	Kansas	1,916	155	80.50	224.50	82,764.48	10,169.61	347,488.74	131,294.17	14,352.10	870,932.83	214,058.65	26,399.49	1,192,343.76
20085	Jackson	Kansas	13,462	780	351.50	1,214.00	355,207.06	83,443.34	926,265.21	547,335.90	67,593.95	2,572,147.93	902,542.96	167,573.14	3,142,141.14
20087	Jefferson	Kansas	19,126	1,061	479.00	1,635.00	463,732.27	118,024.76	1,169,627.78	804,627.33	108,506.47	3,643,977.21	1,268,359.60	253,901.32	4,305,598.03
20089	Jewell	Kansas	3,077	169	62.50	273.00	103,381.32	9,533.71	382,541.80	179,406.13	11,389.44	1,447,138.78	282,787.45	22,267.65	1,635,037.23
20091	Johnson	Kansas	544,179	29,934	13,795.00	47,233.50	11,470,610.65	5,196,070.73	18,868,989.55	20,828,172.69	7,414,251.65	40,616,589.12	32,298,783.34	13,290,442.17	55,804,441.55
20093	Kearny	Kansas	3,977	326	168.00	484.50	127,698.62	23,558.80	467,853.20	225,503.10	29,484.81	1,324,409.49	353,201.72	57,051.01	1,583,525.73
20095	Kingman	Kansas	7,858	632	387.00	877.00	308,966.78	78,792.43	878,413.72	593,290.35	82,418.47	3,260,799.74	902,257.13	179,740.41	3,667,928.00
20097	Kiowa	Kansas	2,553	219	131.00	305.50	116,167.19	19,961.90	450,177.27	182,741.99	20,831.01	1,127,931.29	298,909.18	44,005.12	1,474,677.61
20099	Labette	Kansas	21,607	1,955	1,204.50	2,706.00	861,992.12	359,580.75	1,828,868.25	1,508,501.07	296,403.69	6,186,169.10	2,370,493.19	759,955.83	7,069,729.42
20101	Lane	Kansas	1,750	129	61.00	196.00	64,368.41	6,489.89	299,008.99	118,525.83	11,529.05	922,328.81	182,894.24	20,053.06	1,114,633.20
20103	Leavenworth	Kansas	76,227	4,055	1,851.50	6,378.00	1,559,033.02	576,112.84	3,107,959.82	2,966,440.50	708,442.06	9,113,643.83	4,525,473.52	1,430,504.88	11,017,773.69
20105	Lincoln	Kansas	3,241	197	79.00	320.50	96,241.49	11,359.78	359,553.78	170,095.47	12,777.23	1,264,220.52	266,336.96	28,264.89	1,372,803.41
20107	Linn	Kansas	9,656	623	304.00	931.50	297,125.57	65,812.47	826,384.41	548,567.39	76,889.61	2,953,250.81	845,692.96	162,239.46	3,358,598.21
20109	Logan	Kansas	2,756	193	87.50	302.50	103,015.42	12,006.85	420,244.56	236,385.74	15,102.10	1,309,688.85	339,401.15	31,294.65	1,653,726.09
20111	Lyon	Kansas	33,690	2,369	1,256.00	3,515.00	942,377.62	371,706.23	1,925,362.07	1,504,414.91	291,473.56	6,093,304.61	2,446,792.53	781,227.99	7,214,981.68
20113	McPherson	Kansas	29,180	1,942	1,017.00	2,828.00	930,520.70	313,861.73	2,061,885.24	1,740,971.09	317,297.31	6,271,204.34	2,671,491.79	760,183.45	7,717,129.45
20115	Marion	Kansas	12,660	906	493.00	1,300.50	435,352.16	148,564.41	1,059,492.57	798,249.37	101,482.52	3,894,645.31	1,233,601.53	271,737.58	4,204,091.65
20117	Marshall	Kansas	10,117	585	262.00	928.00	279,265.64	60,234.48	782,125.33	497,746.60	59,855.31	2,618,001.50	777,012.25	137,529.15	3,135,519.77
20119	Meade	Kansas	4,575	427	255.00	600.50	179,270.32	43,067.90	495,452.68	293,971.55	45,873.12	1,770,011.17	473,241.87	96,443.35	2,107,445.36
20121	Miami	Kansas	32,787	1,952	929.00	2,982.00	812,382.69	259,679.37	1,842,767.88	1,433,703.29	241,401.65	6,289,021.50	2,246,085.98	555,005.72	7,848,437.28
20123	Mitchell	Kansas	6,373	355	134.00	571.00	172,524.08	25,711.85	569,717.77	346,073.34	28,635.79	2,063,711.38	518,597.43	59,291.73	2,285,634.48
20125	Montgomery	Kansas	35,471	3,296	2,154.00	4,455.00	1,482,312.57	745,670.44	2,823,703.32	2,618,643.94	665,025.55	8,405,031.20	4,100,956.51	1,590,310.92	10,245,084.19
20127	Morris	Kansas	5,923	403	205.00	595.00	201,667.04	40,065.39	578,702.44	461,345.37	42,006.74	2,080,861.95	663,012.41	86,439.35	2,648,722.19
20129	Morton	Kansas	3,233	282	166.00	407.50	122,590.86	22,316.08	443,986.64	254,050.97	33,597.39	1,491,494.30	376,641.82	60,406.38	1,731,847.24
20131	Nemaha	Kansas	10,178	569	242.50	898.50	268,113.85	56,383.63	771,303.19	483,717.21	49,993.28	2,703,672.97	751,831.06	124,887.99	3,097,800.70
20133	Neosho	Kansas	16,512	1,367	823.00	1,921.00	635,770.12	211,075.58	1,490,478.77	1,080,699.40	189,202.36	4,307,708.90	1,716,469.51	471,490.34	5,180,066.51
20135	Ness	Kansas	3,107	234	114.00	342.50	118,237.61	20,172.06	415,233.31	227,854.41	26,745.56	1,679,379.26	346,092.02	50,580.25	1,885,366.42
20137	Norton	Kansas	5,671	332	110.00	554.00	149,928.34	19,797.86	493,696.21	278,631.60	21,375.08	1,555,048.30	428,559.95	46,264.78	1,779,836.87
20139	Osage	Kansas	16,295	1,077	564.00	1,591.50	495,426.57	152,360.41	1,253,372.12	803,318.59	96,857.19	3,529,148.60	1,298,745.17	280,625.07	4,342,882.30
20141	Osborne	Kansas	3,858	228	87.50	372.50	128,002.81	12,952.67	436,697.36	178,901.56	15,373.99	1,185,435.70	306,904.37	32,773.34	1,557,435.72
20143	Ottawa	Kansas	6,091	394	193.00	607.50	187,702.50	34,083.38	577,819.55	350,230.36	34,040.01	2,118,844.08	537,932.86	74,862.13	2,446,446.64
20145	Pawnee	Kansas	6,973	536	288.00	795.50	254,116.95	62,261.41	769,059.49	401,822.55	58,945.00	2,201,631.18	655,939.50	135,482.81	2,614,907.28
20147	Phillips	Kansas	5,642	330	128.00	549.50	165,343.78	19,456.58	539,580.67	278,469.24	23,058.47	2,070,651.42	443,813.02	50,118.19	2,272,691.11
20149	Pottawatomie	Kansas	21,604	1,345	640.50	2,081.50	527,630.28	148,301.22	1,332,602.80	851,297.26	137,209.78	3,425,221.94	1,378,927.54	321,013.64	4,465,276.34
20151	Pratt	Kansas	9,656	810	470.00	1,145.00	385,225.40	110,664.36	931,004.87	684,422.84	106,317.58	3,345,514.54	1,069,648.24	223,544.91	3,878,705.49
20153	Rawlins	Kansas	2,519	156	59.50	262.00	89,995.76	8,938.48	355,722.41	177,023.26	11,699.85	1,171,332.87	267,019.02	23,994.80	1,381,764.17
20155	Reno	Kansas	64,511	4,693	2,608.00	6,642.00	2,110,969.57	934,918.45	3,850,803.40	3,859,210.18	929,063.25	10,943,370.91	5,970,179.75	2,126,735.12	13,755,189.91
20157	Republic	Kansas	4,980	297	131.50	462.50	168,098.62	25,717.02	548,267.81	300,412.34	25,608.29	1,990,864.78	468,510.97	54,101.45	2,187,920.53
20159	Rice	Kansas	10,083	680	357.00	1,013.50	302,187.16	73,029.15	821,340.22	545,558.93	71,599.82	3,158,903.97	847,746.09	161,012.28	3,572,738.69
20161	Riley	Kansas	71,115	4,237	2,107.50	6,511.00	1,372,673.53	539,756.92	2,625,005.04	2,103,086.50	379,602.65	8,288,430.82	3,475,760.03	1,010,605.90	9,779,357.86
20163	Rooks	Kansas	5,181	317	117.50	508.50	145,357.02	21,022.02	469,876.72	234,537.19	23,462.09	1,564,458.23	379,894.21	51,686.09	1,792,056.31
20165	Rush	Kansas	3,307	238	123.50	358.00	130,399.65	21,053.90	448,651.03	228,017.63	23,090.97	1,604,294.25	358,417.28	49,321.58	1,779,637.04
20167	Russell	Kansas	6,970	443	197.00	678.00	225,945.45	34,512.26	659,272.76	437,548.39	39,224.93	2,285,480.31	663,493.84	86,221.16	2,761,757.31
20169	Saline	Kansas	55,606	3,781	1,806.00	5,726.00	1,601,300.97	631,949.36	3,166,076.37	2,794,985.41	606,888.18	9,148,126.75	4,396,286.38	1,377,706.39	10,987,136.43
20171	Scott	Kansas	4,936	371	178.50	563.00	180,556.64	33,679.46	570,225.11	296,348.83	38,225.55	1,685,876.29	476,905.47	76,932.40	1,971,623.49
20173	Sedgwick	Kansas	498,365	36,695	21,360.00	51,548.50	14,112,883.29	7,599,541.01	21,294,618.31	24,934,854.54	11,054,246.28	46,695,851.40	39,047,737.83	19,471,122.80	65,089,269.01
20175	Seward	Kansas	22,952	2,181	1,257.50	3,023.00	699,228.81	281,971.49	1,521,892.59	1,065,167.87	234,249.59	4,504,271.92	1,764,396.68	553,915.98	5,385,095.05
20177	Shawnee	Kansas	177,934	10,931	5,260.50	16,700.50	4,696,059.35	1,946,330.23	8,126,368.86	8,310,395.25	2,514,062.35	19,266,525.19	13,006,454.61	4,939,090.68	25,439,061.41
20179	Sheridan	Kansas	2,556	169	65.00	279.50	91,090.30	7,807.72	372,034.14	192,930.83	12,679.22	1,401,838.36	284,021.13	22,347.16	1,753,529.60
20181	Sherman	Kansas	6,010	383	151.50	615.50	196,771.94	29,601.28	667,444.81	298,482.77	27,151.63	1,925,455.31	495,254.71	62,157.51	2,299,304.82
20183	Smith	Kansas	3,853	213	74.50	357.00	122,570.10	13,633.38	476,806.48	198,540.78	14,446.21	1,339,110.37	321,110.87	30,430.51	1,584,815.39
20185	Stafford	Kansas	4,437	339	180.50	498.00	166,507.83	33,666.92	506,354.40	277,173.04	32,750.50	1,579,584.76	443,680.87	73,070.98	1,929,792.63
20187	Stanton	Kansas	2,235	192	101.00	284.50	73,907.21	11,058.10	272,642.45	153,641.05	20,781.44	1,166,691.03	227,548.25	32,657.37	1,315,697.84
20189	Stevens	Kansas	5,724	522	300.00	747.00	196,654.32	47,573.99	587,487.36	287,887.66	56,724.87	1,613,821.78	484,541.98	111,234.96	1,876,263.16
20191	Sumner	Kansas	24,132	2,084	1,366.50	2,826.50	897,444.15	400,023.97	1,823,452.98	1,582,313.75	307,079.14	6,055,088.94	2,479,757.91	802,640.93	7,188,380.33
20193	Thomas	Kansas	7,900	510	213.00	805.50	224,531.13	39,265.77	761,930.70	373,633.26	38,701.06	1,890,422.80	598,164.39	88,566.33	2,320,961.97
20195	Trego	Kansas	3,001	194	86.50	309.00	100,134.12	14,655.29	361,064.57	180,084.52	16,093.79	1,210,249.63	280,218.64	33,217.45	1,324,338.49
20197	Wabaunsee	Kansas	7,053	453	228.00	679.50	197,429.85	41,558.96	610,999.10	326,197.98	39,399.48	2,389,855.89	523,627.83	90,462.54	2,680,649.92
20199	Wallace	Kansas	1,485	98	40.00	163.00	49,577.47	3,966.86	240,628.30	92,807.85	6,320.57	597,971.86	142,385.33	12,120.53	766,189.15
20201	Washington	Kansas	5,799	346	158.00	530.00	182,165.75	27,754.68	595,385.19	282,478.15	26,274.96	1,561,505.29	464,643.90	60,623.00	1,965,739.07
20203	Wichita	Kansas	2,234	169	89.50	251.00	73,664.50	9,612.66	291,540.70	156,323.41	17,487.81	1,097,664.81	229,987.91	29,495.08	1,243,467.26
20205	Wilson	Kansas	9,409	802	483.50	1,129.00	386,158.39	125,599.88	982,565.90	669,775.65	113,429.99	4,032,672.46	1,055,934.04	260,331.24	4,391,310.82
20207	Woodson	Kansas	3,309	256	145.00	371.50	128,714.40	24,272.81	440,893.30	216,579.18	26,664.43	1,517,427.90	345,293.58	55,838.60	1,711,092.87
20209	Wyandotte	Kansas	157,505	8,514	3,920.00	13,543.00	3,134,744.52	1,224,747.37	5,579,821.57	5,396,988.36	1,390,309.94	15,003,606.10	8,531,732.88	2,789,565.76	19,656,574.96
21001	Adair	Kentucky	18,656	1,347	816.00	1,876.50	592,390.54	211,319.58	1,335,996.58	993,873.23	152,390.74	4,132,629.86	1,586,263.77	401,300.93	4,922,701.19
21003	Allen	Kentucky	19,956	1,405	794.00	2,044.50	605,056.07	203,580.63	1,487,241.51	979,135.41	165,475.01	4,312,094.20	1,584,191.49	416,335.85	5,122,345.60

21005	Anderson	Kentucky	21,421	1,605	994.50	2,198.00	664,296.57	235,576.37	1,518,739.07	1,071,525.08	201,170.21	4,220,899.67	1,735,821.65	497,081.69	5,484,157.22
21007	Ballard	Kentucky	8,249	492	216.00	776.00	226,625.97	51,783.96	671,487.07	421,880.17	72,541.38	2,240,216.08	648,506.14	142,697.79	2,537,069.58
21009	Barren	Kentucky	42,173	3,026	1,644.00	4,332.50	1,320,037.11	562,901.15	2,631,907.72	2,400,260.84	495,836.52	9,150,403.75	3,720,297.95	1,203,029.53	10,697,208.36
21011	Bath	Kentucky	11,591	853	497.00	1,222.00	359,552.19	111,693.96	895,319.15	595,437.37	93,668.71	3,300,052.95	954,989.56	216,294.41	4,084,011.23
21013	Bell	Kentucky	28,691	2,140	1,259.50	2,993.50	959,619.84	396,204.66	2,062,807.08	1,581,849.23	338,935.51	5,634,882.71	2,541,469.07	880,930.81	6,950,126.73
21015	Boone	Kentucky	118,811	8,833	5,460.50	12,231.00	3,156,780.37	1,636,235.28	5,327,508.66	5,268,515.53	1,830,770.25	13,523,320.50	8,425,295.91	3,908,313.65	17,260,176.57
21017	Bourbon	Kentucky	19,985	1,478	916.00	2,008.00	646,947.80	245,128.68	1,352,544.15	1,098,850.91	223,117.46	4,974,758.50	1,745,798.72	505,820.90	5,856,482.94
21019	Boyd	Kentucky	49,542	3,554	1,954.50	4,985.00	1,685,421.38	718,876.66	3,132,344.27	2,891,251.39	764,383.26	9,046,896.10	4,576,672.77	1,656,414.65	11,450,652.13
21021	Boyle	Kentucky	28,432	2,106	1,345.00	2,932.50	931,536.68	395,248.78	1,872,926.70	1,722,166.04	369,645.96	6,493,250.48	2,653,702.72	838,397.69	7,721,341.44
21023	Bracken	Kentucky	8,488	620	389.50	882.00	252,435.66	67,451.29	770,989.99	420,377.23	70,598.39	2,382,654.66	672,812.89	153,241.82	2,902,805.81
21025	Breathitt	Kentucky	13,878	1,013	582.00	1,438.50	425,476.08	129,220.23	972,343.53	808,548.96	119,523.45	3,906,202.47	1,234,025.04	260,105.20	4,613,435.61
21027	Breckinridge	Kentucky	20,059	1,424	775.00	2,120.50	628,955.94	207,778.18	1,472,880.41	1,157,362.91	156,718.81	5,123,805.74	1,786,318.85	421,995.79	6,066,359.42
21029	Bullitt	Kentucky	74,319	5,380	3,306.50	7,326.50	2,083,086.29	1,025,580.03	3,704,233.14	3,296,572.55	882,613.54	10,562,064.78	5,379,658.83	2,144,467.69	13,223,044.09
21031	Butler	Kentucky	12,690	879	472.00	1,297.00	384,447.41	108,719.51	967,205.51	675,361.10	96,824.22	3,320,507.81	1,059,808.50	230,801.56	4,039,739.24
21033	Caldwell	Kentucky	12,984	830	436.50	1,221.00	391,657.55	121,936.69	962,314.71	625,819.29	93,221.93	2,929,062.61	1,017,476.83	232,957.24	3,461,928.07
21035	Calloway	Kentucky	37,191	2,314	1,133.00	3,433.50	1,033,126.13	360,578.48	2,216,043.57	1,708,076.80	266,226.73	6,231,923.38	2,741,202.92	704,266.39	7,678,259.24
21037	Campbell	Kentucky	90,336	6,537	3,982.00	8,993.00	2,663,314.63	1,375,408.01	4,490,394.76	4,448,209.59	1,304,999.40	11,994,247.02	7,111,524.21	3,070,558.98	15,110,895.08
21039	Carlisle	Kentucky	5,104	318	147.00	488.00	160,557.22	25,553.16	530,503.64	250,751.16	23,352.20	1,661,771.67	411,308.38	51,030.29	2,089,841.88
21041	Carroll	Kentucky	10,811	808	503.00	1,109.50	324,565.82	102,269.72	851,914.88	619,850.23	124,577.85	2,959,873.85	944,416.05	253,592.01	3,496,568.59
21043	Carter	Kentucky	27,720	2,006	1,166.50	2,850.00	853,688.98	331,320.16	1,863,366.29	1,396,151.45	238,919.28	5,171,307.38	2,249,840.43	669,998.44	6,314,797.35
21045	Casey	Kentucky	15,955	1,181	736.50	1,658.50	520,221.03	177,584.29	1,148,430.90	821,239.61	129,706.70	3,591,203.06	1,341,460.63	349,307.89	4,320,213.03
21047	Christian	Kentucky	73,955	5,111	2,651.00	7,675.50	1,850,209.75	735,998.72	3,609,460.05	3,210,248.86	938,134.69	9,095,711.89	5,060,458.61	1,894,793.97	11,859,533.22
21049	Clark	Kentucky	35,613	2,624	1,607.00	3,614.50	1,135,466.89	484,128.41	2,312,968.64	1,936,974.27	436,790.98	8,305,605.05	3,072,441.15	1,015,185.73	9,958,378.59
21051	Clay	Kentucky	21,730	1,609	945.00	2,223.50	632,392.16	231,768.21	1,376,711.96	996,194.42	197,434.91	4,268,149.35	1,628,586.58	490,011.33	5,140,443.23
21053	Clinton	Kentucky	10,272	752	446.00	1,065.50	334,645.94	90,208.17	859,230.17	645,213.47	77,885.06	3,456,679.26	979,859.41	188,216.21	3,932,188.89
21055	Crittenden	Kentucky	9,315	594	292.50	883.00	274,621.89	58,378.59	807,422.50	457,171.45	49,638.15	2,522,333.10	731,793.34	119,380.61	3,020,965.37
21057	Cumberland	Kentucky	6,856	499	295.00	732.00	251,853.65	62,166.05	770,129.77	407,585.03	49,867.74	2,158,960.04	659,438.68	125,259.11	2,617,920.18
21059	Daviess	Kentucky	96,656	6,412	3,233.00	9,474.00	2,744,542.22	1,122,304.89	4,962,308.19	4,683,727.24	1,133,812.76	13,361,254.63	7,428,269.46	2,538,202.14	17,079,326.98
21061	Edmonson	Kentucky	12,161	835	476.00	1,231.00	381,167.65	103,945.25	1,033,613.39	650,929.94	86,013.39	2,932,158.20	1,032,097.59	209,464.47	3,591,069.95
21063	Elliott	Kentucky	7,852	555	328.00	804.50	227,752.82	66,032.13	660,229.09	406,645.28	52,889.00	2,219,229.61	634,398.10	127,892.77	2,501,337.18
21065	Estill	Kentucky	14,672	1,083	657.00	1,500.00	470,126.09	154,258.70	1,109,007.22	783,962.04	119,239.52	3,840,053.37	1,254,088.13	286,731.05	4,584,431.09
21067	Fayette	Kentucky	295,803	21,739	13,855.50	29,472.50	8,365,236.29	4,889,779.15	12,872,094.93	15,009,472.59	6,359,155.04	35,965,004.10	23,374,708.88	12,158,476.93	46,113,756.63
21069	Fleming	Kentucky	14,348	1,066	649.00	1,510.00	447,206.28	139,767.22	1,160,116.12	716,373.02	116,337.98	3,699,816.71	1,163,579.30	274,366.76	4,383,536.66
21071	Floyd	Kentucky	39,451	2,869	1,684.50	4,082.00	1,226,405.25	554,630.49	2,327,752.61	2,201,766.29	474,944.99	7,613,236.79	3,428,171.54	1,133,959.03	8,874,504.22
21073	Franklin	Kentucky	49,285	3,666	2,470.50	5,005.00	1,560,319.43	777,183.24	2,809,463.46	3,019,644.06	791,278.12	10,549,555.41	4,579,963.49	1,712,596.99	12,590,720.39
21075	Fulton	Kentucky	6,813	430	204.50	646.00	200,474.07	43,002.02	634,686.12	337,417.96	37,977.23	2,182,104.87	537,892.03	89,716.62	2,503,448.22
21077	Gallatin	Kentucky	8,589	633	375.00	877.00	238,730.29	63,650.42	631,901.25	413,779.96	76,628.25	2,195,584.03	652,510.25	154,596.34	2,653,610.53
21079	Garrard	Kentucky	16,912	1,270	776.00	1,775.50	548,650.42	198,093.89	1,229,701.53	971,875.80	148,127.19	4,055,078.38	1,520,526.23	377,078.51	4,690,685.93
21081	Grant	Kentucky	24,662	1,863	1,158.50	2,572.00	702,506.29	276,217.68	1,589,908.36	1,430,208.57	209,261.46	5,093,439.53	2,132,714.86	554,280.16	5,921,800.65
21083	Graves	Kentucky	37,121	2,337	1,065.00	3,604.00	1,043,291.49	346,893.21	2,102,543.27	1,814,054.95	296,190.04	6,946,803.82	2,857,346.44	710,834.26	8,837,519.66
21085	Grayson	Kentucky	25,746	1,794	999.50	2,576.50	793,519.55	284,197.34	1,727,512.27	1,230,081.56	214,947.68	5,130,097.63	2,023,601.12	578,564.05	6,331,761.25
21087	Green	Kentucky	11,258	810	435.00	1,160.50	408,555.49	100,277.52	1,075,133.23	626,261.38	74,584.60	2,980,743.64	1,034,816.87	194,253.53	3,983,772.77
21089	Greenup	Kentucky	36,910	2,674	1,511.00	3,835.00	1,220,792.87	505,954.96	2,405,986.54	2,295,954.85	513,786.15	7,297,678.32	3,516,747.73	1,152,789.84	8,661,408.34
21091	Hancock	Kentucky	8,565	596	330.50	863.00	246,331.04	56,226.30	754,631.45	481,360.87	98,398.00	2,598,215.49	727,691.91	164,055.34	2,933,652.97
21093	Hardin	Kentucky	105,543	7,641	4,302.00	10,736.00	2,891,445.20	1,405,301.75	4,867,732.61	4,978,812.62	1,555,228.94	13,296,714.19	7,870,257.83	3,202,155.75	16,800,551.68
21095	Harlan	Kentucky	29,278	2,159	1,268.00	3,088.50	961,598.57	395,415.24	2,064,595.50	1,617,978.80	355,039.69	6,079,233.10	2,579,577.37	786,521.50	7,161,782.13
21097	Harrison	Kentucky	18,846	1,387	849.00	1,935.00	602,861.04	217,109.11	1,346,371.56	1,092,681.89	196,976.57	5,276,143.04	1,695,542.93	466,509.46	6,058,596.81
21099	Hart	Kentucky	18,199	1,313	742.50	1,902.50	568,679.17	196,915.30	1,291,738.60	907,038.55	152,363.22	4,370,691.62	1,475,717.72	387,907.87	5,211,593.61
21101	Henderson	Kentucky	46,250	3,031	1,594.50	4,607.50	1,308,684.70	496,539.72	2,584,941.59	2,207,056.92	431,005.99	7,761,787.35	3,515,741.62	1,065,703.31	9,594,839.76
21103	Henry	Kentucky	15,416	1,145	684.50	1,561.00	511,299.76	172,007.36	1,369,772.98	789,553.81	138,398.46	3,864,413.75	1,300,853.57	356,483.61	4,573,448.02
21105	Hickman	Kentucky	4,902	305	136.50	473.50	145,250.11	22,632.81	501,698.76	238,820.33	24,054.99	1,514,899.36	384,070.45	50,647.83	1,791,202.29
21107	Hopkins	Kentucky	46,920	3,111	1,477.00	4,684.50	1,385,230.40	563,987.42	2,661,574.23	2,495,612.73	532,900.97	9,289,883.64	3,880,843.13	1,200,110.39	10,814,943.00
21109	Jackson	Kentucky	13,494	1,007	636.50	1,413.00	424,875.81	136,688.07	1,047,836.36	762,994.61	107,878.93	3,403,166.97	1,187,870.42	273,000.45	4,082,097.58
21111	Jefferson	Kentucky	741,096	53,536	33,920.00	75,597.50	22,270,354.73	13,190,502.67	32,158,208.79	41,355,356.67	21,550,476.61	70,888,999.35	63,625,711.40	35,562,943.66	98,871,362.18
21113	Jessamine	Kentucky	48,586	3,702	2,301.00	5,034.00	1,423,148.25	655,773.83	2,803,684.42	2,513,193.07	578,621.43	9,119,647.78	3,936,341.32	1,363,378.07	11,199,982.84
21115	Johnson	Kentucky	23,356	1,676	975.50	2,378.50	731,697.67	268,887.92	1,571,984.24	1,328,880.38	193,977.14	5,422,872.45	2,060,578.04	513,901.95	6,691,822.39
21117	Kenton	Kentucky	159,720	11,607	7,210.50	16,142.00	4,584,374.69	2,359,167.05	7,769,194.42	8,066,080.66	3,080,721.14	19,540,021.35	12,650,455.35	6,036,043.37	25,607,108.78
21119	Knott	Kentucky	16,346	1,169	644.50	1,706.00	501,329.70	162,925.02	1,174,015.51	874,859.31	175,066.37	3,679,321.93	1,376,189.01	396,738.54	4,199,031.00
21121	Knox	Kentucky	31,883	2,438	1,441.00	3,437.00	1,037,410.67	469,333.57	2,158,770.83	1,734,629.53	342,987.42	6,624,054.21	2,772,040.20	904,785.87	7,767,340.40
21123	Larue	Kentucky	14,193	1,020	615.50	1,443.00	450,746.85	131,746.75	1,099,893.01	701,226.42	108,539.30	3,463,002.89	1,151,973.28	268,513.83	4,477,553.26
21125	Laurel	Kentucky	58,849	4,450	2,754.00	6,001.50	1,811,069.14	852,555.38	3,138,626.88	3,025,474.33	733,910.41	9,534,381.34	4,836,543.47	1,720,439.50	11,570,003.66
21127	Lawrence	Kentucky	15,860	1,158	668.00	1,667.50	499,074.53	156,783.75	1,195,086.72	865,331.70	156,739.14	3,767,985.54	1,364,406.23	351,285.60	4,506,272.01
21129	Lee	Kentucky	7,887	566	329.00	808.00	250,278.87	67,350.41	718,442.81	404,065.29	58,373.41	2,464,898.18	654,344.17	139,049.08	2,841,656.95
21131	Leslie	Kentucky	11,310	810	464.00	1,138.50	347,866.28	101,154.84	901,183.98	642,743.80	105,454.15	2,784,997.41	990,610.07	225,436.11	3,468,885.82
21133	Letcher	Kentucky	24,519	1,791	1,023.00	2,569.00	809,228.68	280,632.99	1,957,147.88	1,416,512.82	276,382.26	5,313,067.72	2,225,741.50	675,037.80	6,362,802.81
21135	Lewis	Kentucky	13,870	994	550.50	1,423.50	423,527.91	132,564.39	1,044,679.05	791,058.38	103,032.04	3,575,304.46	1,214,586.28	270,299.71	4,153,509.97

21137	Lincoln	Kentucky	24,742	1,857	1,165.00	2,607.00	792,018.50	301,610.71	1,742,632.90	1,399,267.29	230,630.76	5,868,545.57	2,191,285.79	601,246.21	6,791,926.59
21139	Livingston	Kentucky	9,519	587	261.00	901.00	290,738.29	63,017.95	929,655.06	518,904.33	64,538.98	2,606,025.97	809,642.63	147,771.08	3,253,719.10
21141	Logan	Kentucky	26,835	1,832	974.00	2,617.50	798,738.32	273,706.77	1,782,977.11	1,439,567.54	257,366.05	5,937,585.71	2,238,305.86	622,332.87	7,323,879.84
21143	Lyon	Kentucky	8,314	515	255.00	769.50	285,981.06	56,825.00	797,098.71	542,019.61	49,950.06	2,556,420.95	828,000.68	116,033.14	3,096,515.24
21145	McCracken	Kentucky	65,565	3,996	1,862.00	6,095.50	1,849,427.72	669,579.56	3,440,784.31	3,212,597.91	658,639.20	9,463,052.76	5,062,025.63	1,423,867.86	12,081,819.83
21147	McCreary	Kentucky	18,306	1,346	866.00	1,848.50	545,532.15	192,472.95	1,259,814.70	1,028,904.78	175,589.37	5,591,471.06	1,574,436.93	408,379.15	6,772,241.12
21149	McLean	Kentucky	9,531	632	315.50	950.00	287,596.94	74,493.13	728,665.48	489,070.96	58,836.46	2,689,569.99	776,667.90	148,850.75	3,272,355.53
21151	Madison	Kentucky	82,916	6,100	3,859.50	8,368.50	2,354,832.35	1,215,335.54	4,069,006.16	3,906,082.61	1,036,787.69	12,353,823.83	6,260,914.96	2,460,814.81	15,301,857.73
21153	Magoffin	Kentucky	13,333	966	574.50	1,365.50	395,004.70	127,353.29	1,068,818.05	632,439.31	102,345.63	3,143,585.20	1,027,444.01	243,854.32	3,849,366.60
21155	Marion	Kentucky	19,820	1,461	838.00	2,015.50	605,419.17	208,424.04	1,437,320.61	921,402.55	179,260.62	4,190,908.66	1,526,821.72	450,333.23	5,073,329.75
21157	Marshall	Kentucky	31,448	1,988	1,007.00	2,998.50	997,460.76	327,727.84	2,253,397.92	1,861,701.03	356,163.27	6,393,267.51	2,859,161.79	798,125.88	7,864,362.20
21159	Martin	Kentucky	12,929	928	523.50	1,321.00	371,495.46	117,766.27	1,012,264.45	679,334.53	128,643.92	3,295,933.71	1,050,829.99	269,054.30	3,919,982.27
21161	Mason	Kentucky	17,490	1,281	743.00	1,778.50	565,749.81	199,036.71	1,254,157.47	911,348.70	171,509.83	4,239,326.04	1,477,098.51	424,184.45	5,287,468.77
21163	Meade	Kentucky	28,602	2,082	1,235.50	3,006.00	782,770.17	297,747.29	1,740,761.91	1,194,403.00	238,965.44	5,480,043.54	1,977,173.17	573,943.09	6,339,467.61
21165	Menifee	Kentucky	6,306	454	278.50	646.00	208,066.90	48,366.47	651,791.10	385,956.24	48,565.16	1,852,108.39	594,023.14	102,119.83	2,306,245.08
21167	Mercer	Kentucky	21,331	1,591	999.50	2,180.50	714,698.34	273,593.86	1,646,389.50	1,418,890.70	295,572.54	6,039,991.68	2,133,589.04	618,084.10	7,220,614.29
21169	Metcalfe	Kentucky	10,099	729	434.00	1,038.50	318,198.17	92,275.75	862,879.60	492,385.74	75,971.46	2,797,801.54	810,583.91	183,673.05	3,228,830.95
21171	Monroe	Kentucky	10,963	784	442.50	1,115.50	358,746.49	112,386.25	917,789.28	615,214.17	84,451.07	3,317,171.48	973,960.66	219,641.58	3,743,929.93
21173	Montgomery	Kentucky	26,499	1,957	1,162.00	2,651.50	794,091.12	328,311.98	1,596,576.35	1,226,861.23	244,247.10	4,851,467.13	2,020,952.35	645,311.71	6,101,965.09
21175	Morgan	Kentucky	13,923	991	561.50	1,436.00	422,925.83	125,809.21	1,049,389.37	669,508.48	105,846.76	3,287,904.93	1,092,434.32	277,424.50	3,759,835.32
21177	Muhlenberg	Kentucky	31,499	2,060	1,158.50	2,994.50	928,578.02	355,939.14	1,920,393.03	1,739,769.65	336,090.48	6,571,280.21	2,668,347.68	818,842.80	7,642,134.96
21179	Nelson	Kentucky	43,437	3,165	1,913.50	4,361.50	1,249,782.88	558,930.19	2,529,716.24	2,113,577.52	497,924.33	7,236,129.12	3,363,360.39	1,216,728.51	8,669,195.85
21181	Nicholas	Kentucky	7,135	531	330.00	746.50	226,341.64	59,215.29	704,962.68	378,943.59	51,010.28	2,552,637.63	605,285.23	121,496.05	2,875,478.26
21183	Ohio	Kentucky	23,842	1,631	890.50	2,437.50	698,124.52	248,664.97	1,562,515.99	1,274,322.23	195,821.04	4,843,651.26	1,972,446.75	496,760.97	5,640,420.36
21185	Oldham	Kentucky	60,316	4,429	2,802.00	6,096.00	1,630,707.73	780,569.97	3,003,740.82	2,636,106.87	715,903.52	9,307,867.33	4,266,814.60	1,660,573.06	11,337,694.47
21187	Owen	Kentucky	10,841	807	502.00	1,125.00	349,960.51	111,478.10	902,874.04	611,612.37	100,932.85	3,156,575.57	961,572.88	229,214.36	3,577,491.85
21189	Owsley	Kentucky	4,755	350	202.00	491.00	166,905.07	36,180.45	523,656.54	258,731.96	34,441.36	1,603,930.93	425,637.02	74,068.15	2,072,156.98
21191	Pendleton	Kentucky	14,877	1,074	641.50	1,510.00	446,634.12	149,666.01	1,062,568.90	767,598.66	125,701.64	3,911,115.31	1,214,232.79	305,798.55	4,647,320.21
21193	Perry	Kentucky	28,712	2,100	1,279.50	2,979.50	917,122.48	374,592.38	2,012,229.47	1,555,282.90	353,206.12	4,980,246.44	2,472,405.38	802,517.37	6,436,784.57
21195	Pike	Kentucky	65,024	4,676	2,744.50	6,673.00	2,008,444.63	979,963.16	3,544,782.77	3,517,122.02	983,914.92	11,820,667.62	5,525,566.65	2,176,831.64	13,791,176.18
21197	Powell	Kentucky	12,613	946	568.00	1,322.00	393,030.32	131,528.39	989,508.06	588,116.18	95,447.70	2,602,595.33	981,146.50	241,421.48	3,104,430.96
21199	Pulaski	Kentucky	63,063	4,662	2,896.00	6,407.50	2,129,213.31	1,055,571.17	3,817,188.18	3,651,970.42	990,984.08	11,461,444.23	5,781,183.73	2,312,632.90	14,190,302.86
21201	Robertson	Kentucky	2,282	166	102.50	233.00	77,499.38	11,384.62	306,241.78	93,971.83	13,044.74	707,918.88	171,471.21	26,651.29	877,074.33
21203	Rockcastle	Kentucky	17,056	1,263	734.00	1,743.00	558,011.68	188,791.68	1,296,602.81	899,021.44	153,727.88	3,694,418.64	1,457,033.12	387,362.14	4,482,065.42
21205	Rowan	Kentucky	23,333	1,631	935.00	2,297.50	635,173.55	237,982.91	1,346,902.92	1,029,407.16	173,818.37	4,711,757.04	1,664,580.71	452,642.93	5,730,211.75
21207	Russell	Kentucky	17,565	1,287	801.00	1,803.50	594,867.17	219,191.06	1,518,601.99	1,090,007.48	147,495.34	4,333,446.54	1,684,874.64	411,350.09	5,162,390.30
21209	Scott	Kentucky	47,173	3,565	2,270.50	4,919.00	1,262,756.87	597,998.14	2,477,189.73	2,346,371.22	698,125.90	8,448,406.66	3,609,128.09	1,449,356.36	9,934,520.78
21211	Shelby	Kentucky	42,074	3,102	1,965.50	4,282.00	1,244,511.79	558,391.56	2,445,821.18	2,062,087.87	475,697.39	7,669,071.88	3,306,599.66	1,165,021.65	9,219,842.86
21213	Simpson	Kentucky	17,327	1,205	664.50	1,730.00	503,455.13	155,239.47	1,287,142.34	914,239.00	144,290.89	4,671,849.49	1,417,694.13	319,603.76	5,418,399.43
21215	Spencer	Kentucky	17,061	1,250	752.00	1,729.00	491,848.40	166,427.24	1,212,239.23	746,523.55	122,173.65	4,347,893.17	1,238,371.95	312,011.47	4,816,143.08
21217	Taylor	Kentucky	24,512	1,781	1,029.00	2,492.50	782,261.86	304,745.13	1,681,963.63	1,298,704.78	228,783.85	5,517,905.78	2,080,966.64	613,588.83	6,498,971.28
21219	Todd	Kentucky	12,460	877	490.00	1,275.50	357,434.47	103,376.20	950,372.39	504,270.45	76,898.17	2,823,236.55	861,704.91	198,567.40	3,363,940.60
21221	Trigg	Kentucky	14,339	922	491.50	1,383.00	430,087.90	123,231.30	1,047,688.55	855,734.80	106,959.94	4,132,099.80	1,285,822.70	257,309.36	4,883,238.52
21223	Trimble	Kentucky	8,809	648	396.50	901.50	275,750.44	73,603.27	843,869.49	594,770.65	128,519.52	2,717,743.48	870,521.09	219,214.24	3,316,932.00
21225	Union	Kentucky	15,007	955	481.00	1,444.50	374,402.21	100,594.33	916,671.36	649,802.45	101,420.03	3,048,486.28	1,024,204.66	235,818.75	3,541,639.91
21227	Warren	Kentucky	113,792	7,706	4,225.00	11,169.00	2,959,795.56	1,277,543.59	4,991,709.74	5,356,679.32	1,392,003.44	15,299,391.31	8,316,474.88	3,183,559.70	18,621,118.65
21229	Washington	Kentucky	11,717	863	501.00	1,203.50	376,501.23	124,346.55	961,784.60	590,465.58	111,953.16	2,721,483.24	966,966.82	252,165.85	3,124,352.96
21231	Wayne	Kentucky	20,813	1,521	914.50	2,099.50	681,605.04	258,526.31	1,451,482.89	1,304,637.31	179,847.44	6,115,429.52	1,986,242.34	501,208.94	6,940,713.44
21233	Webster	Kentucky	13,621	882	446.00	1,316.00	386,701.14	100,529.01	1,023,474.56	694,557.18	119,607.82	3,256,450.73	1,081,258.32	247,476.04	4,112,326.83
21235	Whitley	Kentucky	35,637	2,672	1,659.00	3,762.50	1,119,653.99	490,705.37	2,114,859.66	1,972,836.74	422,417.80	7,907,649.56	3,092,490.72	1,071,434.27	9,311,107.58
21237	Wolfe	Kentucky	7,355	549	316.00	774.00	266,141.95	64,402.34	890,956.09	405,205.95	53,341.60	2,364,542.45	671,347.90	130,224.25	2,921,223.31
21239	Woodford	Kentucky	24,939	1,847	1,181.00	2,541.50	788,431.01	310,965.96	1,635,805.95	1,282,499.84	292,738.53	5,374,817.14	2,070,930.84	665,922.33	6,621,865.08
22001	Acadia	Louisiana	61,773	8,401	6,000.50	10,683.50	3,326,340.04	1,950,980.10	5,300,482.11	5,763,440.84	2,020,562.58	15,954,810.83	9,089,780.89	4,338,634.72	19,356,076.24
22003	Allen	Louisiana	25,764	3,437	2,542.00	4,349.00	1,413,888.11	737,456.44	2,561,257.70	2,324,707.66	645,780.17	7,433,185.58	3,738,595.77	1,492,814.25	9,694,032.89
22005	Ascension	Louisiana	107,215	13,972	10,813.00	16,992.00	4,919,245.94	3,114,430.19	7,391,833.78	8,482,688.30	3,795,872.61	20,488,322.49	13,401,934.24	7,733,469.54	25,325,746.30
22007	Assumption	Louisiana	23,421	3,034	2,272.00	3,838.50	1,260,335.65	634,196.60	2,500,852.61	2,289,965.85	682,490.99	7,949,410.83	3,550,301.50	1,476,125.56	9,546,400.28
22009	Avoyelles	Louisiana	42,073	5,611	4,251.50	7,103.50	2,317,406.12	1,326,880.72	4,021,093.30	3,813,181.98	1,126,172.92	11,740,272.95	6,130,588.10	2,901,484.81	15,184,910.20
22011	Beauregard	Louisiana	35,654	4,738	3,409.00	6,196.00	1,946,216.31	1,056,703.77	3,517,673.34	3,305,500.35	1,017,824.27	8,769,190.11	5,251,716.65	2,324,204.03	11,099,980.41
22013	Bienville	Louisiana	14,353	1,905	1,394.00	2,394.00	880,314.01	428,981.05	1,759,494.64	1,606,675.61	384,597.79	5,675,558.83	2,486,989.62	909,122.98	6,681,315.66
22015	Bossier	Louisiana	116,979	16,082	12,154.50	20,261.50	6,206,892.53	4,146,508.00	8,913,575.23	10,673,061.80	4,513,528.27	23,109,690.22	16,879,954.33	9,499,436.72	29,794,641.07
22017	Caddo	Louisiana	254,969	35,091	25,615.00	44,248.50	14,612,586.27	9,995,447.95	19,348,221.04	25,340,522.93	14,472,580.49	43,257,993.30	39,953,109.19	25,664,273.61	60,217,247.83
22019	Calcasieu	Louisiana	192,768	25,431	18,936.00	31,879.00	10,161,277.08	6,743,793.81	14,341,972.59	18,051,772.70	9,269,196.18	35,035,482.05	28,213,049.78	17,221,932.05	47,470,334.45
22021	Caldwell	Louisiana	10,132	1,316	991.00	1,637.00	573,575.84	220,096.37	1,389,745.47	955,343.06	189,741.13	4,405,927.34	1,528,918.90	466,871.26	4,994,519.17
22023	Cameron	Louisiana	6,839	882	616.00	1,136.50	370,148.72	124,436.75	983,858.10	773,492.33	181,919.77	3,367,585.70	1,143,641.05	321,720.17	3,945,537.05
22025	Catahoula	Louisiana	10,407	1,352	1,035.50	1,678.00	585,222.66	249,664.02	1,324,792.39	985,684.36	166,963.07	4,621,695.75	1,570,907.02	471,822.50	5,532,664.73
22027	Claiborne	Louisiana	17,195	2,203	1,661.00	2,779.50	994,359.82	488,744.21	1,909,051.06	1,766,033.70	449,593.89	5,881,568.23	2,760,393.52	1,036,552.01	7,050,316.25

22029	Concordia	Louisiana	20,822	2,742	2,074.50	3,364.00	1,177,178.68	618,379.45	2,312,017.84	2,063,278.75	503,372.16	7,487,820.98	3,240,457.42	1,258,707.75	8,869,161.80
22031	De Soto	Louisiana	26,656	3,647	2,738.00	4,567.00	1,593,608.46	849,356.11	2,983,340.23	2,818,038.91	865,202.64	8,657,304.82	4,411,647.37	1,935,272.47	10,528,143.75
22033	East Baton Rouge	Louisiana	440,171	57,318	44,897.50	70,533.50	21,975,312.75	16,484,974.79	29,203,017.75	39,461,476.92	24,332,574.86	65,671,833.01	61,436,789.68	42,309,415.43	90,813,816.20
22035	East Carroll	Louisiana	7,759	910	664.00	1,160.50	357,878.11	127,899.03	943,325.37	631,695.55	110,164.61	3,057,880.14	989,573.66	254,565.23	3,645,733.16
22037	East Feliciana	Louisiana	20,267	2,505	1,884.00	3,074.50	1,088,934.57	555,413.80	2,025,175.22	1,943,163.73	502,545.74	8,080,509.21	3,032,098.30	1,216,280.47	9,517,363.41
22039	Evangeline	Louisiana	33,984	4,667	3,487.50	5,827.50	1,830,586.73	1,027,916.23	3,010,194.83	3,178,613.10	872,003.88	11,799,292.49	5,009,199.83	2,135,718.09	13,790,892.04
22041	Franklin	Louisiana	20,767	2,673	1,973.50	3,373.50	1,158,269.81	586,853.48	2,050,378.59	2,094,397.35	455,873.24	7,568,458.63	3,252,667.17	1,145,223.04	8,916,680.39
22043	Grant	Louisiana	22,309	2,973	2,188.50	3,756.00	1,165,830.85	588,907.66	2,256,527.35	1,975,002.30	505,897.96	6,436,328.49	3,140,833.15	1,240,839.89	8,018,351.89
22045	Iberia	Louisiana	73,240	9,752	7,259.00	12,288.50	3,841,423.20	2,327,816.43	5,840,352.19	7,060,329.64	2,757,248.39	18,048,546.82	10,901,752.84	5,343,166.65	22,036,844.82
22047	Iberville	Louisiana	33,387	4,342	3,302.50	5,410.00	1,752,760.45	976,379.75	2,983,241.40	3,354,082.58	1,236,503.96	9,330,414.65	5,106,843.03	2,458,646.87	11,281,252.60
22049	Jackson	Louisiana	16,274	2,125	1,542.00	2,678.00	946,269.00	439,386.99	2,004,057.74	1,708,323.36	411,486.46	6,665,430.11	2,654,592.36	904,737.14	7,842,387.62
22051	Jefferson	Louisiana	432,552	51,674	39,249.00	64,607.00	22,016,927.00	15,649,678.39	29,027,987.70	40,804,650.07	24,085,861.42	65,790,709.15	62,821,577.07	41,258,413.99	91,490,001.32
22053	Jefferson Davis	Louisiana	31,594	4,293	3,142.50	5,423.50	1,774,267.80	949,599.98	3,150,544.11	3,062,498.38	865,062.03	10,692,078.40	4,836,766.18	2,105,170.08	12,388,428.24
22055	Lafayette	Louisiana	221,578	29,492	22,201.50	36,944.00	11,023,934.94	7,494,117.09	15,162,497.76	19,732,754.90	10,551,014.70	37,901,170.18	30,756,689.85	19,230,056.41	50,368,739.69
22057	LaFourche	Louisiana	96,318	12,028	9,174.50	15,056.00	4,757,678.89	3,098,607.93	7,055,650.92	9,024,986.44	3,931,855.11	21,291,090.03	13,782,665.33	7,456,418.95	26,532,340.04
22059	La Salle	Louisiana	14,890	1,967	1,472.00	2,443.00	850,974.40	400,637.55	1,656,958.50	1,386,018.48	353,145.98	5,323,158.13	2,236,992.88	877,181.90	6,309,592.00
22061	Lincoln	Louisiana	46,735	5,906	4,258.00	7,413.50	2,241,676.92	1,278,973.13	3,682,171.42	3,792,174.59	1,188,291.81	10,811,671.57	6,033,851.51	2,733,445.11	13,323,384.35
22063	Livingston	Louisiana	128,026	16,309	12,417.50	20,173.00	5,994,236.67	3,905,237.36	9,027,941.60	9,667,578.99	4,011,963.69	23,481,570.07	15,661,815.66	8,693,909.74	30,096,822.86
22065	Madison	Louisiana	12,093	1,438	1,077.00	1,840.50	579,313.67	237,649.79	1,371,332.69	917,089.53	179,365.86	4,287,144.13	1,496,403.19	452,804.63	5,072,889.90
22067	Morehouse	Louisiana	27,979	3,477	2,585.50	4,405.00	1,528,685.51	812,291.05	2,972,512.25	2,775,474.25	727,210.05	9,381,419.73	4,304,159.75	1,750,466.87	10,998,456.18
22069	Natchitoches	Louisiana	39,566	5,386	4,075.50	6,765.50	2,201,100.76	1,226,147.21	3,876,074.87	3,682,239.63	1,085,875.60	11,324,137.84	5,883,340.39	2,595,237.27	13,818,981.55
22071	Orleans	Louisiana	343,829	39,060	29,200.50	49,822.50	15,611,018.52	10,971,068.58	21,241,552.48	28,886,052.90	16,579,082.24	51,648,395.50	44,497,071.42	28,646,568.43	68,587,897.56
22073	Ouachita	Louisiana	153,720	20,083	15,173.00	25,122.50	7,782,360.80	5,282,391.48	11,335,593.28	13,356,907.00	6,092,364.98	27,243,089.98	21,139,267.80	12,193,140.28	36,319,678.69
22075	Plaquemines	Louisiana	23,042	2,596	1,804.50	3,361.50	989,834.20	433,470.99	2,012,904.05	1,928,032.57	608,662.87	6,739,816.87	2,917,866.77	1,156,781.54	8,006,019.26
22077	Pointe Coupee	Louisiana	22,802	3,038	2,271.00	3,777.50	1,343,898.67	692,668.39	2,609,847.07	2,323,078.94	606,295.51	7,236,626.18	3,666,977.61	1,452,672.39	9,044,728.17
22079	Rapides	Louisiana	131,613	17,861	13,477.50	22,449.50	7,317,025.39	4,842,232.50	10,527,586.53	12,891,995.95	6,214,095.88	25,598,131.09	20,209,021.33	11,726,811.41	33,563,050.34
22081	Red River	Louisiana	9,091	1,250	931.50	1,577.50	525,542.20	211,959.56	1,238,272.53	858,295.42	195,890.98	3,978,022.50	1,383,837.62	451,283.47	4,881,905.30
22083	Richland	Louisiana	20,725	2,654	1,970.00	3,334.00	1,112,490.34	560,440.47	2,195,319.82	1,831,572.36	421,801.94	6,784,710.46	2,944,062.70	1,143,350.98	8,021,723.73
22085	Sabine	Louisiana	24,233	3,310	2,457.00	4,201.50	1,481,937.62	800,987.20	2,765,588.80	2,676,942.14	686,353.75	9,275,959.82	4,158,879.76	1,723,511.44	10,996,653.46
22087	St. Bernard	Louisiana	35,897	3,892	2,722.50	5,104.50	1,447,282.11	707,290.32	2,733,489.96	2,565,349.42	757,749.75	8,219,145.91	4,012,631.53	1,630,064.38	9,992,891.94
22089	St. Charles	Louisiana	52,780	6,515	4,936.00	8,080.50	2,452,720.94	1,469,881.49	4,151,834.51	4,583,069.72	1,950,768.36	11,802,560.73	7,035,790.67	3,670,546.65	14,826,698.11
22091	St. Helena	Louisiana	11,203	1,401	1,068.00	1,744.50	603,094.65	257,172.98	1,406,687.70	962,365.26	219,554.61	4,157,561.55	1,565,459.90	531,710.92	5,087,481.17
22093	St. James	Louisiana	22,102	2,851	2,223.00	3,477.50	1,140,663.09	591,069.07	2,131,592.74	2,208,429.76	760,649.50	6,841,080.62	3,349,092.85	1,497,833.03	8,470,275.49
22095	St. John the Baptist	Louisiana	45,924	5,775	4,374.50	7,189.00	2,201,160.70	1,244,670.83	3,954,613.81	3,822,522.52	1,461,760.20	10,693,297.07	6,023,683.21	2,927,584.19	12,876,477.51
22097	St. Landry	Louisiana	83,384	11,327	8,516.50	14,397.00	4,656,029.52	2,919,833.64	7,085,849.44	7,844,769.89	3,085,652.26	18,554,906.39	12,500,799.41	6,805,425.33	23,834,066.69
22099	St. Martin	Louisiana	52,160	6,949	5,239.00	8,667.50	2,695,273.95	1,599,623.03	4,261,585.12	4,377,872.96	1,536,769.29	12,758,444.02	7,073,146.91	3,524,386.84	15,740,171.26
22101	St. Mary	Louisiana	54,650	7,121	5,221.00	9,049.00	2,855,962.68	1,678,781.65	4,588,585.27	5,327,683.34	2,011,937.29	13,369,287.76	8,183,646.02	4,075,892.57	16,593,123.73
22103	St. Tammany	Louisiana	233,740	26,944	19,118.00	34,439.50	11,095,277.28	7,261,493.53	15,826,460.36	19,478,375.46	10,130,725.41	36,803,271.79	30,573,652.73	19,028,938.74	49,226,515.77
22105	Tangipahoa	Louisiana	121,097	14,591	10,800.50	18,016.00	5,672,156.36	3,578,623.07	8,411,162.96	9,399,929.81	4,014,014.47	23,605,664.85	15,072,086.17	8,368,636.05	29,691,815.55
22107	Tensas	Louisiana	5,252	660	481.00	836.50	292,061.80	102,317.74	794,097.28	557,894.78	93,481.62	2,884,916.85	849,956.58	215,402.49	3,424,443.81
22109	Terrebonne	Louisiana	111,860	14,308	10,410.00	18,061.50	5,545,119.28	3,404,932.05	8,749,839.93	9,918,715.49	4,459,373.61	22,618,110.35	15,463,834.77	8,646,780.89	28,219,326.93
22111	Union	Louisiana	22,721	2,925	2,125.50	3,678.50	1,346,075.38	637,217.82	2,674,394.92	2,029,148.28	556,462.99	6,932,292.29	3,375,223.66	1,413,466.57	8,876,173.60
22113	Vermilion	Louisiana	57,999	7,664	5,494.50	9,890.00	3,053,373.51	1,785,903.96	4,996,267.23	5,722,944.53	1,885,447.88	15,872,588.05	8,776,318.03	3,994,682.06	19,612,191.72
22115	Vernon	Louisiana	52,334	7,300	5,406.50	9,168.50	2,537,834.39	1,485,536.63	4,208,103.71	4,367,039.68	1,679,613.14	11,446,936.08	6,904,874.07	3,414,552.88	14,566,754.54
22117	Washington	Louisiana	47,168	5,348	3,856.50	6,893.00	2,349,988.12	1,260,181.30	4,016,079.30	3,924,683.51	1,175,202.73	11,733,356.54	6,274,671.63	2,827,168.78	14,250,270.29
22119	Webster	Louisiana	41,207	5,550	4,018.50	6,846.00	2,492,421.93	1,460,941.10	4,126,320.70	4,696,726.52	1,429,130.83	13,236,608.06	7,189,148.44	3,217,872.11	15,307,958.44
22121	West Baton Rouge	Louisiana	23,788	3,144	2,441.50	3,826.00	1,201,833.22	650,703.55	2,343,542.85	2,282,429.89	654,467.68	7,427,519.97	3,484,263.12	1,398,984.59	8,820,883.87
22123	West Carroll	Louisiana	11,604	1,394	1,026.00	1,770.50	600,081.61	250,590.12	1,316,827.00	1,092,468.22	217,821.06	4,496,847.23	1,692,549.84	528,236.29	5,274,628.34
22125	West Feliciana	Louisiana	15,625	1,914	1,493.00	2,340.50	787,343.00	358,366.14	1,577,262.13	1,477,185.52	437,198.25	5,570,286.52	2,264,528.52	882,225.01	6,759,839.41
22127	Winn	Louisiana	15,313	2,019	1,515.50	2,502.00	879,883.26	398,710.54	1,816,970.99	1,530,870.62	330,310.64	6,397,386.98	2,410,753.88	824,408.78	7,296,651.35
23001	Androscoggin	Maine	107,702	5,101	963.50	9,886.00	2,184,911.76	278,058.29	4,609,149.47	3,811,233.26	311,665.57	11,530,311.33	5,996,145.02	632,348.86	15,373,343.98
23003	Aroostook	Maine	71,870	3,579	360.50	7,027.00	1,797,705.47	172,768.75	4,152,607.06	3,062,753.92	99,598.01	10,073,847.42	4,860,459.39	267,127.51	13,542,873.88
23005	Cumberland	Maine	281,674	12,968	2,657.00	23,609.50	5,742,611.77	1,070,877.22	11,160,714.90	10,812,226.28	1,508,494.34	26,728,197.23	16,554,838.05	2,711,831.30	35,879,311.26
23007	Franklin	Maine	30,768	1,466	244.50	2,756.50	675,810.04	77,662.50	1,569,861.54	1,257,017.88	62,937.68	5,421,039.68	1,932,827.92	154,625.36	6,435,326.54
23009	Hancock	Maine	54,418	2,516	210.50	5,154.50	1,262,372.28	58,041.40	3,124,730.32	2,338,425.79	58,421.07	8,125,090.08	3,600,798.07	114,563.92	10,791,972.52
23011	Kennebec	Maine	122,151	5,681	854.50	11,060.50	2,617,320.91	265,819.26	5,707,838.34	4,733,231.24	250,031.40	14,145,464.86	7,350,552.15	581,904.86	18,220,092.25
23013	Knox	Maine	39,736	1,822	182.00	3,689.50	926,840.34	42,126.03	2,356,829.01	1,782,112.34	44,440.80	7,098,336.72	2,708,952.68	92,881.59	8,771,865.92
23015	Lincoln	Maine	34,457	1,576	147.00	3,084.00	842,786.87	49,745.06	2,102,905.28	1,511,725.63	36,986.44	6,174,220.15	2,354,512.50	115,154.33	7,446,158.61
23017	Oxford	Maine	57,833	2,838	648.00	4,886.50	1,374,097.80	256,680.27	2,953,791.77	2,383,151.53	195,160.83	7,668,151.82	3,757,249.32	544,796.28	9,700,892.08
23019	Penobscot	Maine	153,923	7,258	422.50	15,487.00	3,217,147.39	180,706.43	7,403,554.03	5,637,750.81	205,795.91	17,913,764.40	8,854,898.21	490,585.07	23,235,209.93
23021	Piscataquis	Maine	17,535	837	71.00	1,749.50	446,943.36	14,289.28	1,462,667.36	795,634.10	13,462.97	4,764,932.42	1,242,577.46	31,230.98	5,297,464.26
23023	Sagadahoc	Maine	35,293	1,630	246.00	3,095.00	784,619.37	71,608.70	1,934,135.86	1,469,784.95	75,618.38	6,638,664.61	2,254,404.31	172,817.85	8,061,606.30
23025	Somerset	Maine	52,228	2,583	337.00	4,975.00	1,193,543.22	124,902.08	2,734,349.06	2,104,272.30	96,676.03	7,863,890.67	3,297,815.52	241,247.19	9,719,912.41
23027	Waldo	Maine	38,786	1,771	179.50	3,528.00	832,969.08	50,416.74	2,148,388.47	1,436,433.45	47,928.44	5,728,037.76	2,269,402.53	109,980.25	6,885,191.72
23029	Washington	Maine	32,856	1,550	123.50	3,277.50	785,521.35	38,405.52	2,048,544.91	1,426,431.20	26,302.24	5,844,115.50	2,211,952.56	77,516.91	7,707,126.36
23031	York	Maine	197,131	9,058	2,395.50	15,930.50	4,139,324.36	1,003,560.62	8,282,263.90	7,306,126.48	997,267.10	18,764,672.09	11,445,450.83	2,117,550.79	25,423,061.85

24001	Allegany	Maryland	75,087	7,057	4,700.50	9,250.00	3,325,011.69	1,863,409.51	5,335,885.33	6,174,437.70	2,050,409.46	15,711,068.44	9,499,449.40	4,264,357.61	19,465,819.14
24003	Anne Arundel	Maryland	537,656	54,763	39,760.50	69,112.50	22,259,004.89	14,981,559.16	30,000,042.27	43,308,071.22	25,753,690.16	71,211,683.31	65,567,076.12	42,489,200.17	97,240,063.97
24005	Baltimore	Maryland	805,029	81,646	61,094.50	103,084.00	35,270,519.60	25,644,540.56	45,993,007.02	68,396,358.33	43,281,962.72	103,095,878.30	103,666,877.90	70,874,214.49	143,430,733.40
24009	Calvert	Maryland	88,737	8,961	6,608.50	11,382.50	3,508,699.94	1,998,880.13	5,829,403.12	6,486,508.53	2,304,435.63	17,458,064.76	9,995,208.47	4,714,771.40	21,584,105.83
24011	Caroline	Maryland	33,066	3,347	2,292.00	4,394.50	1,374,685.18	667,678.56	2,607,957.74	2,181,389.52	626,928.08	7,035,228.14	3,556,074.71	1,429,714.76	8,702,306.59
24013	Carroll	Maryland	167,134	17,085	12,392.00	21,981.00	7,085,984.96	4,636,432.35	10,329,180.26	12,086,637.93	5,405,296.14	24,481,473.37	19,172,622.89	10,708,209.42	32,486,910.61
24015	Cecil	Maryland	101,108	10,035	7,083.50	13,032.50	4,058,867.69	2,462,719.76	6,336,104.00	7,346,930.33	2,942,271.35	16,942,708.75	11,405,798.03	5,890,099.39	21,776,644.28
24017	Charles	Maryland	146,551	15,069	11,322.50	19,175.50	5,552,429.14	3,607,026.57	8,326,708.18	9,333,680.69	4,099,204.51	20,325,270.14	14,886,109.82	8,119,569.12	26,893,744.81
24019	Dorchester	Maryland	32,618	3,289	2,348.50	4,198.50	1,551,219.63	830,652.50	2,723,428.81	2,752,488.27	717,244.99	9,118,606.61	4,303,707.90	1,761,014.90	10,950,940.04
24021	Frederick	Maryland	233,385	24,063	18,172.00	30,515.50	9,371,623.13	6,379,965.27	13,707,452.95	17,317,448.28	9,039,563.04	33,238,669.29	26,689,071.42	16,009,808.64	44,792,587.14
24023	Garrett	Maryland	30,097	2,888	1,958.00	3,812.50	1,352,856.63	652,088.91	2,500,456.21	2,288,789.98	509,037.92	7,605,078.80	3,641,646.61	1,314,793.29	9,133,214.23
24025	Harford	Maryland	244,826	24,876	18,287.50	31,731.50	10,156,260.49	6,951,162.86	14,399,514.47	18,658,454.94	9,684,967.16	35,443,641.37	28,814,715.43	17,279,625.74	48,311,426.82
24027	Howard	Maryland	287,085	29,274	21,672.50	37,180.00	11,261,979.23	7,576,044.75	15,690,693.96	21,934,249.46	11,527,053.69	41,090,537.66	33,196,228.69	19,833,086.15	54,645,246.40
24029	Kent	Maryland	20,197	2,009	1,467.50	2,564.50	1,054,723.30	485,975.21	2,000,466.67	1,917,125.58	468,920.92	6,832,450.10	2,971,848.88	1,143,188.75	8,167,803.20
24031	Montgomery	Maryland	971,777	100,088	75,178.50	123,559.00	40,667,020.67	29,314,323.81	52,154,342.41	84,593,742.37	55,583,693.23	125,399,720.30	125,260,763.10	86,840,419.73	170,256,040.50
24033	Prince Georges	Maryland	863,420	86,413	65,620.00	108,187.00	32,041,601.22	23,614,987.57	41,472,095.43	59,419,252.32	37,357,063.03	93,841,679.95	91,460,853.53	62,291,284.44	131,626,101.50
24035	Queen Annes	Maryland	47,798	4,833	3,486.50	6,245.00	2,131,340.07	1,127,478.13	3,947,213.73	3,828,194.80	1,181,180.61	12,008,245.25	5,959,534.87	2,558,034.32	14,164,289.87
24037	St. Marys	Maryland	105,151	10,609	7,649.00	13,713.50	3,985,088.19	2,439,481.10	6,440,072.64	7,750,564.03	3,472,172.54	18,890,589.86	11,735,652.22	6,290,352.03	23,182,101.83
24039	Somerset	Maryland	26,470	2,525	1,791.50	3,311.00	1,098,144.69	522,460.55	2,236,233.77	1,925,364.32	508,481.85	6,937,569.59	3,023,509.01	1,203,643.38	8,080,787.27
24041	Talbot	Maryland	37,782	3,838	2,819.00	4,915.00	2,056,984.52	1,155,214.76	3,564,775.99	3,975,404.26	1,242,681.51	10,502,228.77	6,032,388.78	2,638,967.53	12,872,324.21
24043	Washington	Maryland	147,430	14,883	10,753.50	18,803.50	6,308,962.92	4,052,819.33	9,307,490.98	11,192,961.90	4,925,902.62	22,884,595.32	17,501,924.82	9,682,915.42	30,731,699.37
24045	Wicomico	Maryland	98,733	9,724	6,844.50	12,763.50	3,997,463.09	2,343,922.04	6,628,465.68	6,925,650.69	2,777,746.82	16,000,016.21	10,923,113.79	5,657,617.20	20,838,785.01
24047	Worcester	Maryland	51,454	4,855	3,120.50	6,562.00	2,629,300.28	1,367,849.38	4,553,992.54	4,893,174.95	1,495,634.74	12,996,036.63	7,522,475.23	3,180,683.65	16,297,255.35
24510	Baltimore City	Maryland	620,961	63,321	46,701.00	79,075.00	25,210,991.67	17,576,914.48	33,316,797.85	49,694,469.18	30,276,763.98	82,899,652.64	74,905,460.84	49,962,482.75	110,628,263.60
25001	Barnstable	Massachusetts	215,888	9,459	2,552.50	16,266.00	5,327,716.35	1,170,748.20	10,021,756.93	10,551,967.72	1,884,741.49	24,948,969.07	15,879,684.07	3,493,322.41	32,269,817.02
25003	Berkshire	Massachusetts	131,219	8,714	4,845.00	12,353.50	4,208,397.08	2,179,182.08	6,692,166.25	8,008,763.51	2,897,224.39	19,687,085.44	12,217,160.59	5,505,814.73	24,933,102.00
25005	Bristol	Massachusetts	548,285	24,291	9,685.50	39,134.00	10,323,636.18	3,932,286.30	17,440,070.76	19,082,126.52	6,034,917.66	41,001,078.58	29,405,762.70	10,360,207.02	57,817,348.11
25007	Dukes	Massachusetts	16,535	724	244.00	1,209.50	343,496.12	57,484.01	951,572.67	646,777.14	71,406.42	3,290,990.41	990,273.26	138,650.48	3,657,115.86
25009	Essex	Massachusetts	743,159	33,220	11,357.50	54,972.50	14,231,658.08	4,994,818.37	24,294,815.01	27,465,432.73	7,627,836.28	55,372,084.83	41,697,090.81	13,420,007.45	77,173,287.10
25011	Franklin	Massachusetts	71,372	4,155	1,975.00	6,238.50	1,953,381.87	729,679.69	3,660,541.56	3,529,419.57	703,271.01	11,490,588.41	5,482,801.44	1,654,235.96	13,941,574.97
25013	Hampden	Massachusetts	463,490	28,440	14,669.00	41,960.50	12,076,294.84	5,675,777.36	19,062,066.52	22,263,369.71	8,528,213.54	43,510,182.52	34,339,664.55	15,401,597.78	58,693,010.45
25015	Hampshire	Massachusetts	158,080	9,117	4,428.00	13,813.00	3,945,393.97	1,689,056.80	6,729,891.61	6,747,544.28	2,039,717.61	16,101,735.61	10,692,938.25	4,183,194.26	21,187,433.66
25017	Middlesex	Massachusetts	1,503,085	69,563	28,908.00	107,919.00	28,959,559.88	11,459,145.54	46,576,684.22	61,353,862.04	22,895,413.20	105,460,930.60	90,313,421.92	34,982,646.81	148,723,238.10
25019	Nantucket	Massachusetts	10,172	442	105.00	796.50	180,105.51	21,563.95	618,040.27	332,829.93	28,509.24	2,215,347.39	512,935.44	64,296.13	2,482,174.75
25021	Norfolk	Massachusetts	670,850	30,256	11,893.50	47,835.50	13,123,919.97	4,770,015.58	21,401,078.62	26,215,059.40	8,660,928.04	49,184,895.75	39,338,979.37	13,834,770.41	68,868,106.69
25023	Plymouth	Massachusetts	494,919	21,838	8,539.00	36,192.00	9,387,401.72	3,173,375.70	16,739,411.59	17,222,066.67	4,667,076.18	35,791,974.49	26,609,468.40	8,397,580.91	49,495,940.48
25025	Suffolk	Massachusetts	722,023	31,071	12,880.00	49,638.00	11,776,417.49	4,668,672.62	20,107,700.04	25,472,060.38	8,926,550.47	49,588,592.55	37,248,477.87	13,943,491.03	66,830,922.89
25027	Worcester	Massachusetts	798,552	41,240	17,160.50	63,762.50	16,962,924.20	7,235,815.38	27,260,516.53	31,567,375.52	12,320,978.52	56,689,488.54	48,530,299.72	20,517,316.53	81,111,062.39
26001	Alcona	Michigan	10,942	812	386.50	1,206.50	518,821.43	153,911.07	1,208,835.92	1,063,587.05	108,990.79	5,103,643.34	1,582,408.48	324,725.14	5,889,947.20
26003	Alger	Michigan	9,601	678	297.50	1,070.00	353,150.69	69,405.45	957,983.63	667,490.70	67,980.67	3,425,058.94	1,020,641.39	151,391.93	3,892,557.70
26005	Allegan	Michigan	111,408	7,835	4,104.50	11,102.00	3,223,541.80	1,440,785.54	5,529,175.42	5,539,087.88	1,580,609.26	13,911,834.22	8,762,629.69	3,249,763.29	18,589,179.04
26007	Alpena	Michigan	29,598	2,196	1,058.50	3,330.00	1,080,250.04	403,609.81	2,272,933.75	2,042,023.24	375,484.23	8,050,468.51	3,122,273.28	835,912.27	9,476,903.54
26009	Antrim	Michigan	23,580	1,730	972.50	2,579.00	923,871.55	328,824.22	1,975,666.02	1,721,100.04	279,472.41	6,891,143.65	2,644,971.59	707,295.62	8,199,414.42
26011	Arenac	Michigan	15,899	1,168	663.50	1,704.00	607,635.37	191,407.70	1,405,010.83	1,150,645.39	158,613.90	5,237,410.53	1,758,280.76	402,833.41	6,050,353.09
26013	Baraga	Michigan	8,860	628	282.00	981.50	294,590.94	71,038.40	891,788.34	573,196.86	58,592.24	2,612,606.50	867,787.80	146,341.28	3,133,809.94
26015	Barry	Michigan	59,173	4,194	2,574.00	5,717.50	1,846,716.60	886,521.66	3,538,165.50	3,164,883.42	886,261.08	9,307,838.26	5,011,600.01	2,061,227.39	11,660,492.12
26017	Bay	Michigan	107,771	8,142	4,706.00	11,545.00	3,730,353.87	1,866,172.41	6,195,632.69	6,565,083.98	2,074,192.55	16,847,134.80	10,295,437.85	4,396,626.61	21,291,327.62
26019	Benzie	Michigan	17,525	1,266	706.50	1,857.50	649,905.98	207,277.42	1,448,588.69	1,172,288.26	156,447.40	5,076,896.46	1,822,194.24	415,725.53	5,872,121.14
26021	Berrien	Michigan	156,813	11,166	6,374.50	16,238.50	4,994,893.36	2,533,407.83	8,000,924.80	9,128,109.71	3,285,193.27	21,376,836.89	14,123,003.06	6,412,169.73	27,753,416.84
26023	Branch	Michigan	45,248	3,270	1,921.50	4,652.00	1,395,145.08	591,442.61	2,472,112.34	2,417,857.63	591,612.81	8,125,834.51	3,813,002.71	1,298,404.84	10,049,669.56
26025	Calhoun	Michigan	136,146	9,761	5,825.00	13,710.00	4,151,189.96	2,200,420.85	6,753,297.43	7,846,901.42	2,824,390.74	18,456,379.15	11,998,091.38	5,476,181.24	23,844,102.65
26027	Cass	Michigan	52,293	3,627	2,075.50	5,227.00	1,639,266.48	667,130.66	3,149,389.18	2,884,027.99	651,108.42	9,241,390.98	4,523,294.47	1,494,964.13	11,167,668.53
26029	Charlevoix	Michigan	25,949	1,884	876.00	2,885.50	917,240.49	291,354.79	1,933,388.09	1,806,165.05	260,237.59	7,004,885.07	2,723,405.54	610,819.31	8,468,613.48
26031	Cheboygan	Michigan	26,152	1,899	900.50	2,843.00	999,916.25	338,924.78	2,212,152.72	1,814,127.47	265,816.84	7,302,593.86	2,814,043.72	683,067.76	8,677,601.94
26033	Chippewa	Michigan	38,520	2,702	1,157.00	4,246.50	1,177,954.11	379,839.97	2,438,788.04	2,051,386.27	328,227.24	6,732,987.11	3,229,340.39	735,945.37	8,303,931.06
26035	Clare	Michigan	30,926	2,321	1,448.00	3,296.50	1,180,958.48	497,232.39	2,458,425.90	2,115,845.91	396,866.26	7,118,154.59	3,296,804.39	1,027,419.69	8,413,747.13
26037	Clinton	Michigan	75,382	5,568	3,405.00	7,801.50	2,299,695.36	1,109,167.67	4,135,227.95	3,884,612.37	1,185,240.98	10,369,271.55	6,184,307.74	2,569,999.29	13,720,894.24
26039	Crawford	Michigan	14,074	1,028	550.00	1,499.50	540,784.28	156,761.93	1,326,205.39	970,675.34	153,748.92	4,587,909.58	1,511,459.62	356,441.88	5,127,156.08
26041	Delta	Michigan	37,069	2,654	1,278.00	4,034.00	1,318,870.47	470,626.63	2,740,683.01	2,469,108.21	435,786.76	8,560,109.43	3,787,978.68	1,019,367.93	10,258,116.28
26043	Dickinson	Michigan	26,168	1,840	894.00	2,862.00	918,651.07	283,753.60	2,129,186.95	1,817,614.85	261,964.91	7,398,349.20	2,736,265.92	645,670.63	8,293,345.95
26045	Eaton	Michigan	107,759	7,645	4,749.00	10,732.00	3,309,189.47	1,651,535.57	5,720,489.07	6,112,984.87	1,807,966.66	16,525,603.35	9,422,174.35	3,762,713.05	20,976,647.15
26047	Emmet	Michigan	32,694	2,398	1,163.00	3,618.00	1,121,887.37	387,783.13	2,303,632.55	2,072,405.69	332,441.42	8,109,573.44	3,194,293.07	851,339.41	9,578,807.92
26049	Genesee	Michigan	425,790	32,326	18,843.50	45,928.50	13,443,903.05	7,479,799.16	20,044,957.33	24,488,427.21	10,917,086.96	46,857,152.80	37,932,330.26	19,160,654.19	62,799,180.02
26051	Gladwin	Michigan	25,692	1,916	1,044.00	2,773.50	1,005,718.35	374,908.07	2,101,806.28	1,863,402.84	327,812.12	6,679,314.35	2,869,121.20	825,726.48	7,892,851.63
26053	Gogebic	Michigan	16,427	1,157	524.50	1,793.50	593,941.39	160,795.12	1,358,577.01	1,102,672.67	134,802.80	5,158,336.38	1,696,614.06	315,680.78	5,677,080.07
26055	Grand Traverse	Michigan	86,986	6,291	3,415.50	9,204.00	2,767,566.88	1,211,238.96	4,879,121.87	5,029,781.58	1,405,535.22	15,520,032.37	7,797,348.47	2,955,580.67	19,218,015.02

26057	Gratiot	Michigan	42,476	3,141	1,981.50	4,334.00	1,354,018.17	603,780.89	2,665,308.45	2,345,991.76	557,423.98	8,090,540.52	3,700,009.93	1,340,451.13	9,547,742.47
26059	Hillsdale	Michigan	46,688	3,311	1,952.00	4,661.00	1,495,669.30	664,710.61	2,935,651.13	2,661,900.12	641,454.28	9,217,658.26	4,157,569.42	1,508,911.19	10,843,183.42
26061	Houghton	Michigan	36,628	2,618	1,200.00	4,243.50	1,108,016.71	329,144.00	2,283,906.93	1,900,745.00	314,387.59	6,516,405.67	3,008,761.71	735,831.95	7,750,122.31
26063	Huron	Michigan	33,118	2,453	1,296.50	3,658.00	1,272,684.68	509,954.88	2,654,715.38	2,329,401.01	490,740.08	8,020,151.66	3,602,085.69	1,101,057.60	9,826,998.58
26065	Ingham	Michigan	280,895	20,078	12,559.50	28,320.50	7,704,673.50	4,335,983.01	11,890,059.42	13,425,692.39	5,745,912.66	26,751,231.33	21,130,365.88	10,966,650.39	36,781,150.16
26067	Ionia	Michigan	63,905	4,614	2,817.00	6,424.50	1,796,580.28	854,258.56	3,057,760.60	3,105,930.34	763,145.44	9,410,708.54	4,902,510.62	1,837,177.52	11,480,902.16
26069	Iosco	Michigan	25,887	1,913	1,038.50	2,888.00	1,081,144.05	381,129.30	2,248,061.12	2,054,666.21	344,892.20	7,563,690.80	3,135,810.27	868,741.72	8,722,862.44
26071	Iron	Michigan	11,817	838	390.00	1,315.50	484,651.12	132,181.91	1,191,040.56	853,582.91	115,343.28	3,530,990.28	1,338,234.03	302,163.24	4,387,384.24
26073	Isabella	Michigan	70,311	5,007	3,088.50	6,923.50	1,847,376.75	927,755.09	3,415,444.26	2,864,480.37	706,025.56	8,869,462.50	4,711,857.12	1,764,105.80	11,319,868.19
26075	Jackson	Michigan	160,248	11,658	6,872.50	16,378.00	4,957,381.99	2,487,714.72	8,073,714.74	9,239,896.29	3,281,930.69	22,010,652.31	14,197,278.28	6,304,386.97	27,851,440.82
26077	Kalamazoo	Michigan	250,331	17,587	10,757.50	24,636.00	7,065,799.70	3,793,351.43	11,497,258.88	12,461,924.43	5,276,799.28	24,957,619.83	19,527,724.12	9,803,097.33	33,481,607.48
26079	Kalkaska	Michigan	17,153	1,277	704.00	1,843.50	608,174.73	192,537.14	1,507,354.51	1,027,371.60	195,860.78	4,329,448.49	1,635,546.32	438,917.02	5,476,256.46
26081	Kent	Michigan	602,622	43,868	26,337.00	60,331.50	16,625,853.11	9,551,690.08	24,013,404.89	29,481,002.71	14,198,062.14	55,141,074.95	46,106,855.81	23,882,322.56	74,009,221.33
26083	Keweenaw	Michigan	2,156	158	64.00	252.50	93,943.47	9,726.45	355,201.60	203,900.40	10,292.60	1,454,708.64	297,843.87	23,393.26	1,653,752.89
26085	Lake	Michigan	11,539	829	475.50	1,193.00	465,668.95	148,047.43	1,117,887.92	890,437.49	133,463.79	4,031,920.23	1,356,106.44	325,283.40	4,580,555.42
26087	Lapeer	Michigan	88,319	6,574	3,761.50	9,372.50	2,836,872.46	1,297,053.12	5,059,505.21	4,839,222.18	1,354,736.31	15,087,905.73	7,676,094.64	2,963,397.86	19,142,021.05
26089	Leelanau	Michigan	21,708	1,564	802.00	2,334.00	864,573.02	310,716.62	1,804,865.72	1,661,518.20	260,611.75	6,208,518.99	2,526,091.23	718,359.98	7,129,483.93
26091	Lenawee	Michigan	99,892	7,234	4,159.50	10,162.00	3,171,054.14	1,511,876.64	5,475,424.57	5,618,294.25	1,578,485.58	14,331,807.19	8,789,348.40	3,389,671.70	18,543,651.85
26093	Livingston	Michigan	180,967	13,409	8,488.00	18,595.50	5,468,046.74	3,021,298.65	8,551,171.33	9,890,241.88	3,417,696.36	25,530,240.96	15,358,288.62	6,859,408.71	31,315,779.59
26095	Luce	Michigan	6,631	472	209.50	728.00	228,081.14	45,793.97	662,298.72	460,975.70	47,073.87	2,625,264.19	689,056.84	102,692.17	3,028,557.93
26097	Mackinac	Michigan	11,113	799	369.00	1,207.50	429,926.99	112,363.00	1,062,305.87	781,389.77	97,359.28	3,959,134.99	1,211,316.76	248,895.77	4,669,373.61
26099	Macomb	Michigan	840,978	63,566	37,423.00	92,720.50	27,249,413.44	16,309,015.23	40,262,944.24	51,383,687.16	26,308,047.61	86,725,012.83	78,633,100.60	42,901,616.25	120,138,087.70
26101	Manistee	Michigan	24,733	1,736	922.00	2,558.00	894,530.89	349,523.51	1,819,085.86	1,700,346.41	261,198.27	6,392,612.50	2,594,877.31	668,844.03	7,750,247.01
26103	Marquette	Michigan	67,077	4,611	1,877.00	7,169.50	2,109,888.41	703,195.22	4,082,963.70	3,604,791.98	650,886.76	10,292,501.68	5,714,680.39	1,470,716.66	12,887,782.94
26105	Mason	Michigan	28,705	2,028	1,069.50	2,931.50	997,927.28	378,795.46	2,058,201.62	1,837,176.95	330,616.07	6,649,193.85	2,835,104.24	773,706.90	8,150,080.30
26107	Mecosta	Michigan	42,798	3,083	1,838.00	4,257.00	1,319,560.09	576,752.35	2,418,256.55	2,372,012.78	524,550.91	8,118,714.21	3,691,572.87	1,279,899.73	9,784,334.49
26109	Menominee	Michigan	24,029	1,703	876.00	2,582.00	828,557.75	269,674.97	1,751,960.53	1,376,895.53	214,618.96	5,016,768.08	2,205,453.27	522,244.48	6,321,466.84
26111	Midland	Michigan	83,629	6,308	3,798.50	8,615.50	2,776,472.14	1,356,748.59	5,087,015.44	5,299,734.07	1,968,824.24	14,462,871.03	8,076,206.21	3,608,924.80	17,736,316.73
26113	Missaukee	Michigan	14,849	1,128	625.00	1,587.00	514,856.89	162,476.43	1,259,715.16	918,565.63	135,881.87	4,291,267.69	1,433,422.52	326,499.46	5,242,527.11
26115	Monroe	Michigan	152,021	11,113	6,294.50	15,708.00	4,703,002.26	2,522,875.79	7,654,500.65	8,597,720.29	2,818,693.05	21,129,310.70	13,300,722.54	5,729,577.21	27,126,112.79
26117	Montcalm	Michigan	63,342	4,608	2,844.00	6,477.00	1,969,531.72	967,602.10	3,712,315.85	3,170,917.30	846,634.19	8,799,211.14	5,140,449.02	2,008,325.89	11,362,834.25
26119	Montmorency	Michigan	9,765	709	345.00	1,082.00	412,196.33	112,122.48	979,614.84	785,934.93	88,626.38	3,958,637.65	1,198,131.26	229,612.88	4,692,119.95
26121	Muskegon	Michigan	172,188	12,297	6,923.50	17,560.00	5,111,254.50	2,616,545.22	8,106,027.76	8,861,483.95	3,217,636.37	19,387,724.70	13,972,738.45	6,393,957.78	25,877,792.11
26123	Newaygo	Michigan	48,460	3,525	1,978.50	4,948.00	1,572,628.18	674,352.71	3,007,870.79	2,666,671.49	613,063.70	8,221,889.90	4,239,299.67	1,491,657.60	10,355,989.25
26125	Oakland	Michigan	1,202,362	89,182	52,383.50	128,173.00	37,530,259.94	21,960,658.78	55,173,251.87	73,042,699.53	37,088,576.76	120,757,754.80	110,572,959.50	59,889,004.42	167,152,622.60
26127	Oceana	Michigan	26,570	1,926	1,033.00	2,791.00	899,208.81	334,523.75	1,914,750.57	1,537,617.98	231,116.75	6,480,534.73	2,436,826.79	635,321.14	7,711,152.51
26129	Ogemaw	Michigan	21,699	1,612	905.00	2,341.50	841,727.57	298,967.13	1,879,866.61	1,487,140.14	224,018.20	6,167,263.12	2,328,867.71	584,402.11	7,364,046.68
26131	Ontonagon	Michigan	6,780	470	200.50	739.00	282,158.99	54,100.41	808,920.79	557,520.03	48,605.96	3,425,346.59	839,679.02	113,002.71	3,787,138.26
26133	Osceola	Michigan	23,528	1,775	984.50	2,512.00	811,963.01	304,272.01	1,820,385.35	1,560,769.53	316,289.35	6,139,185.38	2,372,732.54	687,735.91	7,634,865.96
26135	Oscoda	Michigan	8,640	636	328.50	964.00	370,258.58	86,765.07	946,803.60	581,946.38	68,785.59	2,949,257.17	952,204.96	166,441.20	3,756,598.39
26137	Otsego	Michigan	24,164	1,795	949.50	2,620.50	837,612.11	304,671.43	1,906,840.78	1,539,933.01	244,648.08	5,790,648.20	2,377,545.12	638,951.10	6,721,246.82
26139	Ottawa	Michigan	263,801	18,679	10,863.50	27,044.00	7,160,458.93	3,831,473.66	10,861,074.34	12,635,211.58	4,693,510.10	26,748,012.39	19,795,670.51	9,275,758.39	35,629,173.11
26141	Presque Isle	Michigan	13,376	982	506.00	1,452.00	587,639.56	156,531.12	1,427,425.03	993,203.69	137,193.92	4,373,546.37	1,580,843.25	344,541.45	5,189,114.39
26143	Roscommon	Michigan	24,449	1,793	969.50	2,586.50	1,111,715.42	436,499.56	2,218,555.76	2,119,979.14	388,958.53	8,223,887.24	3,231,694.56	917,577.60	9,857,830.43
26145	Saginaw	Michigan	200,169	15,046	9,011.50	21,233.50	6,545,016.93	3,539,693.79	10,207,223.64	11,914,825.18	4,596,389.80	24,456,487.59	18,459,842.11	8,893,119.63	32,561,884.07
26147	St. Clair	Michigan	163,040	12,339	6,896.00	17,555.50	5,403,982.90	2,710,648.07	8,625,276.71	9,366,250.38	3,188,941.55	21,530,601.75	14,770,233.28	6,235,447.13	28,383,525.24
26149	St. Joseph	Michigan	61,295	4,416	2,551.50	6,194.50	1,886,488.53	895,065.50	3,293,091.16	3,337,587.18	916,632.28	9,943,730.85	5,224,075.71	1,940,843.60	12,415,673.72
26151	Sanilac	Michigan	43,114	3,268	1,817.00	4,679.00	1,549,870.33	606,768.35	3,205,656.31	2,553,995.58	521,994.31	8,411,573.46	4,103,865.91	1,272,055.06	10,065,331.21
26153	Schoolcraft	Michigan	8,485	608	313.50	951.50	316,043.48	69,846.68	971,887.89	539,853.84	80,054.15	2,378,913.74	855,897.33	172,194.38	2,922,041.29
26155	Shiawassee	Michigan	70,648	5,251	3,146.50	7,405.00	2,252,648.33	1,152,757.38	3,903,303.38	3,796,375.21	1,092,909.35	11,297,068.96	6,049,023.54	2,526,795.04	13,830,408.30
26157	Tuscola	Michigan	55,729	4,252	2,613.00	5,917.50	1,927,800.64	861,949.26	3,572,903.73	3,578,582.96	847,901.14	11,606,374.83	5,506,383.60	2,000,019.23	13,732,410.86
26159	Van Buren	Michigan	76,258	5,382	3,026.00	7,546.00	2,259,593.09	1,055,105.82	3,940,052.72	4,083,028.05	1,093,144.45	12,057,713.25	6,342,621.14	2,365,365.70	15,047,115.10
26161	Washtenaw	Michigan	344,791	24,485	14,716.00	35,419.00	9,318,136.45	5,159,477.28	14,438,822.57	16,899,430.53	7,440,900.93	35,440,981.74	26,217,566.99	13,225,451.14	46,914,088.68
26163	Wayne	Michigan	1,820,584	136,479	76,836.00	194,384.00	55,153,759.76	31,443,282.77	80,753,812.07	105,724,636.10	55,148,145.63	163,585,611.90	160,878,395.80	86,970,061.56	239,383,916.70
26165	Wexford	Michigan	32,735	2,500	1,512.00	3,593.00	1,077,734.16	474,035.99	2,127,188.84	1,901,548.14	396,758.08	7,652,810.38	2,979,282.30	975,427.42	8,721,115.64
27001	Aitkin	Minnesota	16,202	1,124	509.00	1,743.00	656,654.39	184,804.39	1,455,082.85	1,258,387.48	157,275.09	6,053,573.02	1,915,041.87	429,612.29	6,906,739.81
27003	Anoka	Minnesota	330,844	22,718	12,909.50	31,997.00	8,581,067.27	4,426,335.47	13,663,999.97	14,599,902.93	5,575,120.23	30,403,767.37	23,180,970.20	10,214,083.73	41,465,653.31
27005	Becker	Minnesota	32,504	2,076	813.00	3,239.00	951,055.60	269,532.43	2,080,926.64	1,651,317.71	240,759.22	6,833,539.40	2,602,373.31	561,196.34	8,117,357.40
27007	Beltrami	Minnesota	44,442	2,976	893.00	4,970.50	1,212,990.04	292,199.48	2,496,365.35	2,028,970.86	277,676.93	7,208,528.46	3,241,960.90	642,618.91	9,021,501.26
27009	Benton	Minnesota	38,451	2,602	1,343.00	3,868.00	994,742.44	364,893.33	1,958,890.23	1,617,658.68	325,313.30	6,709,571.32	2,612,401.12	780,637.74	7,784,712.92
27011	Big Stone	Minnesota	5,269	312	99.00	526.50	166,046.26	18,808.92	524,263.57	339,259.70	20,834.05	2,295,629.28	505,305.96	44,071.84	2,578,390.97
27013	Blue Earth	Minnesota	64,013	3,693	1,771.50	5,771.00	1,442,342.33	543,659.58	2,784,883.12	2,626,062.95	490,097.20	8,553,083.20	4,068,405.27	1,181,145.17	10,663,561.20
27015	Brown	Minnesota	25,893	1,486	596.00	2,381.00	688,360.51	195,998.09	1,450,712.25	1,231,350.92	153,366.68	4,076,690.10	1,919,711.43	390,709.43	5,190,154.98
27017	Carlton	Minnesota	35,386	2,499	1,217.50	3,785.00	1,114,574.70	359,317.81	2,298,750.28	1,971,876.35	330,815.82	7,277,515.63	3,086,451.06	788,261.32	8,670,745.92
27019	Carver	Minnesota	91,042	6,077	3,330.00	9,020.00	2,113,558.18	935,527.91	3,840,660.44	3,664,489.95	1,021,562.25	11,159,184.13	5,778,048.13	2,102,687.21	14,491,857.08
27021	Cass	Minnesota	28,567	1,958	848.50	3,010.50	993,209.38	343,303.57	2,154,193.60	1,640,771.33	219,272.19	6,910,597.36	2,633,980.71	614,912.64	7,873,831.63

27023	Chippewa	Minnesota	12,441	741	298.00	1,209.50	354,909.27	77,400.30	1,041,889.05	592,649.85	64,316.66	3,028,738.74	947,559.12	156,908.24	3,795,429.69
27025	Chisago	Minnesota	53,887	3,755	1,954.50	5,422.50	1,473,284.84	615,913.73	2,925,407.98	2,362,799.20	500,300.47	7,506,397.65	3,836,084.04	1,263,650.61	9,634,624.58
27027	Clay	Minnesota	58,999	3,624	1,354.00	6,027.00	1,398,332.13	371,696.79	2,849,612.82	2,502,562.04	351,260.88	9,330,666.93	3,900,894.17	836,071.22	10,899,897.95
27029	Clearwater	Minnesota	8,695	573	215.00	942.50	252,962.54	41,865.79	759,240.24	516,762.51	40,156.26	2,749,717.96	769,725.05	87,134.31	2,984,400.18
27031	Cook	Minnesota	5,176	351	135.00	589.50	200,239.98	29,679.42	678,930.53	378,744.29	26,809.79	2,333,479.92	578,984.27	59,391.39	2,683,097.13
27033	Cottonwood	Minnesota	11,687	636	186.00	1,044.50	307,634.46	50,691.25	834,704.66	550,277.28	41,040.85	2,689,122.42	857,911.74	102,638.08	3,104,726.78
27035	Crow Wing	Minnesota	62,500	4,291	2,130.00	6,444.00	2,011,671.59	796,468.90	3,681,988.58	3,738,697.64	784,908.42	13,065,128.49	5,750,369.23	1,743,063.30	15,419,824.90
27037	Dakota	Minnesota	398,552	26,850	15,518.00	38,102.50	10,178,023.04	5,333,175.08	16,280,451.56	17,781,140.62	7,581,794.44	37,762,557.56	27,959,163.66	13,713,596.45	49,452,091.61
27039	Dodge	Minnesota	20,087	1,356	740.50	1,972.50	515,332.81	173,313.54	1,258,559.65	919,972.81	150,902.18	4,552,365.58	1,435,305.62	366,954.55	5,295,919.07
27041	Douglas	Minnesota	36,009	2,255	878.00	3,669.00	1,106,642.20	357,148.11	2,320,665.45	2,137,147.16	285,815.96	8,155,506.99	3,243,789.36	727,429.82	9,423,347.11
27043	Faribault	Minnesota	14,553	831	324.00	1,287.50	412,855.43	89,161.95	999,379.69	938,371.51	133,302.75	4,028,114.97	1,351,226.94	252,442.23	4,653,169.02
27045	Fillmore	Minnesota	20,866	1,407	751.50	2,084.00	668,168.62	212,464.49	1,480,281.55	1,157,993.87	181,590.92	4,995,400.05	1,826,162.49	424,509.71	5,779,030.03
27047	Freeborn	Minnesota	31,255	1,922	926.00	2,832.50	932,494.95	310,045.90	1,867,671.67	1,861,307.27	291,174.52	7,409,595.88	2,793,802.22	679,846.03	8,712,524.80
27049	Goodhue	Minnesota	46,183	3,121	1,858.00	4,425.50	1,422,255.30	614,176.03	2,771,904.87	2,633,187.24	559,018.64	7,739,609.37	4,055,442.54	1,296,928.86	9,792,496.53
27051	Grant	Minnesota	6,018	369	113.00	626.50	188,064.04	22,772.05	611,135.10	321,909.35	23,180.14	2,091,734.83	509,973.39	50,096.67	2,377,240.91
27053	Hennepin	Minnesota	1,152,425	76,605	43,699.00	106,747.00	30,371,978.41	17,400,907.36	44,219,013.09	59,556,325.20	28,146,449.30	98,694,585.80	89,928,303.61	48,522,105.72	137,175,141.30
27055	Houston	Minnesota	19,027	1,277	669.00	1,904.50	614,253.34	195,616.69	1,395,297.38	1,043,781.05	139,104.98	4,841,522.26	1,658,034.39	375,998.20	5,681,988.14
27057	Hubbard	Minnesota	20,428	1,328	527.50	2,112.50	663,239.69	155,475.99	1,583,963.09	1,189,297.61	126,988.80	5,499,968.87	1,852,537.30	303,772.04	6,412,666.28
27059	Isanti	Minnesota	37,816	2,666	1,486.00	3,858.50	1,072,460.48	429,998.93	2,028,077.19	1,854,729.05	342,707.51	7,393,946.59	2,927,189.52	857,105.90	8,626,232.91
27061	Itasca	Minnesota	45,058	3,131	1,290.00	4,944.50	1,550,652.97	472,255.47	3,118,882.83	2,795,469.43	542,379.34	8,837,230.69	4,346,122.41	1,213,577.78	11,081,507.34
27063	Jackson	Minnesota	10,266	546	184.50	902.00	256,691.44	43,404.40	690,426.41	545,891.51	43,575.79	3,558,458.22	802,582.94	94,030.64	3,826,493.60
27065	Kanabec	Minnesota	16,239	1,130	632.50	1,656.50	517,864.62	164,982.31	1,322,423.14	831,373.39	129,087.03	3,907,447.22	1,349,238.01	328,872.25	4,562,104.89
27067	Kandiyohi	Minnesota	42,239	2,580	1,035.50	4,067.50	1,151,355.33	352,006.97	2,500,481.83	1,933,625.86	326,556.91	6,855,742.05	3,084,981.19	813,740.63	8,580,472.92
27069	Kittson	Minnesota	4,552	287	78.50	501.00	147,995.78	11,759.58	481,299.70	259,011.74	15,346.45	1,526,741.21	407,007.51	29,431.97	1,759,144.00
27071	Koochiching	Minnesota	13,311	932	313.00	1,475.50	465,544.70	75,973.71	1,135,634.02	896,721.61	91,145.59	4,043,257.45	1,362,266.31	205,904.83	4,858,883.86
27073	Lac Qui Parle	Minnesota	7,259	409	132.50	697.50	216,896.47	30,315.54	638,947.49	395,875.80	27,841.99	2,048,024.58	612,772.26	68,061.70	2,344,314.59
27075	Lake	Minnesota	10,866	755	302.00	1,223.50	387,767.21	80,383.91	1,007,120.67	777,189.26	81,037.03	3,400,373.28	1,164,956.47	181,077.43	3,852,434.91
27077	Lake of the Woods	Minnesota	4,045	266	90.00	456.50	140,341.76	14,066.52	520,566.52	250,966.32	17,255.64	1,800,136.73	391,308.08	33,671.10	2,160,759.83
27079	Le Sueur	Minnesota	27,703	1,783	881.50	2,616.00	746,687.77	253,971.87	1,617,817.64	1,266,238.52	205,109.19	4,695,094.42	2,012,926.30	518,136.52	5,430,221.99
27081	Lincoln	Minnesota	5,896	333	104.00	546.50	171,327.69	20,438.13	584,534.32	297,897.35	21,377.28	1,763,307.72	469,225.04	44,939.38	2,205,578.61
27083	Lyon	Minnesota	25,857	1,446	454.00	2,359.50	589,527.79	108,304.63	1,487,165.61	1,088,249.64	111,986.20	5,414,679.58	1,677,777.43	242,512.71	6,118,998.00
27085	McLeod	Minnesota	36,651	2,296	1,130.50	3,446.50	976,550.17	330,623.02	2,016,654.36	1,848,322.59	339,861.97	7,431,104.44	2,824,872.76	739,569.80	8,656,897.64
27087	Mahnomen	Minnesota	5,413	358	132.50	589.00	164,258.54	22,746.45	593,378.10	290,774.05	21,993.89	1,671,332.06	455,032.59	48,471.66	1,984,951.48
27089	Marshall	Minnesota	9,439	590	165.00	1,034.00	280,902.25	34,626.62	747,552.68	528,080.12	36,967.19	2,760,976.02	808,982.37	78,514.98	3,334,609.97
27091	Martin	Minnesota	20,840	1,165	453.00	1,859.50	574,109.65	140,677.28	1,371,924.03	981,122.80	135,972.20	3,798,255.00	1,555,232.45	315,760.81	4,732,773.61
27093	Meeker	Minnesota	23,300	1,485	703.50	2,245.50	670,110.40	194,192.85	1,569,337.62	1,127,612.56	171,547.67	4,558,202.99	1,797,722.96	405,357.40	5,407,820.50
27095	Mille Lacs	Minnesota	26,097	1,810	930.00	2,645.50	767,162.92	283,479.34	1,682,822.28	1,348,815.49	206,444.66	5,804,635.03	2,115,978.41	560,517.07	6,578,458.35
27097	Morrison	Minnesota	33,198	2,258	1,097.00	3,389.50	1,005,970.68	349,696.55	2,047,851.49	1,739,554.23	289,725.62	7,071,293.91	2,745,524.91	714,940.97	8,511,592.12
27099	Mower	Minnesota	39,163	2,542	1,299.00	3,762.50	1,141,002.95	446,497.70	2,324,235.32	2,018,919.12	448,103.45	6,711,360.36	3,159,922.07	1,050,489.80	8,285,476.34
27101	Murray	Minnesota	8,725	480	179.00	799.00	241,019.40	40,717.73	693,056.46	425,858.24	35,574.33	2,852,831.34	666,877.64	83,307.97	3,183,134.15
27103	Nicollet	Minnesota	32,727	1,936	860.00	3,078.50	771,078.99	240,633.71	1,798,235.77	1,296,995.36	189,385.19	5,192,252.88	2,068,074.35	482,384.02	6,015,916.10
27105	Nobles	Minnesota	21,378	1,166	417.50	1,943.50	496,948.23	95,136.59	1,232,777.06	923,611.09	88,186.35	3,603,918.04	1,420,559.32	213,936.97	4,449,368.78
27107	Norman	Minnesota	6,852	440	145.50	746.00	215,663.42	30,334.05	700,759.47	382,825.22	29,387.24	2,253,066.45	598,488.64	67,207.98	2,717,279.70
27109	Olmsted	Minnesota	144,248	9,679	5,347.50	14,073.00	3,867,317.31	1,823,236.79	6,428,014.94	7,474,748.39	2,757,168.67	18,378,668.06	11,342,065.70	4,968,678.23	23,273,725.97
27111	Otter Tail	Minnesota	57,303	3,675	1,369.50	6,034.00	1,883,477.62	550,946.78	3,688,865.50	3,497,473.14	530,231.21	11,259,629.22	5,380,950.75	1,266,274.60	13,793,614.75
27113	Pennington	Minnesota	13,930	879	281.50	1,512.00	391,294.34	62,796.21	1,001,664.59	621,579.29	61,415.55	3,106,784.33	1,012,873.63	135,534.14	3,771,248.14
27115	Pine	Minnesota	29,750	2,041	990.50	3,133.50	929,077.56	309,161.32	2,043,246.00	1,639,041.34	240,498.54	6,581,599.72	2,568,118.90	653,400.24	7,831,257.74
27117	Pipestone	Minnesota	9,596	533	187.00	900.50	256,191.40	42,106.70	846,922.07	439,335.64	34,549.59	2,462,247.14	695,527.05	91,153.05	2,767,709.88
27119	Polk	Minnesota	31,600	1,990	593.00	3,428.00	891,259.65	198,246.66	2,147,503.98	1,510,157.94	174,807.12	5,777,945.81	2,401,417.59	422,254.65	6,918,384.50
27121	Pope	Minnesota	10,995	672	261.00	1,053.00	342,144.97	71,574.75	1,039,460.75	662,913.53	59,184.30	3,042,374.87	1,005,058.51	157,056.52	3,630,093.34
27123	Ramsey	Minnesota	508,640	35,049	20,717.50	48,657.50	14,037,532.30	7,897,466.70	20,842,251.67	26,172,315.80	12,191,018.80	46,495,791.91	40,209,848.10	21,394,993.02	65,422,925.74
27125	Red Lake	Minnesota	4,089	263	92.00	434.50	117,536.75	13,342.58	433,398.17	245,582.15	16,294.10	1,316,868.52	363,118.90	32,893.80	1,661,471.27
27127	Redwood	Minnesota	16,059	921	351.00	1,515.50	451,884.62	90,614.24	1,280,763.25	808,314.87	81,922.16	3,633,095.27	1,260,199.49	188,965.11	4,349,502.34
27129	Renville	Minnesota	15,730	945	412.00	1,537.00	471,855.79	94,226.36	1,351,576.73	946,398.51	102,988.77	4,667,155.30	1,418,254.30	221,798.00	5,315,053.32
27131	Rice	Minnesota	64,142	4,120	2,232.00	6,075.50	1,605,533.58	665,479.00	2,944,898.04	2,825,802.19	594,820.84	9,686,988.76	4,431,335.77	1,417,705.74	11,533,910.26
27133	Rock	Minnesota	9,687	531	184.50	897.50	242,552.05	32,878.04	707,920.07	437,421.00	33,035.92	2,128,043.39	679,973.05	75,884.54	2,535,304.55
27135	Roseau	Minnesota	15,629	1,026	352.50	1,755.50	440,033.52	82,357.01	1,159,910.55	824,478.45	82,768.06	3,828,959.08	1,264,511.97	178,999.39	4,776,486.06
27137	St. Louis	Minnesota	200,226	13,731	5,230.00	22,532.00	6,349,609.74	2,416,555.86	10,830,974.98	11,329,378.54	3,244,503.95	25,180,827.76	17,678,988.08	6,443,742.25	34,445,144.28
27139	Scott	Minnesota	129,928	8,797	5,009.50	12,447.00	2,898,874.12	1,391,792.42	4,853,890.71	4,532,165.59	1,431,875.52	12,574,309.54	7,431,039.71	3,076,258.61	16,228,221.58
27141	Sherburne	Minnesota	88,499	6,142	3,495.00	8,860.00	2,115,943.75	933,650.35	4,021,492.28	3,269,814.80	908,248.66	9,688,669.98	5,385,758.55	1,894,076.93	12,518,246.57
27143	Sibley	Minnesota	15,226	954	453.00	1,446.00	434,655.66	106,472.20	1,094,771.95	709,965.76	97,609.54	3,389,336.37	1,144,621.43	234,995.50	4,095,580.92
27145	Stearns	Minnesota	150,642	9,676	4,875.50	14,524.50	3,809,479.07	1,708,873.46	6,289,752.53	6,888,485.13	1,797,501.93	17,850,537.11	10,697,964.20	3,804,369.45	22,353,426.45
27147	Steele	Minnesota	36,576	2,361	1,132.00	3,471.00	991,847.71	304,155.49	2,103,842.93	1,723,762.24	314,334.96	6,095,261.94	2,715,609.95	703,503.05	7,171,818.02
27149	Stevens	Minnesota	9,726	579	215.00	945.00	256,437.30	46,183.91	766,365.39	529,177.40	50,839.69	3,807,998.63	785,614.70	103,357.11	4,229,552.69
27151	Swift	Minnesota	9,783	583	227.50	929.00	276,342.58	46,236.95	787,649.49	491,812.93	49,411.51	2,489,701.11	768,155.51	112,305.42	2,937,629.81
27153	Todd	Minnesota	24,895	1,651	740.50	2,508.00	734,152.91	224,998.19	1,664,687.43	1,361,452.26	184,148.36	5,531,278.44	2,095,605.17	445,644.24	6,742,430.38

27155	Traverse	Minnesota	3,558	210	73.50	346.00	118,855.91	12,593.34	430,606.00	271,168.97	15,951.98	1,697,617.62	390,024.88	34,356.89	1,919,916.64
27157	Wabasha	Minnesota	21,676	1,473	833.00	2,153.50	665,332.10	247,415.39	1,475,634.35	1,324,970.06	199,150.72	5,881,711.85	1,990,302.16	496,748.69	6,665,334.70
27159	Wadena	Minnesota	13,843	906	363.50	1,452.00	443,204.35	110,110.14	1,153,822.41	899,326.11	107,268.04	4,505,628.64	1,342,530.46	250,945.04	5,243,132.47
27161	Waseca	Minnesota	19,136	1,198	578.50	1,817.50	510,166.08	162,888.26	1,282,408.29	813,322.36	134,381.93	3,426,860.34	1,323,488.44	317,664.32	4,156,190.99
27163	Washington	Minnesota	238,136	16,354	9,376.50	23,058.00	6,283,093.15	3,256,791.14	10,321,283.94	10,978,205.08	3,948,074.24	26,023,316.21	17,261,298.23	7,964,828.19	33,608,126.59
27165	Watonwan	Minnesota	11,211	648	251.50	1,047.50	297,119.46	56,452.27	848,644.29	562,352.30	50,373.68	3,306,549.94	859,471.76	116,108.16	3,827,625.32
27167	Wilkin	Minnesota	6,576	396	147.50	644.00	179,722.43	25,933.94	553,553.49	361,726.73	29,147.42	1,994,651.21	541,449.16	64,309.95	2,275,887.86
27169	Winona	Minnesota	51,461	3,364	1,744.00	4,887.50	1,414,475.19	550,791.99	2,768,800.70	2,519,703.86	479,001.81	8,570,483.57	3,934,179.05	1,186,156.42	10,264,082.82
27171	Wright	Minnesota	124,700	8,509	4,633.00	12,292.00	2,978,489.42	1,348,136.09	4,973,397.20	4,583,542.02	1,286,594.09	12,398,520.72	7,562,031.44	2,869,207.75	16,167,040.94
27173	Yellow Medicine	Minnesota	10,438	595	202.50	993.00	271,778.90	41,789.57	833,152.42	451,059.67	45,580.94	2,185,304.84	722,838.57	100,029.52	2,666,740.42
28001	Adams	Mississippi	32,297	4,052	3,032.00	4,996.00	1,818,920.17	1,020,919.43	3,207,056.49	3,161,600.41	870,012.43	10,219,390.56	4,980,520.57	2,061,551.66	12,275,645.75
28003	Alcorn	Mississippi	37,057	2,727	1,445.50	4,000.00	1,185,289.86	454,020.62	2,369,186.28	2,088,574.65	405,597.26	7,407,192.35	3,273,864.51	931,662.30	9,237,359.28
28005	Amite	Mississippi	13,131	1,607	1,198.50	1,998.00	772,186.47	341,809.28	1,719,964.80	1,383,033.09	252,978.37	5,437,407.55	2,155,219.56	693,071.61	6,434,001.54
28007	Attala	Mississippi	19,564	1,787	1,141.00	2,403.50	791,417.35	323,307.85	1,651,635.94	1,414,107.96	276,388.84	5,061,239.85	2,205,525.31	658,276.59	6,241,135.90
28009	Benton	Mississippi	8,729	642	370.50	924.50	278,669.33	80,138.27	756,698.64	464,892.89	68,258.64	2,682,781.38	743,562.21	161,337.70	3,110,652.68
28011	Bolivar	Mississippi	34,145	3,516	2,411.50	4,587.00	1,384,272.17	672,664.77	2,791,768.04	2,248,833.85	559,682.71	7,899,922.41	3,633,106.02	1,402,070.67	9,750,758.32
28013	Calhoun	Mississippi	14,962	1,216	731.00	1,679.00	551,123.34	180,912.60	1,327,578.18	982,790.17	142,213.70	4,748,699.70	1,533,913.51	368,838.25	5,519,380.40
28015	Carroll	Mississippi	10,597	954	639.00	1,288.00	462,278.94	164,343.11	1,159,473.73	753,388.07	110,109.45	3,843,533.50	1,215,667.01	295,565.10	4,434,891.11
28017	Chickasaw	Mississippi	17,392	1,386	791.50	1,940.00	559,190.11	200,775.63	1,266,181.40	902,828.39	157,449.92	4,183,973.24	1,462,018.50	395,655.29	4,850,500.03
28019	Choctaw	Mississippi	8,547	717	415.50	1,006.00	323,903.60	95,721.70	924,845.86	541,912.00	85,694.92	2,622,664.97	865,815.60	195,992.50	3,264,821.83
28021	Claiborne	Mississippi	9,604	1,117	800.50	1,419.00	456,424.99	167,721.82	1,182,538.61	745,277.38	206,290.99	2,885,639.99	1,201,702.37	400,456.28	3,636,423.63
28023	Clarke	Mississippi	16,732	1,503	916.50	2,099.00	653,009.57	258,297.90	1,374,069.56	1,096,122.20	196,803.14	4,657,518.43	1,749,131.77	527,751.32	5,792,658.11
28025	Clay	Mississippi	20,634	1,645	950.50	2,296.50	702,895.00	245,174.92	1,667,182.79	1,223,553.50	213,073.94	4,723,952.41	1,926,448.50	480,371.44	5,519,523.60
28027	Coahoma	Mississippi	26,151	2,527	1,701.50	3,401.00	957,175.77	457,317.57	1,898,873.15	1,557,177.01	345,217.27	5,541,532.36	2,514,352.78	904,370.22	6,640,508.80
28029	Copiah	Mississippi	29,449	3,278	2,343.00	4,200.00	1,382,694.48	704,843.43	2,480,906.79	2,099,956.72	532,651.19	7,249,389.42	3,482,651.19	1,420,893.67	9,006,099.31
28031	Covington	Mississippi	19,568	1,955	1,292.00	2,604.50	852,082.52	343,953.07	1,678,271.15	1,414,360.41	278,611.70	6,124,823.59	2,266,442.93	692,668.45	7,229,751.15
28033	De Soto	Mississippi	161,252	12,327	7,698.50	17,076.50	4,448,406.63	2,484,730.83	7,305,043.06	7,305,872.06	2,346,116.49	18,858,003.81	11,754,278.68	5,014,767.65	23,712,955.90
28035	Forrest	Mississippi	74,934	7,312	4,947.00	9,586.00	2,753,887.59	1,498,011.43	4,489,936.36	4,818,474.98	1,457,456.39	14,602,914.77	7,572,362.57	3,342,665.16	17,344,277.60
28037	Franklin	Mississippi	8,118	992	724.50	1,251.50	446,598.68	164,246.68	1,161,199.09	756,458.60	148,144.89	3,785,501.85	1,203,057.28	346,663.19	4,300,366.88
28039	George	Mississippi	22,578	2,175	1,397.50	2,994.50	852,361.57	371,538.25	1,813,930.80	1,582,009.09	334,659.45	6,670,891.03	2,434,370.66	824,635.65	7,828,884.56
28041	Greene	Mississippi	14,400	1,283	757.00	1,782.00	494,338.92	186,191.20	1,176,179.81	791,387.42	133,139.09	3,649,625.34	1,285,726.34	354,425.72	4,134,318.25
28043	Grenada	Mississippi	21,906	1,903	1,237.50	2,675.00	812,647.98	329,807.20	1,733,754.39	1,357,551.15	257,012.51	5,861,837.95	2,170,199.14	664,130.13	6,657,771.32
28045	Hancock	Mississippi	43,929	4,678	3,315.00	6,021.00	2,074,783.52	1,072,091.58	3,822,462.22	4,009,961.33	1,293,679.19	14,085,124.01	6,084,744.85	2,542,181.38	16,244,021.53
28047	Harrison	Mississippi	187,105	19,084	12,909.00	24,959.50	7,466,573.93	4,677,952.00	10,953,785.36	13,202,992.44	5,610,065.55	27,721,951.25	20,669,566.37	11,009,946.98	35,926,100.23
28049	Hinds	Mississippi	245,285	25,979	18,941.50	33,891.50	9,801,648.05	6,544,022.02	13,942,647.34	16,839,472.96	8,228,170.20	32,271,789.30	26,641,121.02	15,305,135.74	43,283,659.74
28051	Holmes	Mississippi	19,198	1,882	1,294.50	2,528.00	751,935.71	314,677.83	1,601,750.56	1,107,324.28	229,447.83	4,180,033.63	1,859,259.99	601,315.90	5,364,338.84
28053	Humphreys	Mississippi	9,375	988	673.50	1,318.00	394,805.70	137,329.32	1,050,307.13	615,367.70	97,558.50	3,182,524.23	1,010,173.40	252,254.25	3,920,058.50
28055	Issaquena	Mississippi	1,406	150	106.00	201.00	74,943.42	10,518.53	302,077.11	100,426.31	13,474.78	772,169.82	175,369.73	25,823.50	1,073,038.55
28057	Itawamba	Mississippi	23,401	1,746	1,001.00	2,513.00	779,816.78	298,581.63	1,638,100.57	1,331,779.49	226,316.75	5,797,852.01	2,111,596.27	597,664.95	6,587,419.63
28059	Jackson	Mississippi	139,668	13,598	8,266.00	18,841.50	5,469,035.83	2,950,367.18	8,609,929.58	10,270,876.58	3,842,510.53	25,157,445.83	15,739,912.41	7,182,472.51	31,204,731.42
28061	Jasper	Mississippi	17,062	1,589	1,062.50	2,192.00	711,904.35	289,067.39	1,577,216.01	1,278,245.36	254,958.98	4,890,704.21	1,990,149.71	599,581.15	5,666,608.15
28063	Jefferson	Mississippi	7,726	927	685.00	1,150.00	388,304.66	141,581.41	947,349.94	692,202.50	113,300.59	3,444,742.04	1,080,507.16	275,630.94	3,979,474.36
28065	Jefferson Davis	Mississippi	12,487	1,266	870.50	1,644.50	564,422.21	223,422.85	1,323,938.92	1,006,813.72	211,089.48	4,670,068.87	1,571,235.93	482,956.27	5,386,243.27
28067	Jones	Mississippi	67,761	6,576	4,219.50	8,707.50	2,721,218.83	1,446,856.91	4,597,692.85	5,072,451.18	1,571,918.72	14,721,160.07	7,793,670.01	3,388,965.83	18,064,612.44
28069	Kemper	Mississippi	10,456	871	507.50	1,220.50	373,424.52	112,178.29	992,937.50	599,509.41	87,606.44	3,619,253.37	972,933.93	225,180.34	4,112,961.67
28071	Lafayette	Mississippi	47,351	3,477	2,084.00	4,835.00	1,291,186.76	569,803.75	2,426,044.89	2,203,743.28	494,657.92	8,287,285.54	3,494,930.04	1,245,788.96	9,986,002.04
28073	Lamar	Mississippi	55,658	5,692	3,857.50	7,477.50	2,081,250.41	1,127,656.87	3,510,704.44	3,231,759.30	877,155.51	10,726,948.73	5,313,009.71	2,310,326.05	13,372,019.39
28075	Lauderdale	Mississippi	80,261	6,921	4,277.00	9,834.50	2,840,128.09	1,412,487.01	4,774,381.73	5,336,547.64	1,482,027.93	15,134,241.87	8,176,675.73	3,244,767.43	18,294,131.52
28077	Lawrence	Mississippi	12,929	1,397	982.00	1,796.00	589,294.09	246,228.46	1,403,877.85	1,058,647.43	241,067.23	4,664,059.06	1,647,941.52	547,891.90	5,472,190.02
28079	Leake	Mississippi	23,805	2,226	1,430.00	3,021.50	849,041.65	382,403.85	1,767,736.71	1,440,335.72	274,642.91	6,072,073.39	2,289,377.38	709,593.29	7,135,452.76
28081	Lee	Mississippi	82,910	6,303	3,503.00	8,823.00	2,497,991.66	1,145,270.17	4,251,582.79	4,556,975.55	1,191,664.42	12,943,743.36	7,054,967.21	2,451,192.63	15,427,319.35
28083	Leflore	Mississippi	32,317	3,093	1,987.50	4,161.50	1,211,550.06	556,386.47	2,552,176.22	1,900,189.69	459,539.43	6,817,451.65	3,111,739.76	1,142,065.92	8,630,617.24
28085	Lincoln	Mississippi	34,869	4,029	2,995.50	5,171.00	1,686,039.46	937,216.06	2,989,900.53	2,770,957.90	836,337.65	8,707,594.81	4,456,997.36	1,969,548.20	10,890,666.50
28087	Lowndes	Mississippi	59,779	4,755	2,690.50	6,686.00	1,917,061.81	900,329.81	3,510,511.84	3,253,950.89	909,239.61	9,873,685.98	5,171,012.70	2,020,336.98	12,301,264.72
28089	Madison	Mississippi	95,203	9,249	6,448.00	12,166.50	3,480,494.11	1,992,698.05	5,720,618.05	5,756,471.41	2,136,181.13	14,480,430.39	9,236,965.52	4,536,999.28	18,690,322.59
28091	Marion	Mississippi	27,088	2,857	1,961.50	3,755.00	1,215,595.57	588,008.59	2,398,002.27	1,992,879.15	480,617.83	6,993,605.99	3,208,474.73	1,159,936.10	8,823,294.55
28093	Marshall	Mississippi	37,144	2,751	1,633.50	3,794.50	1,154,710.13	504,616.63	2,355,778.87	1,900,734.89	384,287.79	7,102,352.97	3,055,445.02	1,011,138.26	8,499,711.46
28095	Monroe	Mississippi	36,989	2,824	1,649.50	4,066.00	1,228,767.74	484,873.16	2,475,669.94	2,165,693.20	476,695.55	7,378,681.83	3,394,460.95	1,102,315.54	9,054,191.97
28097	Montgomery	Mississippi	10,925	957	603.00	1,352.50	455,208.40	150,731.44	1,167,973.93	778,600.02	113,380.17	3,911,267.88	1,233,808.42	287,636.35	4,524,659.57
28099	Neshoba	Mississippi	29,676	2,675	1,625.50	3,786.00	1,062,962.53	447,043.95	2,394,496.19	1,732,898.22	377,029.97	6,617,521.75	2,795,860.75	934,523.52	7,728,815.98
28101	Newton	Mississippi	21,720	1,978	1,285.50	2,761.00	823,492.49	336,598.13	1,746,555.08	1,474,705.63	276,999.13	6,225,057.27	2,298,198.12	661,669.54	7,258,737.21
28103	Noxubee	Mississippi	11,545	959	540.00	1,358.50	372,222.54	121,865.60	922,257.13	608,285.87	100,370.28	3,341,527.37	980,508.41	237,723.16	3,702,890.86
28105	Oktibbeha	Mississippi	47,671	3,701	2,263.50	5,164.50	1,310,991.42	557,171.41	2,739,730.35	2,221,270.81	462,578.07	10,285,057.72	3,532,262.23	1,159,167.40	11,485,113.57
28107	Panola	Mississippi	34,707	2,878	1,777.00	4,001.00	1,136,208.89	495,102.58	2,234,531.60	1,837,600.97	417,372.43	7,113,128.03	2,973,809.87	1,026,740.80	8,221,763.93
28109	Pearl River	Mississippi	55,834	5,974	4,203.00	7,718.50	2,577,321.87	1,448,366.86	4,600,924.80	4,325,301.89	1,279,340.34	11,988,973.34	6,902,623.76	3,058,468.04	15,064,978.51
28111	Perry	Mississippi	12,250	1,183	752.50	1,578.00	489,048.38	185,711.50	1,135,142.49	862,013.29	193,336.01	4,087,833.15	1,351,061.68	421,503.58	4,817,520.05

28113	Pike	Mississippi	40,404	4,798	3,533.50	6,106.00	1,958,279.85	1,049,647.05	3,328,504.43	3,306,706.85	898,772.13	10,940,294.54	5,264,986.70	2,221,471.42	13,097,224.38
28115	Pontotoc	Mississippi	29,957	2,320	1,368.00	3,255.00	930,676.00	395,388.99	2,073,301.30	1,567,485.69	297,241.11	5,710,270.92	2,498,161.69	759,711.78	6,958,514.10
28117	Prentiss	Mississippi	25,276	1,835	1,014.50	2,690.00	812,021.93	280,910.28	1,710,839.28	1,339,237.48	223,026.70	5,220,335.56	2,151,259.41	584,342.64	6,408,074.64
28119	Quitman	Mississippi	8,223	732	463.00	976.50	294,438.91	95,970.89	740,594.55	481,597.49	74,409.47	2,484,057.70	776,036.40	185,115.94	2,893,979.48
28121	Rankin	Mississippi	141,617	13,590	9,468.00	17,866.00	5,372,996.14	3,287,656.83	8,562,630.51	8,648,864.27	3,617,397.61	18,835,686.48	14,021,860.41	7,427,290.10	25,490,944.30
28123	Scott	Mississippi	28,264	2,657	1,761.00	3,563.50	1,048,692.21	482,688.23	2,189,498.79	1,622,634.46	357,169.21	6,131,588.76	2,671,326.68	929,294.36	7,558,808.87
28125	Sharkey	Mississippi	4,916	531	378.50	690.50	209,244.15	65,600.81	530,498.95	357,572.06	57,029.95	2,090,944.40	566,816.21	132,426.18	2,261,341.66
28127	Simpson	Mississippi	27,503	2,793	1,973.50	3,662.50	1,157,306.03	566,401.26	2,205,423.69	1,901,134.51	408,243.47	7,440,530.98	3,058,440.54	1,073,921.15	8,853,711.61
28129	Smith	Mississippi	16,491	1,592	1,065.50	2,151.00	704,265.50	282,003.65	1,713,327.46	1,188,751.97	234,502.48	4,899,340.43	1,893,017.47	569,487.87	5,960,613.61
28131	Stone	Mississippi	17,786	1,759	1,209.50	2,377.50	688,752.92	287,224.15	1,528,752.44	1,136,409.77	233,471.05	5,137,404.11	1,825,162.69	574,245.93	6,075,912.32
28133	Sunflower	Mississippi	29,450	2,900	1,996.00	3,823.00	1,095,957.87	500,670.33	2,219,731.85	1,587,362.69	361,059.80	5,649,254.40	2,683,320.55	1,006,001.98	6,904,201.97
28135	Tallahatchie	Mississippi	15,378	1,357	902.50	1,831.50	518,078.13	203,864.10	1,179,425.34	890,588.50	146,415.32	4,358,007.50	1,408,666.63	371,052.39	4,977,816.09
28137	Tate	Mississippi	28,886	2,233	1,300.00	3,116.50	893,431.56	340,666.56	1,986,999.78	1,449,739.51	286,434.51	6,328,999.03	2,343,171.07	743,718.88	7,561,356.29
28139	Tippah	Mississippi	22,232	1,646	950.00	2,368.00	682,812.93	235,956.99	1,531,690.58	1,212,971.77	200,385.56	5,076,893.17	1,895,784.69	505,280.00	5,899,568.75
28141	Tishomingo	Mississippi	19,593	1,407	789.00	2,046.50	625,412.72	230,552.22	1,394,864.89	1,075,526.13	169,928.45	4,797,938.51	1,700,938.85	457,407.05	5,597,737.02
28143	Tunica	Mississippi	10,778	953	609.00	1,290.00	338,119.36	106,603.95	902,771.69	551,804.77	101,312.73	3,164,040.82	889,924.14	224,840.83	3,725,015.46
28145	Union	Mississippi	27,134	2,046	1,202.50	2,900.00	857,104.29	315,033.16	1,733,690.94	1,465,785.62	254,771.46	5,920,856.46	2,322,889.91	646,114.10	6,906,642.67
28147	Walthall	Mississippi	15,443	1,753	1,280.00	2,227.50	773,963.79	324,424.04	1,814,106.03	1,202,654.27	260,719.07	4,930,335.66	1,976,618.06	654,781.14	6,041,353.45
28149	Warren	Mississippi	48,773	5,512	3,961.50	7,107.00	2,236,965.66	1,271,333.19	3,862,513.98	4,062,172.97	1,305,946.78	12,572,237.85	6,299,138.63	2,850,837.20	15,019,497.56
28151	Washington	Mississippi	51,137	5,625	4,091.50	7,183.50	2,233,239.02	1,298,827.41	3,869,273.38	3,866,047.24	1,101,015.98	12,039,720.93	6,099,286.27	2,680,567.54	14,769,222.26
28153	Wayne	Mississippi	20,747	1,951	1,239.00	2,647.50	795,234.92	316,705.38	1,739,066.33	1,402,446.63	271,799.86	6,210,952.85	2,197,681.56	659,973.55	7,537,707.83
28155	Webster	Mississippi	10,253	848	510.50	1,176.50	377,957.07	117,996.23	987,160.53	578,811.66	94,798.62	2,864,893.26	956,768.73	232,884.30	3,383,607.57
28157	Wilkinson	Mississippi	9,878	1,259	926.50	1,565.50	521,463.92	223,670.85	1,220,374.60	861,841.69	169,582.98	3,786,130.16	1,383,305.62	427,427.55	4,574,944.21
28159	Winston	Mississippi	19,198	1,664	966.50	2,314.50	735,118.40	282,955.31	1,536,300.49	1,213,672.10	220,390.94	4,946,657.91	1,948,790.50	562,789.53	5,718,136.38
28161	Yalobusha	Mississippi	12,678	1,041	598.50	1,438.00	496,367.08	163,768.91	1,269,886.38	863,576.30	134,201.28	3,915,111.56	1,359,943.38	350,065.13	4,690,068.55
28163	Yazoo	Mississippi	28,065	2,837	1,989.50	3,703.00	1,116,813.89	521,859.88	2,156,724.24	1,923,778.94	452,628.46	6,983,264.63	3,040,592.83	1,120,735.31	8,273,347.18
29001	Adair	Missouri	25,607	1,177	381.00	1,943.50	466,479.30	90,382.99	1,194,209.62	744,823.51	64,812.71	3,816,724.65	1,211,302.80	172,522.69	4,408,861.57
29003	Andrew	Missouri	17,291	849	355.00	1,344.00	381,124.95	83,533.70	1,097,021.88	621,373.97	63,195.20	3,109,568.04	1,002,498.91	161,210.66	3,617,869.59
29005	Atchison	Missouri	5,685	270	107.50	451.00	138,919.07	15,201.41	477,050.65	218,967.22	19,932.70	1,450,093.52	357,886.29	37,024.55	1,832,376.02
29007	Audrain	Missouri	25,529	1,308	427.00	2,121.50	551,126.73	113,163.90	1,332,951.35	935,524.23	93,296.02	4,204,816.81	1,486,650.95	243,596.11	4,822,955.26
29009	Barry	Missouri	35,597	3,079	1,950.00	4,157.00	1,394,174.42	665,421.45	2,725,237.67	2,537,330.41	569,734.46	7,951,703.99	3,931,504.83	1,423,945.10	9,628,319.57
29011	Barton	Missouri	12,402	906	516.00	1,308.50	416,003.07	117,589.66	1,175,160.77	629,226.14	97,055.69	2,990,047.30	1,045,229.21	232,075.43	3,781,855.85
29013	Bates	Missouri	17,049	1,025	498.50	1,560.00	464,590.67	119,305.73	1,130,373.35	784,566.17	98,068.85	3,816,386.24	1,249,156.84	251,962.93	4,526,435.24
29015	Benton	Missouri	19,056	1,024	452.00	1,603.50	599,416.93	158,247.05	1,499,812.67	1,127,883.16	125,650.10	5,580,653.57	1,727,300.09	312,842.23	6,548,313.22
29017	Bollinger	Missouri	12,363	724	331.50	1,117.00	331,224.21	79,173.46	959,966.85	517,715.45	85,339.03	2,166,361.98	848,939.66	189,413.71	2,821,870.33
29019	Boone	Missouri	162,642	8,006	3,067.00	13,396.50	2,904,133.40	937,614.64	5,486,401.87	4,862,200.59	968,235.80	13,888,166.34	7,766,333.98	2,038,307.48	18,049,306.81
29021	Buchanan	Missouri	89,201	4,383	1,840.00	6,894.50	1,809,403.25	604,107.36	3,447,648.34	3,256,117.53	589,326.64	11,174,133.55	5,065,520.78	1,351,643.05	13,852,727.25
29023	Butler	Missouri	42,794	2,626	1,313.50	3,982.00	1,167,235.55	430,047.73	2,278,568.56	2,194,876.76	371,844.18	7,804,254.67	3,362,112.31	905,330.19	9,349,889.85
29025	Caldwell	Missouri	9,424	455	146.00	759.50	193,678.68	26,732.72	554,691.47	346,169.20	28,355.61	2,256,193.44	539,847.88	63,833.76	2,645,534.60
29027	Callaway	Missouri	44,332	2,208	757.00	3,596.00	899,967.68	209,207.72	2,007,687.20	1,477,032.10	168,401.13	5,161,517.74	2,376,999.78	423,660.98	6,451,874.00
29029	Camden	Missouri	44,002	2,443	1,103.50	3,826.00	1,318,641.49	403,104.99	2,725,520.34	2,278,702.37	375,812.65	7,447,699.46	3,597,343.86	886,214.05	9,163,230.55
29031	Cape Girardeau	Missouri	75,674	4,333	1,900.50	6,716.00	1,795,212.19	668,695.13	3,412,657.57	3,123,287.65	606,033.25	9,954,011.54	4,918,499.84	1,456,630.18	12,088,173.55
29033	Carroll	Missouri	9,295	464	166.00	767.00	221,380.68	33,567.88	743,408.55	463,755.05	35,453.38	2,261,067.59	685,135.73	74,665.53	2,581,609.09
29035	Carter	Missouri	6,265	400	192.50	613.50	186,625.80	33,424.64	607,603.68	255,650.05	28,648.05	1,611,692.77	442,275.85	68,889.68	1,949,318.92
29037	Cass	Missouri	99,478	5,534	2,668.50	8,462.00	2,220,398.13	851,049.97	4,079,902.43	3,863,468.91	929,678.95	11,962,975.25	6,083,867.03	1,886,676.49	14,822,290.13
29039	Cedar	Missouri	13,982	880	479.00	1,325.50	437,167.62	120,380.44	1,164,437.68	748,472.16	96,140.19	3,345,802.82	1,185,639.78	233,803.45	3,890,477.58
29041	Chariton	Missouri	7,831	395	144.50	654.50	195,513.41	32,163.78	637,607.35	387,802.81	26,050.94	2,230,813.26	583,316.22	60,769.17	2,510,515.02
29043	Christian	Missouri	77,422	5,544	3,479.50	7,720.00	2,175,437.24	1,047,447.73	3,977,656.86	3,484,137.24	878,832.59	10,263,088.38	5,659,574.48	2,284,563.87	13,344,687.93
29045	Clark	Missouri	7,139	363	128.00	604.50	156,471.45	23,584.78	482,619.15	273,629.15	19,453.01	2,066,725.98	430,100.60	48,740.17	2,241,529.58
29047	Clay	Missouri	221,939	11,208	5,247.00	17,403.00	4,319,719.79	1,691,341.13	7,840,507.90	7,797,426.91	2,228,142.10	19,104,720.51	12,117,146.70	4,311,232.24	24,379,447.10
29049	Clinton	Missouri	20,743	1,011	399.50	1,618.50	442,864.80	102,270.54	1,071,675.83	783,631.98	86,838.03	3,819,926.85	1,226,496.78	228,986.55	4,324,580.07
29051	Cole	Missouri	75,990	3,958	1,617.00	6,387.50	1,632,459.57	558,422.51	3,300,737.34	2,845,380.57	448,858.36	9,587,038.73	4,477,840.14	1,040,736.53	11,342,924.94
29053	Cooper	Missouri	17,601	898	346.50	1,496.50	378,135.67	78,363.32	972,664.34	644,323.72	64,776.24	3,333,998.16	1,022,459.39	156,137.25	3,962,855.72
29055	Crawford	Missouri	24,696	1,374	524.50	2,208.00	593,049.20	152,726.66	1,439,120.86	987,794.57	139,149.42	4,086,779.06	1,580,843.76	312,100.95	4,848,018.83
29057	Dade	Missouri	7,883	533	281.50	764.00	273,026.27	61,058.39	775,340.25	498,080.74	56,520.17	2,635,360.90	771,107.01	138,517.18	3,108,675.37
29059	Dallas	Missouri	16,777	1,023	507.00	1,570.00	462,896.91	145,850.69	1,086,905.44	746,587.97	100,689.79	3,263,652.04	1,209,484.88	268,589.20	3,988,329.92
29061	Daviess	Missouri	8,433	416	140.50	692.00	195,686.89	24,373.97	589,589.86	302,033.02	23,750.46	1,763,725.26	497,719.91	55,087.24	2,175,106.50
29063	De Kalb	Missouri	12,892	586	235.00	965.00	259,377.22	48,998.88	770,627.30	453,759.62	39,215.87	2,813,600.52	713,136.85	96,493.53	3,260,479.68
29065	Dent	Missouri	15,657	931	403.00	1,407.50	440,906.23	111,480.08	1,194,178.81	914,255.44	90,544.25	3,996,060.33	1,355,161.67	226,068.96	4,639,687.40
29067	Douglas	Missouri	13,684	957	531.50	1,383.50	464,023.42	153,026.05	1,190,955.93	847,500.41	124,842.02	4,102,182.63	1,311,523.83	315,138.55	4,878,587.17
29069	Dunklin	Missouri	31,953	2,117	1,072.50	3,156.50	930,364.60	319,595.79	1,995,105.05	1,550,665.36	221,571.01	5,760,496.05	2,481,029.97	619,677.24	6,890,700.27
29071	Franklin	Missouri	101,492	5,155	2,152.50	8,638.50	2,134,811.01	753,531.13	4,062,156.56	3,700,850.40	657,392.08	11,294,709.99	5,835,661.41	1,585,746.35	14,295,130.20
29073	Gasconade	Missouri	15,222	776	272.00	1,283.00	381,738.74	72,159.99	964,350.04	624,178.83	59,081.24	2,887,338.99	1,005,917.57	140,709.83	3,563,356.07
29075	Gentry	Missouri	6,738	330	131.00	547.00	158,489.26	21,318.17	480,388.08	263,625.23	23,441.40	1,754,467.35	422,114.49	47,631.03	2,371,496.23
29077	Greene	Missouri	275,174	17,429	9,806.50	25,222.50	7,270,974.35	3,857,090.04	11,801,579.19	12,557,109.70	5,056,877.12	25,920,680.98	19,828,084.05	9,998,889.25	35,093,866.00
29079	Grundy	Missouri	10,261	498	182.50	800.50	240,949.14	40,701.40	710,667.53	397,401.48	43,020.20	2,077,658.44	638,350.62	95,777.80	2,568,804.40

29081	Harrison	Missouri	8,957	443	153.50	741.00	207,824.05	31,421.36	611,297.05	447,653.70	26,073.11	2,367,519.49	655,477.76	64,417.07	2,647,821.30
29083	Henry	Missouri	22,272	1,236	567.00	1,902.00	601,547.45	160,571.28	1,467,542.04	1,190,893.91	132,443.25	5,572,467.53	1,792,441.36	307,212.88	6,471,793.69
29085	Hickory	Missouri	9,627	553	258.50	844.50	348,946.43	85,878.30	1,023,001.72	576,916.88	60,033.87	3,121,788.89	925,863.31	162,552.30	3,485,572.41
29087	Holt	Missouri	4,912	235	90.00	387.00	117,182.48	11,419.05	370,224.47	198,567.17	15,582.96	1,487,267.77	315,749.65	32,342.45	1,692,640.40
29089	Howard	Missouri	10,144	507	177.00	838.50	218,374.23	34,609.93	665,702.32	379,682.80	26,300.04	2,436,975.80	598,057.03	66,979.51	2,835,001.27
29091	Howell	Missouri	40,400	2,840	1,644.00	4,211.50	1,236,451.37	508,476.76	2,408,951.73	2,100,737.36	437,046.24	7,190,450.20	3,337,188.74	1,123,634.62	8,465,454.40
29093	Iron	Missouri	10,630	598	244.50	942.50	279,359.44	54,432.92	789,141.94	526,764.51	71,910.16	2,630,706.66	806,123.95	132,919.24	3,248,162.79
29095	Jackson	Missouri	674,158	35,091	16,132.00	53,123.50	14,067,690.10	6,380,720.51	22,190,748.78	25,906,103.65	9,383,826.96	47,742,491.60	39,973,793.75	16,347,884.78	67,452,013.42
29097	Jasper	Missouri	117,404	9,612	5,814.50	13,483.00	3,809,663.86	2,042,043.85	6,128,999.61	6,648,721.52	2,099,022.79	18,783,967.64	10,458,385.38	4,459,495.35	23,734,019.56
29099	Jefferson	Missouri	218,733	11,257	4,093.00	18,142.00	4,401,048.18	1,577,232.27	8,060,437.36	7,199,797.80	1,609,001.80	18,322,894.51	11,600,845.98	3,433,279.38	24,150,333.87
29101	Johnson	Missouri	52,595	2,723	1,068.50	4,246.00	1,040,253.75	288,263.38	2,244,294.82	1,667,865.04	238,150.72	6,404,456.35	2,708,118.78	560,757.99	7,753,599.73
29103	Knox	Missouri	4,131	203	67.00	331.50	98,787.44	9,726.58	376,219.93	156,343.00	10,245.24	999,221.15	255,130.44	22,781.85	1,270,686.71
29105	Laclede	Missouri	35,571	2,151	1,019.00	3,325.00	929,307.47	317,902.51	1,962,280.64	1,557,462.95	263,085.71	6,204,849.96	2,486,770.42	661,271.60	7,501,365.81
29107	Lafayette	Missouri	33,381	1,722	715.00	2,780.00	760,413.07	211,993.36	1,670,638.81	1,252,849.78	159,002.04	5,259,950.94	2,013,262.85	431,311.11	6,272,622.35
29109	Lawrence	Missouri	38,634	3,010	1,895.50	4,231.00	1,301,968.28	585,437.35	2,589,059.80	2,275,196.18	508,331.39	9,010,641.20	3,577,164.47	1,224,556.28	10,742,523.69
29111	Lewis	Missouri	10,211	510	176.00	845.50	223,959.43	34,389.78	715,201.94	376,497.36	30,429.70	2,479,174.10	600,456.79	71,202.34	2,797,693.64
29113	Lincoln	Missouri	52,566	2,695	1,141.00	4,413.50	1,015,028.02	320,000.63	2,249,945.83	1,691,649.85	277,296.76	6,358,152.45	2,706,677.87	653,725.87	7,833,812.62
29115	Linn	Missouri	12,761	619	189.00	1,062.00	282,271.67	45,498.12	810,068.47	472,904.37	46,197.40	2,699,008.93	755,176.04	100,459.65	3,055,226.31
29117	Livingston	Missouri	15,195	713	257.50	1,194.50	321,921.88	60,369.34	877,325.21	650,261.12	55,963.71	3,149,650.58	972,183.00	133,673.45	3,571,751.31
29119	McDonald	Missouri	23,083	2,186	1,430.00	2,925.00	860,076.83	379,691.77	1,704,024.62	1,364,605.03	277,053.20	5,180,879.80	2,224,681.86	732,103.92	6,221,402.47
29121	Macon	Missouri	15,566	755	234.50	1,254.00	359,736.98	62,895.48	967,313.33	594,763.29	55,893.81	2,765,140.42	954,500.27	132,013.23	3,579,557.95
29123	Madison	Missouri	12,226	684	292.50	1,050.50	308,946.08	66,892.32	814,160.03	527,311.47	55,933.99	2,954,650.77	836,257.54	136,725.46	3,345,550.31
29125	Maries	Missouri	9,176	505	200.00	808.50	247,864.52	32,742.72	775,339.59	413,700.29	32,444.68	2,474,236.81	661,564.82	71,863.89	2,969,598.30
29127	Marion	Missouri	28,781	1,458	509.50	2,356.50	627,511.98	143,818.52	1,447,749.70	1,097,222.01	122,762.94	4,367,302.11	1,724,733.99	289,232.73	5,281,250.12
29129	Mercer	Missouri	3,785	187	65.50	313.00	87,513.45	9,187.07	314,995.38	144,082.04	18,240.16	813,627.82	231,595.50	32,154.72	1,052,690.52
29131	Miller	Missouri	24,748	1,348	546.00	2,099.00	593,531.24	147,819.65	1,485,142.12	1,226,357.10	119,497.45	5,579,689.20	1,819,888.34	305,883.31	6,496,790.98
29133	Mississippi	Missouri	14,358	872	423.00	1,337.50	373,050.00	99,775.79	1,004,327.44	616,682.39	78,565.41	3,047,247.91	989,732.39	203,289.61	3,575,866.47
29135	Moniteau	Missouri	15,607	826	332.50	1,314.00	332,659.32	73,789.74	953,100.30	549,315.89	57,378.69	3,203,131.98	881,975.21	145,606.20	3,537,781.80
29137	Monroe	Missouri	8,840	454	153.00	742.00	214,565.27	28,539.45	676,456.23	413,087.57	28,215.84	2,313,453.36	627,652.84	64,073.79	2,715,163.78
29139	Montgomery	Missouri	12,236	639	250.00	1,040.00	304,431.90	58,266.70	842,416.73	572,276.12	49,458.82	2,874,095.78	876,708.01	123,302.17	3,280,795.13
29141	Morgan	Missouri	20,565	1,120	486.00	1,831.00	575,958.11	131,769.69	1,407,093.43	966,913.20	109,227.18	3,820,472.53	1,542,871.31	261,588.86	4,679,964.24
29143	New Madrid	Missouri	18,956	1,190	544.50	1,857.50	531,121.52	151,622.44	1,324,674.85	858,037.27	146,276.53	3,664,640.13	1,389,158.80	334,271.29	4,557,887.78
29145	Newton	Missouri	58,114	5,119	3,181.50	6,980.00	2,210,087.92	1,132,106.46	3,882,567.87	3,981,331.90	1,160,451.28	11,738,554.99	6,191,419.82	2,594,478.06	14,075,795.53
29147	Nodaway	Missouri	23,370	1,086	420.00	1,795.50	425,574.71	91,947.13	1,028,156.75	720,741.90	86,131.87	3,822,661.13	1,146,316.61	185,948.15	4,527,854.99
29149	Oregon	Missouri	10,881	741	362.00	1,089.50	382,964.56	92,079.80	1,019,802.06	707,163.15	74,823.72	3,936,572.79	1,090,127.71	196,803.61	4,572,281.77
29151	Osage	Missouri	13,878	719	255.00	1,178.00	310,194.53	58,618.81	884,331.35	548,272.30	51,733.71	2,772,920.59	858,466.83	127,049.33	3,309,901.32
29153	Ozark	Missouri	9,723	709	417.50	1,005.00	371,919.41	111,328.02	1,012,277.48	600,236.75	76,298.93	2,950,727.10	972,156.16	216,179.61	3,466,195.87
29155	Pemiscot	Missouri	18,296	1,218	599.50	1,829.50	496,857.38	145,285.22	1,215,983.58	857,018.56	120,426.15	4,255,037.25	1,353,875.94	301,560.86	4,772,394.02
29157	Perry	Missouri	18,971	1,064	476.00	1,710.00	466,975.75	104,520.93	1,147,857.61	796,130.94	96,347.30	3,918,700.85	1,263,106.69	209,188.15	4,589,640.15
29159	Pettis	Missouri	42,201	2,259	926.00	3,601.00	938,741.80	264,663.50	2,074,408.24	1,676,983.20	236,615.36	6,078,608.75	2,615,724.99	581,097.79	7,665,001.15
29161	Phelps	Missouri	45,156	2,498	1,049.50	3,937.00	1,028,979.58	314,688.95	2,208,015.30	1,730,440.59	274,219.15	6,643,206.59	2,759,420.17	679,143.39	7,820,379.39
29163	Pike	Missouri	18,516	938	378.00	1,534.00	412,967.23	88,043.95	1,069,356.36	673,890.67	78,682.63	3,028,170.11	1,086,857.90	178,238.15	3,795,503.88
29165	Platte	Missouri	89,322	4,378	1,849.00	7,056.50	1,746,336.65	565,947.70	3,357,240.73	2,841,937.01	581,665.43	9,232,811.87	4,588,273.66	1,295,947.89	11,901,745.77
29167	Polk	Missouri	31,137	1,924	980.50	2,857.00	838,308.77	278,163.56	1,803,971.99	1,408,245.93	231,993.35	5,838,843.47	2,246,554.70	578,700.23	7,035,039.73
29169	Pulaski	Missouri	52,274	3,009	1,407.50	4,644.00	962,235.27	329,464.23	2,062,045.20	1,612,388.10	336,435.72	6,175,115.70	2,574,623.36	737,073.20	7,537,858.84
29171	Putnam	Missouri	4,979	233	75.00	393.50	117,273.72	11,100.62	456,604.27	242,586.97	14,896.98	1,411,243.07	359,860.70	27,671.27	1,723,040.66
29173	Ralls	Missouri	10,167	507	191.50	858.00	239,900.85	39,295.18	732,536.31	361,225.97	40,417.87	1,768,963.20	601,126.82	88,259.35	2,358,189.62
29175	Randolph	Missouri	25,414	1,213	360.00	2,027.00	534,490.72	100,953.93	1,279,902.71	899,325.66	87,093.34	3,919,730.66	1,433,816.39	210,308.06	4,730,921.92
29177	Ray	Missouri	23,494	1,184	482.50	1,927.50	513,732.31	127,540.52	1,166,084.58	887,218.45	97,734.15	3,902,373.75	1,400,950.76	242,701.17	4,693,912.10
29179	Reynolds	Missouri	6,696	373	164.50	581.00	180,131.13	31,101.84	550,525.70	373,742.37	38,164.93	2,412,027.07	553,873.50	76,110.19	2,714,476.77
29181	Ripley	Missouri	14,100	918	456.00	1,358.50	431,414.45	123,058.73	1,070,529.20	716,696.05	88,643.08	3,511,613.72	1,148,110.49	226,851.92	3,931,690.83
29183	St. Charles	Missouri	360,485	18,352	7,659.00	28,970.00	7,137,844.82	2,495,961.65	12,350,335.31	12,090,558.74	3,285,106.74	26,313,819.90	19,228,403.55	6,059,084.98	35,120,870.10
29185	St. Clair	Missouri	9,805	563	271.50	877.50	300,867.84	77,807.78	862,658.71	545,951.74	56,528.27	3,011,152.83	846,819.58	150,163.30	3,402,069.85
29186	Ste. Genevieve	Missouri	18,145	986	420.00	1,557.00	452,146.03	103,404.39	1,269,501.47	801,032.46	107,938.28	3,765,378.48	1,253,178.49	219,672.10	4,697,016.83
29187	St. Francois	Missouri	65,359	3,474	1,565.00	5,611.00	1,452,060.61	489,728.62	2,966,753.71	2,521,794.25	436,240.71	9,460,828.69	3,973,854.86	1,018,949.30	11,294,016.47
29189	St. Louis	Missouri	998,954	49,447	20,439.00	79,332.00	21,574,091.32	8,776,022.58	35,539,810.87	42,040,695.47	16,281,809.87	79,911,398.54	63,614,786.79	26,448,926.66	111,510,636.00
29195	Saline	Missouri	23,370	1,203	400.00	1,959.50	525,196.45	99,347.35	1,326,370.47	916,585.83	83,024.72	4,624,582.79	1,441,782.29	195,596.39	5,248,201.03
29197	Schuyler	Missouri	4,431	227	90.50	370.00	103,712.93	12,757.39	385,763.47	217,107.78	14,728.58	1,492,778.85	320,820.72	30,114.19	1,836,996.52
29199	Scotland	Missouri	4,843	253	94.00	418.50	112,795.40	13,289.51	413,392.40	179,879.29	13,592.18	1,240,557.03	292,674.69	30,141.31	1,626,149.03
29201	Scott	Missouri	39,191	2,391	949.00	3,706.50	1,013,071.44	305,582.18	2,271,644.58	1,749,343.62	268,316.74	6,275,478.40	2,762,415.06	652,699.32	7,688,007.14
29203	Shannon	Missouri	8,441	525	252.50	803.00	258,940.32	53,670.44	830,938.47	450,807.10	38,779.69	2,389,409.04	709,747.42	101,334.69	2,781,490.09
29205	Shelby	Missouri	6,373	330	118.00	546.00	159,407.48	20,898.94	601,806.60	237,454.02	19,643.05	1,368,238.57	396,861.50	41,604.23	1,748,145.63
29207	Stoddard	Missouri	29,968	1,823	810.50	2,770.00	847,647.49	273,155.95	1,845,331.95	1,526,802.97	214,797.19	5,871,524.58	2,374,450.47	549,177.77	7,027,463.38
29209	Stone	Missouri	32,202	2,530	1,564.00	3,483.50	1,448,623.09	658,631.86	2,846,708.20	2,620,693.93	537,099.76	8,873,190.32	4,069,317.02	1,371,365.29	10,590,416.79
29211	Sullivan	Missouri	6,714	321	113.00	535.00	143,309.63	17,514.77	484,641.76	340,407.92	24,887.38	1,697,327.65	483,717.55	45,753.14	1,901,649.12
29213	Taney	Missouri	51,675	4,013	2,638.00	5,612.50	1,828,207.36	898,992.78	3,342,537.20	3,073,341.57	803,765.93	8,992,691.44	4,901,548.93	1,971,959.82	11,405,377.76

29215	Texas	Missouri	26,008	1,644	807.50	2,478.50	789,202.45	233,450.62	1,754,604.43	1,404,516.99	181,815.48	5,822,724.78	2,193,719.44	461,847.88	6,760,536.03
29217	Vernon	Missouri	21,159	1,410	758.50	2,092.00	636,826.59	202,964.12	1,545,659.69	1,031,061.04	160,407.93	4,801,474.49	1,667,887.62	406,353.53	5,707,229.69
29219	Warren	Missouri	32,513	1,715	611.00	2,769.00	728,748.06	180,775.01	1,562,127.26	1,198,191.22	142,553.28	4,936,894.12	1,926,939.28	331,133.08	5,993,280.94
29221	Washington	Missouri	25,195	1,354	543.50	2,184.00	581,083.46	125,082.56	1,525,449.83	938,350.18	91,231.42	4,794,232.35	1,519,433.64	273,151.86	5,850,077.54
29223	Wayne	Missouri	13,521	802	363.00	1,220.00	404,704.20	99,259.47	1,112,038.71	752,961.19	71,146.45	3,801,743.61	1,157,665.40	187,607.74	4,404,527.40
29225	Webster	Missouri	36,202	2,395	1,271.00	3,476.00	949,307.41	328,076.09	1,936,124.24	1,571,209.57	260,847.86	6,060,901.03	2,520,516.99	688,249.31	7,537,643.96
29227	Worth	Missouri	2,171	103	36.00	180.50	62,548.51	3,139.93	295,604.08	102,137.20	4,904.20	797,734.03	164,685.72	9,108.37	996,210.46
29229	Wright	Missouri	18,815	1,253	647.50	1,858.50	549,051.19	179,693.59	1,258,365.17	910,717.40	131,668.47	4,131,229.57	1,459,768.59	355,562.67	5,107,562.14
29510	St. Louis City	Missouri	319,294	15,860	6,642.00	24,646.00	6,251,133.39	2,363,534.24	10,909,393.00	11,694,416.09	3,460,695.71	26,751,221.07	17,945,549.48	6,024,356.26	36,274,994.53
30001	Beaverhead	Montana	9,246	509	146.50	885.50	252,880.06	27,254.07	814,549.83	487,264.06	29,385.37	3,571,534.23	740,144.12	58,039.96	3,797,666.92
30003	Big Horn	Montana	12,865	688	71.50	1,317.50	256,964.81	8,331.11	883,353.94	386,477.97	15,542.04	2,316,807.55	643,442.78	27,278.32	2,649,197.97
30005	Blaine	Montana	6,491	384	36.50	742.50	165,737.54	4,664.38	679,886.86	249,831.99	7,056.26	1,806,689.99	415,569.54	12,222.95	2,020,588.53
30007	Broadwater	Montana	5,612	288	52.00	535.00	144,100.81	7,971.06	507,542.58	214,588.53	9,179.15	1,371,631.93	358,689.34	18,613.82	1,599,390.83
30009	Carbon	Montana	10,078	461	45.00	919.00	247,248.98	6,212.08	802,967.43	458,629.57	6,857.62	2,396,972.05	705,878.55	12,617.87	2,833,518.42
30011	Carter	Montana	1,160	57	7.00	114.50	30,217.22	450.91	154,665.06	68,148.84	1,048.11	556,366.96	98,366.06	1,789.60	638,811.00
30013	Cascade	Montana	81,327	4,707	707.00	8,577.00	2,082,286.89	295,746.14	4,453,441.87	3,766,229.16	210,956.38	12,499,888.53	5,848,516.04	654,802.17	15,192,390.75
30015	Chouteau	Montana	5,813	356	43.50	674.00	159,978.23	5,203.84	556,310.59	279,393.23	7,539.19	1,881,545.58	439,371.46	13,396.72	2,415,920.77
30017	Custer	Montana	11,699	558	50.50	1,144.00	257,973.55	9,793.32	814,975.90	502,091.92	10,635.61	3,225,207.56	760,065.48	23,259.92	3,447,937.72
30019	Daniels	Montana	1,751	106	14.00	205.00	57,957.64	1,634.78	270,600.14	111,193.92	3,376.53	837,625.45	169,151.56	5,772.00	1,017,735.82
30021	Dawson	Montana	8,966	506	57.50	980.00	263,403.18	14,081.94	770,653.82	423,205.62	13,478.78	2,319,270.24	686,608.80	30,141.31	2,848,174.12
30023	Deer Lodge	Montana	9,298	505	120.50	849.00	258,526.78	29,673.09	851,047.54	450,237.14	22,256.31	2,537,989.79	708,763.93	58,509.37	3,077,584.35
30025	Fallon	Montana	2,890	158	22.50	306.50	74,797.65	2,512.63	329,287.32	123,482.38	5,751.12	751,529.95	198,280.03	8,873.89	985,510.89
30027	Fergus	Montana	11,586	667	112.50	1,210.50	343,614.75	27,801.14	1,013,424.74	641,634.43	25,670.53	3,464,192.02	985,249.18	61,955.17	3,952,827.29
30029	Flathead	Montana	90,928	5,584	1,533.00	9,603.00	2,466,631.08	535,563.63	4,953,155.58	4,008,674.77	536,504.74	12,053,409.42	6,475,305.85	1,151,411.75	15,331,068.89
30031	Gallatin	Montana	89,513	4,306	603.00	8,037.50	1,634,524.50	198,364.20	3,718,283.41	2,629,677.26	128,685.66	8,947,398.90	4,264,201.76	336,539.15	11,779,941.72
30033	Garfield	Montana	1,206	62	4.00	127.00	28,119.29	102.04	133,888.03	42,801.80	362.04	372,345.86	70,921.09	537.90	500,592.91
30035	Glacier	Montana	13,399	854	188.50	1,599.00	310,307.46	43,648.56	875,382.56	494,339.57	36,765.32	2,632,818.06	804,647.03	94,201.89	3,120,742.44
30037	Golden Valley	Montana	884	47	5.00	91.00	22,885.33	279.99	136,060.19	38,941.27	775.02	276,787.65	61,826.61	1,341.79	452,258.13
30039	Granite	Montana	3,079	173	47.00	296.00	98,253.13	7,167.91	381,323.14	169,708.34	10,019.01	1,090,813.16	267,961.48	19,487.37	1,312,109.16
30041	Hill	Montana	16,096	989	179.50	1,889.00	382,234.67	42,076.10	998,552.41	609,082.44	40,675.75	3,071,672.75	991,317.11	88,210.80	3,666,374.45
30043	Jefferson	Montana	11,406	595	106.50	1,146.00	280,687.10	19,893.90	848,880.85	438,711.27	22,524.57	2,679,554.04	719,398.37	43,868.80	3,153,372.57
30045	Judith Basin	Montana	2,072	116	17.50	216.00	55,270.96	1,577.34	245,615.21	121,005.48	2,921.82	834,561.64	176,276.44	5,145.71	948,360.75
30047	Lake	Montana	28,746	1,779	552.50	3,015.50	810,478.85	142,854.63	2,036,072.76	1,394,006.51	125,489.83	5,383,961.10	2,204,485.36	321,027.74	7,197,942.57
30049	Lewis and Clark	Montana	63,395	3,573	695.00	6,541.00	1,556,841.87	257,193.69	3,453,734.28	2,764,633.60	230,991.56	9,088,173.56	4,321,475.47	527,253.72	11,781,523.79
30051	Liberty	Montana	2,339	142	23.00	264.50	66,813.60	2,552.73	301,229.57	113,144.07	3,545.21	976,348.93	179,957.67	6,380.10	1,138,846.31
30053	Lincoln	Montana	19,687	1,262	454.50	2,049.00	665,615.51	151,699.39	1,591,041.58	1,247,377.42	108,648.69	5,160,594.29	1,912,992.92	312,985.11	6,133,704.52
30055	McCone	Montana	1,734	97	14.00	187.50	50,221.51	1,154.75	216,661.76	87,936.19	2,471.35	626,886.13	138,157.70	4,204.63	833,525.77
30057	Madison	Montana	7,691	389	64.00	726.00	223,409.10	15,055.17	735,694.07	451,667.73	14,079.17	2,098,237.27	675,076.83	33,078.72	2,560,896.37
30059	Meagher	Montana	1,891	103	16.00	192.00	57,865.77	1,527.16	251,118.24	93,737.12	2,302.52	648,863.36	151,602.89	3,990.66	865,151.28
30061	Mineral	Montana	4,223	270	115.00	426.50	146,000.94	21,411.69	547,392.22	231,387.39	19,337.55	1,438,255.58	377,388.34	42,905.37	1,703,315.87
30063	Missoula	Montana	109,299	6,227	2,075.00	10,437.00	2,558,214.72	699,876.85	5,062,704.97	4,175,215.49	651,052.34	13,438,460.60	6,733,430.21	1,511,115.87	16,559,521.79
30065	Musselshell	Montana	4,538	250	36.00	467.00	132,557.46	4,880.17	497,348.33	271,241.95	7,835.48	1,566,112.74	403,799.40	13,872.36	1,842,467.01
30067	Park	Montana	15,636	759	123.50	1,470.00	370,836.80	23,361.25	1,157,257.58	714,373.13	26,676.95	3,419,841.13	1,085,209.94	54,084.49	4,078,864.03
30069	Petroleum	Montana	494	27	3.00	55.00	13,174.90	80.20	83,186.06	25,766.25	262.53	177,760.25	38,941.15	441.08	244,597.96
30071	Phillips	Montana	4,253	244	30.50	472.50	118,566.58	4,166.34	438,370.16	226,810.12	5,982.07	1,703,334.54	345,376.70	12,548.02	1,962,527.22
30073	Pondera	Montana	6,153	393	67.00	723.50	184,544.91	13,289.81	624,257.87	317,573.49	13,872.39	1,981,901.60	502,118.41	29,215.14	2,275,205.32
30075	Powder River	Montana	1,743	85	7.00	167.50	44,865.04	314.70	206,536.91	58,189.82	812.63	486,813.95	103,054.85	1,287.97	772,539.79
30077	Powell	Montana	7,027	370	96.50	694.50	173,225.55	15,450.73	619,458.61	359,100.60	20,632.53	1,950,855.52	532,326.14	39,829.87	2,208,319.69
30079	Prairie	Montana	1,179	65	7.00	129.00	38,830.56	351.30	228,409.43	80,315.19	1,144.82	702,847.61	119,145.75	1,562.05	849,950.63
30081	Ravalli	Montana	40,212	2,393	921.00	3,943.00	1,183,082.20	335,701.98	2,360,090.18	2,100,526.56	275,784.63	6,769,899.39	3,283,608.76	716,897.51	8,570,356.63
30083	Richland	Montana	9,746	555	45.00	1,073.50	251,990.00	6,486.10	795,902.65	414,043.20	9,558.65	2,502,620.37	666,033.20	17,827.13	2,845,395.94
30085	Roosevelt	Montana	10,425	661	101.00	1,234.00	252,050.00	17,371.25	710,066.76	419,492.78	17,959.61	2,650,819.93	671,542.78	46,644.58	3,098,174.73
30087	Rosebud	Montana	9,233	506	69.00	968.00	195,737.67	9,576.89	626,214.04	326,639.00	16,291.28	1,950,874.09	522,376.66	27,685.31	2,245,380.79
30089	Sanders	Montana	11,413	741	270.50	1,224.50	402,637.97	76,670.36	1,112,906.02	624,328.78	62,645.04	3,272,543.75	1,026,966.75	157,825.80	4,045,772.56
30091	Sheridan	Montana	3,384	198	32.50	376.50	113,625.61	5,353.05	449,115.37	240,608.33	6,280.73	1,727,329.33	354,233.95	14,946.49	1,965,644.39
30093	Silver Bow	Montana	34,200	1,866	493.00	3,292.50	859,848.50	144,902.19	1,992,759.58	1,582,932.13	135,051.20	6,199,618.30	2,442,780.63	307,025.90	7,570,158.38
30095	Stillwater	Montana	9,117	427	41.50	846.50	200,637.09	7,255.25	654,999.78	421,799.32	11,609.32	2,033,389.90	622,436.41	19,801.35	2,618,252.66
30097	Sweet Grass	Montana	3,651	174	27.00	335.50	87,792.78	2,664.69	359,222.34	211,086.20	6,137.46	1,549,872.14	298,878.97	9,228.56	1,706,467.69
30099	Teton	Montana	6,073	352	75.50	637.00	184,401.32	13,749.53	741,116.11	294,222.52	16,176.21	1,707,329.58	478,623.84	30,663.18	2,324,494.92
30101	Toole	Montana	5,324	318	51.50	588.00	152,296.15	7,363.99	591,509.99	243,995.36	9,188.32	1,353,502.13	396,291.50	17,856.25	1,842,299.94
30103	Treasure	Montana	718	38	6.00	77.00	20,708.70	240.15	130,569.26	35,277.92	843.47	256,146.20	55,986.62	1,363.57	352,067.79
30105	Valley	Montana	7,369	434	59.50	847.50	216,081.29	10,545.68	668,180.23	347,190.94	14,009.03	1,759,307.27	563,272.23	26,369.02	2,063,490.95
30107	Wheatland	Montana	2,168	124	19.50	232.50	62,304.45	2,348.31	289,588.14	114,467.23	3,311.98	913,714.09	176,771.68	6,440.93	1,064,092.39
30109	Wibaux	Montana	1,017	56	10.00	112.00	36,729.24	477.58	178,685.13	54,459.04	1,283.92	565,691.96	91,188.28	1,904.40	701,157.79
30111	Yellowstone	Montana	147,972	7,723	1,021.50	15,046.50	3,312,065.05	399,356.48	6,674,773.45	5,702,633.62	348,448.80	17,477,633.17	9,014,698.67	769,470.49	23,316,848.33
30113	wstone National Park (	Montana	0	0	0.00	0.00	0.00	0.00	0.00	0.00	0.00	0.00	0.00	0.00	0.00

31001	Adams	Nebraska	31,364	1,592	375.00	2,734.00	711,891.09	119,291.53	1,720,017.13	1,152,759.43	95,322.57	5,185,803.14	1,864,650.51	256,977.00	6,281,286.77
31003	Antelope	Nebraska	6,685	313	81.00	563.00	161,426.67	13,308.02	592,713.01	289,554.77	15,201.16	2,154,347.69	450,981.45	29,616.28	2,396,750.17
31005	Arthur	Nebraska	460	25	5.00	48.00	10,294.47	112.67	66,203.97	24,596.84	575.04	138,956.23	34,891.31	843.41	198,285.40
31007	Banner	Nebraska	690	33	4.00	64.00	17,588.42	55.72	107,700.46	27,491.53	427.83	209,855.64	45,079.95	514.90	309,563.74
31009	Blaine	Nebraska	478	23	4.00	43.00	10,778.22	48.26	82,731.35	16,149.78	418.15	82,759.49	26,928.00	510.11	156,368.40
31011	Boone	Nebraska	5,505	266	76.00	452.50	136,256.16	11,491.53	532,891.00	263,637.12	16,170.80	1,785,169.02	399,893.28	29,357.60	1,998,020.51
31013	Box Butte	Nebraska	11,308	527	49.00	1,029.00	242,721.78	7,208.78	779,187.52	410,556.46	12,261.78	2,057,582.36	653,278.24	21,591.75	2,402,914.29
31015	Boyd	Nebraska	2,099	99	21.00	185.50	52,518.65	1,806.27	234,491.94	104,180.80	3,130.54	786,264.21	156,699.45	4,944.15	925,266.28
31017	Brown	Nebraska	3,145	140	20.50	260.00	75,268.73	1,826.98	329,292.34	118,675.40	3,753.35	853,568.11	193,944.13	7,559.73	993,870.60
31019	Buffalo	Nebraska	46,102	2,261	562.00	4,098.50	872,445.32	157,651.68	2,063,921.12	1,528,817.89	133,058.65	5,892,524.22	2,401,263.21	368,456.61	7,005,704.01
31021	Burt	Nebraska	6,858	339	128.00	550.50	175,996.12	28,656.02	595,441.17	296,211.61	27,302.62	1,760,941.18	472,207.73	61,245.34	1,994,137.08
31023	Butler	Nebraska	8,395	421	162.50	684.50	223,579.23	34,555.04	665,035.58	354,125.79	36,842.79	2,251,817.43	577,705.02	79,458.29	2,747,855.67
31025	Cass	Nebraska	25,241	1,252	505.50	1,984.50	540,351.06	137,921.04	1,350,610.78	846,317.75	117,225.99	3,751,656.25	1,386,668.81	297,442.34	4,452,141.30
31027	Cedar	Nebraska	8,852	463	157.00	771.50	223,288.21	30,813.68	760,531.45	398,522.94	31,288.68	2,265,615.95	621,811.15	64,116.27	2,543,462.72
31029	Chase	Nebraska	3,966	223	60.50	381.00	106,859.35	8,149.10	401,261.08	162,288.69	12,174.37	1,028,424.84	269,148.04	24,092.48	1,220,950.41
31031	Cherry	Nebraska	5,713	256	35.50	508.50	143,246.02	5,688.23	458,943.47	262,440.08	6,539.23	1,779,208.08	405,686.09	13,703.78	2,044,952.16
31033	Cheyenne	Nebraska	9,998	507	79.50	912.50	218,262.06	11,176.59	673,878.47	408,183.23	20,167.03	2,348,874.59	626,445.29	32,760.56	2,598,882.08
31035	Clay	Nebraska	6,542	352	96.50	580.00	178,920.41	15,639.30	603,355.73	243,627.25	18,286.56	1,218,358.02	422,547.65	39,337.82	1,668,823.09
31037	Colfax	Nebraska	10,515	541	193.50	884.50	208,750.74	34,993.30	653,309.33	390,115.88	37,151.08	1,940,165.79	598,866.62	75,543.36	2,327,811.62
31039	Cuming	Nebraska	9,139	456	150.50	720.00	215,303.94	38,808.89	683,113.99	434,534.63	33,313.95	2,117,203.46	649,838.57	78,223.78	2,563,822.81
31041	Custer	Nebraska	10,939	518	85.00	970.00	248,408.28	18,551.28	807,647.08	433,678.97	21,367.69	2,396,717.47	682,087.25	43,736.60	2,908,185.02
31043	Dakota	Nebraska	21,006	1,082	399.50	1,812.50	386,123.32	70,694.33	1,020,033.64	694,875.96	79,756.08	3,183,722.76	1,080,999.28	162,795.60	3,809,870.97
31045	Dawes	Nebraska	9,182	406	49.50	811.00	183,828.44	5,594.38	644,810.10	292,906.71	6,933.62	1,795,278.07	476,735.16	13,918.08	2,125,870.84
31047	Dawson	Nebraska	24,326	1,288	335.50	2,246.00	522,412.84	76,286.59	1,316,259.79	871,390.65	63,633.26	4,162,637.87	1,393,803.49	157,137.55	5,073,067.67
31049	Deuel	Nebraska	1,941	101	21.00	179.50	59,992.99	1,900.62	284,260.22	92,646.19	3,819.13	737,291.91	152,639.17	6,089.12	877,517.79
31051	Dixon	Nebraska	6,000	306	98.50	508.00	143,683.82	18,617.32	499,062.25	247,651.50	17,652.98	1,529,347.89	391,335.31	38,957.31	1,747,859.46
31053	Dodge	Nebraska	36,691	1,812	699.00	2,892.00	823,171.25	222,725.89	1,791,748.91	1,595,342.22	170,903.68	6,740,104.03	2,418,513.47	431,190.79	8,104,842.49
31055	Douglas	Nebraska	517,110	25,772	10,642.50	39,880.50	9,594,840.97	3,748,452.83	16,106,184.94	17,676,287.68	5,268,686.16	37,183,308.51	27,271,128.65	9,204,100.32	51,327,418.22
31057	Dundy	Nebraska	2,008	114	35.50	199.00	57,274.07	3,857.49	256,845.47	456,824.07	7,753.48	817,201.56	514,098.15	13,220.81	1,017,910.84
31059	Fillmore	Nebraska	5,890	327	135.50	523.00	153,007.40	23,518.00	500,683.36	309,239.39	29,369.09	1,804,959.21	462,246.79	56,058.20	2,155,326.11
31061	Franklin	Nebraska	3,225	167	39.00	295.50	82,841.27	5,903.75	316,016.21	150,101.20	7,788.68	1,246,834.51	232,942.47	15,694.50	1,476,510.14
31063	Frontier	Nebraska	2,756	150	36.50	262.00	70,863.00	4,568.02	300,672.43	125,491.67	7,177.36	1,014,474.92	196,354.67	12,911.29	1,190,525.37
31065	Furnas	Nebraska	4,959	279	85.50	471.50	137,462.00	18,878.07	445,431.67	288,091.67	21,609.70	1,722,761.55	425,553.67	46,694.13	2,108,158.70
31067	Gage	Nebraska	22,311	1,186	440.00	1,864.50	579,394.90	124,037.33	1,518,723.24	1,049,491.29	107,038.18	4,418,859.50	1,628,886.19	283,819.54	5,281,464.15
31069	Garden	Nebraska	2,057	102	20.00	187.00	52,813.84	2,012.10	241,199.49	103,727.88	3,782.87	768,898.38	156,541.72	6,020.58	934,179.01
31071	Garfield	Nebraska	2,049	97	18.00	175.00	56,067.53	1,528.63	256,064.68	93,445.89	2,603.88	720,343.36	149,513.41	4,613.96	849,745.84
31073	Gosper	Nebraska	2,044	112	33.00	193.50	58,920.20	3,576.24	250,298.38	83,955.15	5,870.81	543,189.54	142,875.35	10,306.57	750,300.39
31075	Grant	Nebraska	614	30	4.00	58.00	16,323.83	89.35	118,696.91	28,987.75	533.70	241,894.95	45,311.58	769.21	307,011.10
31077	Greeley	Nebraska	2,538	120	25.50	219.00	63,124.10	2,553.29	246,091.27	123,937.10	4,678.11	941,115.65	187,061.20	8,010.48	1,138,549.33
31079	Hall	Nebraska	58,607	2,943	701.00	5,188.50	1,184,833.76	226,836.08	2,428,700.25	1,910,855.57	203,541.84	7,275,002.37	3,095,689.33	517,107.64	9,067,226.35
31081	Hamilton	Nebraska	9,124	453	151.00	764.00	206,150.35	29,493.35	628,171.72	340,385.19	32,972.02	1,848,710.47	546,535.54	66,238.57	2,405,843.86
31083	Harlan	Nebraska	3,423	189	54.50	327.50	103,818.29	8,080.19	400,447.99	186,455.48	10,078.40	1,212,699.96	290,273.78	19,026.58	1,505,508.52
31085	Hayes	Nebraska	967	53	13.00	96.00	26,377.05	791.17	149,563.16	52,903.20	2,586.63	334,831.92	79,280.25	3,509.77	438,653.40
31087	Hitchcock	Nebraska	2,908	167	52.50	284.00	90,796.51	6,660.27	386,569.09	137,510.15	11,217.58	912,724.76	228,306.65	18,404.64	1,202,835.43
31089	Holt	Nebraska	10,435	487	83.50	894.00	245,101.24	14,854.60	759,387.14	390,911.55	19,370.09	1,947,809.13	636,012.79	42,119.88	2,509,907.28
31091	Hooker	Nebraska	736	36	5.00	69.00	17,728.32	168.65	98,342.76	35,699.52	461.55	302,936.85	53,427.84	734.06	364,136.60
31093	Howard	Nebraska	6,274	289	56.00	509.00	133,973.82	9,426.75	490,116.26	217,468.89	10,015.16	1,587,884.58	351,442.70	22,912.55	1,904,489.92
31095	Jefferson	Nebraska	7,547	420	178.00	663.50	213,741.35	40,140.67	707,219.06	377,321.80	43,542.66	1,773,253.32	591,063.15	94,236.01	2,144,700.95
31097	Johnson	Nebraska	5,217	258	119.00	411.00	125,189.01	17,518.05	470,941.18	204,422.66	23,262.37	1,109,834.96	329,611.67	47,132.51	1,303,396.69
31099	Kearney	Nebraska	6,489	337	89.00	576.50	151,892.16	14,038.79	510,562.13	298,116.91	18,164.20	1,963,555.91	450,009.07	38,431.75	2,245,999.90
31101	Keith	Nebraska	8,368	426	79.00	785.50	221,931.17	18,341.86	709,953.45	405,540.67	16,355.25	1,988,812.21	627,471.84	36,021.24	2,361,045.85
31103	Keya Paha	Nebraska	824	37	4.50	74.00	21,141.91	86.46	130,534.81	26,172.97	651.19	251,475.19	47,314.88	772.70	376,003.51
31105	Kimball	Nebraska	3,821	189	43.50	360.50	95,625.25	5,266.14	395,745.55	166,641.70	8,460.92	1,033,670.25	262,266.95	15,333.39	1,286,514.37
31107	Knox	Nebraska	8,701	412	109.50	741.00	208,419.05	19,955.02	659,587.22	363,125.07	20,126.86	2,229,150.88	571,544.12	43,737.58	2,525,822.65
31109	Lancaster	Nebraska	285,407	14,262	6,277.50	21,896.00	5,439,100.66	2,165,709.19	9,044,693.11	9,484,731.83	2,486,393.46	23,164,547.34	14,923,832.49	4,886,475.74	31,140,662.53
31111	Lincoln	Nebraska	36,288	1,935	394.00	3,411.00	855,511.93	134,412.65	1,937,931.07	1,612,752.98	120,748.78	6,427,012.37	2,468,264.91	294,472.05	7,737,035.56
31113	Logan	Nebraska	763	38	6.00	76.00	18,053.73	314.00	126,868.07	29,701.71	773.06	176,319.77	47,755.44	1,311.17	319,142.60
31115	Loup	Nebraska	632	30	5.50	57.50	16,721.19	151.96	111,199.57	23,034.95	722.71	218,169.66	39,756.14	1,029.35	330,617.66
31117	McPherson	Nebraska	539	30	5.50	57.00	13,926.31	158.31	87,675.18	27,243.13	593.51	114,465.25	41,169.44	871.06	217,086.61
31119	Madison	Nebraska	34,876	1,770	606.00	2,997.00	753,473.35	168,603.33	1,758,652.55	1,217,964.12	157,419.19	4,750,858.96	1,971,437.47	347,516.83	5,701,862.41
31121	Merrick	Nebraska	7,845	375	92.50	644.50	172,498.66	18,326.35	562,250.97	284,247.81	15,539.74	1,803,572.90	456,746.47	39,075.28	2,198,950.76
31123	Morrill	Nebraska	5,042	249	33.00	467.50	116,845.31	4,562.08	448,851.15	255,693.50	7,366.20	1,605,229.41	372,538.81	13,344.38	1,841,055.61
31125	Nance	Nebraska	3,735	204	67.50	340.00	99,926.44	11,415.55	401,861.57	153,425.24	13,011.13	1,033,693.08	253,351.68	28,696.17	1,269,044.60
31127	Nemaha	Nebraska	7,248	362	145.00	579.00	167,395.35	23,037.01	558,708.60	387,205.93	45,738.50	2,487,774.09	554,601.27	79,009.08	2,612,033.34
31129	Nuckolls	Nebraska	4,500	243	83.50	400.00	125,293.90	18,168.03	408,037.81	234,910.16	17,624.81	1,829,717.32	360,204.06	41,517.48	2,112,786.30
31131	Otoe	Nebraska	15,740	791	349.00	1,252.50	374,688.90	85,382.71	984,887.72	747,392.93	72,341.32	3,319,599.48	1,122,081.83	172,495.77	3,828,870.51

31133	Pawnee	Nebraska	2,773	148	60.00	234.00	84,416.33	8,394.03	325,330.57	162,405.29	12,544.48	1,141,927.32	246,821.62	23,114.71	1,285,727.90
31135	Perkins	Nebraska	2,970	165	46.00	288.00	81,150.46	5,047.05	336,948.57	149,880.98	10,149.40	815,634.22	231,031.44	17,178.03	1,066,542.04
31137	Phelps	Nebraska	9,188	502	145.50	882.00	234,045.36	28,254.05	684,759.14	460,621.10	35,211.16	2,495,578.36	694,666.46	67,753.23	2,935,465.99
31139	Pierce	Nebraska	7,266	382	146.50	661.50	180,475.88	25,189.44	645,825.83	294,074.63	28,070.77	1,794,570.84	474,550.51	56,792.58	2,161,372.90
31141	Platte	Nebraska	32,237	1,633	528.00	2,682.50	704,890.19	148,922.96	1,655,778.01	1,257,502.02	142,936.69	5,005,346.67	1,962,392.21	347,220.57	6,386,502.54
31143	Polk	Nebraska	5,406	280	108.50	459.00	136,886.60	16,956.94	525,012.29	206,544.75	19,691.81	1,368,384.58	343,431.35	40,877.95	1,666,858.61
31145	Red Willow	Nebraska	11,055	622	185.00	1,035.00	286,204.08	39,755.32	804,582.51	516,994.76	40,224.91	2,613,102.20	803,198.85	88,396.89	3,164,366.96
31147	Richardson	Nebraska	8,363	413	158.00	659.00	229,129.61	30,830.51	800,761.00	415,832.07	32,108.82	2,871,917.07	644,961.68	68,950.51	3,252,738.31
31149	Rock	Nebraska	1,526	67	6.50	127.50	38,191.27	394.12	221,885.37	54,852.43	1,299.06	413,635.14	93,043.70	1,851.11	524,084.49
31151	Saline	Nebraska	14,200	775	312.50	1,219.00	320,607.24	54,965.37	883,606.46	660,398.12	68,705.65	3,405,903.01	981,005.37	131,682.84	3,860,968.85
31153	Sarpy	Nebraska	158,840	7,963	3,404.50	12,704.50	2,733,179.94	1,051,260.16	5,155,191.93	4,543,935.54	1,069,707.34	15,968,715.66	7,277,115.48	2,383,880.21	19,350,632.02
31155	Saunders	Nebraska	20,780	1,046	444.50	1,694.50	445,505.74	110,075.21	1,090,015.64	870,996.72	85,016.24	5,071,708.12	1,316,502.45	218,401.20	5,396,448.64
31157	Scotts Bluff	Nebraska	36,970	1,752	230.50	3,368.00	771,886.71	68,731.74	1,798,393.55	1,345,083.55	61,839.38	6,070,279.99	2,116,970.27	151,586.47	7,010,343.26
31159	Seward	Nebraska	16,750	850	342.50	1,420.00	361,617.67	92,167.28	963,562.36	667,929.71	83,557.88	3,621,558.61	1,029,547.38	194,882.50	4,202,074.15
31161	Sheridan	Nebraska	5,469	261	43.50	511.50	135,192.13	5,295.97	479,773.73	259,768.98	6,639.89	1,895,417.58	394,961.11	13,657.46	2,291,434.06
31163	Sherman	Nebraska	3,152	150	28.00	269.00	92,027.49	3,713.91	374,774.79	183,389.60	4,231.56	1,125,519.42	275,417.09	8,833.19	1,452,080.97
31165	Sioux	Nebraska	1,311	59	8.50	117.00	25,977.63	408.07	122,459.49	48,904.34	992.39	361,097.38	74,881.97	1,412.78	479,449.98
31167	Stanton	Nebraska	6,129	304	100.50	512.00	124,545.30	14,301.78	449,328.88	236,739.75	24,527.33	1,409,137.40	361,285.05	43,933.48	1,650,133.20
31169	Thayer	Nebraska	5,228	308	124.50	501.00	163,623.31	22,466.52	486,391.91	294,178.46	28,536.93	1,610,033.65	457,801.77	55,319.03	1,820,851.31
31171	Thomas	Nebraska	647	28	3.00	59.00	13,386.02	33.23	90,839.19	23,408.72	246.81	163,425.68	36,794.74	408.98	228,963.22
31173	Thurston	Nebraska	6,940	368	108.00	612.50	130,318.56	14,383.26	449,395.86	234,470.49	21,277.65	1,508,091.72	364,789.05	42,804.98	1,852,032.05
31175	Valley	Nebraska	4,260	205	32.50	371.00	103,543.23	3,835.05	455,933.44	183,204.15	5,941.79	1,238,855.23	286,747.37	11,112.35	1,382,808.85
31177	Washington	Nebraska	20,234	993	398.50	1,574.00	430,469.02	92,921.29	1,218,153.80	789,992.69	118,181.75	4,143,029.78	1,220,461.72	229,243.82	4,569,189.16
31179	Wayne	Nebraska	9,595	479	169.00	776.00	208,562.06	28,760.78	706,858.75	331,866.49	29,488.78	2,319,886.90	540,428.54	63,229.16	2,742,372.85
31181	Webster	Nebraska	3,812	204	76.50	342.00	105,518.63	9,887.55	370,851.79	169,508.12	13,416.95	1,282,393.08	275,026.75	25,388.30	1,432,719.64
31183	Wheeler	Nebraska	818	38	8.00	70.50	18,089.42	281.01	122,929.31	41,806.94	1,572.06	241,684.89	59,896.36	1,958.93	345,254.38
31185	York	Nebraska	13,665	735	249.00	1,218.00	349,051.93	62,243.98	894,841.77	687,317.67	59,495.41	3,499,847.49	1,036,369.59	135,390.10	3,904,864.64
32001	Churchill	Nevada	24,877	2,124	1,270.00	2,986.00	933,432.28	381,562.21	2,110,340.97	1,667,912.67	384,176.27	5,706,128.84	2,601,344.95	859,427.24	6,757,500.24
32003	Clark	Nevada	1,951,269	166,772	106,459.00	226,922.00	63,659,390.84	39,482,317.18	87,687,787.85	115,184,681.30	68,286,090.65	174,441,794.80	178,844,072.10	108,441,587.20	256,986,975.90
32005	Douglas	Nevada	46,997	4,120	2,718.00	5,623.00	2,132,141.59	1,079,194.73	3,806,382.61	4,174,179.23	1,182,981.28	11,943,069.01	6,306,320.83	2,492,200.16	14,565,201.95
32007	Elko	Nevada	48,818	3,434	1,745.00	5,189.50	1,227,458.27	461,081.36	2,444,143.89	2,045,746.41	433,606.96	7,773,105.29	3,273,204.68	1,011,531.04	9,020,626.25
32009	Esmeralda	Nevada	783	67	39.00	96.00	39,512.42	3,188.72	193,918.90	65,796.33	8,040.62	539,837.14	105,308.75	12,420.79	693,247.17
32011	Eureka	Nevada	1,987	148	67.50	229.00	63,313.76	6,977.92	278,736.38	132,689.53	27,944.59	801,739.85	196,003.29	38,950.38	946,549.93
32013	Humboldt	Nevada	16,528	1,290	665.00	1,958.00	494,514.28	149,326.51	1,290,170.94	814,918.34	174,829.01	3,505,212.38	1,309,432.62	342,089.21	4,074,375.72
32015	Lander	Nevada	5,775	456	236.00	672.00	178,715.79	36,927.25	533,410.71	378,672.58	73,354.69	1,734,204.66	557,388.36	122,899.25	2,014,166.71
32017	Lincoln	Nevada	5,345	440	232.00	649.00	197,683.53	47,877.90	614,229.96	353,812.42	52,116.79	1,716,750.36	551,495.95	113,332.52	2,049,875.21
32019	Lyon	Nevada	51,980	4,647	3,040.00	6,311.00	2,105,834.63	1,107,827.98	3,800,407.83	3,606,401.91	1,100,490.63	10,687,550.28	5,712,236.55	2,473,324.00	13,168,322.48
32021	Mineral	Nevada	4,772	407	249.50	549.50	222,981.05	60,303.76	662,730.20	420,648.63	66,536.02	2,474,908.85	643,629.68	138,938.47	2,770,182.28
32023	Nye	Nevada	43,946	3,619	2,154.00	5,161.50	1,942,850.02	880,793.43	3,313,909.56	3,820,597.31	1,081,008.35	10,369,383.17	5,763,447.33	2,261,867.17	12,563,161.73
32027	Pershing	Nevada	6,753	516	268.00	784.00	216,742.62	46,537.63	610,914.12	389,899.82	63,363.69	2,048,827.31	606,642.44	126,140.12	2,465,339.77
32029	Storey	Nevada	4,010	338	212.00	457.50	183,415.25	37,805.06	589,126.10	346,608.02	60,766.15	2,214,805.73	530,023.27	103,750.50	2,518,627.73
32031	Washoe	Nevada	421,407	35,031	20,626.50	49,784.50	14,130,170.11	7,766,008.79	20,957,991.85	25,555,920.71	11,621,297.02	46,877,180.10	39,686,090.82	19,978,815.76	66,272,358.69
32033	White Pine	Nevada	10,030	748	346.00	1,158.00	325,622.51	77,020.56	866,786.42	560,950.83	90,683.33	2,755,144.46	886,573.34	183,919.64	3,239,562.10
32510	Carson City	Nevada	55,274	4,857	3,143.50	6,589.00	2,244,612.93	1,117,043.22	4,301,008.34	4,190,175.61	1,426,898.92	12,434,052.17	6,434,788.54	2,762,910.04	14,982,669.74
33001	Belknap	New Hampshire	60,088	2,960	1,106.50	4,927.50	1,393,320.59	372,897.60	2,970,397.43	2,659,272.40	359,679.57	9,132,053.72	4,052,592.99	780,752.93	11,187,886.21
33003	Carroll	New Hampshire	47,818	2,325	672.50	4,012.50	1,240,712.58	245,682.51	2,676,901.92	2,391,600.89	236,658.71	9,204,587.24	3,632,313.27	572,734.67	10,657,253.96
33005	Cheshire	New Hampshire	77,117	4,189	2,003.00	6,432.50	1,903,845.09	693,453.82	3,834,013.08	3,521,858.20	697,435.78	10,930,080.80	5,425,703.29	1,528,189.49	13,855,119.54
33007	Coos	New Hampshire	33,055	1,671	387.50	2,898.50	828,643.20	131,166.19	1,919,771.90	1,592,708.81	99,542.68	6,567,287.63	2,421,352.01	247,706.49	7,949,762.22
33009	Grafton	New Hampshire	89,118	4,477	1,398.50	7,450.50	2,100,196.62	537,691.48	4,351,252.16	4,050,830.71	610,131.24	12,874,149.51	6,151,027.33	1,226,112.21	16,184,800.60
33011	Hillsborough	New Hampshire	400,721	19,992	7,256.50	31,138.00	8,209,058.36	2,776,207.55	13,771,057.31	15,586,329.03	4,451,269.57	36,086,532.52	23,795,387.39	7,818,189.29	46,922,488.04
33013	Merrimack	New Hampshire	146,445	7,346	2,669.00	12,324.00	3,203,123.81	954,266.87	6,266,676.01	5,996,525.08	1,155,821.27	15,688,666.71	9,199,648.89	2,268,135.94	20,851,806.18
33015	Rockingham	New Hampshire	295,223	13,670	4,720.00	22,626.00	5,809,474.96	1,795,659.14	10,268,101.51	10,605,022.05	2,456,567.33	24,799,967.57	16,414,497.01	4,430,280.22	32,601,276.70
33017	Strafford	New Hampshire	123,143	5,790	1,884.50	9,477.50	2,349,386.30	648,992.98	4,613,954.80	4,223,950.45	695,427.64	12,224,984.42	6,573,336.76	1,537,554.60	15,522,491.85
33019	Sullivan	New Hampshire	43,742	2,368	951.00	3,673.00	1,104,682.88	338,855.77	2,357,791.05	2,020,508.36	321,390.28	6,723,195.00	3,125,191.24	686,745.60	8,543,503.65
34001	Atlantic	New Jersey	274,549	23,839	15,062.00	31,968.50	10,213,523.02	5,929,670.09	14,909,829.02	19,173,942.24	8,368,886.88	38,886,949.50	29,387,465.26	15,470,538.31	51,146,888.92
34003	Bergen	New Jersey	905,116	63,818	39,940.50	88,007.00	27,969,676.85	17,362,796.93	39,971,849.88	57,777,452.04	31,838,649.51	95,623,764.37	85,747,128.89	50,373,492.76	130,632,586.40
34005	Burlington	New Jersey	448,734	37,420	24,133.50	50,843.50	15,920,266.75	9,893,091.79	23,055,670.81	30,661,013.13	15,625,658.25	53,840,364.81	46,581,279.88	26,593,020.31	71,746,732.35
34007	Camden	New Jersey	513,657	46,192	30,145.50	61,499.00	18,836,961.30	11,980,500.82	26,352,695.49	34,817,062.04	18,888,347.45	57,238,025.84	53,654,023.34	31,765,215.23	79,528,960.32
34009	Cape May	New Jersey	97,265	8,715	5,747.00	11,801.00	4,598,252.23	2,738,987.69	7,464,043.26	8,567,848.19	3,247,982.72	21,019,633.39	13,166,100.41	6,619,650.25	25,837,790.32
34011	Cumberland	New Jersey	156,898	14,599	9,836.00	19,215.50	5,822,396.74	3,357,184.73	8,667,467.59	10,209,975.21	4,453,200.21	23,073,122.65	16,032,371.95	8,953,961.39	29,585,450.08
34013	Essex	New Jersey	783,969	57,268	35,325.00	78,601.50	22,210,612.69	13,118,040.11	31,647,231.06	43,351,707.79	24,145,431.61	70,328,261.35	65,562,320.47	38,311,882.28	97,381,430.99
34015	Gloucester	New Jersey	288,288	26,506	18,285.00	34,286.50	10,764,489.03	6,666,072.76	15,060,656.88	19,146,651.57	8,907,121.39	38,520,575.65	29,911,140.59	16,332,898.47	50,865,819.72
34017	Hudson	New Jersey	634,266	44,057	27,752.50	60,609.50	16,613,149.81	9,784,425.24	23,794,521.04	34,030,830.74	17,019,226.23	56,559,083.74	50,643,980.55	27,736,167.82	79,111,346.47
34019	Hunterdon	New Jersey	128,349	10,554	6,878.50	14,427.00	4,569,279.38	2,673,543.02	7,644,024.08	8,967,964.39	3,795,005.56	19,027,483.49	13,537,243.77	6,978,490.41	25,446,339.80
34021	Mercer	New Jersey	366,513	29,673	19,221.00	39,894.00	12,050,244.61	7,189,417.43	17,385,176.84	24,405,298.54	12,241,065.10	44,421,555.01	36,455,543.16	20,612,081.86	57,914,428.70
34023	Middlesex	New Jersey	809,858	61,758	39,798.00	83,913.50	24,714,679.51	15,128,600.45	34,670,558.88	49,100,824.53	27,409,233.83	76,515,746.37	73,815,504.05	42,893,077.32	107,645,899.70

34025	Monmouth	New Jersey	630,380	46,937	28,152.00	64,521.50	19,975,494.62	11,726,755.06	28,998,054.37	38,820,671.20	18,620,585.65	68,199,166.47	58,796,165.82	31,699,439.24	92,591,240.23
34027	Morris	New Jersey	492,276	37,309	23,655.50	50,470.50	15,909,726.95	9,614,177.77	22,596,999.92	34,701,956.95	18,356,321.96	62,192,441.38	50,611,683.90	29,344,705.10	82,390,197.98
34029	Ocean	New Jersey	576,567	46,899	26,878.50	65,737.00	22,511,262.79	12,843,208.30	33,287,445.27	44,089,772.41	20,821,757.56	74,363,261.01	66,601,035.20	34,898,506.61	103,247,226.30
34031	Passaic	New Jersey	501,226	37,376	24,168.00	51,613.50	14,675,557.97	8,885,021.49	21,689,015.94	27,682,502.10	13,640,233.70	50,909,972.13	42,358,060.07	22,822,046.43	67,243,411.35
34033	Salem	New Jersey	66,083	6,281	4,360.50	8,288.50	2,774,115.27	1,535,275.90	4,905,019.38	5,341,493.68	1,991,550.06	14,486,552.28	8,115,608.95	3,865,238.72	17,306,795.93
34035	Somerset	New Jersey	323,444	25,672	15,635.00	34,805.00	10,367,158.50	6,067,471.23	15,210,639.52	22,978,450.85	11,596,132.13	43,811,571.70	33,345,609.35	18,217,498.13	55,232,686.54
34037	Sussex	New Jersey	149,265	11,507	7,105.00	15,482.00	4,818,806.19	2,646,553.43	7,743,783.73	8,365,884.03	3,095,081.28	19,287,174.43	13,184,690.22	6,131,291.71	24,945,954.47
34039	Union	New Jersey	536,499	40,442	26,597.50	55,294.00	16,231,862.79	10,053,406.30	24,010,490.46	32,632,015.48	17,737,492.12	55,044,937.48	48,863,878.27	28,083,943.67	76,475,344.36
34041	Warren	New Jersey	108,692	8,895	5,747.50	12,016.50	3,842,239.43	2,121,820.63	6,225,431.44	7,170,376.60	2,703,106.68	17,648,361.43	11,012,616.03	5,308,611.99	22,574,031.59
35001	Bernalillo	New Mexico	662,564	73,950	54,049.00	95,049.00	29,797,314.82	21,199,936.46	38,975,588.38	52,812,623.57	32,548,320.89	84,367,307.22	82,609,938.39	55,971,115.92	118,717,582.30
35003	Catron	New Mexico	3,725	389	240.50	534.00	252,553.39	65,898.78	709,970.67	430,997.64	56,873.87	2,341,774.27	683,551.03	132,960.04	2,782,913.70
35005	Chaves	New Mexico	65,645	8,568	6,112.50	11,028.00	3,486,945.72	2,075,337.13	5,841,110.30	5,566,018.63	1,928,536.76	15,129,050.13	9,052,964.35	4,469,979.87	19,020,951.77
35006	Cibola	New Mexico	27,213	2,844	1,937.00	3,778.00	1,127,408.14	566,556.70	2,218,779.00	1,720,858.96	463,523.41	6,054,727.32	2,848,267.11	1,137,132.18	7,404,266.36
35007	Colfax	New Mexico	13,750	1,244	730.00	1,720.00	616,459.35	223,902.58	1,409,486.08	1,234,866.61	195,612.67	4,933,740.37	1,851,325.96	486,460.48	5,740,766.19
35009	Curry	New Mexico	48,376	6,154	4,410.00	7,845.00	2,277,358.14	1,286,015.65	4,002,864.86	3,865,409.83	1,219,985.36	11,804,211.59	6,142,767.97	2,778,585.46	14,041,264.76
35011	De Baca	New Mexico	2,022	245	172.50	324.00	119,749.75	25,365.71	382,210.04	217,004.75	31,188.09	1,383,343.44	336,754.50	62,059.45	1,562,594.79
35013	Dona Ana	New Mexico	209,233	24,510	16,056.00	33,219.50	9,445,184.01	5,765,467.96	13,759,958.98	16,141,240.05	7,090,596.52	33,193,027.38	25,586,424.06	13,786,423.94	44,188,164.47
35015	Eddy	New Mexico	53,829	7,014	4,839.00	9,179.00	2,943,447.52	1,680,902.95	4,972,360.66	5,431,021.06	2,061,852.46	15,001,497.38	8,374,468.58	4,069,129.66	18,134,630.28
35017	Grant	New Mexico	29,514	3,225	2,060.00	4,374.50	1,645,865.28	785,446.88	3,005,395.32	3,220,920.24	816,266.84	10,291,383.13	4,866,785.51	1,771,127.11	12,141,207.02
35019	Guadalupe	New Mexico	4,687	536	379.00	702.50	237,204.76	65,986.15	667,547.46	341,190.55	60,014.23	1,987,251.00	578,395.31	136,459.32	2,368,590.96
35021	Harding	New Mexico	695	70	41.00	98.00	47,659.49	3,980.96	251,967.05	88,928.27	8,805.57	857,938.67	136,587.76	14,145.56	902,891.51
35023	Hidalgo	New Mexico	4,894	542	348.50	752.00	240,319.35	64,857.27	646,953.98	398,288.87	78,328.46	2,038,692.80	638,608.22	157,145.15	2,372,946.93
35025	Lea	New Mexico	64,727	8,979	6,599.50	11,444.50	3,334,098.59	2,003,818.76	5,209,194.70	5,796,419.11	2,371,556.56	14,296,553.26	9,130,517.70	4,682,250.87	18,387,637.30
35027	Lincoln	New Mexico	20,497	2,417	1,683.50	3,147.00	1,311,922.25	653,485.96	2,443,643.25	2,347,986.72	513,405.33	7,857,965.25	3,659,908.97	1,348,972.88	9,331,241.52
35028	Los Alamos	New Mexico	17,950	1,804	1,182.50	2,389.00	821,507.01	351,708.54	1,756,388.19	1,919,891.68	615,259.50	6,868,158.26	2,741,398.69	1,034,584.77	7,835,522.90
35029	Luna	New Mexico	25,095	2,901	1,852.00	3,962.50	1,315,911.90	594,164.96	2,483,779.81	2,380,296.98	591,917.29	7,741,588.00	3,696,208.88	1,340,821.03	9,532,856.61
35031	McKinley	New Mexico	71,492	7,377	4,768.50	10,321.00	2,575,186.24	1,326,089.08	4,437,593.82	4,061,781.05	1,177,631.95	12,721,259.48	6,636,967.29	2,776,201.29	15,772,126.92
35033	Mora	New Mexico	4,881	482	314.50	661.00	242,642.59	64,915.73	682,294.99	431,883.14	62,351.84	2,327,939.85	674,525.74	142,090.89	2,915,320.83
35035	Otero	New Mexico	63,797	7,882	5,402.50	10,258.00	3,317,398.15	1,863,438.02	5,277,447.61	5,711,557.92	2,082,923.80	16,104,068.98	9,028,956.07	4,336,496.24	19,555,565.74
35037	Quay	New Mexico	9,041	1,050	746.00	1,362.00	544,560.48	201,658.27	1,416,398.39	951,570.14	170,982.50	4,020,244.83	1,496,130.63	401,249.74	4,750,087.53
35039	Rio Arriba	New Mexico	40,246	3,757	2,402.00	5,104.00	1,591,376.56	791,330.55	2,839,813.60	2,725,202.39	628,601.07	8,755,063.62	4,316,578.95	1,625,454.15	10,747,383.02
35041	Roosevelt	New Mexico	19,846	2,586	1,846.50	3,280.50	952,241.77	474,189.17	1,890,448.43	1,468,408.58	381,490.50	5,999,065.87	2,420,650.35	953,621.38	6,936,634.92
35043	Sandoval	New Mexico	131,561	13,804	9,268.50	18,134.50	5,506,278.30	3,241,592.94	8,650,723.77	9,793,151.37	4,154,795.14	23,381,064.97	15,299,429.67	7,982,407.95	30,024,019.41
35045	San Juan	New Mexico	130,044	12,309	7,672.50	17,189.50	4,608,668.81	2,478,371.20	7,502,319.78	7,853,020.91	3,040,311.46	18,154,297.41	12,461,689.72	6,036,215.18	24,439,106.10
35047	San Miguel	New Mexico	29,393	3,086	2,142.50	4,134.50	1,399,935.17	666,920.22	2,607,482.61	2,338,000.34	573,011.20	7,814,581.43	3,737,935.51	1,373,241.96	10,005,495.87
35049	Santa Fe	New Mexico	144,170	15,040	10,083.00	19,959.50	6,993,475.98	4,174,337.13	10,764,666.69	12,603,928.87	5,374,260.73	26,770,349.62	19,597,404.85	10,219,847.55	34,851,778.62
35051	Sierra	New Mexico	11,988	1,349	881.50	1,832.50	863,400.17	350,404.73	1,883,202.51	1,782,536.36	289,791.48	6,625,340.96	2,645,936.53	760,956.38	7,603,990.76
35053	Socorro	New Mexico	17,866	2,035	1,392.50	2,651.50	847,985.34	385,223.57	1,757,025.69	1,522,327.97	345,646.24	6,619,952.93	2,370,313.32	816,551.82	7,448,448.58
35055	Taos	New Mexico	32,937	2,974	1,854.50	4,115.00	1,473,076.03	649,155.22	2,740,509.34	2,693,212.92	513,346.07	9,461,682.68	4,166,288.95	1,372,073.19	11,040,400.59
35057	Torrance	New Mexico	16,383	1,859	1,265.50	2,391.00	836,612.67	358,009.75	1,785,735.61	1,345,308.00	288,052.54	5,517,415.77	2,181,920.67	701,711.16	6,605,468.72
35059	Union	New Mexico	4,549	419	256.00	569.00	204,826.22	43,288.32	615,202.17	311,077.25	47,907.06	1,816,553.57	515,903.47	96,400.86	2,212,627.57
35061	Valencia	New Mexico	76,569	8,683	6,197.00	11,192.50	3,526,225.47	2,061,947.08	5,786,034.21	5,888,932.98	2,028,739.95	14,475,335.85	9,415,158.45	4,593,024.61	18,970,784.87
36001	Albany	New York	304,204	22,273	14,179.00	30,589.00	9,644,668.11	5,632,140.27	14,240,475.07	18,286,309.08	8,384,025.66	34,337,598.79	27,930,977.19	14,807,995.15	45,708,437.84
36003	Allegany	New York	48,946	4,153	2,677.50	5,544.00	1,840,613.27	921,884.80	3,498,500.43	3,276,008.57	830,974.24	10,887,466.19	5,116,621.84	1,941,353.12	13,779,108.89
36005	Bronx	New York	1,385,108	100,356	62,113.50	139,719.00	36,652,999.36	21,932,408.70	52,263,554.15	65,644,494.28	34,601,822.54	105,158,488.10	102,297,493.60	56,726,152.74	151,437,861.40
36007	Broome	New York	200,600	16,554	10,718.00	22,141.00	7,518,831.19	4,396,635.67	11,567,297.90	13,796,899.62	5,730,642.75	28,028,339.19	21,315,730.81	10,424,095.62	36,385,897.87
36009	Cattaraugus	New York	80,317	7,023	4,610.00	9,470.00	3,152,677.58	1,741,468.25	5,130,857.79	5,610,994.04	1,802,570.72	15,734,750.34	8,763,671.61	3,952,191.24	18,935,213.87
36011	Cayuga	New York	80,026	6,576	4,233.50	8,753.50	2,929,362.62	1,605,588.24	4,924,986.97	5,273,245.50	1,703,363.70	14,720,886.42	8,202,608.12	3,668,444.29	17,648,246.40
36013	Chautauqua	New York	134,905	11,698	7,681.00	15,526.00	5,355,671.41	3,106,296.82	8,521,478.18	9,471,719.19	3,658,715.84	20,717,862.02	14,827,390.59	7,354,661.03	27,150,498.03
36015	Chemung	New York	88,830	7,749	5,193.00	10,406.50	3,422,092.53	1,913,692.69	5,604,756.95	6,412,922.22	2,276,383.92	16,652,958.52	9,835,014.75	4,558,431.30	20,340,434.48
36017	Chenango	New York	50,477	4,129	2,674.50	5,643.50	1,903,370.14	927,416.50	3,324,588.59	3,468,218.27	944,222.56	10,667,980.58	5,371,588.41	1,974,571.39	12,730,131.04
36019	Clinton	New York	82,128	5,098	2,183.50	8,201.00	2,166,535.54	761,158.76	4,033,893.53	3,962,445.64	703,232.00	12,383,598.48	6,128,981.18	1,638,337.77	15,426,028.64
36021	Columbia	New York	63,096	4,550	2,803.00	6,343.00	2,249,259.19	1,033,636.37	4,068,574.89	4,314,094.57	1,036,100.70	13,521,068.69	6,563,353.75	2,408,946.91	15,951,389.24
36023	Cortland	New York	49,336	4,071	2,634.50	5,485.50	1,705,674.33	803,968.67	3,211,235.23	2,830,485.31	746,224.80	8,570,494.20	4,536,159.64	1,732,500.07	10,309,895.15
36025	Delaware	New York	47,980	3,718	2,307.00	5,107.00	1,873,910.54	879,991.45	3,426,285.28	3,403,758.91	899,329.80	10,789,147.04	5,277,669.46	2,039,837.23	12,801,060.09
36027	Dutchess	New York	297,488	21,258	12,919.50	29,645.50	9,031,042.35	4,934,726.78	13,416,343.34	17,449,751.43	7,362,650.85	36,543,665.22	26,480,793.78	12,579,375.31	46,026,027.96
36029	Erie	New York	919,040	74,171	48,438.50	99,959.00	32,964,008.95	20,826,911.30	46,074,064.34	61,602,170.16	33,193,963.76	96,822,419.08	94,566,179.11	55,081,099.52	137,986,829.20
36031	Essex	New York	39,370	2,627	1,299.00	3,923.00	1,281,071.90	458,045.23	2,680,840.34	2,318,602.03	431,273.19	9,003,624.73	3,599,673.92	936,258.56	10,537,253.13
36033	Franklin	New York	51,599	3,554	1,871.50	5,370.50	1,501,554.15	582,865.15	3,105,961.78	2,665,461.35	614,609.35	9,123,577.18	4,167,015.50	1,324,450.69	11,406,790.25
36035	Fulton	New York	55,531	4,139	2,478.50	5,834.00	1,867,310.56	846,872.73	3,539,278.30	3,196,683.79	843,800.37	9,358,092.05	5,063,994.35	1,949,793.91	11,547,251.49
36037	Genesee	New York	60,079	4,767	3,144.00	6,523.50	2,128,358.25	1,074,585.67	3,873,205.02	3,725,541.75	1,034,723.85	10,894,734.36	5,853,900.00	2,371,983.78	13,371,016.91
36039	Greene	New York	49,221	3,617	2,182.00	4,960.00	1,727,648.37	825,573.95	3,243,883.45	3,122,582.53	799,595.52	11,037,337.17	4,850,230.90	1,852,459.42	12,863,739.50
36041	Hamilton	New York	4,836	349	193.50	513.50	200,053.46	39,547.50	615,700.24	401,446.59	43,370.82	2,331,022.67	601,500.05	95,076.03	2,755,398.86
36043	Herkimer	New York	64,519	4,883	2,936.50	6,834.50	2,206,002.27	1,072,019.13	3,721,214.71	3,775,903.72	911,285.74	11,017,386.28	5,981,905.98	2,204,760.88	13,944,990.83
36045	Jefferson	New York	116,229	9,247	5,507.00	12,934.50	3,473,202.28	1,728,805.43	5,603,039.83	6,090,110.31	2,016,423.90	15,602,668.83	9,563,312.59	4,116,993.17	19,928,262.93
36047	Kings	New York	2,504,700	179,423	110,745.00	248,727.00	68,987,456.76	42,134,574.57	95,878,197.29	120,411,889.40	65,031,565.08	178,769,583.60	189,399,346.10	108,457,825.40	271,102,935.60

36049	Lewis	New York	27,087	2,137	1,204.00	3,062.00	930,092.19	357,498.25	1,997,405.39	1,565,283.02	305,730.26	5,698,689.46	2,495,375.21	785,249.85	7,032,673.85
36051	Livingston	New York	65,393	5,244	3,374.50	7,020.00	2,288,804.91	1,139,865.62	4,111,277.20	3,877,348.79	1,117,387.44	10,982,382.37	6,166,153.70	2,582,926.24	14,003,783.03
36053	Madison	New York	73,442	5,845	3,788.50	7,919.00	2,584,951.30	1,289,341.89	4,624,120.25	4,343,795.70	1,224,663.85	12,235,200.82	6,928,747.00	2,840,709.45	15,058,549.34
36055	Monroe	New York	744,344	57,770	38,144.00	78,253.50	24,446,248.04	15,859,273.41	34,426,753.03	45,720,968.90	25,463,227.82	76,564,757.91	70,167,216.94	42,448,137.30	109,247,243.20
36057	Montgomery	New York	50,219	3,866	2,250.50	5,343.00	1,762,777.52	792,894.66	3,500,528.59	3,098,675.11	779,777.53	10,480,479.56	4,861,452.63	1,826,340.59	12,577,537.74
36059	Nassau	New York	1,339,532	91,963	56,073.00	131,548.00	40,413,494.50	23,105,631.80	58,634,350.46	80,557,244.54	41,329,807.41	128,150,044.90	120,970,739.00	67,519,090.08	180,932,613.80
36061	New York	New York	1,585,873	104,424	66,692.50	142,420.00	44,414,776.62	28,517,708.28	62,494,298.23	110,929,896.70	65,747,093.46	165,709,729.70	155,344,673.40	93,217,327.02	223,455,452.70
36063	Niagara	New York	216,469	16,483	10,407.00	22,746.50	7,528,608.97	4,279,219.75	11,572,740.44	13,687,900.96	5,155,276.44	30,611,668.09	21,216,509.94	10,156,312.60	38,944,116.85
36065	Oneida	New York	234,878	18,225	10,898.00	24,951.00	8,191,046.38	4,558,381.86	12,575,927.58	14,818,153.51	6,200,528.28	30,401,525.38	23,009,199.90	11,380,348.68	40,754,884.05
36067	Onondaga	New York	467,026	38,349	25,611.50	51,624.50	16,159,127.44	10,225,976.99	22,543,123.21	30,308,972.21	15,646,285.53	56,132,580.91	46,468,099.66	27,056,675.27	75,180,152.12
36069	Ontario	New York	107,931	8,774	5,487.00	12,160.50	3,957,403.04	2,187,872.30	6,247,787.01	7,382,248.52	2,542,750.60	18,421,298.90	11,339,651.56	5,105,282.21	23,050,394.07
36071	Orange	New York	372,813	28,082	17,587.50	39,000.00	10,698,987.72	6,313,164.40	15,919,580.25	18,301,006.29	8,023,838.67	37,250,092.02	28,999,994.00	14,980,774.45	50,454,216.54
36073	Orleans	New York	42,883	3,288	1,991.50	4,519.00	1,410,605.96	647,633.14	2,703,314.34	2,628,944.27	580,914.52	9,987,864.44	4,039,550.24	1,314,821.27	12,357,866.91
36075	Oswego	New York	122,109	9,778	6,210.00	13,107.00	3,998,515.02	2,164,366.05	6,278,545.93	6,956,484.19	2,501,662.93	16,865,806.15	10,954,999.21	5,146,576.77	21,285,565.12
36077	Otsego	New York	62,259	4,678	2,814.50	6,490.50	2,162,987.25	1,018,350.90	3,926,440.36	4,066,325.17	1,054,690.80	12,801,965.08	6,229,312.41	2,272,635.42	15,034,100.29
36079	Putnam	New York	99,710	7,061	4,143.00	10,023.50	2,947,715.30	1,443,895.40	5,040,340.75	5,252,842.37	1,615,708.05	13,670,102.92	8,200,557.67	3,397,151.61	17,547,762.68
36081	Queens	New York	2,230,722	153,473	98,663.00	211,546.00	63,306,270.27	39,918,493.69	88,512,437.16	117,252,100.90	67,416,231.15	175,677,662.40	180,558,371.20	109,413,342.70	260,916,838.10
36083	Rensselaer	New York	159,429	11,159	6,640.00	15,987.00	4,800,069.88	2,535,935.54	7,874,684.38	8,741,981.31	3,277,834.15	20,919,879.62	13,542,051.19	6,271,479.24	27,855,355.42
36085	Richmond	New York	468,730	33,747	21,467.50	47,159.50	13,797,811.84	8,532,034.45	20,106,524.11	24,944,465.80	11,366,872.68	45,930,555.75	38,742,277.64	21,263,671.74	63,532,498.40
36087	Rockland	New York	311,687	22,829	13,769.50	31,671.00	9,241,749.24	4,994,310.10	14,053,632.69	17,502,112.82	7,434,187.82	35,448,278.16	26,743,862.06	13,743,577.13	47,116,253.65
36089	St. Lawrence	New York	111,944	8,004	4,138.00	11,659.00	3,409,044.99	1,547,509.10	5,820,663.56	6,070,534.78	1,588,905.24	16,317,501.44	9,479,579.77	3,557,502.38	19,929,246.19
36091	Saratoga	New York	219,607	15,989	9,528.00	22,336.00	6,934,170.63	3,708,552.84	11,081,304.90	12,252,113.63	4,793,056.53	27,398,845.86	19,186,284.27	9,119,423.31	36,457,495.02
36093	Schenectady	New York	154,727	11,770	7,099.00	16,281.50	5,122,482.96	2,711,037.98	8,171,732.13	9,984,601.14	4,023,721.50	22,187,732.24	15,107,084.10	7,161,089.21	29,334,123.95
36095	Schoharie	New York	32,749	2,428	1,506.50	3,391.00	1,109,635.37	493,912.18	2,092,235.42	1,938,541.24	454,538.39	7,057,184.35	3,048,176.61	1,074,607.63	8,501,867.12
36097	Schuyler	New York	18,343	1,547	1,018.50	2,073.50	746,579.24	278,971.07	1,674,264.84	1,220,695.21	248,250.14	5,124,428.57	1,967,274.44	609,636.01	6,153,766.37
36099	Seneca	New York	35,251	2,918	1,885.50	3,924.00	1,329,228.16	609,078.28	2,568,584.57	2,394,319.17	565,342.43	8,919,148.45	3,723,547.34	1,317,190.30	10,259,784.64
36101	Steuben	New York	98,990	8,552	5,719.00	11,231.50	3,843,359.68	2,279,624.42	6,080,866.99	7,202,336.42	2,896,516.12	18,099,390.75	11,045,696.11	5,684,391.36	22,406,829.43
36103	Suffolk	New York	1,493,350	93,340	45,102.50	140,182.00	38,873,752.90	18,092,623.81	58,277,110.96	76,547,519.99	33,358,070.97	132,269,885.60	115,421,272.90	52,639,214.50	188,425,357.80
36105	Sullivan	New York	77,547	6,028	3,689.50	8,256.50	2,638,434.34	1,328,606.56	4,518,226.74	4,789,687.97	1,453,037.92	14,763,677.14	7,428,122.32	3,099,235.71	17,672,639.18
36107	Tioga	New York	51,125	4,422	2,881.50	5,872.00	1,991,553.96	1,002,477.48	3,496,597.26	3,720,255.78	1,230,742.91	10,739,597.99	5,711,809.74	2,417,999.47	12,819,202.29
36109	Tompkins	New York	101,564	8,045	5,215.00	10,692.00	3,177,863.52	1,693,136.79	5,286,599.78	5,442,742.67	1,796,752.76	15,672,324.94	8,620,606.20	3,762,652.42	19,295,736.24
36111	Ulster	New York	182,493	13,191	7,943.00	18,356.00	5,953,658.51	3,251,712.23	9,404,550.60	10,375,958.02	3,974,568.68	23,050,814.95	16,329,616.52	7,912,046.75	29,943,987.51
36113	Warren	New York	65,707	4,569	2,557.50	6,516.50	2,218,747.29	987,839.29	4,191,522.95	3,921,940.00	1,002,589.21	11,462,688.60	6,140,687.28	2,238,274.77	14,401,028.36
36115	Washington	New York	63,216	4,293	2,207.00	6,231.50	1,949,991.10	742,941.63	3,656,103.69	3,373,523.61	796,707.94	10,267,619.76	5,323,514.71	1,729,368.28	13,282,137.02
36117	Wayne	New York	93,772	7,594	4,867.00	10,410.50	3,311,152.95	1,738,051.29	5,645,994.64	5,926,890.51	1,816,264.10	15,684,256.64	9,238,043.46	3,869,649.81	20,038,387.27
36119	Westchester	New York	949,113	67,155	37,620.50	94,498.50	28,834,595.75	15,281,478.75	42,299,336.94	60,570,636.73	29,887,086.21	96,331,526.31	89,405,232.48	46,137,929.14	135,104,195.80
36121	Wyoming	New York	42,155	3,328	2,159.00	4,502.50	1,440,533.97	659,320.74	2,746,161.78	2,419,375.61	596,398.93	7,571,839.00	3,859,909.58	1,403,401.54	9,541,010.46
36123	Yates	New York	25,348	2,162	1,448.50	2,973.00	965,437.14	422,098.30	1,938,872.09	1,618,769.23	338,609.92	6,735,259.51	2,584,206.38	867,903.29	7,678,091.81
37001	Alamance	North Carolina	151,131	12,038	8,112.50	16,028.50	5,080,676.71	2,931,631.77	8,009,123.35	8,966,514.51	3,467,680.87	21,580,520.39	14,047,191.21	7,067,542.41	26,303,225.16
37003	Alexander	North Carolina	37,198	2,673	1,576.00	3,704.50	1,157,877.03	485,668.22	2,284,883.84	1,944,702.30	385,545.74	7,270,186.13	3,102,579.34	995,104.04	9,010,417.01
37005	Alleghany	North Carolina	11,155	811	460.50	1,141.50	416,480.22	130,130.00	992,835.95	732,010.54	91,883.12	4,127,378.11	1,148,490.76	255,493.11	4,457,406.62
37007	Anson	North Carolina	26,948	1,966	1,149.00	2,793.50	844,696.82	310,751.90	1,980,304.85	1,530,095.18	276,895.55	6,796,392.94	2,374,791.99	673,452.79	7,700,584.66
37009	Ashe	North Carolina	27,281	1,957	1,183.50	2,779.00	965,387.43	399,338.95	1,869,345.45	1,621,993.68	297,259.08	5,574,732.24	2,587,381.11	796,860.39	6,773,682.83
37011	Avery	North Carolina	17,797	1,255	736.00	1,825.50	594,339.87	204,622.17	1,271,086.06	1,090,821.43	151,134.25	4,884,986.39	1,685,161.30	418,845.15	5,871,687.53
37013	Beaufort	North Carolina	47,759	4,611	3,087.50	6,111.00	2,217,038.13	1,200,540.96	3,828,785.81	4,011,471.04	1,177,996.18	12,211,309.29	6,228,509.16	2,649,689.76	14,381,290.82
37015	Bertie	North Carolina	21,282	2,095	1,454.50	2,741.00	999,488.52	443,384.58	2,177,164.45	1,728,017.50	372,407.31	6,741,401.07	2,727,506.01	962,286.74	8,087,424.68
37017	Bladen	North Carolina	35,190	2,864	1,850.50	3,927.00	1,346,415.44	562,153.13	2,969,442.22	2,205,597.13	482,410.64	7,600,335.43	3,552,012.57	1,129,621.90	9,409,355.60
37019	Brunswick	North Carolina	107,431	8,464	4,928.50	12,078.50	4,462,607.13	2,203,642.45	7,510,247.33	8,449,421.11	2,945,902.87	20,625,729.60	12,912,028.24	5,706,041.82	26,507,190.00
37021	Buncombe	North Carolina	238,318	17,569	10,545.50	24,196.50	7,970,985.63	4,420,150.16	12,035,006.55	14,310,038.82	6,002,556.01	30,490,814.13	22,281,024.45	10,856,266.76	39,852,540.78
37023	Burke	North Carolina	90,912	6,602	3,966.50	9,308.50	2,949,811.42	1,496,120.74	5,100,198.16	5,140,474.39	1,592,437.91	13,414,437.27	8,090,285.82	3,521,131.68	17,128,173.43
37025	Cabarrus	North Carolina	178,011	13,184	7,818.00	18,424.50	5,000,406.95	2,701,320.53	8,525,130.42	8,140,656.56	3,054,508.68	18,443,706.67	13,141,063.50	6,264,264.81	25,247,324.53
37027	Caldwell	North Carolina	83,029	6,023	3,685.00	8,619.00	2,618,480.78	1,282,290.93	4,354,265.72	4,764,320.18	1,254,427.60	14,426,588.86	7,382,800.96	2,912,819.74	17,235,548.97
37029	Camden	North Carolina	9,980	986	675.00	1,297.00	403,855.30	137,929.46	1,012,160.51	714,673.52	136,278.19	3,098,505.80	1,118,528.81	294,534.28	3,545,662.17
37031	Carteret	North Carolina	66,469	6,036	3,763.50	8,439.00	3,049,349.08	1,552,693.62	5,360,514.33	5,388,976.40	1,575,119.19	14,888,992.62	8,438,325.48	3,505,084.86	18,565,150.44
37033	Caswell	North Carolina	23,719	1,901	1,230.00	2,512.50	896,094.57	371,707.16	1,957,366.02	1,433,761.31	284,771.19	5,082,550.93	2,329,855.88	750,007.26	6,429,640.14
37035	Catawba	North Carolina	154,358	11,159	6,331.00	15,593.00	4,777,552.26	2,435,227.06	7,799,790.43	8,325,130.67	2,812,727.99	20,204,485.02	13,102,682.93	5,445,001.86	26,180,997.01
37037	Chatham	North Carolina	63,505	5,053	3,223.00	6,744.50	2,400,595.59	1,208,946.24	4,050,249.84	4,247,825.78	1,248,396.66	11,875,077.37	6,648,421.37	2,884,306.07	15,580,619.52
37039	Cherokee	North Carolina	27,444	1,969	1,172.50	2,740.50	1,055,682.72	442,561.18	2,141,753.34	1,958,338.73	362,170.54	6,815,196.10	3,014,021.46	966,534.56	8,227,068.69
37041	Chowan	North Carolina	14,793	1,475	978.50	1,944.00	735,744.92	306,028.68	1,698,248.90	1,236,284.68	259,390.72	4,944,751.24	1,972,029.60	664,301.13	5,965,146.13
37043	Clay	North Carolina	10,587	758	459.00	1,063.00	421,741.39	126,703.14	1,023,087.73	853,669.40	102,516.71	3,615,857.52	1,275,410.78	266,936.29	4,299,681.29
37045	Cleveland	North Carolina	98,078	7,116	4,189.00	10,250.50	3,134,342.90	1,633,146.54	5,188,467.54	5,443,558.10	1,808,366.78	14,151,779.11	8,577,901.00	3,595,645.94	18,166,365.80
37047	Columbus	North Carolina	58,098	4,583	2,637.50	6,444.00	1,999,400.42	962,834.44	3,589,180.34	3,645,191.95	858,721.11	10,034,338.59	5,644,592.77	2,060,519.24	11,998,858.88
37049	Craven	North Carolina	103,505	9,751	6,256.50	13,190.00	4,144,901.14	2,317,410.09	6,360,760.87	7,755,491.78	2,800,904.74	19,364,289.28	11,900,392.92	5,609,118.45	24,323,827.74
37051	Cumberland	North Carolina	319,431	26,497	16,708.00	36,561.00	9,459,662.72	5,523,514.99	14,003,922.07	16,379,694.66	7,496,746.31	31,933,053.95	25,839,357.38	13,745,530.06	43,655,370.53
37053	Currituck	North Carolina	23,547	2,267	1,566.00	3,001.00	959,866.33	435,815.28	1,994,835.98	1,602,567.48	326,971.00	6,252,642.69	2,562,433.81	834,916.50	7,612,790.40
37055	Dare	North Carolina	33,920	3,134	2,011.00	4,346.00	1,465,960.68	666,867.23	2,820,386.55	2,348,649.10	546,331.13	7,909,936.92	3,814,609.78	1,353,910.98	9,275,571.86

37057	Davidson	North Carolina	162,878	12,191	7,361.00	17,357.00	5,245,231.97	2,741,533.70	8,400,087.28	8,951,480.90	3,163,097.18	21,518,607.27	14,196,712.87	6,384,301.32	27,906,711.84
37059	Davie	North Carolina	41,240	3,049	1,758.50	4,264.50	1,391,301.98	620,188.37	2,679,646.27	2,465,079.33	545,963.63	7,546,066.17	3,856,381.31	1,346,575.00	9,275,977.64
37061	Duplin	North Carolina	58,505	5,194	3,394.00	7,027.50	2,146,428.43	1,065,506.58	3,767,930.39	3,780,355.69	951,391.68	12,277,901.23	5,926,784.12	2,308,838.12	14,655,017.00
37063	Durham	North Carolina	267,587	22,301	15,831.50	29,437.00	8,203,831.51	5,253,374.90	12,019,491.35	16,250,455.20	8,228,001.67	31,682,369.85	24,454,286.71	14,003,200.58	42,214,398.41
37065	Edgecombe	North Carolina	56,552	5,325	3,524.00	7,016.00	2,285,036.93	1,208,037.01	4,055,660.26	3,991,635.84	1,260,443.94	12,725,601.67	6,276,672.77	2,697,547.48	15,521,417.82
37067	Forsyth	North Carolina	350,670	26,483	16,468.50	36,592.50	10,857,002.80	6,315,587.79	16,389,989.14	19,594,043.56	8,863,640.90	38,510,340.54	30,451,046.36	15,742,436.32	51,629,752.16
37069	Franklin	North Carolina	60,619	5,435	3,672.50	7,327.50	2,191,308.01	1,169,583.49	3,716,147.47	3,563,341.25	1,095,652.68	9,975,568.69	5,754,649.26	2,513,282.65	12,873,154.34
37071	Gaston	North Carolina	206,086	14,737	9,050.00	20,523.50	6,097,545.72	3,463,667.75	9,607,153.16	10,980,614.91	3,922,852.13	24,836,350.68	17,078,160.63	7,700,947.51	31,759,031.92
37073	Gates	North Carolina	12,197	1,218	861.50	1,576.50	542,087.67	204,305.02	1,290,153.10	868,740.64	176,993.64	3,529,862.38	1,410,828.30	428,789.51	4,205,920.63
37075	Graham	North Carolina	8,861	649	386.50	912.00	326,903.30	90,156.48	788,308.42	555,516.05	77,257.96	2,602,016.60	882,419.34	189,304.11	3,180,463.46
37077	Granville	North Carolina	59,916	5,136	3,574.00	6,826.00	2,104,495.23	1,156,517.30	3,662,892.91	3,786,797.88	1,228,263.40	11,629,383.42	5,891,293.11	2,563,418.05	13,976,435.35
37079	Greene	North Carolina	21,362	1,931	1,278.50	2,588.00	798,524.62	326,060.63	1,834,241.70	1,356,294.82	242,823.77	6,076,763.25	2,154,819.44	624,531.28	6,918,409.45
37081	Guilford	North Carolina	488,406	37,195	24,098.50	49,894.00	14,897,322.45	9,144,670.13	20,821,581.87	26,846,610.34	13,213,373.14	50,147,199.32	41,743,932.79	23,873,357.53	66,593,295.14
37083	Halifax	North Carolina	54,691	5,269	3,630.50	7,021.00	2,373,188.97	1,185,398.61	4,130,273.34	4,229,515.70	1,216,675.05	12,138,345.01	6,602,704.67	2,660,532.62	14,937,461.15
37085	Harnett	North Carolina	114,678	9,707	6,226.50	13,014.50	3,517,326.69	1,906,132.03	5,518,701.60	5,648,049.11	1,789,504.20	14,348,026.17	9,165,375.80	4,150,935.46	18,077,311.17
37087	Haywood	North Carolina	59,036	4,412	2,809.50	6,015.50	2,256,282.21	1,162,805.15	3,830,716.31	4,233,855.12	1,208,311.69	11,290,531.69	6,490,137.33	2,791,238.28	13,924,676.28
37089	Henderson	North Carolina	106,740	7,984	4,815.00	11,037.00	4,118,216.30	2,142,070.58	6,860,412.89	7,559,145.58	2,547,778.97	18,809,866.36	11,677,361.89	4,968,468.40	24,099,659.84
37091	Hertford	North Carolina	24,669	2,453	1,674.50	3,198.50	1,124,287.36	494,920.34	2,199,116.49	1,923,464.97	425,015.42	6,954,853.51	3,047,752.33	1,082,340.03	8,387,305.27
37093	Hoke	North Carolina	46,952	3,880	2,410.00	5,398.50	1,274,793.23	578,272.77	2,449,344.58	1,848,357.36	450,030.58	7,138,764.23	3,123,150.60	1,145,953.56	8,500,127.39
37095	Hyde	North Carolina	5,810	539	347.00	751.00	252,938.14	60,903.31	735,040.73	423,241.34	64,840.06	2,117,274.52	676,179.48	133,701.13	2,563,359.42
37097	Iredell	North Carolina	159,437	11,655	6,922.00	16,462.00	4,706,166.63	2,477,678.18	7,585,294.15	8,691,286.49	3,003,377.82	19,680,781.43	13,397,453.12	5,772,890.25	26,057,865.70
37099	Jackson	North Carolina	40,271	2,893	1,666.00	3,978.00	1,303,156.14	557,628.17	2,534,029.59	2,240,443.24	459,953.17	7,327,331.81	3,543,599.38	1,181,111.49	9,542,872.06
37101	Johnston	North Carolina	168,878	14,872	9,809.00	19,981.50	5,459,832.66	3,169,423.72	8,503,545.67	9,092,033.30	3,562,027.84	22,324,389.15	14,551,865.97	7,336,112.71	28,103,784.57
37103	Jones	North Carolina	10,153	925	589.50	1,300.50	445,088.28	154,306.71	1,112,489.23	783,583.02	128,774.52	4,144,858.69	1,228,671.30	312,839.91	4,720,479.09
37105	Lee	North Carolina	57,866	4,679	3,056.00	6,460.50	1,919,056.22	901,580.64	3,428,705.24	3,510,881.98	998,379.17	11,895,155.72	5,429,938.20	2,169,378.81	13,899,516.49
37107	Lenoir	North Carolina	59,495	5,362	3,585.50	7,284.00	2,414,749.30	1,286,274.56	4,165,829.52	4,049,367.12	1,249,651.33	12,513,495.90	6,464,116.42	2,767,961.42	15,479,095.69
37109	Lincoln	North Carolina	78,265	5,604	3,313.50	7,924.50	2,339,781.17	1,163,350.10	4,135,032.63	4,014,623.24	965,536.86	12,164,743.87	6,354,404.41	2,325,712.09	14,817,780.29
37111	McDowell	North Carolina	44,996	3,283	1,884.50	4,694.00	1,521,143.01	669,116.36	2,954,198.62	2,667,047.08	581,385.36	8,746,304.89	4,188,190.10	1,428,799.61	10,750,582.20
37113	Macon	North Carolina	33,922	2,497	1,526.50	3,437.00	1,354,355.79	569,521.54	2,750,003.35	2,524,758.41	529,046.16	8,721,136.36	3,879,114.20	1,229,695.44	10,620,159.14
37115	Madison	North Carolina	20,764	1,505	905.50	2,150.00	740,037.77	281,831.67	1,692,800.14	1,249,835.70	200,594.49	5,221,139.00	1,989,873.47	545,501.11	6,135,536.02
37117	Martin	North Carolina	24,505	2,367	1,573.50	3,150.50	1,144,313.79	510,877.95	2,325,932.55	1,954,281.31	434,578.96	6,848,159.56	3,098,595.11	1,119,635.40	8,075,972.12
37119	Mecklenburg	North Carolina	919,628	66,102	39,503.50	92,868.00	23,334,921.69	13,055,018.98	33,697,258.50	43,403,822.31	22,598,614.43	72,961,060.24	66,738,744.00	36,385,599.91	102,755,126.10
37121	Mitchell	North Carolina	15,579	1,120	655.00	1,578.50	562,147.39	194,441.49	1,288,024.39	1,119,613.79	161,461.23	4,780,385.51	1,681,761.18	413,526.46	5,482,470.45
37123	Montgomery	North Carolina	27,798	2,079	1,223.50	2,933.00	926,028.93	361,665.66	2,095,779.74	1,513,555.56	324,036.97	5,374,166.46	2,439,584.49	795,831.97	6,655,983.45
37125	Moore	North Carolina	88,247	6,862	4,431.00	9,496.00	3,508,715.99	1,820,882.82	5,800,239.40	6,912,973.86	2,368,961.47	17,927,885.54	10,421,689.84	4,656,664.38	22,246,400.70
37127	Nash	North Carolina	95,840	8,942	5,821.00	11,681.50	3,852,048.16	2,156,170.56	6,329,728.55	6,719,614.47	2,290,796.54	17,974,589.02	10,571,662.63	4,936,195.26	22,558,822.75
37129	New Hanover	North Carolina	202,667	16,383	9,738.50	23,236.00	6,951,794.55	3,723,757.57	10,822,343.78	12,626,178.57	5,063,651.61	27,725,256.28	19,577,973.11	9,646,619.41	35,820,360.10
37131	Northampton	North Carolina	22,099	2,162	1,486.00	2,833.50	1,077,331.59	509,960.54	2,265,602.21	2,073,558.68	425,941.20	7,103,374.41	3,150,890.27	1,018,492.88	8,681,640.69
37133	Onslow	North Carolina	177,772	16,523	9,933.00	22,064.50	5,346,536.28	2,950,535.06	8,284,776.85	8,839,893.41	3,350,946.30	21,608,146.14	14,186,429.69	6,716,074.47	28,196,836.23
37135	Orange	North Carolina	133,801	10,542	7,095.00	13,883.50	4,023,860.25	2,307,741.69	6,480,081.69	7,081,182.60	2,601,284.14	16,866,919.39	11,105,042.86	5,290,407.71	21,871,134.80
37137	Pamlico	North Carolina	13,144	1,218	761.00	1,653.50	657,039.69	238,687.84	1,513,321.28	1,182,955.69	182,459.40	4,954,514.99	1,839,995.39	479,024.72	5,817,949.13
37139	Pasquotank	North Carolina	40,661	4,031	2,753.50	5,290.00	1,657,777.83	874,368.69	2,928,424.27	2,885,760.20	791,841.15	10,602,672.48	4,543,538.03	1,796,657.50	12,558,382.40
37141	Pender	North Carolina	52,217	4,514	2,813.00	6,195.50	1,999,463.54	970,106.75	3,806,351.99	3,532,184.70	938,726.43	10,012,403.77	5,531,648.24	2,192,485.64	12,591,834.10
37143	Perquimans	North Carolina	13,453	1,339	919.50	1,748.50	715,040.37	270,935.03	1,674,772.93	1,201,356.79	222,400.09	5,172,949.60	1,916,397.16	553,653.08	6,047,272.00
37145	Person	North Carolina	39,464	3,293	2,230.50	4,423.50	1,471,467.59	730,284.22	2,865,321.04	2,533,949.79	656,899.40	8,183,234.60	4,005,417.37	1,546,569.42	10,015,078.59
37147	Pitt	North Carolina	168,148	15,553	10,501.00	20,872.00	5,699,299.27	3,487,717.22	8,531,714.02	9,424,117.82	3,757,584.70	21,336,272.19	15,123,417.09	7,845,168.65	28,648,948.90
37149	Polk	North Carolina	20,510	1,503	912.00	2,093.50	817,659.11	321,998.75	1,783,233.38	1,533,754.94	285,680.24	6,566,616.37	2,351,414.05	744,299.18	7,827,195.22
37151	Randolph	North Carolina	141,752	10,724	6,772.00	14,692.50	4,531,237.96	2,414,187.97	7,490,343.00	7,721,297.49	2,511,379.01	19,113,881.54	12,252,535.45	5,435,125.67	24,052,672.05
37153	Richmond	North Carolina	46,639	3,582	1,987.00	5,146.00	1,529,249.65	636,324.36	2,822,721.25	2,606,353.04	555,723.10	8,305,599.46	4,135,602.69	1,295,776.00	10,518,483.93
37155	Robeson	North Carolina	134,168	10,771	6,460.50	15,092.50	4,132,511.54	2,150,063.90	6,496,229.97	6,612,915.69	1,947,851.48	17,488,053.40	10,745,427.23	4,314,072.04	22,601,400.83
37157	Rockingham	North Carolina	93,643	7,354	4,647.50	10,076.00	3,336,514.26	1,799,325.51	5,420,385.62	5,705,807.76	1,880,320.62	13,633,274.11	9,042,322.03	4,116,714.62	17,557,518.81
37159	Rowan	North Carolina	138,428	10,134	6,077.50	13,977.50	4,321,232.78	2,379,886.65	7,070,465.74	7,755,130.37	2,682,360.75	17,838,219.02	12,076,363.16	5,358,609.52	23,132,597.22
37161	Rutherford	North Carolina	67,810	4,977	3,007.00	6,947.00	2,280,535.97	1,149,231.24	3,881,542.10	4,102,355.67	1,031,180.00	11,828,924.48	6,382,891.64	2,475,639.50	14,445,705.01
37163	Sampson	North Carolina	63,431	5,499	3,730.50	7,503.50	2,284,115.83	1,166,754.15	3,823,453.36	3,917,125.60	1,053,779.00	12,049,782.92	6,201,241.43	2,506,796.50	14,897,426.92
37165	Scotland	North Carolina	36,157	2,809	1,752.50	3,871.50	1,182,770.74	517,627.06	2,317,690.26	1,924,535.65	440,847.16	7,178,999.10	3,107,306.38	1,057,330.43	8,752,968.68
37167	Stanly	North Carolina	60,585	4,465	2,597.50	6,283.50	1,986,706.22	910,167.81	3,724,347.42	3,446,358.94	834,163.73	10,781,479.16	5,433,065.16	1,996,942.95	13,266,489.97
37169	Stokes	North Carolina	47,401	3,565	2,201.00	4,872.50	1,622,224.61	773,727.15	3,061,001.43	2,879,660.41	647,323.28	9,923,299.72	4,501,885.02	1,594,712.82	12,067,589.33
37171	Surry	North Carolina	73,673	5,481	3,289.00	7,684.00	2,481,414.27	1,149,260.78	4,260,668.63	4,355,409.19	1,189,357.57	13,074,697.27	6,836,823.46	2,569,640.37	15,415,281.91
37173	Swain	North Carolina	13,981	1,055	664.00	1,488.50	475,963.45	164,174.28	1,171,886.60	783,331.28	138,855.70	3,306,845.39	1,259,294.73	348,355.16	3,972,513.84
37175	Transylvania	North Carolina	33,090	2,465	1,548.50	3,433.00	1,384,067.55	598,611.62	2,706,191.93	2,673,022.07	577,903.20	9,748,959.34	4,057,089.62	1,301,582.61	11,718,027.89
37177	Tyrrell	North Carolina	4,407	415	270.00	561.00	186,134.63	48,799.74	607,065.01	353,395.72	48,672.07	2,295,614.86	539,530.34	110,249.80	2,802,786.72
37179	Union	North Carolina	201,292	14,994	8,925.00	21,499.00	5,266,695.95	2,831,912.04	8,402,811.84	8,914,049.58	3,243,881.03	22,148,010.61	14,180,745.53	6,396,954.36	28,341,778.81
37181	Vance	North Carolina	45,422	4,197	2,890.50	5,580.50	1,765,540.31	921,724.38	3,169,109.95	2,781,284.64	820,754.33	7,978,265.99	4,546,824.95	1,938,542.57	9,898,056.11
37183	Wake	North Carolina	900,993	76,746	51,242.50	102,192.00	26,793,687.10	17,527,168.27	36,191,449.05	46,282,395.10	26,042,603.90	75,856,790.13	73,076,082.20	44,725,079.17	107,367,929.60
37185	Warren	North Carolina	20,972	1,955	1,324.50	2,606.50	954,710.02	433,250.45	1,872,076.47	1,718,981.25	360,818.24	5,804,146.57	2,673,691.26	886,768.82	7,021,358.21
37187	Washington	North Carolina	13,228	1,325	886.50	1,766.00	633,597.40	259,173.83	1,458,242.85	1,086,109.87	184,472.72	4,815,672.20	1,719,707.27	513,413.48	5,532,547.91

37189	Watauga	North Carolina	51,079	3,470	1,931.00	4,791.50	1,422,299.06	582,868.00	2,590,519.22	2,366,659.42	522,900.43	8,145,080.80	3,788,958.48	1,281,060.68	9,743,979.90
37191	Wayne	North Carolina	122,623	11,083	7,205.50	15,151.50	4,506,606.41	2,463,957.10	7,158,809.64	7,677,681.13	2,697,858.13	19,311,620.32	12,184,287.54	5,863,741.70	24,410,803.78
37193	Wilkes	North Carolina	69,340	5,053	2,983.00	7,180.50	2,310,484.52	1,121,639.15	4,226,719.15	3,970,495.15	1,004,949.19	11,737,897.24	6,280,979.67	2,287,314.03	14,014,685.85
37195	Wilson	North Carolina	81,234	7,540	5,034.00	10,028.00	3,168,978.03	1,781,483.99	5,201,679.37	5,543,773.36	1,973,306.93	13,655,792.42	8,712,751.38	4,107,381.27	17,340,612.16
37197	Yadkin	North Carolina	38,406	2,812	1,662.00	4,101.00	1,263,835.92	524,637.16	2,620,414.62	2,247,278.15	441,452.02	8,462,222.05	3,511,114.07	1,116,284.11	10,424,136.50
37199	Yancey	North Carolina	17,818	1,301	759.00	1,820.50	644,157.48	219,444.67	1,358,354.93	1,195,533.10	178,314.06	4,914,895.07	1,839,690.58	488,113.39	5,691,305.51
38001	Adams	North Dakota	2,343	110	11.50	221.50	60,307.29	1,091.01	271,110.91	95,041.75	1,716.43	736,278.28	155,349.04	3,183.69	892,755.06
38003	Barnes	North Dakota	11,066	625	145.00	1,105.50	326,153.79	31,820.96	910,690.52	631,136.08	31,197.40	3,604,883.85	957,289.87	65,454.11	4,308,537.65
38005	Benson	North Dakota	6,660	396	69.50	739.00	139,518.67	8,910.93	436,808.71	212,744.76	12,193.86	1,422,403.81	352,263.42	20,966.67	1,733,729.76
38007	Billings	North Dakota	783	38	3.00	79.00	23,621.51	97.11	125,073.94	36,166.79	412.58	168,524.25	59,788.30	627.53	371,780.32
38009	Bottineau	North Dakota	6,429	369	54.50	733.00	196,356.30	9,496.25	694,289.34	357,467.28	10,137.65	2,216,361.84	553,823.58	22,348.88	2,439,039.75
38011	Bowman	North Dakota	3,151	150	13.00	310.00	88,481.12	633.65	356,532.10	130,096.64	2,334.82	918,194.05	218,577.76	3,197.11	1,172,021.33
38013	Burke	North Dakota	1,968	112	11.50	222.00	64,965.64	798.94	291,347.97	119,973.20	2,138.55	880,571.11	184,938.84	3,612.46	1,026,869.78
38015	Burleigh	North Dakota	81,308	4,223	499.50	8,211.50	1,759,400.13	161,008.34	3,976,730.75	3,258,630.97	163,581.94	11,420,051.42	5,018,031.10	364,037.10	14,373,314.11
38017	Cass	North Dakota	149,778	8,371	1,887.00	14,813.00	3,079,036.96	613,651.53	6,087,086.62	5,385,162.93	617,519.48	16,000,965.07	8,464,199.89	1,350,335.70	19,666,715.58
38019	Cavalier	North Dakota	3,993	228	39.50	429.00	124,897.09	5,613.76	435,071.75	212,020.11	9,224.65	1,522,710.22	336,917.20	15,100.16	1,733,805.99
38021	Dickey	North Dakota	5,289	278	42.50	521.50	139,327.73	8,735.52	514,850.30	242,254.18	9,519.69	1,592,858.13	381,581.91	19,176.70	1,936,002.19
38023	Divide	North Dakota	2,071	124	20.00	236.00	74,807.02	1,705.19	292,217.30	127,021.31	3,341.54	943,772.88	201,828.33	5,947.70	1,111,524.18
38025	Dunn	North Dakota	3,536	186	24.00	379.00	90,318.36	2,168.52	330,064.43	135,331.58	4,582.29	994,772.56	225,649.94	8,081.65	1,202,288.15
38027	Eddy	North Dakota	2,385	129	23.50	241.00	71,826.66	2,165.80	309,482.15	161,219.61	4,055.98	1,126,151.05	233,046.27	7,283.14	1,305,785.70
38029	Emmons	North Dakota	3,550	185	34.50	359.00	111,887.98	3,718.05	440,061.14	201,502.31	6,062.46	1,294,772.34	313,390.28	9,872.99	1,517,764.79
38031	Foster	North Dakota	3,343	182	21.50	334.50	87,947.60	3,026.38	351,355.01	199,238.43	5,780.70	1,472,413.53	287,186.03	9,908.59	1,654,129.47
38033	Golden Valley	North Dakota	1,680	82	9.00	170.00	41,229.57	431.88	245,014.77	66,156.56	1,132.08	501,847.89	107,386.13	1,881.32	626,787.70
38035	Grand Forks	North Dakota	66,861	3,705	795.00	6,484.50	1,414,624.31	260,493.96	3,090,162.67	2,294,645.05	224,656.71	8,088,471.63	3,709,269.35	557,480.72	10,295,476.53
38037	Grant	North Dakota	2,394	117	13.00	233.00	64,822.65	1,535.79	276,369.26	109,721.65	2,111.47	824,583.50	174,544.30	4,068.54	999,015.09
38039	Griggs	North Dakota	2,420	133	28.00	243.50	79,279.40	3,345.39	339,940.77	141,732.19	5,761.54	1,209,334.18	221,011.60	9,557.97	1,422,352.71
38041	Hettinger	North Dakota	2,477	121	12.00	244.50	62,773.92	1,222.74	262,858.74	115,366.84	2,297.94	766,873.62	178,140.75	3,690.87	855,108.20
38043	Kidder	North Dakota	2,435	132	22.00	241.50	64,366.74	2,270.93	286,272.89	116,753.14	2,981.27	1,061,637.03	181,119.88	5,998.34	1,159,865.58
38045	La Moure	North Dakota	4,139	219	38.50	410.50	121,729.05	6,415.36	479,787.47	225,246.56	9,077.24	1,360,984.86	346,975.61	17,116.07	1,533,865.54
38047	Logan	North Dakota	1,990	104	13.00	198.50	68,543.21	956.84	332,895.90	135,310.20	1,920.41	962,577.13	203,853.42	3,108.19	1,193,158.12
38049	McHenry	North Dakota	5,395	303	46.50	557.00	155,097.70	7,888.46	631,064.48	262,633.24	10,406.94	1,660,846.25	417,730.94	21,520.29	1,895,067.41
38051	McIntosh	North Dakota	2,809	150	21.50	282.50	99,622.01	2,758.63	399,639.83	242,633.14	4,005.82	1,647,996.37	342,255.15	9,514.96	1,961,335.49
38053	McKenzie	North Dakota	6,360	338	40.50	674.00	138,987.56	3,890.53	500,392.11	258,598.06	8,072.26	1,635,018.07	397,585.61	13,639.34	1,975,925.87
38055	McLean	North Dakota	8,962	486	87.50	904.50	249,425.14	20,577.47	780,415.41	512,062.78	29,099.69	2,960,336.42	761,487.92	52,070.43	3,339,365.05
38057	Mercer	North Dakota	8,424	451	65.50	853.00	211,408.44	10,551.30	701,555.54	403,975.62	22,343.13	1,871,252.87	615,384.07	35,539.74	2,184,948.35
38059	Morton	North Dakota	27,471	1,412	112.00	2,771.50	632,096.06	22,976.70	1,658,077.04	1,015,184.56	23,262.73	4,486,895.08	1,647,280.61	44,642.68	5,619,354.65
38061	Mountrial	North Dakota	7,673	416	65.00	805.50	184,382.66	10,472.42	683,144.87	272,227.46	14,146.63	1,518,045.89	456,610.12	28,004.20	1,834,027.00
38063	Nelson	North Dakota	3,126	176	37.00	314.00	101,019.42	4,329.77	408,710.48	179,531.43	7,004.64	1,329,976.84	280,550.85	12,996.49	1,565,251.40
38065	Oliver	North Dakota	1,846	96	11.50	186.00	45,945.03	823.18	218,955.79	93,099.11	3,682.40	616,594.87	139,044.15	4,810.49	745,415.52
38067	Pembina	North Dakota	7,413	425	85.50	756.50	207,783.45	18,506.04	717,519.18	474,518.59	21,841.68	2,641,445.21	682,302.04	46,465.02	2,962,124.86
38069	Pierce	North Dakota	4,357	244	32.00	462.00	130,219.21	5,080.74	496,229.80	186,967.30	5,811.42	1,388,095.13	317,186.51	11,057.95	1,675,989.54
38071	Ramsey	North Dakota	11,451	635	93.00	1,207.00	316,055.12	18,183.87	1,069,122.52	518,410.70	17,416.91	2,537,246.39	834,465.82	39,332.88	3,456,630.48
38073	Ransom	North Dakota	5,457	311	86.50	541.50	149,456.36	13,833.15	485,619.37	302,452.02	18,760.99	1,750,278.39	451,908.39	35,092.42	2,034,342.59
38075	Renville	North Dakota	2,470	144	24.50	269.50	65,684.90	1,729.51	257,218.63	100,292.24	4,944.19	685,912.04	165,977.14	6,920.65	826,801.95
38077	Richland	North Dakota	16,321	937	278.00	1,587.50	424,950.70	55,205.84	1,014,500.25	711,528.08	60,007.78	3,370,197.54	1,136,478.79	129,226.20	3,859,855.19
38079	Rolette	North Dakota	13,937	874	114.50	1,596.50	299,104.63	18,792.04	830,108.79	534,235.22	15,456.60	2,507,244.90	833,339.86	34,130.44	2,882,857.01
38081	Sargent	North Dakota	3,829	208	55.00	369.00	104,733.34	7,296.05	401,519.61	189,880.18	14,933.42	1,383,269.86	294,613.51	24,359.25	1,568,842.76
38083	Sheridan	North Dakota	1,321	71	13.50	135.50	44,394.97	911.03	227,327.07	70,509.39	2,126.08	539,144.61	114,904.36	3,183.73	767,163.25
38085	Sioux	North Dakota	4,153	227	27.00	446.50	68,038.60	2,514.00	295,949.46	86,192.66	4,015.36	671,403.46	154,231.26	6,969.46	836,054.67
38087	Slope	North Dakota	727	34	2.50	70.00	16,265.78	37.56	101,707.47	24,384.83	267.72	163,314.82	40,650.60	330.59	240,763.95
38089	Stark	North Dakota	24,199	1,208	140.00	2,358.00	528,841.88	26,928.31	1,419,052.02	987,280.80	29,439.72	4,437,931.52	1,516,122.67	61,445.37	5,412,367.57
38091	Steele	North Dakota	1,975	111	24.00	205.50	62,676.90	2,271.57	297,150.02	123,302.07	5,482.79	843,841.30	185,978.97	8,448.60	959,740.61
38093	Stutsman	North Dakota	21,100	1,134	203.50	2,091.50	546,457.08	56,981.38	1,431,128.68	978,570.81	43,816.73	5,139,524.51	1,525,027.88	119,701.07	5,883,917.48
38095	Towner	North Dakota	2,246	124	14.50	232.50	69,980.31	1,069.01	318,918.94	110,296.17	2,801.84	898,756.08	180,276.49	4,254.22	1,072,122.28
38097	Traill	North Dakota	8,121	470	123.50	823.50	224,976.25	20,222.07	735,996.62	374,167.88	26,865.68	2,359,949.84	599,144.13	49,240.67	2,789,114.04
38099	Walsh	North Dakota	11,119	634	106.50	1,170.00	314,219.06	22,189.80	956,315.29	622,594.57	26,600.50	3,654,273.96	936,813.63	61,733.19	4,302,534.08
38101	Ward	North Dakota	61,675	3,543	439.50	6,627.00	1,429,781.11	141,449.93	3,349,359.32	2,383,712.40	95,682.11	8,515,481.61	3,813,493.50	260,687.95	10,264,094.10
38103	Wells	North Dakota	4,207	240	47.00	426.50	141,228.47	7,572.00	506,271.45	285,595.37	10,338.63	1,516,718.38	426,823.78	20,100.80	1,851,359.07
38105	Williams	North Dakota	22,398	1,197	149.00	2,341.50	542,286.42	44,068.00	1,563,108.49	968,713.88	49,154.41	4,239,605.51	1,511,000.30	101,981.81	5,134,531.87
39001	Adams	Ohio	28,550	2,096	1,204.50	3,004.00	911,206.17	319,527.19	1,904,712.02	1,661,682.77	246,253.64	7,357,815.48	2,572,888.94	650,582.23	8,547,094.20
39003	Allen	Ohio	106,331	7,660	4,683.00	10,575.50	3,320,427.47	1,677,844.41	5,880,898.16	5,891,406.42	1,803,154.84	16,337,981.08	9,211,833.89	3,898,337.01	19,758,520.02
39005	Ashland	Ohio	53,139	4,020	2,412.50	5,583.50	1,775,022.25	876,623.89	3,157,130.74	3,066,334.10	777,861.95	9,569,052.11	4,841,356.36	1,819,468.18	11,249,114.51
39007	Ashtabula	Ohio	101,497	8,657	5,241.00	11,756.50	3,845,142.27	2,105,870.56	6,033,853.95	6,990,286.42	2,473,169.14	18,726,528.26	10,835,428.69	5,098,347.76	23,444,890.54
39009	Athens	Ohio	64,757	4,447	2,541.50	6,249.50	1,670,804.70	752,121.97	3,094,047.50	2,819,869.78	638,381.81	10,514,408.78	4,490,674.47	1,614,073.88	12,720,054.69
39011	Auglaize	Ohio	45,949	3,341	1,984.50	4,689.00	1,468,590.27	643,613.71	2,792,125.20	2,683,499.85	646,375.67	9,769,838.63	4,152,090.12	1,414,726.67	12,010,765.00
39013	Belmont	Ohio	70,400	5,687	3,505.00	7,882.50	2,721,296.13	1,373,619.95	4,672,975.58	5,080,032.07	1,358,500.76	13,729,439.25	7,801,328.20	3,179,949.42	16,824,848.96

39015	Brown	Ohio	44,846	3,213	1,874.00	4,556.50	1,373,960.94	577,135.85	2,635,092.38	2,449,279.26	525,386.05	9,132,201.49	3,823,240.20	1,346,073.57	10,705,469.93
39017	Butler	Ohio	368,130	26,712	16,117.50	37,260.50	10,407,256.96	5,795,574.01	15,347,033.35	17,924,988.20	8,312,527.83	33,885,353.86	28,332,245.16	15,457,567.79	46,781,111.84
39019	Carroll	Ohio	28,836	2,396	1,490.50	3,230.00	1,091,901.58	501,640.13	2,229,149.67	1,954,318.71	371,180.51	6,806,328.07	3,046,220.29	987,097.55	8,376,844.50
39021	Champaign	Ohio	40,097	2,842	1,665.00	3,967.00	1,190,394.67	515,488.00	2,440,046.87	2,127,237.57	454,864.28	7,794,907.00	3,317,632.24	1,068,371.63	9,350,931.64
39023	Clark	Ohio	138,333	9,765	5,976.00	13,409.50	4,379,810.14	2,265,704.80	7,292,286.73	7,689,197.37	2,787,449.40	19,714,227.92	12,069,007.51	5,571,520.94	24,607,952.66
39025	Clermont	Ohio	197,363	14,413	8,817.50	19,955.00	5,812,162.59	3,144,256.09	9,119,518.60	9,725,891.26	3,669,442.04	21,699,873.48	15,538,053.85	7,374,212.61	28,097,838.33
39027	Clinton	Ohio	42,040	3,024	1,820.00	4,251.50	1,288,233.91	544,581.09	2,661,694.04	2,548,507.23	665,983.27	8,302,445.19	3,836,741.15	1,362,345.84	9,544,015.19
39029	Columbiana	Ohio	107,841	9,356	6,207.00	12,667.00	4,335,434.55	2,442,113.06	7,046,716.93	7,705,598.05	2,681,954.64	18,641,204.39	12,041,032.60	5,588,777.02	24,689,853.66
39031	Coshocton	Ohio	36,901	2,829	1,671.00	3,940.00	1,289,273.15	560,649.84	2,629,469.23	2,364,219.74	490,497.10	8,661,843.90	3,653,492.89	1,210,855.87	10,470,576.77
39033	Crawford	Ohio	43,784	3,209	1,803.00	4,564.50	1,470,717.98	647,918.95	2,658,620.45	2,685,374.46	587,978.67	8,227,638.96	4,156,092.44	1,425,828.60	10,370,585.83
39035	Cuyahoga	Ohio	1,280,122	97,429	61,619.50	134,437.00	42,886,153.93	26,104,659.04	60,626,548.12	84,282,346.07	46,675,289.94	133,248,533.10	127,168,500.00	75,046,576.88	188,976,963.70
39037	Darke	Ohio	52,959	3,829	2,365.00	5,490.50	1,706,361.35	832,782.67	3,071,433.71	3,033,731.20	825,560.80	9,130,317.26	4,740,092.56	1,869,663.31	11,208,065.05
39039	Defiance	Ohio	39,037	2,777	1,626.50	3,950.50	1,200,927.76	503,080.60	2,380,828.43	2,153,673.01	495,360.61	7,314,557.64	3,354,600.78	1,117,003.60	9,154,872.62
39041	Delaware	Ohio	174,214	12,749	7,847.50	17,986.50	4,697,311.16	2,498,660.69	7,858,251.35	8,228,989.59	3,264,837.87	18,251,730.22	12,926,300.75	6,146,138.90	24,696,903.48
39043	Erie	Ohio	77,079	5,744	3,417.00	8,068.50	2,697,993.68	1,324,678.78	4,583,921.82	4,921,532.90	1,394,959.15	14,480,673.56	7,619,526.58	2,998,188.44	17,832,318.52
39045	Fairfield	Ohio	146,156	10,631	6,212.00	14,611.50	4,294,489.42	2,112,682.08	7,315,587.63	7,098,160.53	2,374,791.64	17,629,580.92	11,392,649.95	4,986,207.00	23,688,081.54
39047	Fayette	Ohio	29,030	2,079	1,263.00	2,871.00	895,475.76	371,309.48	1,884,794.31	1,452,584.02	313,106.08	5,562,918.79	2,348,059.79	772,882.53	6,958,528.25
39049	Franklin	Ohio	1,163,414	81,948	46,985.00	113,809.00	30,319,093.37	16,871,568.35	43,387,945.59	54,346,861.20	25,978,565.02	89,695,888.19	84,665,954.57	44,736,617.18	128,885,218.10
39051	Fulton	Ohio	42,698	3,137	1,813.50	4,368.00	1,344,787.41	574,376.21	2,662,211.48	2,291,079.39	520,771.30	8,555,464.62	3,635,866.80	1,233,736.82	10,194,364.46
39053	Gallia	Ohio	30,934	2,252	1,291.50	3,184.50	986,123.17	381,026.36	2,024,670.35	1,876,730.21	385,924.17	7,418,468.57	2,862,853.38	892,092.92	8,760,968.55
39055	Geauga	Ohio	93,389	7,659	4,856.50	10,513.00	3,421,231.06	1,842,682.90	5,786,288.48	6,059,791.43	2,102,898.43	16,296,012.53	9,481,022.50	4,299,212.42	20,681,296.68
39057	Greene	Ohio	161,573	11,296	7,014.50	15,803.50	4,810,615.38	2,580,889.34	7,733,605.21	8,994,295.75	3,362,533.13	20,384,128.29	13,804,911.13	6,353,604.85	26,464,326.92
39059	Guernsey	Ohio	40,087	3,137	1,803.00	4,422.00	1,398,328.71	604,903.30	2,658,048.95	2,376,447.60	564,447.03	8,240,051.02	3,774,776.31	1,387,986.10	10,105,165.85
39061	Hamilton	Ohio	802,374	58,032	35,884.00	79,296.00	24,131,285.76	13,933,581.45	34,764,679.57	46,466,176.42	24,367,768.26	77,229,680.31	70,597,462.18	39,597,474.53	106,758,979.20
39063	Hancock	Ohio	74,782	5,370	2,940.50	7,573.00	2,278,052.36	1,011,585.61	3,887,301.42	4,129,544.37	1,231,545.18	12,479,828.64	6,407,596.72	2,367,926.36	14,969,413.94
39065	Hardin	Ohio	32,058	2,268	1,336.50	3,247.00	937,756.61	378,750.86	1,892,962.42	1,536,851.85	313,222.73	5,184,967.81	2,474,608.46	774,084.61	6,460,017.83
39067	Harrison	Ohio	15,864	1,297	823.00	1,797.00	629,893.03	237,156.10	1,406,529.50	1,166,813.45	200,586.06	4,974,259.41	1,796,706.48	471,014.26	5,854,649.94
39069	Henry	Ohio	28,215	2,053	1,178.00	2,938.50	902,102.91	356,082.53	1,927,998.79	1,533,753.32	329,773.07	5,688,941.94	2,435,856.22	756,833.61	6,971,148.32
39071	Highland	Ohio	43,589	3,178	1,890.00	4,501.00	1,362,506.52	589,819.81	2,758,573.23	2,405,088.59	537,522.62	7,876,236.20	3,767,595.11	1,322,739.20	9,244,001.77
39073	Hocking	Ohio	29,380	2,109	1,208.50	2,960.00	924,258.52	364,762.03	1,980,399.74	1,605,628.49	291,705.48	6,726,186.62	2,529,887.01	724,995.14	7,857,809.32
39075	Holmes	Ohio	42,366	3,486	2,138.50	4,834.00	1,264,824.87	542,821.52	2,401,452.37	1,965,385.90	463,904.89	6,948,873.12	3,230,210.77	1,101,141.12	8,439,942.99
39077	Huron	Ohio	59,626	4,538	2,637.00	6,507.50	1,871,872.22	842,551.56	3,547,891.50	3,315,722.81	825,617.60	10,177,195.61	5,187,595.03	1,931,125.89	12,589,036.95
39079	Jackson	Ohio	33,225	2,406	1,332.00	3,449.50	1,030,427.60	390,535.08	2,113,909.91	1,873,738.33	339,807.58	8,185,700.14	2,904,165.93	852,992.07	9,513,732.41
39081	Jefferson	Ohio	69,709	5,914	3,851.50	8,077.50	2,872,815.28	1,544,779.84	4,769,242.36	5,378,338.52	1,790,999.04	14,640,027.68	8,251,153.79	3,752,516.69	17,976,955.82
39083	Knox	Ohio	60,921	4,530	2,698.00	6,387.50	1,953,379.33	966,776.64	3,476,678.40	3,404,787.98	941,676.17	10,470,582.17	5,358,167.31	2,016,882.81	12,777,615.90
39085	Lake	Ohio	230,041	18,616	11,221.00	26,093.00	8,513,318.39	4,798,787.59	12,977,834.55	15,672,844.54	6,209,347.38	33,271,621.80	24,186,162.94	11,698,352.93	44,982,556.18
39087	Lawrence	Ohio	62,450	4,576	2,690.50	6,460.00	2,014,300.69	961,213.20	3,773,770.06	3,479,438.18	801,841.96	11,021,334.23	5,493,738.86	1,996,884.83	13,735,557.09
39089	Licking	Ohio	166,492	12,145	7,259.50	17,205.50	5,057,062.05	2,568,248.07	7,952,287.98	8,683,177.52	3,282,057.75	20,830,410.89	13,740,239.57	6,485,094.98	26,425,336.01
39091	Logan	Ohio	45,858	3,283	1,928.00	4,666.50	1,414,451.93	620,090.49	2,687,675.36	2,519,081.70	681,805.48	8,387,872.66	3,933,533.63	1,414,522.78	9,994,499.86
39093	Lorain	Ohio	301,356	22,981	13,896.00	32,032.50	9,850,242.58	5,493,356.93	15,416,712.98	17,860,722.75	7,860,912.52	37,693,489.58	27,710,965.33	14,036,961.05	50,482,867.36
39095	Lucas	Ohio	441,815	32,685	19,525.50	46,493.00	13,524,898.03	7,575,892.66	20,135,982.20	24,173,620.33	10,780,177.95	46,051,597.10	37,698,518.35	20,249,501.54	62,896,444.88
39097	Madison	Ohio	43,435	3,025	1,817.50	4,214.50	1,248,180.53	515,371.58	2,511,305.15	2,103,489.66	480,135.79	7,194,639.70	3,351,670.19	1,074,019.92	8,782,647.64
39099	Mahoning	Ohio	238,823	20,204	13,341.00	27,059.00	9,642,276.70	5,788,172.97	14,272,928.86	17,180,811.20	8,080,356.66	33,250,967.11	26,823,087.90	14,937,895.80	45,765,641.06
39101	Marion	Ohio	66,501	4,720	2,903.00	6,486.00	2,038,010.88	987,430.99	3,740,609.29	3,467,358.51	930,853.49	10,389,242.65	5,505,369.39	2,174,080.00	12,858,498.81
39103	Medina	Ohio	172,332	13,298	8,161.50	18,364.50	5,514,210.83	3,054,485.05	8,763,472.66	9,817,654.31	3,487,610.06	22,705,080.56	15,331,865.14	7,185,647.10	28,810,721.20
39105	Meigs	Ohio	23,770	1,731	994.50	2,459.50	774,009.66	284,353.73	1,667,644.19	1,282,808.39	221,834.77	4,685,692.25	2,056,818.05	539,782.54	5,573,991.35
39107	Mercer	Ohio	40,814	2,979	1,838.50	4,149.00	1,314,870.55	575,884.17	2,578,155.62	2,335,555.21	510,696.25	7,816,132.33	3,650,425.76	1,223,531.42	9,569,762.27
39109	Miami	Ohio	102,506	7,225	4,503.00	10,101.00	3,106,862.48	1,620,973.52	5,020,412.00	5,513,715.53	1,698,365.92	14,239,884.75	8,620,578.01	3,585,473.25	17,610,614.99
39111	Monroe	Ohio	14,642	1,166	704.00	1,660.50	574,482.77	196,705.11	1,379,777.29	1,141,309.95	185,723.35	4,805,044.25	1,715,792.71	431,385.59	5,721,012.13
39113	Montgomery	Ohio	535,153	37,638	23,394.00	52,956.00	16,481,118.19	9,319,682.83	24,502,599.02	30,607,756.14	15,343,934.65	56,725,966.42	47,088,874.33	26,177,459.48	78,214,993.01
39115	Morgan	Ohio	15,054	1,154	687.00	1,627.00	534,266.53	184,318.61	1,367,036.59	910,562.48	149,367.07	3,965,758.88	1,444,829.02	377,365.61	4,667,778.90
39117	Morrow	Ohio	34,827	2,566	1,597.00	3,596.50	1,080,634.16	451,279.26	2,294,828.69	1,783,381.53	355,927.56	6,525,601.90	2,864,015.69	954,264.68	8,129,570.96
39119	Muskingum	Ohio	86,074	6,458	3,633.00	9,192.00	2,827,334.91	1,390,396.67	5,023,091.67	5,098,456.64	1,427,415.71	14,695,764.64	7,925,791.55	3,224,497.31	17,921,355.86
39121	Noble	Ohio	14,645	1,134	665.50	1,608.00	596,336.58	218,715.11	1,373,865.36	1,120,915.54	162,854.11	4,283,465.20	1,717,252.12	430,596.02	5,563,919.14
39123	Ottawa	Ohio	41,428	3,027	1,817.50	4,279.50	1,529,941.14	687,103.99	3,068,040.46	2,982,489.86	669,850.50	12,191,510.62	4,512,431.00	1,498,808.46	13,659,916.33
39125	Paulding	Ohio	19,614	1,398	843.00	1,956.00	595,047.59	230,271.39	1,290,233.84	934,053.75	196,191.59	3,632,909.30	1,529,101.33	483,946.67	4,520,428.23
39127	Perry	Ohio	36,058	2,673	1,551.50	3,841.50	1,098,525.37	444,939.86	2,303,749.97	1,873,578.08	389,035.66	6,812,379.74	2,972,103.46	904,551.94	8,058,487.18
39129	Pickaway	Ohio	55,698	3,919	2,329.50	5,536.00	1,586,084.00	739,141.27	3,016,153.70	2,801,425.37	698,242.74	9,699,055.53	4,387,509.37	1,614,636.56	11,634,927.24
39131	Pike	Ohio	28,709	2,086	1,249.00	3,021.00	905,509.61	335,107.63	2,042,175.44	1,844,505.69	386,091.50	7,434,563.37	2,750,015.30	803,028.32	8,416,871.50
39133	Portage	Ohio	161,419	12,788	7,989.50	17,428.00	5,366,964.61	3,018,251.95	8,444,996.76	9,464,951.54	3,520,163.19	22,093,898.26	14,831,916.16	7,002,290.68	29,123,136.88
39135	Preble	Ohio	42,270	3,045	1,866.00	4,256.00	1,348,630.54	620,807.97	2,625,837.03	2,431,780.53	532,011.16	8,013,187.79	3,780,411.07	1,283,207.37	9,649,332.87
39137	Putnam	Ohio	34,499	2,559	1,475.50	3,669.00	1,067,334.00	426,317.30	2,121,873.26	1,813,337.23	390,896.02	6,967,642.89	2,880,671.23	940,467.21	8,194,268.01
39139	Richland	Ohio	124,475	9,214	5,322.00	12,867.00	4,183,534.31	2,101,781.05	6,703,155.43	7,378,017.64	2,624,183.89	18,964,468.96	11,561,551.95	5,233,428.10	23,909,005.15
39141	Ross	Ohio	78,064	5,527	3,253.00	7,846.00	2,374,861.41	1,179,836.91	4,318,233.23	3,965,339.24	1,229,651.74	10,544,096.45	6,340,200.65	2,621,207.27	13,618,835.38
39143	Sandusky	Ohio	60,944	4,482	2,682.50	6,306.50	1,950,437.56	915,770.74	3,513,414.41	3,400,731.04	871,723.45	10,639,876.26	5,351,168.60	2,002,932.22	12,847,407.59
39145	Scioto	Ohio	79,499	5,731	3,326.50	7,908.00	2,515,581.05	1,250,476.91	4,348,893.20	4,409,248.92	1,211,135.06	12,702,660.86	6,924,829.97	2,650,972.69	15,684,549.18

39147	Seneca	Ohio	56,745	4,191	2,508.00	6,001.00	1,819,858.07	863,459.41	3,303,040.83	2,982,573.13	804,645.88	8,740,396.94	4,802,431.20	1,766,106.07	11,166,349.73
39149	Shelby	Ohio	49,423	3,564	2,126.50	4,983.00	1,428,641.44	616,668.88	2,739,860.46	2,601,472.75	662,439.76	9,177,595.55	4,030,114.19	1,386,753.77	10,796,289.84
39151	Stark	Ohio	375,586	30,090	18,673.50	41,494.00	13,619,668.83	7,990,616.77	20,264,811.80	24,496,414.10	11,162,427.92	46,349,921.52	38,116,082.93	20,689,509.41	61,899,344.06
39153	Summit	Ohio	541,781	41,971	27,084.50	58,117.00	18,393,440.40	11,327,404.79	26,971,644.40	34,065,488.75	17,685,420.04	63,560,498.69	52,458,929.14	29,829,047.58	84,687,439.64
39155	Trumbull	Ohio	210,312	17,935	11,680.00	23,930.00	8,457,065.85	4,855,143.68	12,948,009.83	15,423,851.37	6,483,475.69	31,400,404.52	23,880,917.22	12,276,029.93	42,436,460.91
39157	Tuscarawas	Ohio	92,582	7,318	4,490.50	9,949.00	3,277,036.02	1,673,994.81	5,606,545.08	5,722,540.74	1,861,662.38	15,282,031.56	8,999,576.75	3,851,415.07	19,080,901.30
39159	Union	Ohio	52,300	3,736	2,092.00	5,268.00	1,349,843.34	542,402.23	2,534,519.95	2,599,795.33	740,430.07	8,041,636.43	3,949,638.67	1,458,827.62	9,431,796.99
39161	Van Wert	Ohio	28,744	2,061	1,296.50	2,844.00	927,132.36	401,597.58	1,843,082.94	1,557,541.85	303,531.56	5,817,011.79	2,484,674.21	805,534.81	6,901,879.20
39163	Vinton	Ohio	13,435	973	594.00	1,375.00	403,676.82	119,399.49	1,004,594.54	696,132.03	118,822.47	3,365,057.39	1,099,808.84	267,919.87	3,891,786.81
39165	Warren	Ohio	212,693	15,446	9,547.50	21,900.00	5,823,925.66	3,178,740.85	9,248,234.98	10,120,794.08	3,972,001.09	22,014,536.69	15,944,719.74	7,750,293.28	29,355,673.39
39167	Washington	Ohio	61,778	4,691	2,580.00	6,644.00	2,227,017.43	1,023,561.51	3,983,284.76	4,195,894.19	1,077,769.45	12,224,627.94	6,422,911.62	2,270,858.61	15,142,804.18
39169	Wayne	Ohio	114,520	8,849	5,144.50	12,480.00	3,734,777.13	1,809,249.90	6,434,714.29	6,366,266.51	2,189,818.87	15,018,210.68	10,101,043.64	4,325,290.43	19,485,970.52
39171	Williams	Ohio	37,642	2,708	1,543.00	3,826.00	1,215,411.08	464,030.69	2,517,984.85	2,060,382.42	431,723.75	7,298,868.09	3,275,793.50	1,034,794.37	9,156,632.18
39173	Wood	Ohio	125,488	8,960	5,220.50	12,662.50	3,643,467.45	1,819,522.33	6,190,080.78	6,764,724.15	2,111,398.18	17,833,743.53	10,408,191.61	4,330,152.02	22,586,954.47
39175	Wyandot	Ohio	22,615	1,645	1,004.00	2,304.00	746,918.36	282,802.06	1,751,725.60	1,367,464.82	263,429.74	6,342,716.16	2,114,383.19	606,706.68	7,251,537.00
40001	Adair	Oklahoma	22,683	2,567	1,856.50	3,327.00	993,892.89	493,970.19	1,881,594.93	1,649,756.24	342,202.06	6,589,609.58	2,643,649.13	920,471.50	7,666,217.69
40003	Alfalfa	Oklahoma	5,642	558	376.50	743.50	290,990.84	88,434.39	771,609.40	447,189.39	74,838.75	2,269,959.14	738,180.24	180,164.40	2,900,325.52
40005	Atoka	Oklahoma	14,182	1,899	1,441.00	2,385.50	823,598.89	387,380.58	1,768,073.42	1,440,866.59	305,245.89	6,079,075.67	2,264,465.48	770,314.81	7,189,155.59
40007	Beaver	Oklahoma	5,636	550	344.50	765.50	243,734.83	65,414.19	680,780.05	376,689.67	61,842.55	1,826,675.24	620,424.50	142,294.71	2,234,868.36
40009	Beckham	Oklahoma	22,119	2,538	1,792.00	3,307.00	1,002,502.84	466,849.83	2,011,968.83	1,628,190.16	477,434.34	6,059,037.98	2,630,693.00	1,010,496.97	6,741,384.81
40011	Blaine	Oklahoma	11,943	1,366	1,004.00	1,728.50	577,619.23	239,189.42	1,420,778.87	996,994.43	192,111.96	4,728,456.19	1,574,613.66	478,947.97	5,490,575.78
40013	Bryan	Oklahoma	42,416	5,767	4,407.50	7,173.50	2,474,856.65	1,431,034.89	4,013,491.92	4,292,093.20	1,406,873.15	12,198,197.65	6,766,949.85	3,272,956.65	15,110,432.89
40015	Caddo	Oklahoma	29,600	3,620	2,722.50	4,586.50	1,493,510.84	814,463.26	2,660,700.07	2,492,308.78	730,411.68	7,566,608.26	3,985,819.62	1,787,416.19	9,530,185.96
40017	Canadian	Oklahoma	115,541	14,168	10,435.50	17,713.00	5,419,616.24	3,546,521.97	8,298,127.96	8,870,611.42	3,791,866.24	21,770,559.36	14,290,227.65	7,724,742.91	27,692,739.02
40019	Carter	Oklahoma	47,557	6,411	5,000.00	7,819.00	2,722,127.14	1,656,352.50	4,427,294.66	4,894,853.10	1,731,661.32	14,685,930.92	7,616,980.25	3,758,049.34	17,581,932.95
40021	Cherokee	Oklahoma	46,987	5,392	3,953.50	6,774.00	2,206,692.14	1,263,113.68	3,719,249.01	3,734,495.39	1,090,262.71	11,170,550.04	5,941,187.53	2,572,801.78	13,906,783.89
40023	Choctaw	Oklahoma	15,205	2,110	1,564.00	2,599.50	987,474.13	485,793.79	1,854,471.09	1,619,544.59	367,444.42	5,391,963.15	2,607,018.72	939,979.59	6,532,363.43
40025	Cimarron	Oklahoma	2,475	229	133.50	324.50	111,829.14	17,589.99	483,517.68	190,324.01	23,731.53	1,052,502.24	302,153.15	42,291.76	1,333,522.88
40027	Cleveland	Oklahoma	255,755	32,076	24,414.50	39,561.50	12,072,656.44	8,440,724.21	16,103,716.67	19,893,641.95	9,759,585.38	38,621,317.12	31,966,298.38	19,748,175.00	52,310,183.52
40029	Coal	Oklahoma	5,925	793	610.00	1,003.00	358,948.71	126,956.62	940,683.33	573,068.11	104,677.17	3,048,720.04	932,016.82	254,517.55	3,603,149.19
40031	Comanche	Oklahoma	124,098	15,338	11,351.50	19,359.50	5,586,258.65	3,698,241.61	8,149,024.46	9,687,528.30	4,027,203.69	23,668,226.92	15,273,786.95	8,281,479.14	28,876,806.62
40033	Cotton	Oklahoma	6,193	779	573.50	991.50	356,260.08	120,188.91	1,011,946.87	668,401.01	110,531.11	3,462,329.87	1,024,661.10	248,876.61	4,296,119.49
40035	Craig	Oklahoma	15,029	1,470	1,004.50	1,946.50	686,498.63	283,371.09	1,601,583.70	1,227,475.93	242,124.19	5,270,200.31	1,913,974.56	588,361.49	6,430,797.05
40037	Creek	Oklahoma	69,967	8,317	6,233.50	10,477.00	3,603,355.23	2,156,297.81	5,660,350.22	6,440,598.22	2,463,773.91	17,751,167.19	10,043,953.45	5,019,964.15	21,703,612.55
40039	Custer	Oklahoma	27,469	3,162	2,270.00	4,123.00	1,287,636.98	622,209.20	2,550,391.16	2,182,511.81	524,420.79	7,891,793.76	3,470,148.78	1,316,353.56	9,151,505.74
40041	Delaware	Oklahoma	41,487	4,235	2,964.00	5,624.50	2,118,673.00	1,086,605.02	3,615,674.64	3,612,309.31	1,032,535.66	10,455,964.03	5,730,982.30	2,463,374.97	13,042,353.33
40043	Dewey	Oklahoma	4,810	549	377.00	724.50	262,110.14	77,354.42	736,108.10	487,919.55	73,124.03	2,384,791.64	750,029.69	163,128.05	2,756,107.57
40045	Ellis	Oklahoma	4,151	443	299.00	586.00	213,145.81	55,071.56	589,510.12	353,749.64	63,761.56	1,982,396.93	566,895.45	133,289.53	2,423,686.45
40047	Garfield	Oklahoma	60,580	6,543	4,529.00	8,519.00	2,842,223.33	1,637,002.52	4,775,743.42	5,151,572.77	1,733,561.03	15,226,682.80	7,993,796.10	3,782,033.35	18,599,776.49
40049	Garvin	Oklahoma	27,576	3,643	2,781.50	4,420.50	1,629,196.33	883,515.33	2,881,590.21	3,031,775.30	871,276.31	9,518,359.55	4,660,971.63	2,019,167.80	11,507,320.66
40051	Grady	Oklahoma	52,431	6,626	5,040.00	8,158.50	2,806,090.45	1,633,082.09	4,561,932.70	4,980,256.39	1,498,339.00	15,036,107.31	7,786,346.84	3,617,002.68	18,460,949.87
40053	Grant	Oklahoma	4,527	444	298.50	590.00	218,651.96	60,023.49	607,066.88	433,962.25	67,331.32	2,558,728.78	652,614.21	142,026.03	2,953,130.48
40055	Greer	Oklahoma	6,239	718	498.00	934.50	332,726.80	112,355.26	801,929.31	591,578.19	94,119.20	3,391,721.61	924,304.99	233,702.58	3,958,473.71
40057	Harmon	Oklahoma	2,922	363	252.50	476.00	160,876.12	36,414.75	523,332.93	266,673.11	44,398.96	1,487,463.84	427,549.23	87,418.31	1,689,700.51
40059	Harper	Oklahoma	3,685	382	240.00	516.00	168,696.29	41,530.28	496,297.36	319,369.57	44,031.99	2,048,874.50	488,065.86	92,702.32	2,305,466.33
40061	Haskell	Oklahoma	12,769	1,630	1,204.00	2,030.50	755,102.86	330,389.77	1,620,317.78	1,304,498.62	225,112.95	5,162,232.53	2,059,601.48	618,313.07	6,173,693.89
40063	Hughes	Oklahoma	14,003	1,771	1,347.50	2,202.00	819,745.63	368,702.53	1,609,529.15	1,350,389.63	269,971.35	5,711,839.74	2,170,135.25	755,363.13	6,783,077.48
40065	Jackson	Oklahoma	26,446	3,251	2,348.00	4,100.00	1,311,732.43	609,270.75	2,490,054.99	2,289,492.05	571,405.39	7,910,583.12	3,601,224.48	1,353,650.76	9,693,564.41
40067	Jefferson	Oklahoma	6,472	852	630.00	1,059.00	403,893.24	145,138.95	948,491.61	691,561.55	114,774.74	3,760,483.67	1,095,454.79	290,496.02	4,336,854.61
40069	Johnston	Oklahoma	10,957	1,483	1,154.00	1,836.00	680,337.38	283,666.05	1,398,513.96	1,064,595.63	218,764.62	4,657,109.28	1,744,933.01	587,754.76	5,271,477.04
40071	Kay	Oklahoma	46,562	4,611	3,182.00	6,070.00	2,072,082.68	1,106,899.17	3,693,679.73	3,714,906.95	1,112,857.52	12,061,891.07	5,786,989.63	2,490,688.91	14,930,883.64
40073	Kingfisher	Oklahoma	15,034	1,794	1,301.00	2,276.50	738,893.95	335,948.87	1,491,989.28	1,344,236.82	322,788.62	5,272,933.56	2,083,130.78	741,701.51	6,257,143.64
40075	Kiowa	Oklahoma	9,446	1,134	804.00	1,470.50	536,303.04	204,521.67	1,398,568.93	855,918.48	160,929.37	4,061,740.79	1,392,221.52	423,156.24	4,825,448.25
40077	Latimer	Oklahoma	11,154	1,452	1,091.00	1,836.00	663,028.08	280,932.68	1,374,271.04	1,166,267.14	286,061.80	4,693,330.02	1,829,295.23	636,481.28	5,661,657.95
40079	Le Flore	Oklahoma	50,384	6,407	4,769.50	8,042.00	2,758,709.36	1,640,086.51	4,571,001.91	4,581,187.93	1,487,458.40	12,875,468.80	7,339,897.29	3,429,277.78	15,499,643.60
40081	Lincoln	Oklahoma	34,273	4,148	3,010.00	5,310.00	1,829,601.33	1,001,056.50	3,433,549.37	3,020,915.80	854,940.00	8,853,014.87	4,850,517.14	2,042,378.16	11,424,529.22
40083	Logan	Oklahoma	41,848	4,877	3,542.00	6,095.50	1,992,097.28	1,112,280.14	3,397,274.97	3,236,993.46	893,369.82	12,040,696.75	5,229,090.74	2,311,284.06	14,336,094.99
40085	Love	Oklahoma	9,423	1,270	979.50	1,573.50	580,900.29	248,320.86	1,264,358.83	1,083,725.97	196,903.20	4,024,744.73	1,664,626.26	495,844.29	4,933,161.65
40087	McClain	Oklahoma	34,506	4,500	3,494.00	5,560.50	1,829,587.51	1,040,614.90	3,150,053.16	3,142,921.41	881,791.72	10,146,673.30	4,972,508.92	2,206,375.82	11,935,568.49
40089	McCurtain	Oklahoma	33,151	4,549	3,406.00	5,729.00	1,975,710.72	1,034,251.36	3,631,292.48	3,270,974.77	971,305.40	8,784,461.98	5,246,685.49	2,369,061.78	11,096,034.81
40091	McIntosh	Oklahoma	20,252	2,546	1,919.00	3,184.00	1,291,428.00	646,593.41	2,392,826.93	2,287,191.95	496,740.17	7,121,716.17	3,578,619.95	1,352,288.15	8,614,785.03
40093	Major	Oklahoma	7,527	825	580.50	1,086.50	394,903.91	128,928.30	996,599.77	696,660.79	120,940.74	3,680,547.21	1,091,564.70	280,000.30	4,083,026.58
40095	Marshall	Oklahoma	15,840	2,153	1,654.50	2,657.00	1,041,097.54	521,323.77	2,008,268.57	1,818,171.03	445,046.92	6,081,432.56	2,859,268.57	1,118,052.32	7,301,111.29
40097	Mayes	Oklahoma	41,259	4,534	3,198.50	5,870.50	1,979,669.75	1,115,991.01	3,442,010.67	3,462,077.87	1,019,527.50	9,824,299.98	5,441,747.62	2,398,887.18	12,299,496.70
40099	Murray	Oklahoma	13,488	1,788	1,371.00	2,188.00	826,779.64	374,658.61	1,742,258.61	1,406,393.50	310,149.47	5,326,940.89	2,233,173.15	819,704.60	6,553,017.99
40101	Muskogee	Oklahoma	70,990	8,810	6,470.50	10,960.50	3,767,989.67	2,288,981.78	6,170,306.75	6,466,236.18	2,415,044.97	16,792,388.88	10,234,225.85	5,188,087.06	20,964,863.26

40103	Noble	Oklahoma	11,561	1,264	903.00	1,643.00	575,997.19	227,296.27	1,327,747.64	1,033,115.28	195,436.64	5,034,710.45	1,609,112.46	472,593.07	5,883,248.05
40105	Nowata	Oklahoma	10,536	1,065	705.50	1,413.00	494,393.16	183,075.75	1,182,866.22	854,608.59	137,078.15	4,005,435.46	1,349,001.75	344,705.48	4,840,072.90
40107	Okfuskee	Oklahoma	12,191	1,504	1,116.00	1,899.00	684,027.04	296,300.96	1,596,016.81	1,151,788.07	227,963.58	5,018,547.08	1,835,815.11	587,306.15	6,004,296.87
40109	Oklahoma	Oklahoma	718,633	89,605	67,385.50	112,579.00	35,088,465.38	25,107,638.74	45,372,322.77	63,819,237.99	40,374,443.06	99,021,949.95	98,907,703.37	68,194,912.43	140,965,561.90
40111	Okmulgee	Oklahoma	40,069	4,963	3,723.00	6,121.00	2,138,896.76	1,205,752.42	3,819,285.91	3,606,365.41	1,075,440.12	10,752,072.45	5,745,262.18	2,672,134.45	12,940,041.07
40113	Osage	Oklahoma	47,472	5,001	3,701.00	6,383.50	2,238,889.22	1,281,022.02	3,929,904.28	3,816,149.92	1,099,304.90	10,309,757.44	6,055,039.14	2,659,367.91	13,384,166.20
40115	Ottawa	Oklahoma	31,848	3,023	2,043.00	4,056.50	1,317,121.49	621,882.42	2,548,028.28	2,190,044.18	519,031.61	6,562,924.60	3,507,165.67	1,348,358.97	8,251,465.07
40117	Pawnee	Oklahoma	16,577	1,827	1,348.00	2,358.00	838,206.39	372,235.03	1,663,683.11	1,418,011.17	313,202.38	5,128,755.35	2,256,217.56	794,686.56	6,310,394.30
40119	Payne	Oklahoma	77,350	8,471	6,145.00	10,810.00	3,115,537.86	1,853,693.87	4,979,495.50	5,117,116.38	1,658,891.42	12,958,394.56	8,232,654.24	3,899,203.35	16,214,929.74
40121	Pittsburg	Oklahoma	45,837	5,944	4,453.50	7,320.00	2,773,616.04	1,618,057.42	4,531,164.83	5,059,317.19	1,765,995.30	12,213,662.04	7,832,933.23	3,729,617.99	15,319,357.87
40123	Pontotoc	Oklahoma	37,492	4,937	3,867.50	6,105.50	2,038,807.35	1,166,170.64	3,403,441.55	3,667,022.20	1,063,986.50	10,413,196.16	5,705,829.55	2,473,621.10	12,492,261.36
40125	Pottawatomie	Oklahoma	69,442	8,895	6,613.00	11,026.00	3,669,378.71	2,281,626.50	5,564,120.71	6,213,055.03	2,153,935.05	17,507,940.61	9,882,433.74	4,793,360.88	21,125,477.85
40127	Pushmataha	Oklahoma	11,572	1,556	1,168.00	1,909.00	764,862.88	345,423.38	1,768,849.34	1,314,605.79	253,982.09	5,016,693.56	2,079,468.67	688,955.20	6,013,631.62
40129	Roger Mills	Oklahoma	3,647	415	274.00	554.00	197,475.69	46,302.08	609,515.63	330,474.36	54,814.32	1,776,012.79	527,950.05	107,880.93	2,165,888.42
40131	Rogers	Oklahoma	86,905	9,705	7,337.00	12,177.50	3,929,854.52	2,511,238.87	6,315,002.77	7,002,575.80	2,796,955.25	17,788,348.42	10,932,430.31	5,694,869.55	21,814,106.37
40133	Seminole	Oklahoma	25,482	3,266	2,472.50	4,119.50	1,430,091.94	762,551.49	2,715,581.52	2,556,491.46	654,014.67	9,204,876.78	3,986,583.40	1,649,160.63	10,787,387.22
40135	Sequoyah	Oklahoma	42,391	5,113	3,730.50	6,555.00	2,146,632.68	1,184,097.58	3,515,892.12	3,597,112.86	943,158.92	10,188,284.96	5,743,745.54	2,478,399.72	13,228,416.25
40137	Stephens	Oklahoma	45,048	5,778	4,416.50	7,186.50	2,638,256.06	1,539,620.95	4,479,911.18	4,621,307.78	1,638,444.55	11,501,667.08	7,259,563.84	3,503,489.16	14,571,843.50
40139	Texas	Oklahoma	20,640	1,970	1,101.50	2,728.50	696,751.52	278,916.62	1,501,652.40	1,117,604.90	235,324.54	5,111,650.55	1,814,356.42	564,684.71	5,933,454.39
40141	Tillman	Oklahoma	7,992	989	706.50	1,277.50	434,174.29	168,287.60	996,558.03	732,142.38	139,775.38	3,371,065.57	1,166,316.67	325,143.35	3,874,633.22
40143	Tulsa	Oklahoma	603,403	72,146	54,336.00	89,666.00	28,303,070.19	20,174,468.30	37,170,351.55	50,206,412.89	30,845,570.56	75,896,857.34	78,509,483.08	53,576,504.67	110,692,148.20
40145	Wagoner	Oklahoma	73,085	8,789	6,606.00	11,156.50	3,523,816.53	2,172,686.65	5,498,588.22	5,940,362.78	2,023,842.18	15,965,282.88	9,464,179.31	4,619,851.27	19,642,166.50
40147	Washington	Oklahoma	50,976	5,359	3,832.00	6,887.00	2,470,304.77	1,373,718.10	4,301,350.67	4,485,087.03	1,535,344.53	12,427,482.78	6,955,391.79	3,230,356.75	14,923,860.91
40149	Washita	Oklahoma	11,629	1,378	958.50	1,781.00	616,760.22	242,330.44	1,347,005.87	1,078,049.71	225,313.64	4,564,536.25	1,694,809.93	525,316.92	5,369,961.90
40151	Woods	Oklahoma	8,878	878	576.00	1,166.00	408,051.34	129,393.05	1,024,850.88	691,120.38	104,183.27	3,514,673.89	1,099,171.72	265,476.46	3,882,556.87
40153	Woodward	Oklahoma	20,081	2,136	1,439.50	2,863.50	888,209.26	367,488.96	1,847,414.86	1,632,875.91	357,451.70	6,093,447.79	2,521,085.17	854,680.03	7,175,109.58
41001	Baker	Oregon	16,134	1,209	680.50	1,734.50	626,406.44	210,214.55	1,537,269.20	1,176,402.66	180,074.57	4,669,240.91	1,802,809.09	453,955.33	5,469,157.55
41003	Benton	Oregon	85,579	6,399	3,968.50	8,914.00	2,676,395.21	1,325,681.24	4,777,151.35	4,577,432.97	1,413,196.21	11,366,817.25	7,253,828.18	3,053,087.11	15,210,873.38
41005	Clackamas	Oregon	375,992	28,559	17,616.50	40,086.50	12,364,656.87	7,018,050.52	18,471,572.54	22,766,098.15	10,508,380.03	43,581,983.72	35,130,755.03	18,213,494.29	58,441,721.88
41007	Clatsop	Oregon	37,039	2,891	1,711.50	4,075.00	1,377,945.79	574,380.23	2,710,627.45	2,614,469.35	523,789.01	9,391,888.20	3,992,415.13	1,168,790.44	11,336,446.85
41009	Columbia	Oregon	49,351	3,810	2,331.50	5,433.00	1,637,236.46	742,867.30	2,984,915.12	2,850,859.52	681,014.83	10,219,882.91	4,488,095.98	1,631,714.28	12,014,848.20
41011	Coos	Oregon	63,043	5,265	3,113.50	7,635.50	2,759,665.65	1,332,306.80	4,720,763.44	5,154,052.47	1,327,412.31	14,538,049.82	7,913,718.12	2,956,034.48	18,009,262.73
41013	Crook	Oregon	20,978	1,621	941.50	2,301.50	826,215.84	311,136.01	1,865,002.00	1,441,461.75	246,356.67	5,767,537.32	2,267,677.58	648,799.37	6,975,126.46
41015	Curry	Oregon	22,364	1,896	1,016.50	2,697.50	1,177,450.28	449,378.54	2,336,299.47	2,207,316.27	428,246.00	7,216,587.88	3,384,766.55	1,039,666.00	8,630,214.37
41017	Deschutes	Oregon	157,733	12,246	6,826.50	17,382.50	5,422,898.85	2,701,102.05	8,848,767.66	9,566,568.79	3,424,321.76	23,078,818.76	14,989,467.64	6,643,159.76	29,685,327.82
41019	Douglas	Oregon	107,667	8,838	5,086.00	12,574.50	4,520,201.45	2,342,446.21	7,442,635.80	8,220,837.50	2,708,024.49	19,886,803.24	12,741,038.95	5,480,379.01	25,371,062.20
41021	Gilliam	Oregon	1,871	141	76.50	206.00	76,093.33	9,502.04	303,177.74	153,437.72	19,575.69	962,538.43	229,531.06	32,834.95	1,169,722.81
41023	Grant	Oregon	7,445	557	310.00	824.00	302,215.89	80,998.64	847,165.78	595,991.54	74,464.61	3,119,071.34	898,207.42	172,405.99	3,599,213.59
41025	Harney	Oregon	7,422	549	283.50	819.50	261,933.85	63,348.31	703,014.92	485,964.34	60,221.33	2,713,555.44	747,898.19	137,014.72	3,069,068.21
41027	Hood River	Oregon	22,346	1,726	1,046.50	2,406.00	687,984.50	270,407.35	1,467,545.29	1,086,096.35	213,819.15	4,133,208.08	1,774,080.85	542,221.22	4,984,057.20
41029	Jackson	Oregon	203,206	17,147	9,786.00	24,147.00	8,113,468.27	4,458,341.17	12,279,570.92	14,671,246.85	5,924,070.50	29,735,607.88	22,784,715.12	11,272,833.43	40,211,531.35
41031	Jefferson	Oregon	21,720	1,707	991.50	2,449.50	745,517.77	282,808.02	1,685,501.96	1,234,359.54	251,173.28	4,953,848.49	1,979,877.31	588,032.03	6,303,504.10
41033	Josephine	Oregon	82,713	7,203	4,096.00	10,254.50	3,831,260.42	1,877,435.02	6,416,071.07	7,103,356.71	2,190,554.05	18,557,664.83	10,934,617.13	4,258,000.56	22,492,865.72
41035	Klamath	Oregon	66,380	5,378	3,145.50	7,621.00	2,518,088.55	1,170,825.97	4,658,513.93	4,256,931.43	1,237,437.66	11,422,916.74	6,775,019.98	2,710,664.48	14,413,228.07
41037	Lake	Oregon	7,895	610	346.00	886.50	310,925.03	72,947.48	791,851.19	518,368.65	78,864.72	2,631,119.38	829,293.68	174,544.53	3,072,342.18
41039	Lane	Oregon	351,715	27,508	17,528.50	37,586.50	12,338,490.82	7,387,050.08	18,081,793.21	22,031,221.90	10,157,836.24	43,812,826.64	34,369,712.72	18,297,504.52	57,720,734.48
41041	Lincoln	Oregon	46,034	3,639	2,284.00	5,037.00	1,989,203.88	966,209.37	3,606,633.00	3,550,856.45	851,439.94	10,832,971.38	5,540,060.33	1,957,919.66	13,829,869.43
41043	Linn	Oregon	116,672	9,313	5,775.50	12,939.50	4,068,817.15	2,171,578.43	6,646,318.85	7,433,667.38	2,401,169.93	17,615,856.07	11,502,484.53	4,998,747.40	22,367,634.28
41045	Malheur	Oregon	31,313	2,395	1,312.50	3,425.50	1,003,582.77	395,143.86	2,025,435.62	1,682,359.78	309,395.69	6,156,031.74	2,685,942.54	763,663.89	7,295,835.34
41047	Marion	Oregon	315,335	25,052	14,373.00	35,343.00	9,958,408.30	5,198,155.52	14,951,450.24	17,814,979.77	6,887,243.67	37,392,866.46	27,773,388.07	12,707,087.85	50,223,073.20
41049	Morrow	Oregon	11,173	876	514.50	1,249.50	364,944.49	106,731.90	1,009,832.75	653,618.85	119,145.50	3,851,031.55	1,018,563.34	239,463.48	4,437,457.33
41051	Multnomah	Oregon	735,334	55,108	33,636.00	76,250.50	21,534,349.69	13,104,291.97	31,237,764.91	39,362,265.41	20,869,089.42	68,954,069.33	60,896,615.10	34,538,012.70	97,099,579.83
41053	Polk	Oregon	75,403	6,009	3,547.00	8,420.00	2,580,631.23	1,274,610.98	4,350,576.75	4,211,766.01	1,184,639.04	12,135,381.61	6,792,397.24	2,735,976.01	15,128,397.89
41055	Sherman	Oregon	1,765	135	76.00	197.50	69,632.01	9,284.10	280,259.08	130,346.71	20,923.55	994,913.46	199,978.72	32,316.67	1,065,932.17
41057	Tillamook	Oregon	25,250	1,980	1,146.50	2,756.00	1,036,987.29	420,970.62	2,172,538.12	1,809,697.50	355,866.63	5,958,324.57	2,846,684.79	900,208.73	7,551,236.98
41059	Umatilla	Oregon	75,889	5,957	3,404.00	8,324.50	2,403,071.51	1,166,325.83	4,430,339.49	4,170,132.91	1,124,198.24	12,046,073.61	6,573,204.42	2,485,925.62	15,013,375.09
41061	Union	Oregon	25,748	1,910	1,069.50	2,771.50	884,686.47	323,417.25	1,867,730.33	1,539,756.01	277,939.25	5,923,769.30	2,424,442.48	667,896.67	7,095,026.41
41063	Wallowa	Oregon	7,008	500	258.50	732.00	281,234.17	69,327.38	861,845.47	445,982.38	59,963.25	2,282,891.26	727,216.55	140,582.99	2,847,317.34
41065	Wasco	Oregon	25,213	1,944	1,165.50	2,706.00	921,857.35	386,609.74	1,890,418.47	1,805,671.37	344,340.23	7,075,108.37	2,727,528.71	847,061.86	8,445,010.22
41067	Washington	Oregon	529,710	41,103	23,865.00	57,919.00	15,321,362.73	8,429,099.79	23,128,215.34	28,519,273.72	13,501,135.39	49,624,859.01	43,840,636.45	23,055,109.04	68,405,935.45
41069	Wheeler	Oregon	1,441	108	56.00	162.00	69,204.66	6,321.89	305,876.68	118,015.13	9,208.27	852,289.67	187,219.79	17,090.90	1,076,626.37
41071	Yamhill	Oregon	99,193	7,773	4,485.50	10,910.50	3,171,780.64	1,585,258.92	5,448,289.89	5,446,180.52	1,602,088.79	14,344,723.17	8,617,961.16	3,405,231.59	18,357,403.50
42001	Adams	Pennsylvania	101,407	10,407	7,542.50	13,155.50	4,662,424.36	2,963,975.14	6,977,266.96	8,517,893.12	3,463,067.64	20,298,012.85	13,180,317.48	6,962,123.99	26,665,636.62
42003	Allegheny	Pennsylvania	1,223,348	116,504	79,615.00	150,748.00	54,190,050.85	35,747,406.91	71,616,909.97	107,267,002.30	64,971,977.58	156,088,090.20	161,457,053.20	104,120,860.90	220,076,381.10
42005	Armstrong	Pennsylvania	68,941	6,443	4,417.50	8,413.50	3,128,496.19	1,760,440.16	5,165,664.76	5,814,274.53	1,813,694.16	15,423,133.57	8,942,770.72	3,849,280.30	18,764,244.21
42007	Beaver	Pennsylvania	170,539	15,108	10,310.00	20,069.00	7,330,508.32	4,469,474.41	10,783,924.12	13,600,189.28	6,196,150.78	27,427,289.94	20,930,697.60	11,468,076.36	36,211,664.74

42009	Bedford	Pennsylvania	49,762	4,950	3,473.50	6,457.00	2,422,397.66	1,242,090.49	4,223,913.96	4,316,608.34	1,221,041.61	12,565,419.36	6,739,006.01	2,900,441.65	16,280,378.32
42011	Berks	Pennsylvania	411,442	38,893	26,962.50	51,413.00	16,579,377.14	10,678,208.46	23,689,561.07	30,933,525.98	16,356,687.22	53,399,981.23	47,512,903.12	28,085,102.38	73,841,933.73
42013	Blair	Pennsylvania	127,089	12,686	8,933.50	16,539.50	6,019,302.74	3,774,983.02	9,037,468.12	11,115,282.53	4,494,132.76	24,366,373.04	17,134,585.27	9,097,773.31	31,275,342.82
42015	Bradford	Pennsylvania	62,622	5,567	3,751.00	7,355.00	2,631,565.25	1,434,144.21	4,502,186.01	4,742,337.82	1,562,223.42	13,813,670.55	7,373,903.07	3,311,970.91	16,944,287.04
42017	Bucks	Pennsylvania	625,249	54,741	36,882.50	72,102.50	24,104,342.76	15,658,287.48	33,050,579.73	45,374,750.86	25,336,753.90	74,458,506.69	69,479,093.63	42,445,777.20	103,534,184.40
42019	Butler	Pennsylvania	183,862	17,226	11,861.50	22,764.00	7,653,049.75	4,714,700.96	11,186,970.38	13,906,228.74	6,253,761.01	27,173,630.33	21,559,278.49	11,807,275.83	36,426,505.89
42021	Cambria	Pennsylvania	143,679	13,894	9,405.50	18,345.50	6,847,015.69	4,205,602.07	10,451,470.66	12,527,813.06	5,324,407.93	26,963,500.66	19,374,828.75	10,111,089.87	34,919,550.31
42023	Cameron	Pennsylvania	5,085	479	330.00	625.50	251,919.02	65,654.95	788,188.11	452,942.18	72,275.48	2,194,978.82	704,861.19	157,114.46	2,513,553.04
42025	Carbon	Pennsylvania	65,249	5,873	3,885.00	7,875.00	2,814,665.15	1,567,592.94	4,744,526.14	5,040,299.88	1,608,162.38	12,856,887.60	7,854,965.03	3,636,554.32	16,319,286.43
42027	Centre	Pennsylvania	153,990	14,797	10,928.00	18,841.00	5,779,319.57	3,762,389.85	8,429,825.28	9,609,201.88	4,302,535.92	20,960,011.29	15,388,521.45	8,689,588.31	27,905,907.12
42029	Chester	Pennsylvania	498,886	47,845	32,739.50	62,172.00	19,670,070.56	12,946,230.47	27,290,713.12	39,992,710.08	22,934,885.36	68,001,452.92	59,662,780.65	37,597,465.27	92,440,750.32
42031	Clarion	Pennsylvania	39,988	3,657	2,383.00	4,847.50	1,683,405.85	847,145.10	3,070,409.88	2,870,431.85	703,714.91	9,356,149.56	4,553,837.70	1,781,725.37	10,988,834.02
42033	Clearfield	Pennsylvania	81,642	7,821	5,660.00	10,088.00	3,626,060.51	2,143,633.49	5,748,673.38	6,525,781.06	2,216,507.48	15,531,559.90	10,151,841.57	4,775,482.87	19,690,575.61
42035	Clinton	Pennsylvania	39,238	3,771	2,708.00	4,877.50	1,704,247.20	877,073.13	3,099,667.04	2,931,372.67	793,910.68	9,083,439.26	4,635,619.87	1,888,404.25	10,936,964.62
42037	Columbia	Pennsylvania	67,295	6,091	4,248.50	7,877.00	2,797,192.24	1,534,863.84	4,728,786.23	4,858,820.87	1,522,960.30	13,872,730.69	7,656,013.10	3,409,738.27	16,968,824.70
42039	Crawford	Pennsylvania	88,765	7,799	4,927.00	10,544.00	3,616,413.01	1,912,509.74	6,133,462.50	6,488,459.97	2,067,834.50	18,290,928.38	10,104,872.98	4,247,126.70	22,561,560.13
42041	Cumberland	Pennsylvania	235,406	23,655	17,754.00	30,112.00	10,572,729.51	7,201,187.35	15,001,592.37	19,551,795.08	10,107,148.21	36,704,043.86	30,124,524.59	18,625,582.04	47,576,538.00
42043	Dauphin	Pennsylvania	268,100	26,891	18,554.00	34,752.00	11,419,512.37	7,562,914.66	15,984,298.56	21,728,696.56	11,014,197.92	41,847,252.02	33,148,208.93	19,617,354.06	54,966,581.78
42045	Delaware	Pennsylvania	558,979	52,471	37,094.00	68,539.00	22,274,129.24	14,698,917.81	30,871,026.77	43,250,370.32	23,996,020.90	75,223,646.51	65,524,499.56	39,858,434.14	102,121,305.50
42047	Elk	Pennsylvania	31,946	2,993	1,952.50	3,982.50	1,452,507.47	686,382.00	2,800,126.06	2,618,507.89	653,173.49	8,526,568.11	4,071,015.37	1,552,012.15	10,432,872.14
42049	Erie	Pennsylvania	280,566	24,137	15,458.50	33,343.00	10,305,133.60	6,061,502.20	15,110,581.17	18,531,625.16	8,055,825.58	38,139,914.63	28,836,758.75	14,763,619.53	50,517,996.99
42051	Fayette	Pennsylvania	136,606	13,124	9,214.00	17,232.50	6,401,573.02	3,979,935.28	9,539,040.83	11,285,456.42	4,793,327.04	24,695,580.50	17,687,029.44	9,437,048.06	32,833,450.05
42053	Forest	Pennsylvania	7,716	661	433.00	901.00	337,448.00	100,661.31	987,943.01	631,178.87	96,770.90	3,296,259.15	968,626.87	223,739.17	3,802,657.82
42055	Franklin	Pennsylvania	149,618	15,650	11,269.50	19,748.50	6,981,867.14	4,559,495.35	10,181,379.84	12,665,340.49	5,766,573.52	26,816,252.77	19,647,207.63	11,178,165.17	34,997,991.74
42057	Fulton	Pennsylvania	14,845	1,511	1,056.50	1,939.50	671,415.32	300,605.19	1,482,739.54	1,269,674.08	266,871.76	6,006,376.93	1,941,089.41	636,516.89	6,745,829.20
42059	Greene	Pennsylvania	38,686	3,436	2,368.00	4,592.50	1,547,785.11	797,075.54	2,822,478.04	2,990,426.04	909,919.03	9,144,914.49	4,538,211.15	1,880,525.82	10,969,967.91
42061	Huntingdon	Pennsylvania	45,913	4,632	3,356.50	5,799.00	2,125,232.98	1,162,569.81	3,761,307.06	3,732,525.13	1,109,888.98	10,146,261.35	5,857,758.12	2,569,515.07	13,158,987.76
42063	Indiana	Pennsylvania	88,880	8,326	5,757.00	10,987.00	3,712,790.49	2,220,396.51	6,333,194.51	6,762,070.77	2,360,138.94	16,992,754.32	10,474,861.26	5,031,199.55	20,894,620.74
42065	Jefferson	Pennsylvania	45,200	4,282	2,852.00	5,744.00	2,039,756.77	1,029,904.08	3,517,626.98	3,440,773.43	1,026,710.17	9,739,252.07	5,480,530.21	2,221,697.74	12,296,338.73
42067	Juniata	Pennsylvania	24,636	2,533	1,818.00	3,226.50	1,148,496.38	549,075.13	2,504,116.15	2,017,098.12	418,493.58	7,477,524.25	3,165,594.50	1,109,591.92	8,455,674.54
42069	Lackawanna	Pennsylvania	214,437	18,103	11,762.50	24,215.50	8,467,031.83	4,949,173.85	12,383,310.33	15,388,425.36	6,830,959.79	30,386,603.07	23,855,457.19	12,846,147.12	40,619,390.92
42071	Lancaster	Pennsylvania	519,445	51,991	36,324.00	67,135.50	21,996,610.33	15,197,120.64	30,001,722.89	40,470,624.12	22,569,146.73	68,597,084.24	62,467,234.44	39,362,213.12	94,841,512.46
42073	Lawrence	Pennsylvania	91,108	8,229	5,449.50	10,755.00	3,998,856.49	2,293,192.58	6,433,082.86	7,378,435.08	2,661,960.96	20,029,486.05	11,377,291.57	5,476,147.98	24,408,302.34
42075	Lebanon	Pennsylvania	133,568	13,200	9,290.50	17,102.50	5,950,593.95	3,704,955.60	8,799,786.89	10,651,319.39	4,473,092.34	22,430,291.30	16,601,913.95	9,052,618.61	28,535,259.35
42077	Lehigh	Pennsylvania	349,497	32,387	21,735.00	42,462.00	13,879,364.39	8,765,971.13	19,578,740.09	26,459,783.24	13,371,563.10	47,347,864.28	40,339,147.62	23,074,698.20	64,663,981.27
42079	Luzerne	Pennsylvania	320,918	28,623	18,711.50	38,164.00	13,556,553.59	8,450,753.90	19,752,870.16	25,028,014.83	12,388,138.66	45,372,666.50	38,584,568.42	21,567,330.51	61,612,477.86
42081	Lycoming	Pennsylvania	116,111	10,822	7,390.50	14,070.00	4,935,472.37	2,991,365.14	7,558,281.93	9,017,120.79	3,489,709.45	21,306,537.45	13,952,593.16	7,198,525.00	27,189,948.77
42083	McKean	Pennsylvania	43,450	3,967	2,683.00	5,222.50	1,835,823.85	932,456.88	3,399,280.80	3,558,452.14	929,242.20	11,333,239.06	5,394,275.98	2,145,335.18	13,150,498.42
42085	Mercer	Pennsylvania	116,638	10,344	6,974.00	13,872.50	4,921,203.75	2,806,364.46	7,755,916.17	8,968,519.82	3,421,143.39	20,361,724.37	13,889,723.57	6,921,250.41	25,849,929.55
42087	Mifflin	Pennsylvania	46,682	4,857	3,490.50	6,179.50	2,288,808.72	1,299,239.72	3,784,506.19	4,046,505.31	1,324,715.44	11,547,825.88	6,335,314.02	3,103,448.01	14,766,145.99
42089	Monroe	Pennsylvania	169,842	14,475	9,544.00	19,370.00	6,043,430.13	3,535,029.56	9,370,293.19	10,552,524.31	4,528,156.97	23,593,891.32	16,595,954.44	8,681,615.40	31,296,121.36
42091	Montgomery	Pennsylvania	799,874	73,792	50,387.00	96,414.00	32,340,345.10	21,790,425.89	44,082,906.81	67,309,202.70	40,287,524.69	102,016,476.20	99,649,547.80	63,469,889.80	139,766,176.20
42093	Montour	Pennsylvania	18,267	1,720	1,182.50	2,256.50	823,640.14	369,530.00	1,830,393.88	1,606,209.60	480,637.94	5,057,137.93	2,429,849.74	942,539.71	5,988,323.22
42095	Northampton	Pennsylvania	297,735	26,072	17,851.50	35,138.50	11,654,102.03	7,364,206.70	16,856,628.39	21,500,188.74	10,795,762.14	40,890,462.98	33,154,290.77	19,140,909.98	55,150,140.53
42097	Northumberland	Pennsylvania	94,528	8,910	6,371.00	11,538.50	4,306,890.16	2,611,804.15	6,660,443.47	8,077,392.27	2,968,994.31	19,406,648.20	12,384,282.43	6,203,808.81	24,036,978.74
42099	Perry	Pennsylvania	45,969	4,662	3,322.50	5,998.00	2,024,729.06	1,095,850.12	3,568,202.35	3,388,080.56	903,782.10	10,048,457.83	5,412,809.63	2,310,508.23	12,053,591.26
42101	Philadelphia	Pennsylvania	1,526,006	136,618	93,050.50	180,492.00	53,833,350.54	35,094,270.82	72,184,440.27	105,850,060.80	65,145,954.51	156,157,686.00	159,683,411.30	101,656,598.20	223,223,412.60
42103	Pike	Pennsylvania	57,369	4,579	2,939.00	6,214.50	2,083,330.88	1,071,218.40	3,623,535.60	3,482,026.99	939,290.56	10,121,295.53	5,565,357.87	2,227,847.21	12,496,115.48
42105	Potter	Pennsylvania	17,457	1,604	1,086.50	2,125.50	813,660.45	334,782.42	1,806,968.69	1,404,490.09	299,581.12	4,870,114.02	2,218,150.54	751,567.38	6,038,235.57
42107	Schuylkill	Pennsylvania	148,289	13,815	9,309.00	18,151.00	6,618,117.07	4,054,626.87	10,141,163.75	11,952,764.61	5,090,032.93	24,040,984.04	18,570,827.78	10,072,224.17	32,926,711.45
42109	Snyder	Pennsylvania	39,702	3,908	2,715.00	5,048.50	1,712,285.88	940,262.21	3,176,096.57	3,172,623.66	785,227.61	9,915,924.86	4,884,909.54	1,952,341.84	12,136,081.50
42111	Somerset	Pennsylvania	77,742	7,607	5,317.50	9,872.50	3,730,696.53	2,140,191.98	5,976,589.09	6,675,379.39	2,354,921.07	16,136,016.88	10,406,075.93	4,989,415.85	20,566,796.97
42113	Sullivan	Pennsylvania	6,428	567	373.50	757.00	321,988.50	104,606.48	824,924.91	566,281.31	78,925.61	2,924,608.72	888,269.81	197,480.58	3,399,044.17
42115	Susquehanna	Pennsylvania	43,356	3,671	2,326.00	4,882.50	1,762,475.95	868,919.79	3,163,022.79	3,029,650.99	711,673.61	9,918,483.83	4,792,126.94	1,848,088.40	11,650,488.48
42117	Tioga	Pennsylvania	41,981	3,766	2,455.50	4,986.50	1,766,830.78	881,083.94	3,061,047.21	3,173,942.02	797,115.51	9,264,296.04	4,940,772.81	1,858,220.96	11,462,188.11
42119	Union	Pennsylvania	44,947	4,205	2,999.00	5,544.00	1,828,710.59	938,150.73	3,555,879.97	3,445,183.34	981,828.78	11,253,907.67	5,273,893.93	2,168,142.51	13,053,496.31
42121	Venango	Pennsylvania	54,984	4,973	3,234.00	6,696.00	2,398,034.67	1,244,562.81	4,185,308.14	4,288,524.85	1,233,273.78	12,821,467.57	6,686,559.53	2,807,350.75	16,019,572.86
42123	Warren	Pennsylvania	41,815	3,709	2,450.00	4,853.00	1,827,342.29	955,230.69	3,563,699.70	3,379,131.95	891,324.73	10,414,227.63	5,206,474.24	2,008,744.62	12,413,606.43
42125	Washington	Pennsylvania	207,820	18,994	12,935.50	25,224.50	9,034,534.11	5,622,908.85	13,200,916.08	17,118,362.85	7,795,925.66	33,450,538.56	26,152,896.96	13,746,444.36	45,220,110.83
42127	Wayne	Pennsylvania	52,822	4,229	2,702.00	5,678.00	2,135,714.67	1,059,207.29	3,740,897.47	3,814,308.04	1,032,685.34	11,667,483.28	5,950,022.71	2,305,729.21	14,415,152.74
42129	Westmoreland	Pennsylvania	365,169	36,112	25,466.00	45,970.00	17,815,473.47	12,295,070.19	24,496,330.51	34,017,648.99	17,840,719.19	60,937,045.31	51,833,122.46	31,229,360.44	81,432,590.06
42131	Wyoming	Pennsylvania	28,276	2,449	1,593.50	3,225.50	1,092,039.54	519,905.72	2,126,468.12	1,968,533.18	499,405.57	6,641,016.70	3,060,572.72	1,106,315.20	7,926,825.09
42133	York	Pennsylvania	434,972	44,123	31,320.00	56,659.50	18,822,391.03	12,796,777.54	25,542,436.11	33,857,700.16	18,936,503.40	59,318,685.01	52,680,091.18	33,570,310.09	80,886,420.24
44001	Bristol	Rhode Island	49,875	2,237	973.00	3,518.50	1,043,456.63	322,417.38	2,245,034.74	1,964,576.89	297,477.28	7,993,946.02	3,008,033.52	653,655.53	9,626,240.10
44003	Kent	Rhode Island	166,158	7,933	3,378.50	12,439.50	3,582,067.76	1,460,505.17	6,153,601.46	6,996,400.14	1,848,536.61	20,753,263.88	10,578,467.90	3,477,287.34	24,487,239.12
44005	Newport	Rhode Island	82,888	3,856	1,404.00	6,150.50	1,814,302.42	519,782.57	3,597,143.87	3,489,884.59	696,534.78	9,825,488.63	5,304,187.01	1,333,885.50	12,407,644.97

44007	Providence	Rhode Island	626,667	30,040	13,280.50	49,555.00	12,483,791.84	5,208,510.30	20,698,633.74	23,524,134.84	8,442,349.34	44,322,133.85	36,007,926.68	14,371,224.95	63,105,587.99
44009	Washington	Rhode Island	126,979	6,166	2,611.00	9,799.00	2,781,685.67	1,030,338.10	5,206,418.42	5,155,005.06	1,166,710.24	14,287,616.59	7,936,690.73	2,463,662.24	17,860,216.33
45001	Abbeville	South Carolina	25,417	1,846	1,100.50	2,628.00	852,922.20	314,726.04	1,869,035.58	1,519,244.42	265,839.78	5,726,489.47	2,372,166.61	675,223.64	6,877,518.40
45003	Aiken	South Carolina	160,099	11,683	7,261.00	16,305.00	5,110,198.92	2,703,254.84	8,021,664.74	9,972,208.75	3,733,202.17	23,740,441.50	15,082,407.67	6,971,845.90	29,264,742.34
45005	Allendale	South Carolina	10,419	731	423.00	1,073.50	305,157.45	90,780.41	819,648.02	582,606.92	97,880.22	3,280,092.07	887,764.37	209,108.07	3,791,512.65
45007	Anderson	South Carolina	187,126	13,959	8,280.00	19,882.50	6,051,406.03	3,399,215.67	9,427,598.66	10,681,662.26	3,855,219.96	25,251,885.47	16,733,068.29	7,807,391.28	32,092,825.82
45009	Bamberg	South Carolina	15,987	1,146	664.50	1,675.00	526,544.53	166,971.37	1,362,631.20	987,006.80	121,560.34	5,061,522.84	1,513,551.33	334,624.50	6,084,537.30
45011	Barnwell	South Carolina	22,621	1,649	946.50	2,362.50	692,333.73	254,839.55	1,575,770.82	1,164,675.24	215,247.97	5,021,602.00	1,857,008.96	541,557.69	6,109,501.65
45013	Beaufort	South Carolina	162,233	11,329	5,502.00	16,723.50	5,523,815.46	2,514,673.22	9,021,394.21	10,263,257.47	3,360,299.43	22,545,200.21	15,787,072.93	6,512,152.89	29,295,861.29
45015	Berkeley	South Carolina	177,843	13,051	7,432.50	19,015.50	4,874,251.50	2,335,909.16	8,055,568.95	8,534,173.52	2,994,830.23	20,835,641.44	13,408,425.02	5,898,610.96	26,923,438.96
45017	Calhoun	South Carolina	15,175	1,092	660.00	1,554.50	485,945.90	163,095.62	1,197,822.76	897,020.53	171,038.03	4,051,482.10	1,382,966.43	375,240.94	4,715,810.62
45019	Charleston	South Carolina	350,209	24,406	12,734.50	35,771.50	10,103,404.57	5,061,499.88	15,872,936.38	18,217,901.08	7,023,941.45	34,968,494.52	28,321,305.65	12,654,221.84	47,466,370.75
45021	Cherokee	South Carolina	55,342	4,126	2,616.00	5,667.50	1,698,920.86	805,545.32	3,096,819.10	2,791,145.01	674,016.98	8,719,366.48	4,490,065.87	1,743,485.51	10,773,277.46
45023	Chester	South Carolina	33,140	2,430	1,487.50	3,461.00	1,035,065.12	431,393.05	1,997,115.25	1,766,993.72	390,869.62	6,362,818.03	2,802,058.84	950,775.23	8,047,696.46
45025	Chesterfield	South Carolina	46,734	3,396	1,983.00	4,834.00	1,442,336.60	646,161.80	2,703,768.01	2,406,490.80	543,709.53	8,183,632.99	3,848,827.40	1,361,373.86	10,163,181.54
45027	Clarendon	South Carolina	34,971	2,555	1,433.00	3,730.50	1,194,984.01	521,813.87	2,384,455.90	2,106,329.93	386,112.35	8,044,932.47	3,301,313.94	1,016,286.01	9,742,163.31
45029	Colleton	South Carolina	38,892	2,795	1,572.50	4,089.00	1,216,672.47	507,847.06	2,384,203.86	2,088,384.12	388,960.42	7,400,442.68	3,305,056.60	1,029,761.29	9,070,661.18
45031	Darlington	South Carolina	68,681	5,111	2,901.50	7,307.00	2,188,860.85	1,023,638.36	4,103,009.24	3,981,427.12	1,141,541.23	11,592,494.45	6,170,287.97	2,388,347.83	14,242,262.03
45033	Dillon	South Carolina	32,062	2,557	1,459.00	3,662.50	1,038,656.34	398,424.64	2,219,018.30	1,728,203.32	313,653.82	6,660,941.38	2,766,859.66	846,792.50	8,208,005.31
45035	Dorchester	South Carolina	136,555	9,871	5,467.00	14,147.50	3,648,567.76	1,711,847.36	6,386,264.93	5,916,392.84	1,735,413.74	15,248,080.90	9,564,960.59	3,808,275.77	19,515,250.74
45037	Edgefield	South Carolina	26,985	1,884	1,068.50	2,659.00	803,546.46	319,213.33	1,700,569.03	1,387,495.20	231,591.13	6,415,529.44	2,191,041.66	605,640.74	7,407,202.04
45039	Fairfield	South Carolina	23,956	1,758	1,118.00	2,485.50	831,311.62	326,172.39	2,023,176.58	1,557,155.92	346,446.40	6,222,215.20	2,388,467.54	761,497.32	7,273,973.64
45041	Florence	South Carolina	136,885	10,268	6,032.50	14,777.50	4,208,651.86	2,115,569.35	6,850,052.61	7,580,774.39	2,415,237.78	20,164,813.46	11,789,426.25	4,995,451.33	25,515,011.31
45043	Georgetown	South Carolina	60,158	4,474	2,453.00	6,452.00	2,225,557.11	1,011,462.49	3,875,534.39	4,196,731.28	1,098,781.80	11,956,835.86	6,422,288.39	2,325,816.54	14,895,316.37
45045	Greenville	South Carolina	451,225	34,049	21,303.50	46,884.00	13,734,807.69	8,402,579.83	19,872,020.28	24,818,973.22	11,897,389.84	47,265,463.81	38,553,780.91	21,411,966.69	64,722,518.68
45047	Greenwood	South Carolina	69,661	5,117	2,927.00	7,262.50	2,161,913.19	1,073,908.13	3,667,281.69	3,895,965.12	1,096,960.25	11,209,686.47	6,057,878.31	2,235,027.28	13,654,621.97
45049	Hampton	South Carolina	21,090	1,490	838.50	2,139.00	620,787.05	207,442.45	1,475,211.34	1,107,354.90	202,216.27	4,746,112.57	1,728,141.94	469,493.00	5,388,217.70
45051	Horry	South Carolina	269,291	20,426	11,481.50	29,118.50	9,549,163.63	5,084,669.87	14,905,931.01	16,628,944.22	6,888,635.44	34,191,792.71	26,178,107.85	13,144,806.55	46,709,321.72
45053	Jasper	South Carolina	24,777	1,770	992.50	2,630.50	674,624.93	240,980.23	1,562,458.53	1,117,056.90	207,338.86	4,616,873.86	1,791,681.83	516,968.96	5,450,225.36
45055	Kershaw	South Carolina	61,697	4,413	2,555.00	6,140.00	1,889,096.18	864,375.62	3,273,407.82	3,360,299.86	842,300.78	11,094,634.03	5,249,396.03	2,011,014.53	13,576,189.88
45057	Lancaster	South Carolina	76,652	5,588	3,250.50	7,850.50	2,415,113.59	1,195,354.32	4,310,472.91	4,467,888.14	1,142,528.58	13,602,083.81	6,883,001.73	2,514,255.72	16,369,381.32
45059	Laurens	South Carolina	66,537	4,895	3,024.50	6,875.50	2,127,595.02	1,013,786.95	3,998,786.94	3,725,765.33	996,189.43	10,600,261.53	5,853,360.35	2,334,569.97	13,338,282.10
45061	Lee	South Carolina	19,220	1,373	781.00	1,941.50	590,164.20	205,053.75	1,377,498.76	979,966.24	172,893.43	4,309,336.12	1,570,130.44	410,423.77	5,152,396.77
45063	Lexington	South Carolina	262,391	19,297	11,519.50	26,649.00	7,923,002.14	4,328,827.48	12,669,700.84	13,366,275.49	5,465,500.05	29,639,988.86	21,289,277.64	10,652,790.19	39,257,543.44
45065	McCormick	South Carolina	10,233	716	410.00	1,034.00	405,829.74	119,621.40	1,042,222.72	738,217.46	106,480.28	3,452,849.25	1,144,047.19	248,721.45	4,059,805.18
45067	Marion	South Carolina	33,062	2,535	1,381.50	3,665.50	1,104,479.65	438,543.04	2,311,304.00	1,790,157.12	339,795.45	6,689,527.97	2,894,636.77	861,413.30	7,849,245.58
45069	Marlboro	South Carolina	28,933	2,098	1,178.00	2,973.00	883,862.86	329,376.75	1,913,364.51	1,604,574.65	299,489.68	6,528,287.17	2,488,437.50	667,875.83	7,956,626.08
45071	Newberry	South Carolina	37,508	2,774	1,659.00	3,813.00	1,220,839.17	554,261.12	2,405,759.81	2,258,683.32	467,311.34	7,911,517.86	3,479,522.49	1,113,650.27	9,671,233.19
45073	Oconee	South Carolina	74,273	5,491	3,291.50	7,772.00	2,655,239.62	1,387,533.78	4,497,786.20	4,987,089.51	1,495,004.61	12,999,848.78	7,642,329.13	3,129,391.28	16,091,761.65
45075	Orangeburg	South Carolina	92,501	6,783	4,036.50	9,457.00	2,942,866.31	1,416,050.71	5,106,423.01	5,093,590.01	1,641,128.52	12,310,054.48	8,036,456.32	3,543,095.61	15,806,374.96
45077	Pickens	South Carolina	119,224	8,687	5,292.50	12,102.50	3,613,031.95	1,839,825.61	5,955,889.45	6,084,590.18	1,997,003.32	14,794,906.03	9,697,622.13	4,401,991.98	19,802,914.77
45079	Richland	South Carolina	384,504	27,279	16,977.00	38,577.00	10,111,474.66	5,804,032.95	15,878,713.02	17,995,335.23	7,684,623.69	38,160,283.16	28,106,809.89	13,731,228.44	48,835,380.22
45081	Saluda	South Carolina	19,875	1,476	895.50	2,106.50	681,024.69	241,542.50	1,589,505.32	1,145,266.34	172,754.26	4,887,983.19	1,826,291.02	462,125.15	5,443,763.03
45083	Spartanburg	South Carolina	284,307	21,271	13,515.50	30,010.50	8,840,367.36	5,218,209.17	13,583,216.34	15,784,042.13	6,821,822.50	32,552,742.74	24,624,409.49	12,693,831.17	43,942,651.41
45085	Sumter	South Carolina	107,456	7,933	4,435.00	11,382.50	3,175,102.05	1,577,246.72	5,602,028.39	5,506,812.91	1,587,752.53	16,991,248.97	8,681,914.96	3,445,828.49	19,972,238.47
45087	Union	South Carolina	28,961	2,109	1,245.50	2,937.00	955,881.72	374,883.05	2,049,967.96	1,654,561.24	316,478.94	5,843,225.18	2,610,442.96	810,066.78	7,210,514.51
45089	Williamsburg	South Carolina	34,423	2,504	1,310.00	3,646.50	1,077,063.45	423,772.70	2,108,354.59	1,798,428.76	363,763.71	6,559,865.36	2,875,492.20	861,861.85	8,044,101.24
45091	York	South Carolina	226,073	16,469	10,042.00	23,324.50	6,416,507.77	3,661,671.88	10,131,240.03	11,060,452.96	4,416,033.55	24,444,157.43	17,476,960.73	8,693,263.03	32,831,925.31
46003	Aurora	South Dakota	2,710	132	21.50	242.00	64,671.00	2,690.82	310,851.10	119,470.33	3,361.18	830,554.67	184,141.33	6,983.38	990,792.55
46005	Beadle	South Dakota	17,398	876	195.00	1,589.50	398,950.37	35,320.71	1,101,287.38	736,670.29	36,234.08	3,317,994.53	1,135,620.66	76,787.41	4,090,207.65
46007	Bennett	South Dakota	3,431	170	20.00	341.00	63,606.70	1,383.17	293,021.04	102,596.52	2,044.06	484,298.72	166,203.22	3,650.25	830,816.37
46009	Bon Homme	South Dakota	7,070	332	80.00	587.50	160,702.49	16,363.41	552,144.03	272,387.02	15,131.44	1,824,264.75	433,089.50	34,238.08	2,136,334.64
46011	Brookings	South Dakota	31,965	1,659	490.00	2,792.50	599,967.05	109,472.60	1,424,603.37	1,016,802.29	89,710.99	4,962,075.48	1,616,769.34	213,581.09	5,804,613.33
46013	Brown	South Dakota	36,531	1,900	399.00	3,455.50	829,395.04	117,508.64	1,871,353.66	1,443,740.45	95,153.14	5,449,620.06	2,273,135.49	264,454.98	6,811,516.74
46015	Brule	South Dakota	5,255	238	32.50	445.50	108,098.18	3,007.19	400,542.98	163,824.57	4,597.28	1,321,469.33	271,922.75	8,394.10	1,560,916.23
46017	Buffalo	South Dakota	1,912	93	9.00	191.00	26,185.21	704.15	153,903.02	39,449.08	1,290.81	262,529.27	65,634.29	2,089.27	429,900.83
46019	Butte	South Dakota	10,110	445	38.50	922.00	199,115.62	5,008.02	682,229.49	321,432.43	5,896.73	2,152,492.85	520,548.05	12,596.73	2,769,677.35
46021	Campbell	South Dakota	1,466	71	9.00	141.50	39,502.25	669.50	236,941.61	72,984.27	1,397.36	454,260.92	112,486.52	2,508.21	683,373.54
46023	Charles Mix	South Dakota	9,129	440	85.50	781.50	185,905.88	14,687.49	601,751.41	319,871.49	13,098.23	1,918,416.85	505,777.36	29,968.71	2,300,768.66
46025	Clark	South Dakota	3,691	192	49.50	340.00	95,717.35	5,927.97	397,888.36	139,062.28	7,626.75	1,002,971.77	234,779.63	13,625.63	1,378,903.13
46027	Clay	South Dakota	13,864	665	200.00	1,097.50	244,893.38	30,869.34	740,789.15	356,634.84	28,301.13	2,106,437.38	601,528.22	64,135.46	2,497,324.06
46029	Codington	South Dakota	27,227	1,462	412.50	2,632.00	617,494.01	96,030.54	1,487,192.21	1,074,777.09	88,530.91	4,650,280.54	1,692,271.10	198,930.67	5,478,174.80
46031	Corson	South Dakota	4,050	214	27.00	413.50	72,357.14	2,333.54	333,645.78	114,396.23	3,633.59	898,233.65	186,753.36	6,435.24	1,153,881.26
46033	Custer	South Dakota	8,216	380	37.00	738.50	206,774.79	4,488.76	701,338.05	347,488.02	6,108.53	1,943,790.11	554,262.80	10,572.18	2,298,237.25
46035	Davison	South Dakota	19,504	915	185.50	1,635.00	395,088.34	48,550.89	1,042,708.24	638,702.93	35,593.87	2,975,002.52	1,033,791.27	88,593.02	3,483,163.29
46037	Day	South Dakota	5,710	307	76.50	549.50	160,585.99	12,211.03	549,198.22	308,645.99	12,730.82	1,745,503.58	469,231.98	26,480.94	1,897,707.21

46039	Deuel	South Dakota	4,364	246	80.50	413.00	119,507.60	11,128.27	452,561.74	245,735.85	16,719.60	1,675,905.71	365,243.45	29,707.29	1,872,440.90
46041	Dewey	South Dakota	5,301	275	39.00	525.50	105,789.95	4,150.01	405,076.09	142,652.94	5,938.61	1,097,793.64	248,442.89	10,346.94	1,451,200.85
46043	Douglas	South Dakota	3,002	145	35.00	271.50	70,579.75	3,567.15	273,702.03	134,825.08	6,356.74	1,017,134.02	205,404.83	11,577.54	1,094,747.41
46045	Edmunds	South Dakota	4,071	207	26.50	393.00	104,974.30	2,549.40	437,406.22	180,006.20	5,508.45	1,133,460.00	284,980.50	9,024.23	1,355,633.49
46047	Fall River	South Dakota	7,094	317	36.50	613.50	177,851.67	6,581.04	671,904.55	366,563.92	7,646.43	2,368,834.01	544,415.58	16,492.81	2,953,541.02
46049	Faulk	South Dakota	2,364	121	20.00	229.00	60,110.67	1,846.76	286,636.30	94,204.23	3,107.50	637,815.47	154,314.89	6,056.42	790,538.12
46051	Grant	South Dakota	7,356	415	111.50	700.50	190,026.25	20,803.87	609,852.64	341,960.51	23,241.86	1,851,540.48	531,986.76	52,819.97	2,337,141.81
46053	Gregory	South Dakota	4,271	187	35.50	352.00	99,752.84	4,804.17	417,891.04	187,413.53	5,494.81	1,308,572.29	287,166.37	11,423.98	1,524,477.20
46055	Haakon	South Dakota	1,937	87	8.00	174.00	49,191.36	546.22	249,476.30	84,762.33	1,306.11	523,484.05	133,953.69	1,826.19	751,824.71
46057	Hamlin	South Dakota	5,903	332	70.50	576.50	144,546.53	10,646.32	545,426.74	232,060.47	13,423.70	1,560,095.62	376,607.00	26,985.09	1,842,175.56
46059	Hand	South Dakota	3,431	168	22.00	306.00	93,242.18	2,952.65	413,781.64	201,846.17	3,958.63	1,365,985.10	295,088.35	7,002.64	1,633,744.18
46061	Hanson	South Dakota	3,331	175	48.00	310.50	72,069.26	4,380.68	275,881.42	119,231.05	6,750.92	773,159.13	191,300.31	14,375.25	885,819.95
46063	Harding	South Dakota	1,255	53	3.50	115.00	26,614.19	114.41	176,748.29	46,132.30	456.21	266,872.65	72,746.49	764.20	428,911.59
46065	Hughes	South Dakota	17,022	766	88.50	1,473.50	323,489.14	15,052.48	933,959.18	517,030.50	16,585.79	2,529,047.37	840,519.64	37,409.87	3,219,529.36
46067	Hutchinson	South Dakota	7,343	358	98.50	654.50	190,780.42	22,059.79	630,938.01	300,418.98	19,652.80	1,855,318.07	491,199.41	48,401.85	2,264,841.39
46069	Hyde	South Dakota	1,420	63	7.00	126.00	31,062.84	335.37	162,406.71	54,775.99	1,245.50	470,936.19	85,838.83	1,729.89	592,502.65
46071	Jackson	South Dakota	3,031	151	16.50	295.00	58,101.83	1,055.53	280,730.62	70,361.29	1,876.34	651,185.82	128,463.12	4,040.17	764,710.12
46073	Jerauld	South Dakota	2,071	108	23.50	190.00	59,952.48	2,659.31	264,073.04	109,134.44	4,598.46	763,060.87	169,086.92	7,957.51	954,445.93
46075	Jones	South Dakota	1,006	46	3.50	95.00	24,157.69	54.62	154,096.03	33,129.00	280.28	302,250.21	57,286.69	366.90	382,467.22
46077	Kingsbury	South Dakota	5,148	260	53.50	472.00	132,027.86	8,509.85	463,081.18	225,249.98	10,155.91	1,333,746.47	357,277.84	22,224.06	1,642,431.48
46079	Lake	South Dakota	11,200	571	151.50	980.50	271,223.27	34,193.21	830,827.14	430,598.63	26,021.26	2,092,603.94	701,821.91	70,408.22	2,624,216.64
46081	Lawrence	South Dakota	24,097	1,061	96.50	2,096.00	511,100.40	24,600.58	1,421,653.67	832,517.17	16,304.94	4,741,599.24	1,343,617.57	40,068.92	5,395,263.59
46083	Lincoln	South Dakota	44,828	2,410	755.00	4,058.50	824,537.27	170,694.47	1,858,233.42	1,377,674.77	174,792.91	5,394,379.17	2,202,212.05	399,267.96	6,500,973.06
46085	Lyman	South Dakota	3,755	172	16.50	343.00	65,608.94	1,071.59	254,795.74	146,570.48	1,709.52	1,420,908.44	212,179.42	2,981.38	1,669,378.43
46087	McCook	South Dakota	5,618	284	63.50	507.50	136,077.32	9,603.36	525,014.72	213,996.11	11,778.96	1,326,738.56	350,073.43	25,878.20	1,701,608.84
46089	McPherson	South Dakota	2,459	128	20.50	230.00	72,675.97	2,157.17	313,172.67	133,977.73	4,257.85	988,162.96	206,653.70	6,929.60	1,244,373.28
46091	Marshall	South Dakota	4,656	252	43.00	449.00	124,922.52	6,449.58	535,411.24	190,327.30	8,275.03	1,242,910.58	315,249.81	14,854.75	1,611,190.45
46093	Meade	South Dakota	25,434	1,140	102.50	2,258.00	472,758.66	16,428.71	1,342,790.99	788,279.10	17,870.06	3,777,183.88	1,261,037.77	34,074.85	4,670,574.87
46095	Mellette	South Dakota	2,048	99	13.00	189.00	37,012.24	907.63	192,722.11	55,066.10	1,560.90	394,864.75	92,078.34	2,662.02	517,353.48
46097	Miner	South Dakota	2,389	119	25.00	214.00	62,408.03	2,083.68	270,071.28	111,950.13	3,842.78	735,515.92	174,358.16	7,137.50	924,692.94
46099	Minnehaha	South Dakota	169,468	9,137	3,108.00	15,598.00	3,468,748.16	894,912.42	6,451,282.60	5,994,724.35	1,046,240.61	18,249,249.04	9,463,472.51	2,224,817.53	22,716,628.61
46101	Moody	South Dakota	6,486	356	103.00	609.50	144,464.58	15,207.77	454,036.59	277,425.29	17,964.07	1,746,294.94	421,889.87	39,224.43	1,945,297.23
46103	Pennington	South Dakota	100,948	4,662	420.00	9,592.50	1,951,379.30	102,269.59	4,452,071.79	3,247,815.73	97,451.19	11,499,613.65	5,199,195.03	213,014.41	14,789,750.01
46105	Perkins	South Dakota	2,982	130	10.50	276.00	68,106.77	901.16	316,737.60	102,021.85	1,259.30	577,487.35	170,128.62	2,615.91	953,994.60
46107	Potter	South Dakota	2,329	114	18.00	215.00	67,078.58	2,114.05	336,934.21	121,198.32	3,378.08	999,313.63	188,276.90	6,040.45	1,224,994.14
46109	Roberts	South Dakota	10,149	583	174.00	994.00	254,690.10	34,765.74	783,145.88	443,821.02	29,366.18	2,214,261.70	698,511.13	79,897.49	2,757,737.68
46111	Sanborn	South Dakota	2,355	114	20.50	214.50	57,536.41	2,593.27	270,979.19	100,368.60	3,400.59	767,047.10	157,905.01	6,632.96	970,965.90
46113	Shannon	South Dakota	13,586	688	79.00	1,337.50	188,476.70	11,628.92	608,758.14	272,616.70	13,227.67	1,543,224.53	461,093.41	26,723.65	1,890,203.43
46115	Spink	South Dakota	6,415	327	70.50	596.00	161,566.34	12,230.26	579,510.60	302,261.28	14,984.83	2,222,418.07	463,827.62	28,481.47	2,596,220.92
46117	Stanley	South Dakota	2,966	137	17.50	264.50	58,380.78	1,683.68	266,351.42	105,455.38	2,262.46	699,768.38	163,836.16	4,624.07	916,323.91
46119	Sully	South Dakota	1,373	64	7.00	128.00	31,617.73	313.60	173,759.28	57,900.67	989.04	419,810.41	89,518.40	1,465.13	606,071.68
46121	Todd	South Dakota	9,612	495	59.50	921.00	148,953.36	9,452.04	478,011.07	205,958.06	8,856.00	1,344,706.80	354,911.41	18,073.48	1,615,637.43
46123	Tripp	South Dakota	5,644	252	28.50	472.00	128,780.61	4,065.19	481,180.10	210,366.62	4,662.89	1,423,151.08	339,147.23	10,071.02	1,683,626.94
46125	Turner	South Dakota	8,347	415	94.50	715.00	199,785.36	20,284.86	668,879.20	426,248.65	25,658.72	2,551,295.74	626,034.02	54,028.26	2,999,008.59
46127	Union	South Dakota	14,399	715	214.50	1,246.50	306,195.99	45,930.77	888,474.77	531,094.07	52,270.65	2,942,440.61	837,290.07	115,629.19	3,379,402.36
46129	Walworth	South Dakota	5,438	271	28.50	522.50	148,780.43	4,117.53	474,699.10	257,937.22	5,172.12	1,719,827.03	406,717.66	9,764.51	2,176,267.79
46135	Yankton	South Dakota	22,438	1,054	276.00	1,868.50	482,372.21	70,494.51	1,311,318.60	838,139.19	59,588.88	4,293,916.28	1,320,511.40	147,573.44	4,899,477.34
46137	Ziebach	South Dakota	2,801	134	10.50	273.00	42,798.69	514.14	190,119.21	48,275.44	1,366.80	318,320.04	91,074.13	2,195.23	487,316.20
47001	Anderson	Tennessee	75,129	5,559	3,542.50	7,777.00	2,562,877.47	1,285,325.25	4,413,854.07	5,137,304.46	1,783,558.80	14,366,046.90	7,700,181.93	3,271,454.22	17,463,827.08
47003	Bedford	Tennessee	45,058	3,199	1,813.50	4,586.50	1,297,321.83	565,493.99	2,530,712.92	2,182,014.10	441,478.58	7,675,972.93	3,479,335.93	1,136,424.15	9,317,511.96
47005	Benton	Tennessee	16,489	1,084	612.50	1,627.50	551,488.42	167,123.66	1,207,650.59	972,261.30	116,813.30	4,629,567.81	1,523,749.72	358,966.49	5,418,452.48
47007	Bledsoe	Tennessee	12,876	888	526.00	1,263.50	395,196.49	122,048.94	980,814.20	624,117.62	87,078.70	3,026,908.88	1,019,314.11	239,383.11	3,613,488.74
47009	Blount	Tennessee	123,010	9,097	5,768.00	12,628.00	4,123,203.90	2,206,319.18	6,859,197.02	7,404,978.80	2,611,268.71	18,362,278.24	11,528,182.70	5,320,501.96	23,648,748.06
47011	Bradley	Tennessee	98,963	6,952	4,064.50	9,827.00	2,951,173.43	1,462,294.96	5,394,073.22	5,014,268.97	1,479,769.68	13,461,179.73	7,965,442.40	3,222,959.68	17,449,294.21
47013	Campbell	Tennessee	40,716	3,043	1,875.50	4,234.50	1,419,278.30	658,965.07	2,724,476.31	2,532,043.27	550,156.26	8,190,160.11	3,951,321.57	1,281,695.63	10,358,611.19
47015	Cannon	Tennessee	13,801	955	534.00	1,373.00	421,061.82	120,389.84	1,068,620.09	684,491.14	96,193.73	3,279,005.10	1,105,552.95	238,898.78	3,838,246.61
47017	Carroll	Tennessee	28,522	1,881	915.00	2,838.00	853,349.93	311,899.06	1,930,704.80	1,444,389.35	236,410.50	5,817,242.58	2,297,739.28	602,598.38	6,950,624.56
47019	Carter	Tennessee	57,424	4,135	2,393.50	5,887.50	1,933,838.67	893,099.43	3,531,657.57	3,278,831.37	720,739.66	10,903,193.61	5,212,670.04	1,865,557.53	13,206,757.02
47021	Cheatham	Tennessee	39,105	2,638	1,541.50	3,768.50	1,029,604.50	398,024.95	2,206,957.04	1,572,409.70	342,800.48	5,919,426.88	2,602,014.21	791,167.61	7,398,343.26
47023	Chester	Tennessee	17,131	1,172	646.50	1,703.00	500,138.96	150,993.96	1,234,125.71	857,554.45	120,628.41	4,320,713.67	1,357,693.41	318,670.63	4,993,233.79
47025	Claiborne	Tennessee	32,213	2,349	1,368.50	3,264.00	1,076,190.03	450,708.10	2,339,408.95	1,760,462.33	316,335.50	6,811,553.82	2,836,652.36	882,975.67	7,955,808.92
47027	Clay	Tennessee	7,861	560	322.00	804.00	283,811.36	73,270.67	816,199.54	477,163.94	55,755.50	2,644,483.36	760,975.31	141,522.46	2,976,870.72
47029	Cocke	Tennessee	35,662	2,665	1,572.00	3,697.00	1,248,818.15	503,144.41	2,483,572.03	2,164,971.35	459,120.57	7,761,771.24	3,413,789.51	1,101,168.66	9,212,004.27
47031	Coffee	Tennessee	52,796	3,746	2,115.50	5,248.50	1,629,362.35	676,129.79	3,114,873.73	2,914,951.14	745,959.83	8,977,255.32	4,544,313.50	1,547,621.59	11,164,096.53
47033	Crockett	Tennessee	14,586	997	517.00	1,493.50	445,422.32	138,452.25	1,047,157.65	804,281.53	124,715.08	3,749,917.47	1,249,703.85	285,750.88	4,380,209.21
47035	Cumberland	Tennessee	56,053	4,003	2,336.00	5,566.50	2,237,799.56	999,976.39	3,888,365.42	4,330,606.99	1,155,218.51	12,392,449.04	6,568,406.55	2,651,731.02	14,865,838.27

47037	Davidson	Tennessee	626,681	43,219	26,701.50	59,762.50	16,610,676.55	9,671,756.39	24,523,352.76	29,341,143.03	13,828,647.65	51,050,744.85	45,951,819.59	24,219,518.34	73,968,089.55
47039	Decatur	Tennessee	11,757	796	413.00	1,215.50	391,891.45	107,850.21	1,001,593.50	743,103.81	104,097.88	3,768,676.30	1,134,995.25	243,239.05	4,461,655.93
47041	De Kalb	Tennessee	18,723	1,304	731.00	1,873.00	586,550.50	198,423.94	1,365,309.53	926,861.29	157,258.80	3,604,551.15	1,513,411.79	389,323.54	4,407,876.73
47043	Dickson	Tennessee	49,666	3,359	1,769.00	4,921.50	1,387,343.96	558,816.06	2,868,578.99	2,309,633.70	466,085.64	7,419,955.73	3,696,977.66	1,114,594.35	9,180,051.80
47045	Dyer	Tennessee	38,335	2,543	1,244.50	3,813.50	1,063,866.60	379,052.20	2,203,245.91	1,787,070.75	332,394.68	6,853,832.29	2,850,937.35	781,822.22	8,357,730.27
47047	Fayette	Tennessee	38,413	2,690	1,458.00	3,883.00	1,265,638.29	506,187.82	2,646,544.25	2,246,212.77	465,012.87	8,011,707.39	3,511,851.06	1,126,104.69	9,903,846.20
47049	Fentress	Tennessee	17,959	1,305	784.00	1,854.00	596,050.30	217,380.33	1,370,048.76	1,034,629.04	160,933.47	4,962,855.14	1,630,679.35	426,927.36	5,678,962.20
47051	Franklin	Tennessee	41,052	2,876	1,694.00	4,115.50	1,318,000.97	551,550.95	2,663,581.71	2,265,091.24	505,699.60	6,951,445.70	3,583,092.21	1,260,030.79	8,533,553.74
47053	Gibson	Tennessee	49,683	3,321	1,582.50	4,897.50	1,452,310.13	584,180.35	2,699,075.70	2,475,602.36	512,739.01	8,159,661.71	3,927,912.49	1,288,421.40	10,024,365.35
47055	Giles	Tennessee	29,485	2,060	1,152.00	2,986.50	959,633.18	353,739.12	1,934,531.56	1,748,333.12	315,336.83	6,590,997.42	2,707,966.30	775,164.77	8,255,647.27
47057	Grainger	Tennessee	22,657	1,682	981.50	2,371.50	750,808.80	298,089.78	1,619,041.58	1,325,793.10	214,252.83	5,827,637.67	2,076,601.90	600,236.08	6,789,449.12
47059	Greene	Tennessee	68,831	5,119	2,883.50	7,211.50	2,438,447.20	1,147,328.66	4,336,338.43	4,525,634.32	1,067,417.28	12,783,224.77	6,964,081.51	2,476,220.55	15,346,375.60
47061	Grundy	Tennessee	13,703	960	544.00	1,377.50	436,773.24	126,980.98	1,040,873.17	689,970.17	102,193.69	3,349,047.84	1,126,743.40	258,547.36	3,894,100.09
47063	Hamblen	Tennessee	62,544	4,759	3,014.00	6,566.00	2,089,745.35	1,020,370.58	3,624,810.02	3,779,248.94	1,031,045.13	10,803,626.19	5,868,994.29	2,328,053.73	13,037,854.94
47065	Hamilton	Tennessee	336,463	23,287	14,004.50	33,518.50	10,174,390.48	5,470,236.93	15,765,940.41	18,767,720.51	7,781,584.66	38,326,561.73	28,942,110.99	13,567,856.76	51,626,521.13
47067	Hancock	Tennessee	6,819	503	302.50	704.00	236,434.70	52,501.80	698,315.28	405,639.95	42,747.35	2,288,636.40	642,074.64	107,071.92	2,726,476.30
47069	Hardeman	Tennessee	27,253	1,868	949.50	2,776.50	802,259.23	289,115.22	1,755,756.25	1,380,933.26	251,620.28	5,524,204.17	2,183,192.49	588,652.96	6,510,449.65
47071	Hardin	Tennessee	26,026	1,819	950.50	2,639.00	861,520.70	319,788.05	1,825,945.99	1,669,060.21	287,301.26	6,875,227.94	2,530,580.91	695,013.36	7,824,958.18
47073	Hawkins	Tennessee	56,833	4,225	2,502.50	5,992.00	1,927,936.35	845,099.61	3,565,607.92	3,329,702.16	857,639.50	9,252,474.80	5,257,638.52	1,958,794.78	11,974,959.96
47075	Haywood	Tennessee	18,787	1,293	699.50	1,906.50	543,227.16	162,111.75	1,341,617.36	937,780.36	172,647.04	3,685,628.89	1,481,007.52	373,489.66	4,705,630.12
47077	Henderson	Tennessee	27,769	1,902	1,025.00	2,769.00	826,203.64	298,449.90	1,862,132.74	1,346,462.04	230,373.88	5,372,238.51	2,172,665.67	603,883.94	6,591,585.59
47079	Henry	Tennessee	32,330	2,096	1,084.50	3,190.00	1,019,516.65	384,520.08	2,108,757.05	1,949,003.36	323,296.34	7,778,507.61	2,968,520.00	798,293.24	8,889,787.88
47081	Hickman	Tennessee	24,690	1,655	935.00	2,432.50	697,802.09	247,714.71	1,588,857.84	1,089,304.56	190,875.61	4,399,593.95	1,787,106.66	491,115.01	5,469,566.29
47083	Houston	Tennessee	8,426	566	302.00	837.50	268,732.71	64,823.48	806,157.40	418,893.57	49,615.58	2,170,206.39	687,626.29	118,782.20	2,518,976.60
47085	Humphreys	Tennessee	18,538	1,244	686.50	1,840.50	571,563.97	172,473.22	1,434,035.85	1,131,760.11	189,769.44	4,987,175.13	1,703,324.08	437,368.21	5,732,525.36
47087	Jackson	Tennessee	11,638	801	450.50	1,157.00	389,765.72	119,062.47	995,285.79	663,951.92	91,045.29	3,763,989.17	1,053,717.64	232,499.64	4,340,024.40
47089	Jefferson	Tennessee	51,407	3,841	2,290.50	5,266.50	1,711,456.23	804,768.61	3,175,430.06	2,981,660.45	760,338.45	8,751,113.81	4,693,116.67	1,782,313.29	10,920,690.23
47091	Johnson	Tennessee	18,244	1,293	717.00	1,851.00	595,537.52	205,363.32	1,265,114.67	1,238,244.69	166,636.08	4,814,396.12	1,833,782.21	428,221.29	5,443,564.55
47093	Knox	Tennessee	432,226	31,836	19,454.00	43,044.00	13,240,449.91	7,843,793.45	18,920,604.86	23,421,166.61	10,896,138.57	42,347,357.32	36,661,616.53	19,790,599.01	57,419,989.01
47095	Lake	Tennessee	7,832	474	241.50	732.00	207,322.99	38,959.23	677,645.31	324,706.58	34,731.05	1,996,707.93	532,029.57	82,484.15	2,365,258.49
47097	Lauderdale	Tennessee	27,815	1,875	968.00	2,718.50	736,515.26	238,045.91	1,615,854.21	1,176,749.21	179,052.68	5,109,660.77	1,913,264.47	482,822.13	6,014,392.83
47099	Lawrence	Tennessee	41,869	2,985	1,670.50	4,394.50	1,292,174.36	543,178.48	2,544,841.78	2,218,161.08	469,371.09	9,881,446.94	3,510,335.44	1,147,103.98	11,555,553.14
47101	Lewis	Tennessee	12,161	846	463.00	1,229.50	388,294.69	113,872.58	1,032,967.27	650,943.24	76,192.94	3,661,753.23	1,039,237.93	205,505.34	4,602,627.09
47103	Lincoln	Tennessee	33,361	2,360	1,378.00	3,366.00	1,069,976.53	432,715.19	2,042,236.54	1,831,396.51	369,163.02	6,252,197.29	2,901,373.04	899,404.85	7,851,134.07
47105	Loudon	Tennessee	48,556	3,549	2,144.50	4,917.50	1,835,370.65	806,588.71	3,359,216.17	3,370,643.70	890,124.97	9,970,175.10	5,206,014.35	1,919,941.58	12,273,290.31
47107	McMinn	Tennessee	52,266	3,718	2,125.00	5,345.00	1,693,464.52	711,884.23	3,114,471.99	3,076,344.47	781,147.93	9,565,861.49	4,769,808.99	1,669,472.65	12,109,818.45
47109	McNairy	Tennessee	26,075	1,829	931.00	2,656.00	832,590.24	277,158.51	1,757,797.53	1,362,545.91	243,125.89	5,546,655.54	2,195,136.16	602,007.06	6,445,865.49
47111	Macon	Tennessee	22,248	1,586	873.50	2,294.00	638,009.62	235,116.78	1,452,615.44	1,113,091.83	168,221.92	4,499,827.76	1,751,101.45	449,957.49	5,312,379.44
47113	Madison	Tennessee	98,294	6,686	3,534.00	9,838.50	2,709,551.07	1,127,089.91	4,621,601.99	4,842,364.28	1,193,953.19	13,587,561.77	7,551,915.36	2,652,827.69	16,673,762.16
47115	Marion	Tennessee	28,237	1,953	1,139.50	2,820.50	895,686.35	365,204.84	1,907,330.34	1,555,782.97	290,051.95	6,278,343.19	2,451,469.32	706,038.05	7,592,705.70
47117	Marshall	Tennessee	30,617	2,103	1,162.00	3,080.00	864,977.28	321,521.69	1,850,143.13	1,423,831.53	269,849.99	5,965,106.77	2,288,808.82	670,245.27	7,032,429.11
47119	Maury	Tennessee	80,956	5,667	3,087.00	8,131.50	2,390,505.53	1,129,285.53	4,208,769.81	4,014,761.85	1,041,137.23	12,118,966.18	6,405,267.38	2,331,337.44	15,455,050.70
47121	Meigs	Tennessee	11,753	829	479.50	1,170.50	387,915.43	110,071.60	1,062,429.02	667,667.71	99,064.08	3,105,676.51	1,055,583.14	238,303.66	3,677,799.75
47123	Monroe	Tennessee	44,519	3,235	2,040.00	4,492.50	1,483,473.30	677,296.80	2,882,625.40	2,491,681.72	556,472.86	9,654,522.69	3,975,155.02	1,387,789.97	11,175,405.23
47125	Montgomery	Tennessee	172,331	12,019	6,918.50	17,274.00	3,949,887.53	1,937,992.29	6,308,211.17	6,352,487.39	1,797,579.39	18,490,692.49	10,302,374.92	3,950,769.37	22,503,532.77
47127	Moore	Tennessee	6,362	440	247.50	648.00	211,920.50	47,305.41	669,901.56	397,920.97	56,290.98	2,194,266.50	609,841.47	110,776.89	2,495,860.77
47129	Morgan	Tennessee	21,987	1,566	927.00	2,223.00	682,015.00	252,050.98	1,510,090.99	1,163,097.81	199,827.29	5,241,297.41	1,845,112.81	490,017.16	6,207,149.62
47131	Obion	Tennessee	31,807	2,019	956.50	3,106.50	912,456.52	287,554.62	1,942,501.35	1,767,316.79	303,338.92	7,296,069.10	2,679,773.31	628,907.43	8,460,001.64
47133	Overton	Tennessee	22,083	1,576	946.50	2,266.00	723,305.67	264,370.56	1,652,248.77	1,206,008.82	197,928.81	5,543,368.39	1,929,314.48	520,352.35	6,276,992.89
47135	Perry	Tennessee	7,915	541	296.00	797.00	256,477.76	64,066.69	733,435.37	437,454.33	53,015.40	2,925,630.06	693,932.10	125,251.31	3,353,335.25
47137	Pickett	Tennessee	5,077	368	213.00	521.00	192,644.86	49,009.28	553,126.58	381,919.07	38,409.14	2,482,388.12	574,563.93	96,107.47	2,852,936.81
47139	Polk	Tennessee	16,825	1,161	654.00	1,670.00	518,192.22	168,825.53	1,224,065.73	883,502.13	132,159.96	4,668,616.79	1,401,694.35	329,033.83	5,201,659.86
47141	Putnam	Tennessee	72,321	5,025	2,894.00	7,228.50	2,127,679.26	962,721.31	3,848,946.93	3,653,222.84	889,121.19	10,765,661.75	5,780,902.10	2,102,736.59	12,897,900.27
47143	Rhea	Tennessee	31,809	2,264	1,302.50	3,209.00	979,831.10	410,929.56	2,009,992.29	1,732,553.49	371,240.39	6,331,551.77	2,712,384.59	868,855.12	7,511,463.49
47145	Roane	Tennessee	54,181	3,876	2,159.00	5,470.50	1,915,279.11	773,014.05	3,511,280.20	3,663,839.43	1,017,600.77	10,018,557.13	5,579,118.54	1,932,232.85	12,487,632.51
47147	Robertson	Tennessee	66,283	4,630	2,599.00	6,603.00	1,818,501.30	828,246.67	3,193,954.01	2,998,818.85	710,876.90	9,104,750.92	4,817,320.15	1,752,008.14	11,329,341.37
47149	Rutherford	Tennessee	262,604	18,536	10,679.00	25,926.00	6,435,412.49	3,457,397.33	10,255,120.84	10,720,213.56	3,989,088.49	26,175,558.77	17,155,626.04	8,024,806.11	33,582,508.05
47151	Scott	Tennessee	22,228	1,669	1,019.50	2,342.00	686,713.30	270,434.41	1,568,782.70	1,116,862.39	189,966.57	5,492,254.81	1,803,575.69	493,221.35	6,279,655.02
47153	Sequatchie	Tennessee	14,112	993	563.50	1,426.00	440,886.26	137,053.57	1,119,603.39	753,000.12	99,511.92	3,575,637.04	1,193,886.38	250,789.89	4,193,345.67
47155	Sevier	Tennessee	89,889	6,716	4,211.00	9,384.50	3,019,321.25	1,517,876.46	5,000,891.38	5,021,764.90	1,429,531.96	14,447,434.65	8,041,086.16	3,433,063.80	17,508,465.78
47157	Shelby	Tennessee	927,644	68,233	42,199.00	94,446.00	25,560,953.14	15,193,089.78	37,160,856.08	46,376,074.05	23,799,250.91	78,013,953.87	71,937,027.18	40,445,543.12	107,794,061.70
47159	Smith	Tennessee	19,166	1,357	799.00	1,933.00	565,066.20	191,168.49	1,330,926.90	899,142.52	160,502.90	3,738,916.23	1,464,208.72	389,596.10	4,471,039.81
47161	Stewart	Tennessee	13,324	870	443.00	1,274.00	406,173.26	112,760.92	1,044,968.35	679,707.42	111,795.13	2,739,867.69	1,085,880.68	251,929.71	3,515,071.43
47163	Sullivan	Tennessee	156,823	11,395	6,692.00	16,055.00	5,539,090.44	2,963,819.99	9,045,181.03	10,552,196.74	3,963,340.66	24,669,376.56	16,091,287.18	7,349,712.29	31,724,767.43
47165	Sumner	Tennessee	160,645	11,226	6,384.50	15,756.50	4,608,763.54	2,374,330.29	7,620,895.51	7,740,069.86	2,609,699.11	18,768,344.37	12,348,833.40	5,479,668.23	25,324,624.95
47167	Tipton	Tennessee	61,081	4,395	2,563.50	6,403.50	1,629,230.77	745,073.34	2,983,963.10	2,680,530.44	617,620.61	8,492,684.47	4,309,761.21	1,544,495.95	10,450,455.08

47169	Trousdale	Tennessee	7,870	543	305.50	787.50	233,874.76	57,054.97	656,170.25	387,744.01	49,179.89	2,276,837.41	621,618.76	119,279.05	2,707,376.57
47171	Unicoi	Tennessee	18,313	1,335	776.00	1,878.50	661,697.63	226,817.69	1,471,072.95	1,288,171.46	236,177.98	4,865,669.19	1,949,869.09	531,041.58	5,626,024.72
47173	Union	Tennessee	19,109	1,449	896.00	1,998.00	618,968.55	227,269.87	1,417,488.44	1,130,853.97	201,252.74	4,821,163.53	1,749,822.52	476,383.77	5,488,341.06
47175	Van Buren	Tennessee	5,548	384	225.00	564.00	180,418.21	36,743.44	591,366.16	329,402.41	55,241.13	1,976,583.47	509,820.61	100,223.09	2,190,171.84
47177	Warren	Tennessee	39,839	2,830	1,619.00	4,080.50	1,216,425.78	491,008.88	2,401,623.11	2,124,968.93	446,853.58	7,960,829.91	3,341,394.71	1,039,348.37	10,273,815.50
47179	Washington	Tennessee	122,979	8,941	5,184.50	12,732.00	3,984,975.48	1,994,868.72	6,700,274.42	6,804,192.64	2,352,729.11	17,538,531.15	10,789,168.11	4,785,305.36	22,687,382.15
47181	Wayne	Tennessee	17,021	1,158	631.00	1,674.00	532,365.81	172,128.98	1,355,883.80	852,556.33	118,106.57	4,010,731.40	1,384,922.14	321,165.25	5,056,786.34
47183	Weakley	Tennessee	35,021	2,211	1,159.50	3,343.00	965,656.53	307,951.88	1,987,905.71	1,727,715.12	292,953.92	6,541,236.84	2,693,371.65	656,277.06	7,722,752.28
47185	White	Tennessee	25,841	1,825	1,038.50	2,618.00	857,280.68	322,173.23	1,954,573.49	1,462,835.35	240,712.19	6,566,917.04	2,320,116.03	626,584.74	7,465,164.39
47187	Williamson	Tennessee	183,182	12,793	7,347.50	18,149.00	4,764,798.69	2,429,279.91	7,741,773.63	8,837,948.59	3,295,283.56	21,759,455.09	13,602,747.28	6,186,261.07	27,985,272.16
47189	Wilson	Tennessee	113,993	7,949	4,543.50	11,246.00	3,212,851.99	1,515,450.95	5,561,018.83	5,579,856.42	1,723,896.36	15,239,170.11	8,792,708.41	3,626,020.48	18,594,509.78
48001	Anderson	Texas	58,458	7,457	5,748.50	9,160.00	3,103,427.18	1,951,136.31	4,902,213.84	5,273,964.72	1,842,730.17	13,761,317.07	8,377,391.90	4,082,604.47	17,477,147.53
48003	Andrews	Texas	14,786	2,108	1,528.50	2,731.00	808,879.49	368,101.55	1,858,684.15	1,429,086.65	429,281.15	5,705,058.82	2,237,966.14	886,505.23	6,563,194.37
48005	Angelina	Texas	86,771	11,434	8,323.00	14,647.50	4,628,303.27	2,933,323.36	6,938,518.72	7,912,726.36	3,258,401.31	17,603,017.89	12,541,029.64	6,997,783.84	22,710,567.04
48007	Aransas	Texas	23,158	3,209	2,419.50	4,048.50	1,755,877.16	939,790.18	3,077,098.60	3,189,926.32	832,518.36	8,397,646.18	4,945,803.47	2,056,983.46	10,439,106.22
48009	Archer	Texas	9,054	1,177	908.00	1,477.50	521,842.46	216,010.26	1,198,424.84	947,457.32	188,536.97	4,881,311.65	1,469,299.78	469,206.15	5,576,994.29
48011	Armstrong	Texas	1,901	230	161.00	306.00	116,865.43	20,295.85	401,330.48	178,267.72	26,723.53	1,339,639.32	295,133.14	51,571.21	1,593,725.02
48013	Atascosa	Texas	44,911	6,608	4,964.50	8,196.50	2,597,626.41	1,575,139.26	4,150,767.09	4,327,986.45	1,509,068.11	12,221,983.90	6,925,612.86	3,466,104.11	15,666,205.89
48015	Austin	Texas	28,417	3,875	2,955.00	4,769.50	1,711,151.95	923,740.92	3,185,372.78	3,172,644.47	1,019,215.23	10,382,630.75	4,883,796.42	2,150,739.97	12,709,580.32
48017	Bailey	Texas	7,165	990	725.50	1,252.50	383,522.68	142,756.24	954,435.99	612,009.36	137,461.01	2,976,273.25	995,532.05	319,367.29	3,733,578.28
48019	Bandera	Texas	20,485	2,877	2,194.00	3,557.00	1,518,019.65	819,408.27	2,913,936.00	2,739,678.76	631,282.67	10,123,258.05	4,257,698.41	1,659,550.44	12,001,046.36
48021	Bastrop	Texas	74,171	10,761	8,534.50	12,847.50	4,333,766.31	2,805,642.91	6,692,149.83	7,103,048.57	2,727,777.37	17,842,997.67	11,436,814.88	5,957,318.12	22,688,942.60
48023	Baylor	Texas	3,726	487	351.50	626.00	257,504.68	73,950.21	827,204.31	553,144.48	72,070.13	3,243,766.78	810,649.16	162,744.84	3,577,848.00
48025	Bee	Texas	31,861	4,462	3,352.00	5,524.50	1,714,844.05	967,035.91	3,075,381.51	2,645,784.29	751,897.47	8,459,570.37	4,360,628.34	1,890,561.42	10,311,530.54
48027	Bell	Texas	310,235	44,972	35,288.00	55,443.00	15,247,667.33	11,149,094.59	19,925,656.81	26,761,259.77	15,524,476.33	48,690,472.39	42,008,927.10	28,001,202.22	66,467,257.27
48029	Bexar	Texas	1,714,773	248,160	198,022.00	298,373.00	89,998,928.15	70,557,843.35	111,434,787.30	157,061,855.30	111,525,274.90	217,268,421.80	247,060,783.50	184,199,858.70	322,286,011.30
48031	Blanco	Texas	10,497	1,518	1,198.50	1,828.00	740,028.01	334,475.85	1,601,520.17	1,310,550.46	300,344.18	4,501,918.42	2,050,578.47	729,504.73	5,479,956.77
48033	Borden	Texas	641	91	66.00	118.50	49,144.00	5,122.68	268,136.68	113,597.10	10,378.26	992,242.58	162,741.11	17,450.54	1,161,099.19
48035	Bosque	Texas	18,212	2,482	1,907.50	3,064.50	1,231,560.59	656,052.21	2,260,977.31	2,296,048.29	519,487.16	8,103,571.73	3,527,608.88	1,310,706.15	9,535,994.54
48037	Bowie	Texas	92,565	12,488	9,395.50	15,720.50	5,222,246.27	3,314,406.46	7,655,088.44	9,174,628.59	4,019,083.60	20,200,031.47	14,396,874.86	7,921,147.30	26,063,390.12
48039	Brazoria	Texas	313,166	42,204	31,888.50	52,338.00	15,341,325.31	10,743,996.94	20,949,643.61	26,344,956.99	14,797,156.14	49,181,277.00	41,686,282.31	26,799,513.42	66,861,168.96
48041	Brazos	Texas	194,851	24,803	18,855.50	30,536.00	7,990,690.30	5,468,548.84	11,001,610.61	12,612,328.21	5,617,188.72	29,523,765.60	20,603,018.51	12,063,952.64	38,537,196.00
48043	Brewster	Texas	9,232	1,218	821.00	1,632.50	571,196.78	214,990.36	1,317,361.36	1,035,192.16	184,789.02	4,425,045.09	1,606,388.94	456,823.82	5,514,600.37
48045	Briscoe	Texas	1,637	205	141.50	264.50	101,076.75	17,742.86	394,608.83	183,468.76	22,784.92	1,332,658.71	284,545.51	43,173.32	1,422,208.67
48047	Brooks	Texas	7,223	1,066	718.00	1,421.50	478,963.33	183,913.98	1,236,469.54	891,239.65	155,075.16	4,109,118.52	1,370,202.98	381,978.35	4,693,239.10
48049	Brown	Texas	38,106	5,448	4,181.50	6,603.50	2,481,780.82	1,490,517.02	4,070,224.97	4,515,848.09	1,415,958.63	12,809,398.47	6,997,628.91	3,319,089.77	15,371,790.42
48051	Burleson	Texas	17,187	2,351	1,768.50	2,901.50	1,097,698.90	540,445.98	2,169,905.68	2,023,018.42	510,028.21	7,859,187.92	3,120,717.32	1,181,812.96	8,744,550.33
48053	Burnet	Texas	42,750	6,207	4,947.00	7,468.00	2,979,459.61	1,818,421.85	4,834,689.32	5,524,565.98	1,915,820.35	15,823,327.47	8,504,025.59	4,244,933.11	19,418,809.02
48055	Caldwell	Texas	38,066	5,707	4,664.00	6,739.00	2,238,189.35	1,351,001.90	3,918,591.84	3,670,878.09	1,114,245.34	11,908,892.56	5,909,067.44	2,789,299.81	14,000,859.32
48057	Calhoun	Texas	21,381	3,049	2,302.50	3,827.50	1,293,157.06	678,068.45	2,510,210.87	2,600,346.24	818,042.95	9,022,459.65	3,893,503.30	1,633,309.60	10,460,571.84
48059	Callahan	Texas	13,544	1,894	1,453.50	2,319.50	884,996.37	405,943.71	1,756,392.24	1,975,050.05	358,642.61	8,354,443.06	2,860,046.42	856,510.69	9,433,828.73
48061	Cameron	Texas	406,220	59,893	41,065.00	80,533.50	21,180,758.79	14,149,238.02	30,198,905.19	33,064,317.89	17,833,045.84	57,041,468.78	54,245,076.68	33,887,234.11	81,971,344.97
48063	Camp	Texas	12,401	1,781	1,344.50	2,201.00	777,693.27	341,197.99	1,769,486.53	1,277,387.67	301,148.64	4,844,703.80	2,055,080.94	716,442.54	5,871,153.12
48065	Carson	Texas	6,182	690	457.50	919.00	330,641.78	93,656.00	998,073.54	690,764.22	195,420.09	3,194,790.26	1,021,406.01	316,389.95	3,560,335.95
48067	Cass	Texas	30,464	4,164	3,091.50	5,211.00	1,976,999.84	1,099,670.32	3,453,894.75	3,419,368.56	1,042,667.82	9,313,520.59	5,396,368.40	2,420,832.11	11,592,873.81
48069	Castro	Texas	8,062	1,090	828.00	1,358.00	419,434.63	166,849.24	1,033,644.83	731,485.78	146,013.02	3,364,782.30	1,150,920.40	342,687.75	3,902,801.72
48071	Chambers	Texas	35,096	4,589	3,439.00	5,736.50	1,665,629.13	941,163.63	2,957,552.80	3,038,467.88	1,079,252.29	10,111,685.41	4,704,097.01	2,197,749.44	11,919,282.04
48073	Cherokee	Texas	50,845	6,783	5,124.50	8,440.50	2,864,723.61	1,699,561.12	4,677,525.30	4,754,198.95	1,655,005.00	12,379,062.13	7,618,922.57	3,717,200.66	15,475,940.22
48075	Childress	Texas	7,041	868	622.00	1,111.50	352,875.63	127,873.72	906,113.46	612,440.16	104,958.52	2,946,913.21	965,315.79	259,170.49	3,400,062.04
48077	Clay	Texas	10,752	1,371	1,067.00	1,693.00	647,412.24	274,365.30	1,446,594.87	1,052,010.45	219,405.02	4,417,070.95	1,699,422.69	554,902.47	5,103,897.14
48079	Cochran	Texas	3,127	432	318.50	551.00	186,346.31	49,303.59	572,501.03	294,857.14	52,864.35	1,937,207.56	481,203.45	107,610.10	2,304,657.98
48081	Coke	Texas	3,320	483	365.00	604.00	264,145.19	76,508.54	769,899.58	570,389.82	72,699.21	3,089,521.24	834,535.01	168,522.77	3,603,289.98
48083	Coleman	Texas	8,895	1,275	979.00	1,557.00	643,628.67	278,111.27	1,458,404.07	1,076,259.94	197,071.71	4,688,686.51	1,719,888.61	570,789.10	5,724,791.32
48085	Collin	Texas	782,341	107,547	84,575.00	129,742.00	36,248,915.54	27,592,527.11	46,062,961.21	66,332,723.55	44,427,227.81	99,671,719.30	102,581,639.10	75,027,760.37	141,944,738.70
48087	Collingsworth	Texas	3,057	373	251.00	481.00	172,252.10	43,207.58	562,636.18	304,875.39	40,120.40	1,966,289.26	477,127.49	90,636.47	2,454,335.27
48089	Colorado	Texas	20,874	2,922	2,279.50	3,602.50	1,395,907.44	779,889.78	2,486,942.08	2,442,089.92	662,344.64	7,642,506.45	3,837,997.36	1,636,595.11	9,508,848.61
48091	Comal	Texas	108,472	15,572	12,325.50	18,622.00	7,035,292.33	5,030,993.69	9,885,866.32	12,510,535.02	5,928,441.89	27,773,232.41	19,545,827.36	11,830,276.57	34,919,929.93
48093	Comanche	Texas	13,974	1,991	1,524.00	2,430.50	974,718.63	459,295.04	1,959,831.91	1,720,713.78	375,210.32	6,116,984.00	2,695,432.41	986,080.87	7,099,989.99
48095	Concho	Texas	4,087	548	422.50	672.50	244,693.72	73,069.46	694,336.33	427,781.08	66,271.25	2,922,033.15	672,474.80	146,315.59	3,138,680.03
48097	Cooke	Texas	38,437	5,173	4,060.50	6,340.50	2,305,375.62	1,397,350.87	3,808,755.45	4,035,159.91	1,377,803.71	10,444,636.05	6,340,535.54	3,121,733.07	13,267,809.27
48099	Coryell	Texas	75,388	10,781	8,500.00	13,118.00	3,461,185.25	2,229,832.29	5,463,529.55	5,224,059.09	2,016,526.79	14,573,916.74	8,685,244.34	4,637,484.96	18,244,748.83
48101	Cottle	Texas	1,505	192	137.50	255.50	115,247.97	17,621.50	431,443.24	176,526.20	27,054.17	906,977.85	291,774.18	49,337.95	1,317,711.30
48103	Crane	Texas	4,375	623	441.50	813.00	224,685.82	66,231.43	639,308.87	450,603.96	117,424.75	2,428,148.43	675,289.78	201,118.70	2,697,481.50
48105	Crockett	Texas	3,719	547	389.50	709.00	228,178.96	69,734.79	700,207.32	358,167.88	66,239.10	1,900,822.49	586,346.84	150,510.65	2,329,316.15
48107	Crosby	Texas	6,059	886	692.50	1,088.00	372,461.07	148,708.95	882,503.16	622,857.21	147,743.33	2,968,267.25	995,318.28	317,905.51	3,361,926.44
48109	Culberson	Texas	2,398	316	210.00	420.00	124,940.56	27,285.66	401,513.69	236,869.74	32,654.46	1,523,701.41	361,810.30	65,153.18	1,819,908.25

48111	Dallam	Texas	6,703	681	431.50	929.50	238,442.37	65,529.81	678,721.33	453,769.12	80,152.05	2,147,888.82	692,211.49	159,157.76	2,375,668.18
48113	Dallas	Texas	2,368,139	322,882	262,355.00	387,298.00	111,782,060.00	89,709,274.57	135,560,363.80	211,513,801.90	159,713,991.20	280,542,501.00	323,295,861.90	254,473,453.70	408,635,229.40
48115	Dawson	Texas	13,833	1,984	1,522.00	2,460.00	779,960.81	362,040.74	1,538,952.20	1,214,141.22	286,360.85	4,558,131.91	1,994,102.04	730,057.24	5,609,120.73
48117	Deaf Smith	Texas	19,372	2,479	1,773.50	3,187.50	880,111.74	420,213.60	1,835,454.51	1,559,062.28	361,824.94	6,497,725.52	2,439,174.02	879,830.62	7,493,078.09
48119	Delta	Texas	5,231	727	551.50	912.00	353,261.87	123,252.26	858,673.92	588,633.38	91,926.42	3,029,169.77	941,895.25	246,508.21	3,490,820.10
48121	Denton	Texas	662,614	88,656	71,562.50	106,066.00	29,090,386.35	22,441,538.37	37,117,459.59	47,455,835.36	29,667,144.37	76,716,672.41	76,546,221.71	53,986,309.43	109,467,279.20
48123	De Witt	Texas	20,097	2,938	2,359.00	3,612.00	1,384,278.82	725,787.49	2,669,584.90	2,436,962.17	591,825.07	7,807,212.52	3,821,240.99	1,559,541.46	9,332,644.18
48125	Dickens	Texas	2,444	325	244.50	408.00	156,037.75	39,708.38	490,555.10	304,481.45	41,905.13	1,829,990.31	460,519.20	86,190.29	2,153,329.55
48127	Dimmit	Texas	9,996	1,490	1,054.50	1,911.50	595,832.54	249,323.21	1,354,944.38	993,998.27	212,551.82	4,068,327.46	1,589,830.81	503,962.95	4,764,070.47
48129	Donley	Texas	3,677	432	302.50	564.00	214,066.69	56,492.46	585,805.17	362,633.68	51,610.89	2,207,129.14	576,700.37	115,611.14	2,456,699.11
48131	Duval	Texas	11,782	1,691	1,190.50	2,176.50	707,411.22	302,992.35	1,426,699.78	1,302,723.58	275,017.54	5,204,032.64	2,010,134.79	620,358.36	5,979,043.93
48133	Eastland	Texas	18,583	2,580	1,971.00	3,171.50	1,250,965.17	653,229.01	2,428,825.83	2,276,358.34	528,734.36	7,775,974.54	3,527,323.51	1,368,360.91	9,165,418.19
48135	Ector	Texas	137,130	19,563	14,146.00	25,539.50	7,015,121.69	4,548,200.44	10,023,514.20	12,468,679.45	5,648,111.56	27,332,834.70	19,483,801.14	10,852,516.39	34,635,236.81
48137	Edwards	Texas	2,002	287	209.00	363.50	149,875.98	35,007.14	524,563.26	261,828.49	37,875.07	1,449,551.88	411,704.47	77,225.70	1,751,577.29
48139	Ellis	Texas	149,610	20,408	16,123.00	24,011.50	7,429,773.61	5,219,111.92	10,616,611.37	12,222,304.59	5,859,139.07	26,526,158.71	19,652,078.19	11,923,174.73	36,758,856.98
48141	El Paso	Texas	800,647	97,760	62,827.00	134,702.00	34,806,493.46	21,996,838.42	48,347,961.84	58,077,559.05	31,463,856.78	96,011,103.15	92,884,052.50	54,999,655.92	139,559,617.80
48143	Erath	Texas	37,890	5,116	3,928.00	6,256.00	2,027,118.57	1,107,852.79	3,389,142.90	3,320,828.08	955,069.95	10,108,219.89	5,347,946.65	2,368,534.33	12,435,630.29
48145	Falls	Texas	17,866	2,409	1,896.50	2,948.50	1,084,305.99	531,204.36	2,075,966.46	1,910,055.98	416,702.44	7,336,119.46	2,994,361.98	1,049,867.90	8,587,445.47
48147	Fannin	Texas	33,915	4,599	3,510.00	5,696.00	2,093,346.14	1,201,468.85	3,591,733.80	3,688,236.72	1,150,607.22	10,533,767.24	5,781,582.87	2,681,583.74	12,858,409.57
48149	Fayette	Texas	24,554	3,482	2,736.00	4,212.00	1,821,066.09	1,009,128.03	3,328,042.36	3,489,245.55	996,764.19	10,209,110.58	5,310,311.65	2,214,041.45	11,997,847.71
48151	Fisher	Texas	3,974	566	430.50	704.00	284,154.85	95,796.08	711,228.12	512,653.99	90,333.85	2,315,169.69	796,808.83	202,923.72	2,730,935.16
48153	Floyd	Texas	6,446	893	672.50	1,108.00	388,079.00	138,975.33	943,444.23	650,399.29	119,424.53	2,753,353.80	1,038,478.29	290,551.89	3,400,409.46
48155	Foard	Texas	1,336	167	115.50	219.00	87,781.98	13,875.66	311,058.41	155,986.72	17,228.05	1,143,450.15	243,768.70	32,855.24	1,206,938.40
48157	Fort Bend	Texas	585,375	78,957	61,418.50	96,485.50	26,999,674.77	19,557,092.09	34,825,864.92	46,759,046.21	29,242,816.62	80,022,538.13	73,758,720.97	51,987,392.95	110,935,357.60
48159	Franklin	Texas	10,605	1,497	1,124.00	1,856.00	679,872.36	302,523.58	1,458,041.25	1,282,816.59	253,915.06	4,954,441.61	1,962,688.95	612,972.01	5,900,942.28
48161	Freestone	Texas	19,816	2,597	2,002.50	3,155.00	1,179,073.50	601,090.34	2,286,253.83	2,114,153.13	600,446.88	6,467,430.87	3,293,226.63	1,350,170.45	7,801,521.70
48163	Frio	Texas	17,217	2,448	1,791.50	3,053.50	916,099.20	447,410.41	1,934,283.84	1,432,534.61	334,066.43	5,578,950.66	2,348,633.81	869,761.91	7,047,500.88
48165	Gaines	Texas	17,526	2,599	1,958.00	3,282.00	836,348.55	440,182.96	1,713,165.57	1,371,989.71	396,902.33	5,252,611.17	2,208,338.26	912,841.97	6,383,719.72
48167	Galveston	Texas	291,309	37,708	28,596.50	47,253.00	14,873,430.44	10,381,731.73	20,244,188.35	26,698,787.63	14,829,349.58	47,305,118.81	41,572,218.07	26,220,854.49	66,157,632.34
48169	Garza	Texas	6,461	895	683.00	1,106.00	346,159.31	127,482.15	831,609.00	555,514.22	126,407.30	3,316,703.35	901,673.53	269,647.77	3,647,949.34
48171	Gillespie	Texas	24,837	3,585	2,833.50	4,379.00	1,997,129.37	1,166,676.12	3,251,300.44	3,821,415.57	1,114,532.61	11,563,005.86	5,818,544.94	2,612,854.50	13,804,931.29
48173	Glasscock	Texas	1,226	178	125.00	232.00	72,379.04	11,756.47	297,143.21	105,427.21	19,755.67	775,297.59	177,806.25	33,799.34	959,334.78
48175	Goliad	Texas	7,210	1,018	790.50	1,271.00	493,236.96	197,965.50	1,106,760.01	927,015.37	153,730.77	4,208,192.63	1,420,252.33	390,199.18	4,894,578.79
48177	Gonzales	Texas	19,807	3,023	2,421.50	3,581.50	1,283,727.77	712,374.80	2,448,044.64	2,100,941.13	551,197.76	6,594,987.00	3,384,668.90	1,357,932.17	8,138,001.79
48179	Gray	Texas	22,535	2,546	1,760.50	3,367.00	1,079,801.96	490,885.65	2,034,023.50	2,072,749.81	558,421.41	7,257,067.49	3,152,551.77	1,151,400.12	8,700,624.82
48181	Grayson	Texas	120,877	16,355	12,953.00	20,054.50	7,096,285.51	4,967,413.85	10,226,521.19	12,917,625.98	5,888,813.88	28,314,040.61	20,013,911.50	11,362,905.76	36,392,208.73
48183	Gregg	Texas	121,730	17,044	12,784.00	21,105.00	6,913,218.33	4,697,649.08	10,148,542.93	12,249,993.27	5,690,887.67	25,265,927.12	19,163,211.61	10,953,162.22	33,932,784.61
48185	Grimes	Texas	26,604	3,444	2,627.50	4,243.50	1,479,754.80	797,033.60	2,606,278.91	2,565,224.77	755,563.01	8,135,156.29	4,044,979.57	1,757,012.57	9,904,821.64
48187	Guadalupe	Texas	131,533	19,566	16,083.50	23,285.00	7,478,097.23	5,379,496.78	10,539,686.92	12,715,441.53	6,209,137.03	25,949,634.97	20,193,538.76	12,348,357.37	34,366,415.07
48189	Hale	Texas	36,273	5,056	3,898.50	6,208.00	1,908,768.68	1,096,056.44	3,164,681.30	3,118,762.85	908,942.14	9,701,448.47	5,027,531.53	2,282,123.94	12,606,421.45
48191	Hall	Texas	3,353	425	303.00	550.00	196,245.59	56,190.20	536,976.17	329,928.01	47,755.15	1,860,902.64	526,173.60	114,189.59	2,188,942.01
48193	Hamilton	Texas	8,517	1,195	905.50	1,467.50	641,344.73	270,178.17	1,421,549.16	1,190,397.20	225,048.69	4,736,939.38	1,831,741.92	562,315.65	5,506,122.09
48195	Hansford	Texas	5,613	589	376.50	788.00	220,955.70	65,729.12	589,078.66	408,188.58	76,600.24	2,063,779.10	629,144.27	154,574.23	2,318,755.04
48197	Hardeman	Texas	4,139	535	384.00	690.50	240,698.73	81,674.23	708,712.59	410,469.18	70,179.48	2,148,172.66	651,167.91	167,036.11	2,471,001.68
48199	Hardin	Texas	54,635	7,115	5,201.50	9,038.00	2,981,771.10	1,766,935.99	4,938,561.75	4,860,936.57	1,696,893.81	12,206,796.69	7,842,707.67	3,717,879.57	15,558,749.84
48201	Harris	Texas	4,092,459	537,820	410,488.00	652,909.00	184,075,446.10	139,575,462.60	226,454,000.40	348,438,611.60	255,230,688.00	448,628,415.40	532,514,057.60	402,020,125.00	666,999,925.90
48203	Harrison	Texas	65,631	9,187	6,795.00	11,693.50	3,722,545.36	2,320,426.25	5,764,043.11	6,671,238.73	2,564,912.01	16,052,413.40	10,393,784.08	5,310,671.61	20,774,186.56
48205	Hartley	Texas	6,062	622	420.00	859.00	244,854.11	70,523.48	699,627.61	483,625.28	75,692.37	2,468,636.19	728,479.39	158,511.54	2,806,480.72
48207	Haskell	Texas	5,899	795	587.50	1,010.00	401,299.19	147,593.42	979,215.81	715,743.99	130,273.45	3,435,150.24	1,117,043.19	301,665.59	3,853,744.65
48209	Hays	Texas	157,107	22,881	18,544.50	27,341.00	7,991,809.21	5,770,331.12	11,213,120.23	12,426,941.06	5,869,999.44	25,930,140.82	20,418,750.27	12,706,962.25	34,497,102.71
48211	Hemphill	Texas	3,807	428	286.50	576.00	165,234.21	39,791.55	476,496.29	331,352.58	66,714.80	2,092,392.90	496,586.79	115,255.90	2,426,797.70
48213	Henderson	Texas	78,532	10,460	7,953.00	12,860.00	5,005,970.29	3,215,538.60	7,764,628.21	9,069,717.37	3,551,471.73	21,015,430.79	14,075,687.66	7,453,418.20	27,975,051.14
48215	Hidalgo	Texas	774,769	114,247	74,354.50	153,452.00	37,098,974.60	24,467,084.84	50,405,185.72	57,277,523.48	32,187,786.15	92,613,772.25	94,376,498.08	58,141,699.51	138,857,356.10
48217	Hill	Texas	35,089	4,766	3,736.50	5,780.50	2,212,553.83	1,285,194.51	3,667,075.00	3,901,992.03	1,169,954.44	11,815,940.85	6,114,545.87	2,716,357.38	13,836,751.63
48219	Hockley	Texas	22,935	3,219	2,443.00	3,975.00	1,271,195.89	668,717.65	2,442,088.63	2,174,319.68	584,253.91	7,357,348.64	3,445,515.57	1,347,788.07	8,921,355.53
48221	Hood	Texas	51,182	6,875	5,266.00	8,444.00	3,499,276.75	2,190,365.55	5,449,484.17	6,848,635.05	2,361,114.24	20,132,763.16	10,347,911.80	4,993,841.57	23,506,568.49
48223	Hopkins	Texas	35,161	4,927	3,793.00	6,072.00	2,113,649.57	1,251,668.00	3,537,656.30	3,681,950.71	1,197,274.55	11,163,529.96	5,795,600.28	2,792,988.56	13,405,308.05
48225	Houston	Texas	23,732	3,088	2,340.00	3,849.00	1,507,755.06	831,771.12	2,747,468.39	2,823,701.49	794,977.89	9,064,814.73	4,331,456.55	1,829,170.01	11,587,405.41
48227	Howard	Texas	35,012	4,933	3,680.50	6,135.00	2,049,315.91	1,141,442.80	3,444,430.24	3,491,289.90	1,146,844.07	10,961,590.39	5,540,605.81	2,532,541.87	13,012,908.37
48229	Hudspeth	Texas	3,476	442	283.50	585.50	181,336.90	43,131.62	639,838.84	278,392.58	64,871.00	1,487,524.23	459,729.49	112,650.02	1,897,482.12
48231	Hunt	Texas	86,129	11,769	8,989.50	14,398.50	4,922,398.38	3,225,878.98	7,210,005.09	8,955,805.19	3,820,579.49	20,746,277.90	13,878,203.57	7,559,350.28	26,207,009.51
48233	Hutchinson	Texas	22,150	2,378	1,560.50	3,173.00	996,833.05	435,322.24	2,169,670.76	1,958,162.17	534,202.78	6,435,985.86	2,954,995.21	1,055,894.93	7,471,066.15
48235	Irion	Texas	1,599	223	164.00	284.00	106,083.94	19,704.30	387,492.70	194,355.84	37,429.59	1,290,738.90	300,439.79	62,501.62	1,566,857.59
48237	Jack	Texas	9,044	1,162	888.50	1,452.00	488,020.31	201,935.97	1,043,760.70	880,557.32	219,753.85	3,716,344.01	1,368,577.63	475,803.16	4,280,752.12
48239	Jackson	Texas	14,075	2,012	1,544.00	2,480.50	886,209.82	425,189.86	1,707,861.02	1,576,665.28	385,267.77	6,350,483.36	2,462,875.10	902,572.50	7,336,581.05
48241	Jasper	Texas	35,710	4,634	3,375.50	5,796.00	2,059,840.14	1,191,028.26	3,500,969.86	3,906,849.69	1,123,662.04	14,373,537.66	5,966,689.83	2,556,377.57	16,190,619.74

48243	Jeff Davis	Texas	2,342	295	194.00	398.00	177,465.65	34,118.90	564,110.33	308,444.24	42,423.84	1,772,665.81	485,909.90	82,983.06	2,055,809.66
48245	Jefferson	Texas	252,273	32,509	23,899.50	41,911.00	13,126,153.91	9,134,561.10	18,349,172.61	24,376,024.70	13,229,841.80	44,757,991.05	37,502,178.61	23,107,301.53	58,377,452.06
48247	Jim Hogg	Texas	5,300	783	528.50	1,049.50	326,275.12	96,080.80	844,590.27	514,606.51	100,186.30	2,680,473.98	840,881.62	221,796.68	3,147,776.21
48249	Jim Wells	Texas	40,838	5,994	4,289.00	7,600.00	2,369,559.97	1,384,473.52	3,961,020.59	4,036,742.65	1,318,764.33	11,798,017.54	6,406,302.62	3,009,407.25	14,426,036.52
48251	Johnson	Texas	150,934	20,479	15,869.50	25,146.50	7,909,874.56	5,475,635.99	11,299,403.29	13,627,866.66	6,231,060.43	31,322,009.42	21,537,741.22	12,333,103.70	41,323,803.21
48253	Jones	Texas	20,202	2,681	2,035.50	3,356.50	1,130,700.76	580,582.14	2,141,921.64	1,912,948.86	441,246.49	6,898,802.51	3,043,649.62	1,181,730.79	8,202,713.67
48255	Karnes	Texas	14,824	2,076	1,660.50	2,541.50	865,690.79	416,112.46	1,695,896.07	1,557,503.72	321,757.88	6,914,462.08	2,423,194.50	862,907.04	7,969,970.87
48257	Kaufman	Texas	103,350	14,344	11,397.00	17,323.50	5,326,286.25	3,656,294.96	7,853,083.71	8,442,187.74	3,717,864.80	19,926,779.50	13,768,473.99	7,976,273.21	25,854,703.38
48259	Kendall	Texas	33,410	4,767	3,738.00	5,823.50	2,212,109.59	1,347,625.13	3,679,895.58	3,992,218.47	1,384,377.75	12,153,092.71	6,204,328.06	3,070,290.71	14,934,469.35
48261	Kenedy	Texas	416	59	38.00	82.00	26,714.13	1,868.79	149,186.71	39,849.52	6,943.54	278,914.57	66,563.66	9,285.30	420,531.45
48263	Kent	Texas	808	113	81.50	150.00	59,613.13	8,110.70	282,965.33	137,521.28	12,201.52	1,005,786.11	197,134.41	22,550.02	1,144,556.74
48265	Kerr	Texas	49,625	7,162	5,577.00	8,761.50	3,856,822.51	2,442,102.57	5,699,514.23	7,589,827.85	2,888,298.07	20,610,771.01	11,446,650.35	5,815,782.10	24,252,308.25
48267	Kimble	Texas	4,607	663	509.50	819.50	352,853.33	116,007.29	908,711.63	589,587.47	94,561.57	2,944,731.68	942,440.80	240,203.59	3,318,661.27
48269	King	Texas	286	37	23.00	52.00	16,010.46	819.92	94,720.35	26,554.25	4,033.70	83,794.42	42,564.71	5,524.45	198,384.20
48271	Kinney	Texas	3,598	509	352.50	655.00	254,449.61	73,940.42	684,483.21	517,051.34	86,980.89	2,606,841.06	771,500.94	188,441.01	2,981,923.04
48273	Kleberg	Texas	32,061	4,593	3,433.00	5,956.00	1,701,717.99	907,575.97	2,882,783.73	2,824,791.81	829,954.43	8,481,318.06	4,526,509.79	1,977,194.01	10,466,964.22
48275	Knox	Texas	3,719	504	361.00	644.00	235,640.07	68,059.01	660,682.96	450,771.04	81,135.60	2,537,845.77	686,411.11	171,828.85	2,916,793.43
48277	Lamar	Texas	49,793	6,905	5,338.00	8,589.50	3,091,833.57	1,880,860.51	5,009,781.62	5,627,429.16	2,064,458.75	15,001,847.26	8,719,262.73	4,472,830.60	18,686,849.92
48279	Lamb	Texas	13,977	1,946	1,466.50	2,442.00	817,162.45	369,447.04	1,755,333.37	1,339,091.91	318,257.19	5,150,598.30	2,156,254.36	784,684.02	6,344,306.09
48281	Lampasas	Texas	19,677	2,823	2,192.00	3,417.00	1,225,489.49	663,780.61	2,402,773.91	2,091,062.09	506,420.33	6,792,880.77	3,316,551.58	1,311,064.87	8,344,381.27
48283	La Salle	Texas	6,886	963	673.00	1,249.50	382,963.04	138,330.84	1,096,838.78	647,905.89	134,511.63	2,999,315.79	1,030,868.93	294,210.08	3,335,490.45
48285	Lavaca	Texas	19,263	2,795	2,183.50	3,400.50	1,409,502.56	767,576.72	2,585,006.36	2,370,408.81	622,565.89	7,267,818.14	3,779,911.37	1,590,543.05	8,665,829.66
48287	Lee	Texas	16,612	2,368	1,856.50	2,889.50	1,032,700.48	538,024.82	2,076,403.38	1,847,000.32	479,903.78	6,266,110.56	2,879,700.79	1,148,545.39	7,382,069.11
48289	Leon	Texas	16,801	2,219	1,733.00	2,730.00	1,128,333.03	607,345.77	2,079,272.46	2,322,255.27	614,575.64	8,116,413.44	3,450,588.30	1,375,151.08	9,139,665.23
48291	Liberty	Texas	75,643	9,731	7,491.00	12,268.00	3,762,115.05	2,335,935.01	5,835,767.67	6,099,802.60	2,374,003.72	13,916,713.84	9,861,917.65	5,349,085.79	18,462,394.09
48293	Limestone	Texas	23,384	3,133	2,409.50	3,873.50	1,385,835.22	727,467.92	2,584,208.24	2,398,288.97	618,389.42	7,978,070.06	3,784,124.19	1,524,266.66	9,686,176.28
48295	Lipscomb	Texas	3,302	345	224.50	474.00	138,591.48	30,351.01	471,343.39	224,897.23	39,128.41	1,573,801.20	363,488.71	76,852.86	1,808,487.46
48297	Live Oak	Texas	11,531	1,596	1,194.00	1,994.00	779,497.90	353,297.73	1,623,859.46	1,350,805.43	300,629.22	5,153,869.92	2,130,303.34	764,097.90	6,474,336.28
48299	Llano	Texas	19,301	2,756	2,126.00	3,334.50	1,752,025.85	1,004,774.72	3,186,316.48	3,504,798.46	922,265.02	11,149,402.03	5,256,824.30	2,197,928.66	12,787,264.05
48301	Loving	Texas	82	10	4.00	16.50	4,455.53	29.45	36,672.82	7,855.63	376.85	7,906.29	12,311.16	468.92	80,125.04
48303	Lubbock	Texas	278,831	39,918	31,870.50	47,720.50	14,795,291.32	11,014,961.71	19,205,207.32	25,331,231.32	13,702,202.92	50,267,905.27	40,126,522.63	26,514,635.07	66,612,465.31
48305	Lynn	Texas	5,915	861	664.00	1,059.00	365,600.36	132,501.76	940,389.73	603,438.57	126,321.40	2,842,244.14	969,038.93	282,425.98	3,244,202.43
48307	McCulloch	Texas	8,283	1,211	912.50	1,501.00	587,987.98	237,528.09	1,302,620.61	1,039,250.45	192,022.16	4,992,559.78	1,627,238.43	476,158.88	5,799,411.29
48309	McLennan	Texas	234,906	32,219	25,005.00	39,506.00	12,495,392.93	8,808,570.33	16,829,148.60	21,882,516.43	11,428,474.44	42,344,054.24	34,377,909.37	21,537,867.47	56,214,780.41
48311	McMullen	Texas	707	98	66.00	129.00	51,080.25	6,654.17	215,221.64	101,854.47	13,136.44	738,857.00	152,934.72	21,166.55	853,076.63
48313	Madison	Texas	13,664	1,761	1,367.50	2,174.50	710,461.13	337,246.11	1,476,858.35	1,491,237.19	268,598.44	5,995,623.58	2,201,698.32	675,086.56	6,945,065.79
48315	Marion	Texas	10,546	1,422	1,062.00	1,794.50	761,707.51	331,391.60	1,631,717.92	1,341,700.42	243,341.92	5,628,350.11	2,103,407.93	684,895.77	6,602,588.20
48317	Martin	Texas	4,799	711	544.50	894.50	282,845.00	91,216.56	831,441.54	486,534.36	112,153.56	2,837,632.68	769,379.37	214,300.69	3,256,349.54
48319	Mason	Texas	4,012	579	454.50	711.50	316,437.53	99,392.19	803,521.71	581,060.71	89,966.26	3,019,733.59	897,498.24	205,423.42	3,517,721.58
48321	Matagorda	Texas	36,702	5,119	3,852.00	6,354.00	2,147,107.05	1,216,951.83	3,581,114.89	4,199,676.44	1,401,970.44	13,395,167.73	6,346,783.49	2,782,828.90	15,489,496.21
48323	Maverick	Texas	54,258	8,156	5,860.50	10,499.00	2,855,510.36	1,677,451.30	4,869,424.87	4,214,430.50	1,431,271.17	12,148,048.59	7,069,940.85	3,505,812.69	14,973,626.36
48325	Medina	Texas	46,006	6,657	4,950.00	8,262.00	2,699,442.38	1,592,838.38	4,272,494.85	4,531,198.70	1,361,003.49	13,493,709.30	7,230,641.08	3,364,703.24	16,399,326.39
48327	Menard	Texas	2,242	326	245.50	412.50	190,071.19	47,779.03	606,804.27	366,591.41	40,739.41	2,099,127.27	556,662.60	93,322.46	2,429,992.62
48329	Midland	Texas	136,872	19,763	14,161.00	25,206.00	7,490,420.92	4,887,673.16	10,814,696.09	13,547,529.81	6,459,769.43	26,443,517.98	21,037,950.73	12,094,966.22	34,401,245.43
48331	Milam	Texas	24,757	3,494	2,736.00	4,342.00	1,568,979.52	836,233.48	3,029,217.06	3,001,200.41	884,629.91	10,677,669.17	4,570,179.93	1,939,776.82	12,476,765.46
48333	Mills	Texas	4,936	726	558.00	892.00	365,304.59	132,008.54	907,042.25	637,944.25	102,037.38	2,668,419.83	1,003,248.84	260,074.06	3,211,411.88
48335	Mitchell	Texas	9,403	1,307	1,005.50	1,607.50	528,677.19	214,979.41	1,158,071.41	904,966.86	191,174.17	3,794,271.29	1,433,644.05	462,881.50	4,411,061.51
48337	Montague	Texas	19,719	2,591	1,988.00	3,161.50	1,242,005.84	652,480.73	2,381,525.26	2,219,578.70	551,276.21	8,288,518.22	3,461,584.55	1,435,437.87	9,353,636.08
48339	Montgomery	Texas	455,746	59,566	45,321.00	72,781.00	22,390,351.17	15,755,220.32	29,749,730.56	38,833,777.91	23,015,680.96	64,764,708.82	61,224,129.08	40,461,157.64	91,104,329.29
48341	Moore	Texas	21,904	2,432	1,572.50	3,278.00	852,425.55	379,554.26	1,711,831.89	1,366,541.65	324,633.03	5,469,452.21	2,218,967.19	777,912.83	6,458,461.97
48343	Morris	Texas	12,934	1,784	1,335.50	2,218.00	863,621.89	432,622.23	1,769,108.91	1,538,857.75	412,224.38	5,835,496.69	2,402,479.63	952,726.13	6,746,574.25
48345	Motley	Texas	1,210	158	105.50	207.50	78,996.85	14,536.45	295,698.47	141,375.99	18,813.90	1,075,191.62	220,372.85	36,815.15	1,198,938.51
48347	Nacogdoches	Texas	64,524	8,606	6,340.50	10,840.50	3,348,340.57	1,977,032.25	5,190,966.68	5,459,834.95	1,900,779.62	15,386,260.95	8,808,175.52	4,521,945.73	19,403,629.02
48349	Navarro	Texas	47,735	6,466	5,011.50	7,984.50	2,671,076.84	1,663,325.85	4,350,591.58	4,437,269.92	1,417,403.61	12,496,262.41	7,108,346.76	3,600,463.42	15,555,327.77
48351	Newton	Texas	14,445	1,864	1,326.00	2,375.00	829,992.95	380,846.87	1,628,706.61	1,381,558.35	266,645.28	5,906,429.82	2,211,551.30	740,147.90	6,684,434.06
48353	Nolan	Texas	15,216	2,236	1,715.50	2,778.50	980,539.64	460,643.41	1,994,100.64	1,730,815.88	413,964.79	6,353,914.90	2,711,355.52	1,024,630.03	7,484,498.14
48355	Nueces	Texas	340,223	48,900	35,771.50	62,006.50	19,343,345.10	13,222,741.91	26,717,554.91	34,454,392.76	18,961,702.23	63,040,511.68	53,797,737.86	32,697,950.57	85,084,360.60
48357	Ochiltree	Texas	10,223	1,084	673.50	1,484.50	397,116.10	128,942.81	994,610.19	609,631.72	145,566.59	3,394,167.85	1,006,747.82	303,551.30	4,088,082.44
48359	Oldham	Texas	2,052	234	156.50	316.00	88,085.54	16,298.27	338,888.25	182,778.72	23,744.53	1,429,701.19	270,864.25	44,477.91	1,583,155.07
48361	Orange	Texas	81,837	10,691	7,861.50	13,674.00	4,524,409.62	2,840,745.87	6,972,835.57	8,364,300.68	3,450,042.18	20,217,472.50	12,888,710.30	6,895,268.98	25,547,439.98
48363	Palo Pinto	Texas	28,111	3,814	2,913.00	4,769.00	1,702,561.91	953,189.44	3,215,852.96	2,909,592.85	838,931.22	8,403,547.17	4,612,154.76	2,004,636.35	10,291,607.22
48365	Panola	Texas	23,796	3,262	2,417.50	4,066.50	1,410,949.81	735,076.43	2,496,630.34	2,516,309.28	745,943.11	8,229,386.73	3,927,259.09	1,661,250.70	9,772,809.09
48367	Parker	Texas	116,927	15,423	11,898.00	18,689.00	6,280,908.09	4,199,079.44	9,290,697.61	11,076,395.86	4,620,112.83	24,520,910.55	17,357,303.94	9,559,767.77	31,571,552.07
48369	Parmer	Texas	10,269	1,370	994.50	1,728.50	504,132.92	206,933.96	1,225,654.73	740,273.10	180,845.61	3,299,162.79	1,244,406.02	412,682.56	4,062,415.00
48371	Pecos	Texas	15,507	2,140	1,469.50	2,829.00	842,986.89	351,633.35	1,947,584.95	1,461,160.05	364,503.23	5,779,456.40	2,304,146.94	810,087.76	6,728,903.00
48373	Polk	Texas	45,413	5,818	4,244.00	7,224.50	2,803,412.26	1,648,036.08	4,769,397.17	5,134,027.47	1,747,920.63	15,077,883.81	7,937,439.74	3,859,742.65	18,040,899.43

48375	Potter	Texas	121,073	13,939	9,283.00	18,303.00	5,132,887.93	3,041,100.43	7,831,545.46	8,781,821.40	3,630,625.23	22,051,283.28	13,914,709.34	7,008,818.15	27,568,737.35
48377	Presidio	Texas	7,818	1,043	684.50	1,437.50	448,161.43	167,599.80	1,072,136.18	823,016.02	199,423.98	3,541,726.68	1,271,177.44	394,544.97	4,168,693.72
48379	Rains	Texas	10,914	1,489	1,139.50	1,841.50	761,155.35	341,258.42	1,657,638.62	1,332,119.44	239,882.05	5,637,370.73	2,093,274.79	682,664.87	6,649,283.23
48381	Randall	Texas	120,725	14,438	10,227.50	18,401.50	5,755,281.79	3,473,059.31	8,805,343.10	9,548,993.86	3,806,113.57	20,790,612.20	15,304,275.65	8,075,476.72	27,829,924.91
48383	Reagan	Texas	3,367	497	362.00	626.00	176,397.17	51,873.68	539,686.36	285,372.29	80,483.74	1,498,991.21	461,769.46	137,446.94	1,757,089.24
48385	Real	Texas	3,309	475	354.50	600.00	267,532.00	78,513.65	668,425.53	515,586.75	60,591.95	2,829,269.62	783,118.75	156,486.55	3,108,207.37
48387	Red River	Texas	12,860	1,782	1,344.50	2,230.50	900,011.64	432,151.24	1,764,538.48	1,545,373.76	308,844.26	6,089,901.41	2,445,385.40	833,804.98	7,038,143.48
48389	Reeves	Texas	13,783	1,786	1,214.50	2,378.00	666,638.45	287,361.25	1,439,152.99	1,129,384.22	235,025.28	4,468,863.86	1,796,022.67	574,987.55	5,213,116.25
48391	Refugio	Texas	7,383	1,050	801.50	1,301.00	493,228.39	201,106.96	1,116,805.62	814,124.46	176,306.45	3,706,826.04	1,307,352.85	419,266.64	4,282,322.68
48393	Roberts	Texas	929	100	65.00	140.00	43,342.43	4,972.03	215,003.62	68,802.60	8,383.80	482,552.86	112,145.03	14,658.98	595,028.27
48395	Robertson	Texas	16,622	2,280	1,789.50	2,784.50	1,024,612.46	509,469.98	1,957,235.16	1,806,589.99	471,751.65	7,017,787.66	2,831,202.46	1,079,859.99	8,120,352.72
48397	Rockwall	Texas	78,337	10,864	8,615.50	13,048.00	3,851,150.97	2,567,340.10	5,848,422.11	6,383,711.26	2,370,316.13	17,673,218.70	10,234,862.23	5,469,754.77	22,312,611.16
48399	Runnels	Texas	10,501	1,554	1,206.50	1,903.50	737,148.29	341,493.26	1,619,945.93	1,289,009.74	269,929.51	4,918,363.82	2,026,158.03	684,766.05	5,959,976.43
48401	Rusk	Texas	53,330	7,207	5,487.00	8,978.50	3,098,088.62	1,842,390.99	5,029,042.91	5,571,375.75	2,074,853.31	15,140,028.75	8,669,464.37	4,351,256.87	18,279,944.87
48403	Sabine	Texas	10,834	1,468	1,061.50	1,852.50	817,162.39	368,205.10	1,754,583.37	1,841,031.48	411,431.53	6,499,781.05	2,658,193.87	905,140.30	7,384,793.31
48405	San Augustine	Texas	8,865	1,189	863.00	1,514.00	629,398.69	255,369.86	1,398,977.33	1,186,090.67	194,640.58	5,190,496.92	1,815,489.36	509,449.10	5,716,433.94
48407	San Jacinto	Texas	26,384	3,404	2,561.00	4,228.00	1,600,293.28	850,334.42	2,765,223.24	2,733,584.48	733,365.06	8,528,542.12	4,333,877.76	1,772,344.36	10,461,961.40
48409	San Patricio	Texas	64,804	9,428	7,128.50	11,723.50	3,748,700.65	2,355,250.20	5,735,086.82	6,830,192.29	2,659,927.89	18,424,354.82	10,578,892.94	5,632,962.09	21,938,497.99
48411	San Saba	Texas	6,131	882	676.50	1,076.50	422,301.11	148,239.10	976,583.91	766,530.42	123,598.63	3,609,264.58	1,188,831.53	300,476.17	4,351,851.62
48413	Schleicher	Texas	3,461	534	394.00	674.50	209,744.41	57,862.42	710,074.44	357,398.17	64,567.56	1,901,916.17	567,142.59	132,381.41	2,504,396.10
48415	Scurry	Texas	16,921	2,451	1,865.00	3,044.50	1,016,230.52	479,008.26	1,955,587.33	1,915,731.48	506,797.42	6,862,522.03	2,931,962.00	1,090,598.64	8,402,401.78
48417	Shackelford	Texas	3,378	470	348.50	601.50	219,588.52	59,305.31	699,173.61	380,902.38	66,851.34	2,223,155.03	600,490.90	137,982.60	2,782,622.73
48419	Shelby	Texas	25,448	3,536	2,596.50	4,479.00	1,505,685.67	784,058.67	2,806,051.59	2,578,676.11	652,983.10	8,190,407.00	4,084,361.78	1,653,152.58	10,414,745.14
48421	Sherman	Texas	3,034	306	193.00	415.00	119,320.70	23,907.57	412,199.90	188,684.49	30,184.10	1,284,425.25	308,005.19	58,410.38	1,674,226.27
48423	Smith	Texas	209,714	29,851	22,978.50	37,011.50	12,288,277.66	8,685,664.80	16,887,964.53	22,167,207.98	11,706,533.76	40,878,622.26	34,455,485.64	21,826,569.89	55,729,188.76
48425	Somervell	Texas	8,490	1,153	867.00	1,429.00	494,064.34	201,903.32	1,210,181.92	1,030,863.77	305,286.84	4,506,137.26	1,524,928.10	557,356.43	5,124,101.40
48427	Starr	Texas	60,968	9,021	5,988.50	11,973.50	3,040,555.49	1,613,472.61	4,835,283.07	4,536,390.72	1,387,747.37	12,551,550.13	7,576,946.20	3,312,759.56	15,844,041.56
48429	Stephens	Texas	9,630	1,325	1,005.00	1,653.00	607,925.89	249,845.41	1,409,860.60	1,010,311.38	234,561.31	4,004,708.25	1,618,237.27	549,245.81	5,110,715.31
48431	Sterling	Texas	1,143	168	123.00	216.00	77,033.33	12,534.99	333,525.35	144,736.36	25,240.33	832,807.30	221,769.69	41,857.79	942,142.52
48433	Stonewall	Texas	1,490	204	150.00	262.00	99,354.00	18,026.55	345,297.89	201,382.22	26,347.88	1,418,646.03	300,736.21	48,662.69	1,631,666.89
48435	Sutton	Texas	4,128	609	445.00	773.50	245,449.31	75,487.21	696,661.31	507,797.59	136,824.17	2,434,400.64	753,246.89	238,538.73	2,776,912.57
48437	Swisher	Texas	7,854	1,028	766.00	1,295.00	449,367.20	171,006.53	1,116,669.72	785,720.44	143,000.12	3,542,291.28	1,235,087.64	344,094.29	4,312,710.02
48439	Tarrant	Texas	1,809,034	244,964	195,718.00	297,870.00	85,909,253.13	66,488,237.28	105,023,201.80	150,975,030.50	107,137,352.40	211,810,949.80	236,884,283.60	179,120,847.00	305,138,293.60
48441	Taylor	Texas	131,506	18,983	14,778.00	23,260.50	7,571,318.50	5,242,861.07	10,741,417.88	13,287,297.48	6,036,149.18	29,427,936.00	20,858,615.97	12,089,012.84	37,680,949.28
48443	Terrell	Texas	984	137	89.00	185.50	66,909.01	9,962.62	290,625.13	138,988.44	18,573.53	1,132,711.49	205,897.45	32,141.51	1,292,778.64
48445	Terry	Texas	12,651	1,793	1,353.00	2,256.50	774,781.05	319,313.09	1,856,274.05	1,219,265.92	310,607.43	4,306,556.16	1,994,046.98	701,346.62	5,655,079.47
48447	Throckmorton	Texas	1,641	220	157.50	284.50	114,858.41	21,289.21	412,592.34	201,293.12	27,681.33	1,385,379.85	316,151.53	52,046.42	1,707,354.83
48449	Titus	Texas	32,334	4,719	3,633.00	5,878.00	1,731,808.72	992,914.05	2,811,006.73	2,853,021.65	881,084.81	9,692,991.62	4,584,830.37	2,130,838.12	11,392,888.73
48451	Tom Green	Texas	110,224	16,003	12,304.00	19,856.50	6,553,204.73	4,437,078.75	9,734,128.98	11,205,732.41	4,945,286.28	23,735,212.83	17,758,937.14	10,100,168.93	30,753,539.91
48453	Travis	Texas	1,024,266	147,061	119,715.00	175,561.00	49,540,037.86	38,998,955.37	61,584,065.13	87,474,392.84	60,290,862.04	124,943,306.50	137,014,430.70	102,541,345.70	178,599,609.20
48455	Trinity	Texas	14,585	1,875	1,384.00	2,360.50	980,327.34	454,791.18	1,834,105.79	1,713,730.71	332,939.79	6,481,789.22	2,694,058.05	962,711.87	7,481,251.57
48457	Tyler	Texas	21,766	2,750	2,011.00	3,523.00	1,330,496.23	657,592.00	2,551,379.04	2,421,413.56	595,524.46	8,058,822.48	3,751,909.79	1,458,371.33	9,842,242.30
48459	Upshur	Texas	39,309	5,524	4,112.50	6,954.50	2,447,951.19	1,398,452.31	4,149,536.60	4,281,853.98	1,265,470.08	13,027,040.75	6,729,805.18	2,995,362.58	15,696,951.68
48461	Upton	Texas	3,355	483	346.50	627.00	206,962.76	57,121.81	644,043.42	379,001.26	84,392.90	1,749,064.16	585,964.02	150,103.19	2,024,567.08
48463	Uvalde	Texas	26,405	3,957	2,954.00	4,993.00	1,607,125.61	850,569.96	2,789,344.26	2,689,270.30	730,819.96	9,613,592.89	4,296,395.91	1,784,395.30	11,432,383.17
48465	Val Verde	Texas	48,879	7,205	5,250.50	9,266.00	2,687,934.48	1,522,176.00	4,449,427.22	4,531,844.60	1,512,035.83	11,591,864.88	7,219,779.07	3,458,962.16	14,461,477.03
48467	Van Zandt	Texas	52,579	7,175	5,602.50	8,836.50	3,327,262.21	2,054,926.26	5,224,688.58	5,832,896.08	2,044,646.92	14,275,485.64	9,160,158.29	4,529,491.08	18,027,301.76
48469	Victoria	Texas	86,793	12,819	9,735.00	15,800.50	5,270,462.38	3,476,515.55	7,834,240.09	8,941,515.90	4,105,650.87	20,464,761.22	14,211,978.28	7,959,656.20	26,078,502.63
48471	Walker	Texas	67,861	8,229	6,403.00	10,096.50	3,181,318.46	1,906,919.61	4,927,199.05	5,174,903.17	1,773,803.98	13,835,891.88	8,356,221.63	4,250,458.72	16,989,316.86
48473	Waller	Texas	43,205	5,743	4,435.00	7,048.00	2,140,979.63	1,261,331.68	3,523,208.14	3,799,292.99	1,278,825.02	12,576,419.36	5,940,272.62	2,824,555.83	15,338,024.61
48475	Ward	Texas	10,658	1,467	1,024.50	1,904.00	611,264.01	248,034.71	1,382,035.86	1,194,372.56	284,966.80	4,904,888.37	1,805,636.57	575,979.75	5,763,837.37
48477	Washington	Texas	33,718	4,556	3,569.00	5,579.50	2,157,194.38	1,267,683.58	3,990,432.21	3,785,589.75	1,217,396.66	10,836,261.93	5,942,784.13	2,818,555.84	13,152,485.02
48479	Webb	Texas	250,304	37,727	26,398.50	49,850.50	11,675,995.50	7,558,674.48	16,754,555.83	17,463,199.13	8,206,314.46	33,373,660.00	29,139,194.64	16,468,842.09	47,971,568.15
48481	Wharton	Texas	41,280	5,796	4,469.50	7,186.50	2,401,640.46	1,419,326.81	4,254,347.37	3,989,764.44	1,299,254.28	10,794,838.53	6,391,404.90	3,063,097.14	13,471,482.19
48483	Wheeler	Texas	5,410	618	410.50	817.00	278,143.12	78,125.79	779,473.40	449,776.54	86,721.77	2,360,754.57	727,919.66	185,257.16	2,722,060.14
48485	Wichita	Texas	131,500	16,598	12,685.00	20,625.00	6,658,108.50	4,462,084.66	9,762,299.02	11,525,786.74	5,110,256.81	26,333,413.79	18,183,895.24	10,194,242.47	33,722,049.06
48487	Wilbarger	Texas	13,535	1,745	1,254.00	2,187.00	760,399.15	328,568.55	1,741,913.27	1,348,459.79	288,889.63	5,772,404.73	2,108,858.94	687,467.07	6,651,494.41
48489	Willacy	Texas	22,134	3,116	2,116.00	4,115.00	1,157,255.04	569,986.93	2,242,814.43	1,812,765.43	428,691.40	6,467,652.72	2,970,020.47	1,129,941.16	8,311,583.21
48491	Williamson	Texas	422,679	61,701	49,435.50	73,966.00	21,225,962.02	15,924,143.60	27,246,382.06	36,637,664.95	23,401,953.39	57,317,825.38	57,863,626.96	41,708,219.69	80,405,874.32
48493	Wilson	Texas	42,918	6,172	4,894.00	7,531.50	2,508,520.99	1,516,852.58	4,098,636.09	3,955,367.21	1,238,757.27	11,488,999.51	6,463,888.20	3,126,172.56	14,389,728.55
48495	Winkler	Texas	7,110	1,004	689.00	1,287.50	384,017.56	125,422.49	999,890.17	601,729.02	178,462.86	2,828,298.08	985,746.59	320,673.72	3,307,674.51
48497	Wise	Texas	59,127	7,862	6,142.00	9,593.50	3,189,033.58	2,028,591.22	5,051,495.46	5,382,797.79	2,032,774.41	14,920,443.46	8,571,831.37	4,403,140.92	18,268,972.62
48499	Wood	Texas	41,964	5,878	4,609.00	7,153.00	3,168,780.71	2,034,753.68	5,012,277.72	5,845,215.60	1,998,461.76	13,234,512.23	9,013,996.32	4,391,621.71	16,868,879.13
48501	Yoakum	Texas	7,879	1,133	827.50	1,433.50	417,721.97	161,449.42	1,004,250.12	778,966.60	202,734.64	4,201,243.32	1,196,688.57	385,734.19	4,810,413.01
48503	Young	Texas	18,550	2,462	1,850.50	3,054.50	1,159,486.78	579,865.69	2,212,981.26	2,166,261.90	582,327.22	7,764,188.32	3,325,748.67	1,311,309.86	9,252,532.05
48505	Zapata	Texas	14,018	2,108	1,373.00	2,785.00	730,402.91	312,007.20	1,516,375.13	1,147,935.29	279,765.57	5,132,815.44	1,878,338.20	677,309.25	6,018,302.21

48507	Zavala	Texas	11,677	1,745	1,259.50	2,237.00	650,043.55	270,001.82	1,565,267.03	982,016.28	176,934.73	4,886,890.31	1,632,059.82	499,979.47	5,836,142.88
49001	Beaver	Utah	6,629	531	263.50	814.00	199,793.10	44,792.31	591,616.14	347,596.52	57,522.69	1,882,440.66	547,389.62	115,402.47	2,329,004.71
49003	Box Elder	Utah	49,975	2,788	1,257.00	4,345.50	984,816.19	310,513.62	2,096,445.61	1,692,968.13	351,888.65	5,512,665.43	2,677,784.32	722,530.94	7,089,629.24
49005	Cache	Utah	112,656	5,748	1,722.50	9,850.00	1,800,310.00	440,892.48	3,490,994.91	2,753,565.17	395,126.38	9,450,967.86	4,553,875.17	886,261.58	12,103,672.07
49007	Carbon	Utah	21,403	1,196	506.50	1,911.00	492,521.74	117,119.21	1,238,700.03	909,903.43	130,907.06	4,445,747.13	1,402,425.17	273,878.90	4,941,203.70
49009	Daggett	Utah	1,059	52	10.00	97.00	28,785.43	510.10	168,285.12	44,743.06	1,587.26	297,234.29	73,528.49	2,216.07	423,996.03
49011	Davis	Utah	306,479	15,332	6,114.00	24,166.00	4,943,928.30	1,777,753.22	8,429,957.26	7,851,243.73	1,946,749.22	19,081,007.62	12,795,172.03	3,834,195.04	25,712,281.82
49013	Duchesne	Utah	18,607	1,034	332.00	1,703.00	352,294.99	73,928.16	900,550.43	648,432.02	80,059.02	3,367,960.08	1,000,727.00	167,177.40	3,761,053.90
49015	Emery	Utah	10,976	717	318.00	1,117.00	285,494.01	61,360.79	794,896.65	526,519.30	83,025.08	2,903,790.31	812,013.31	157,170.54	3,588,285.96
49017	Garfield	Utah	5,172	396	194.00	602.50	169,474.95	30,961.08	556,316.28	269,118.78	32,292.50	1,626,333.93	438,593.73	68,866.31	1,786,159.87
49019	Grand	Utah	9,225	561	245.00	887.50	234,847.68	46,310.74	730,083.78	362,575.89	43,085.52	1,992,035.76	597,423.57	101,361.71	2,615,621.07
49021	Iron	Utah	46,163	3,770	1,890.50	5,687.00	1,305,559.58	480,399.68	2,535,956.93	1,898,624.83	378,351.17	6,846,465.46	3,204,184.41	982,829.14	8,761,757.77
49023	Juab	Utah	10,246	688	287.50	1,087.00	223,881.20	42,753.46	642,402.76	360,819.27	48,203.77	2,087,462.23	584,700.47	96,284.16	2,520,050.19
49025	Kane	Utah	7,125	580	299.50	857.00	274,621.81	65,938.81	799,308.94	496,877.98	56,568.50	3,106,654.82	771,499.79	138,694.80	3,447,317.15
49027	Millard	Utah	12,503	930	409.00	1,458.50	358,348.40	90,561.23	915,896.99	657,526.08	96,250.12	3,297,903.88	1,015,874.48	222,740.92	3,734,610.36
49029	Morgan	Utah	9,469	476	158.50	815.00	169,586.90	22,071.45	603,632.96	279,539.37	29,451.16	1,735,657.97	449,126.26	56,745.63	2,046,104.29
49031	Piute	Utah	1,556	120	54.00	185.50	56,346.37	5,016.34	244,447.40	92,782.34	8,855.21	815,276.53	149,128.71	15,140.83	863,403.05
49033	Rich	Utah	2,264	113	31.00	202.00	43,178.06	2,222.43	206,847.87	61,105.14	3,968.78	533,829.79	104,283.19	7,629.73	632,119.75
49035	Salt Lake	Utah	1,029,655	48,050	18,412.50	75,535.00	16,264,308.44	6,284,144.73	26,346,240.33	28,488,330.92	8,883,709.37	56,927,230.82	44,752,639.36	16,129,628.35	81,045,245.15
49037	San Juan	Utah	14,746	1,194	603.50	1,783.50	414,138.82	112,233.31	987,230.36	739,280.90	117,921.18	3,312,630.65	1,153,419.72	259,250.96	3,797,533.22
49039	Sanpete	Utah	27,822	1,702	763.50	2,612.00	600,926.69	178,164.17	1,315,504.41	919,198.64	131,604.05	3,726,620.00	1,520,125.33	358,879.10	4,493,554.47
49041	Sevier	Utah	20,802	1,488	680.50	2,299.50	576,868.04	159,485.90	1,341,609.27	1,020,702.33	137,913.03	4,396,732.01	1,597,570.37	323,020.22	5,032,479.67
49043	Summit	Utah	36,324	1,770	572.50	3,026.00	657,258.14	156,688.32	1,688,846.32	966,291.91	105,408.35	4,664,861.41	1,623,550.05	268,226.15	6,055,783.54
49045	Tooele	Utah	58,218	3,571	1,518.00	5,685.00	1,105,749.25	336,078.07	2,336,525.97	1,851,605.16	364,850.66	7,244,408.32	2,957,354.41	761,904.88	8,582,434.99
49047	Uintah	Utah	32,588	1,845	611.50	3,045.50	623,073.83	155,930.64	1,503,070.76	1,050,954.79	158,027.20	4,222,287.25	1,674,028.63	339,665.82	5,220,493.00
49049	Utah	Utah	516,564	28,384	11,585.50	46,103.00	8,169,149.01	3,148,442.48	13,750,497.43	12,491,773.02	3,496,313.19	26,792,364.19	20,660,922.03	6,898,719.44	39,510,683.04
49051	Wasatch	Utah	23,530	1,215	459.00	1,987.50	408,942.32	77,969.65	1,056,965.78	598,894.19	89,134.76	2,703,268.14	1,007,836.52	185,598.21	3,472,438.66
49053	Washington	Utah	138,115	12,116	6,800.00	17,410.00	5,001,707.49	2,528,239.69	8,096,529.85	8,757,375.81	2,983,286.73	20,215,545.39	13,759,083.30	5,944,173.61	25,797,735.40
49055	Wayne	Utah	2,778	201	89.50	311.50	89,340.28	10,563.60	329,947.74	121,194.71	14,376.34	977,191.25	210,534.99	25,482.45	1,183,187.76
49057	Weber	Utah	231,236	11,391	4,322.50	18,766.00	4,003,163.06	1,307,426.48	7,386,884.87	6,649,811.70	1,324,816.14	18,324,955.44	10,652,974.76	2,725,962.56	23,695,531.99
50001	Addison	Vermont	36,821	2,288	1,104.50	3,506.00	1,016,749.93	357,957.15	2,172,232.90	1,737,893.84	312,410.62	7,197,009.34	2,754,643.77	748,476.67	8,354,215.26
50003	Bennington	Vermont	37,125	2,425	1,316.00	3,556.50	1,192,836.78	454,044.43	2,378,491.56	2,220,562.14	452,280.17	8,085,884.84	3,413,398.92	969,740.94	9,656,778.28
50005	Caledonia	Vermont	31,227	1,665	481.00	2,814.50	766,331.29	143,929.18	1,797,401.82	1,331,247.46	127,516.74	6,430,143.94	2,097,578.74	298,092.78	7,562,907.80
50007	Chittenden	Vermont	156,545	9,345	4,099.00	14,553.00	3,777,799.65	1,406,729.52	6,681,410.17	6,713,448.43	1,703,006.25	16,632,193.26	10,491,248.08	3,466,813.55	21,627,575.00
50009	Essex	Vermont	6,306	319	94.50	547.00	174,841.52	18,245.83	562,971.94	322,451.67	16,915.67	2,026,748.41	497,293.19	34,750.43	2,397,962.96
50011	Franklin	Vermont	47,746	2,927	1,090.50	4,771.00	1,193,330.99	316,441.39	2,500,527.67	2,346,502.33	315,600.93	8,750,603.03	3,539,833.32	705,909.64	10,587,904.40
50013	Grand Isle	Vermont	6,970	428	171.50	688.50	199,266.86	28,925.57	710,927.63	309,703.87	31,394.19	1,588,354.77	508,970.73	69,061.61	2,096,798.63
50015	Lamoille	Vermont	24,475	1,441	595.00	2,279.00	615,924.06	155,962.86	1,498,562.02	1,020,910.34	123,134.62	4,669,087.50	1,636,834.41	305,606.40	5,396,186.48
50017	Orange	Vermont	28,936	1,631	635.00	2,602.50	782,685.70	186,773.33	1,759,205.48	1,255,763.21	159,541.81	5,955,903.70	2,038,448.91	383,911.55	6,816,928.95
50019	Orleans	Vermont	27,231	1,532	506.00	2,508.00	735,373.27	158,341.01	1,756,288.55	1,267,711.99	130,143.13	6,010,893.19	2,003,085.26	295,046.88	7,157,545.91
50021	Rutland	Vermont	61,642	3,771	1,744.00	5,852.50	1,822,680.65	640,776.87	3,723,835.45	3,406,662.54	647,393.23	11,420,523.15	5,229,343.19	1,510,279.35	14,247,706.98
50023	Washington	Vermont	59,534	3,409	1,506.50	5,388.00	1,521,110.23	483,534.39	3,086,976.78	2,848,131.04	503,988.11	9,669,552.29	4,369,241.27	1,109,087.84	11,883,962.81
50025	Windham	Vermont	44,513	2,637	1,284.00	4,107.50	1,263,212.15	446,537.18	2,550,283.72	2,163,806.36	425,981.18	6,980,764.04	3,427,018.51	986,321.72	8,478,377.89
50027	Windsor	Vermont	56,670	3,223	1,281.50	5,034.50	1,576,543.71	480,598.03	3,035,190.14	2,988,363.40	518,906.56	10,303,211.14	4,564,907.11	1,150,579.18	12,244,017.59
51001	Accomack	Virginia	33,164	3,289	2,307.50	4,313.50	1,616,207.30	824,133.94	3,043,081.65	2,926,303.91	762,676.66	9,593,013.59	4,542,511.21	1,806,632.78	11,630,244.87
51003	Albemarle	Virginia	98,970	9,488	6,586.00	12,405.50	4,066,229.49	2,293,655.71	6,412,945.76	7,678,413.66	3,116,825.98	19,375,984.57	11,744,643.15	5,881,064.14	24,118,595.64
51005	Alleghany	Virginia	16,250	1,339	839.00	1,819.50	687,263.19	265,547.46	1,538,400.57	1,253,961.57	257,004.96	5,081,849.34	1,941,224.77	585,773.72	6,133,564.23
51007	Amelia	Virginia	12,690	1,274	906.00	1,663.50	570,034.12	218,659.03	1,218,319.55	990,788.67	189,149.17	4,360,511.18	1,560,822.79	472,209.30	5,232,718.69
51009	Amherst	Virginia	32,353	2,880	1,880.00	3,898.50	1,333,965.92	637,148.38	2,512,186.80	2,324,611.26	576,485.27	7,919,565.32	3,658,577.18	1,393,478.64	9,624,613.28
51011	Appomattox	Virginia	14,973	1,359	885.00	1,825.50	631,846.38	254,628.04	1,370,415.34	1,104,839.53	210,750.16	4,312,229.86	1,736,685.91	535,295.95	5,702,237.84
51013	Arlington	Virginia	207,627	19,825	15,128.50	24,513.50	7,258,182.92	4,969,154.06	10,293,931.04	15,799,749.24	9,103,129.40	29,342,198.22	23,057,932.16	14,959,416.95	37,782,810.02
51015	Augusta	Virginia	73,750	6,830	4,506.00	9,037.00	3,088,908.64	1,708,865.31	5,133,121.42	5,777,162.24	1,889,533.60	14,519,109.66	8,866,070.88	3,976,483.40	18,580,806.66
51017	Bath	Virginia	4,731	395	253.50	549.50	225,467.69	55,615.26	745,059.99	491,779.82	59,220.01	3,286,572.49	717,247.50	124,450.47	3,433,767.29
51019	Bedford	Virginia	68,676	5,761	3,596.50	7,753.00	2,674,474.63	1,394,088.63	4,479,820.88	4,832,907.36	1,413,596.33	15,011,970.54	7,507,381.99	3,195,278.47	17,944,660.80
51021	Bland	Virginia	6,824	487	267.00	696.50	229,417.41	58,972.65	660,173.58	389,634.52	67,372.62	2,027,479.02	619,051.94	135,006.25	2,396,417.63
51023	Botetourt	Virginia	33,148	2,736	1,717.00	3,722.00	1,283,830.58	583,187.81	2,515,577.81	2,420,848.92	497,865.00	8,295,728.00	3,704,679.50	1,193,044.05	10,217,486.57
51025	Brunswick	Virginia	17,434	1,659	1,139.50	2,205.50	761,398.89	320,682.17	1,549,148.06	1,372,162.78	263,393.58	5,255,204.24	2,133,561.66	661,414.77	6,000,040.04
51027	Buchanan	Virginia	24,098	1,693	939.50	2,388.00	823,054.60	301,538.66	1,845,308.38	1,619,077.23	298,776.88	6,552,314.44	2,442,131.83	671,577.70	7,476,378.89
51029	Buckingham	Virginia	17,146	1,610	1,109.50	2,128.50	720,637.68	307,163.76	1,617,434.49	1,235,328.71	259,593.63	5,045,249.33	1,955,966.39	654,235.01	6,303,816.84
51031	Campbell	Virginia	54,842	4,784	3,277.50	6,409.50	2,163,383.43	1,143,769.89	3,892,961.21	3,929,156.70	1,318,040.68	10,827,872.96	6,092,540.13	2,666,354.03	13,235,971.14
51033	Caroline	Virginia	28,545	3,040	2,225.50	3,845.50	1,273,238.22	671,020.08	2,394,978.57	2,105,085.81	585,431.17	7,228,771.62	3,378,324.04	1,385,539.24	8,915,027.14
51035	Carroll	Virginia	30,042	2,214	1,341.50	3,122.50	1,071,394.02	427,399.52	2,148,094.08	1,826,278.75	344,153.24	6,173,053.26	2,897,672.76	881,960.51	7,582,157.92
51036	Charles City	Virginia	7,256	726	532.00	925.50	366,038.27	112,758.30	1,107,348.44	639,036.50	131,874.72	3,060,564.62	1,005,074.77	267,047.68	3,600,238.42
51037	Charlotte	Virginia	12,586	1,170	777.00	1,535.50	543,157.64	212,877.49	1,207,541.04	955,018.22	165,730.27	4,334,004.24	1,498,175.86	440,816.08	4,997,016.82
51041	Chesterfield	Virginia	316,236	32,741	24,067.50	41,793.50	12,620,086.88	8,694,025.40	17,161,812.40	22,220,583.11	11,754,279.78	42,137,787.67	34,840,669.99	21,732,282.00	56,175,643.40
51043	Clarke	Virginia	14,034	1,432	1,038.00	1,857.50	684,344.63	290,333.22	1,567,660.17	1,214,909.43	281,351.62	5,427,134.27	1,899,254.06	646,289.40	6,168,473.97

51045	Craig	Virginia	5,190	417	260.00	573.50	210,788.85	45,072.11	641,818.95	324,734.08	49,536.54	1,993,315.50	535,522.93	103,175.94	2,518,488.36
51047	Culpeper	Virginia	46,689	4,878	3,504.00	6,271.00	1,972,283.83	1,036,601.30	3,562,078.64	3,369,810.49	1,064,756.90	10,169,916.34	5,342,094.31	2,366,073.19	12,257,114.31
51049	Cumberland	Virginia	10,052	995	706.00	1,306.00	448,423.60	149,887.26	1,045,142.61	842,693.61	129,323.96	3,867,250.05	1,291,117.21	309,134.04	4,531,463.66
51051	Dickenson	Virginia	15,903	1,151	647.50	1,699.00	551,799.10	168,472.56	1,265,040.06	1,014,827.82	179,213.30	4,234,363.57	1,566,626.93	395,284.94	4,847,599.30
51053	Dinwiddie	Virginia	28,001	2,811	1,988.00	3,642.00	1,271,222.14	571,017.79	2,473,750.09	1,928,341.28	523,700.81	6,011,007.99	3,199,563.42	1,255,523.67	7,820,838.89
51057	Essex	Virginia	11,151	1,149	824.00	1,459.50	535,658.22	215,270.92	1,268,607.07	955,799.97	170,021.94	4,884,355.19	1,491,458.20	420,865.24	5,454,357.16
51059	Fairfax	Virginia	1,081,726	111,132	84,714.50	138,343.00	42,582,367.14	31,436,128.74	54,874,791.34	90,593,544.65	61,064,739.33	133,593,707.20	133,175,911.80	94,651,158.61	181,832,884.00
51061	Fauquier	Virginia	65,203	6,765	4,970.50	8,579.00	2,771,214.60	1,661,539.46	4,275,488.70	5,007,878.99	1,878,283.70	12,802,002.28	7,779,093.59	4,041,863.15	15,910,264.32
51063	Floyd	Virginia	15,279	1,188	743.50	1,615.00	564,762.22	220,534.61	1,388,669.46	1,000,847.18	151,106.91	4,834,561.47	1,565,609.40	421,178.63	5,369,136.44
51065	Fluvanna	Virginia	25,691	2,569	1,797.00	3,320.50	1,134,633.01	529,502.58	2,233,028.53	2,024,464.30	464,682.15	7,915,430.97	3,159,097.31	1,089,661.70	9,019,123.49
51067	Franklin	Virginia	56,159	4,467	2,791.00	6,063.00	2,164,582.19	1,033,811.90	4,005,888.47	3,827,838.73	958,919.76	13,678,017.23	5,992,420.92	2,344,059.28	15,632,039.00
51069	Frederick	Virginia	78,305	7,854	5,385.00	10,085.50	3,207,827.73	1,806,771.09	5,227,360.56	5,583,755.96	2,036,846.26	13,536,411.96	8,791,583.69	4,245,114.89	17,483,023.01
51071	Giles	Virginia	17,286	1,310	754.00	1,844.50	613,686.55	215,065.73	1,416,099.83	1,185,904.99	219,962.05	4,920,171.39	1,799,591.53	490,186.06	5,614,922.03
51073	Gloucester	Virginia	36,858	3,645	2,673.50	4,700.50	1,641,894.10	843,524.76	3,069,202.01	2,803,212.98	737,542.23	9,995,607.77	4,445,107.08	1,816,569.04	11,970,896.16
51075	Goochland	Virginia	21,717	2,190	1,509.00	2,849.00	1,012,749.27	452,598.17	2,037,370.15	2,148,426.86	714,628.86	6,873,858.78	3,161,176.13	1,277,703.49	8,113,759.05
51077	Grayson	Virginia	15,533	1,114	679.00	1,585.00	576,105.53	190,042.43	1,355,711.92	995,593.48	143,477.46	4,228,755.37	1,571,699.01	396,691.60	4,997,145.39
51079	Greene	Virginia	18,403	1,839	1,278.50	2,404.00	750,939.47	323,175.33	1,660,752.88	1,253,354.32	254,627.84	5,612,598.05	2,004,293.79	621,584.49	6,575,040.98
51081	Greensville	Virginia	12,243	1,155	802.00	1,518.00	488,330.37	167,673.95	1,188,184.74	809,272.47	148,954.16	3,924,634.31	1,297,602.84	352,260.26	4,586,009.15
51083	Halifax	Virginia	36,241	3,152	2,131.50	4,224.50	1,585,694.41	736,353.55	3,044,165.78	2,830,125.32	686,602.54	8,535,648.27	4,415,819.73	1,636,980.28	10,304,402.77
51085	Hanover	Virginia	99,863	10,550	7,631.00	13,284.00	4,405,727.44	2,750,176.01	6,742,380.74	7,943,193.30	3,204,640.25	18,710,176.86	12,348,920.74	6,328,452.96	23,273,445.23
51087	Henrico	Virginia	306,935	32,622	24,450.50	41,572.50	13,037,472.00	9,145,753.78	18,022,160.96	24,583,973.89	13,574,799.27	43,407,277.34	37,621,445.89	23,881,987.29	58,783,125.67
51089	Henry	Virginia	54,151	4,269	2,718.50	5,761.50	2,097,671.42	1,053,797.97	3,577,666.64	3,808,238.04	1,034,331.12	11,724,679.28	5,905,909.46	2,410,950.16	13,877,894.87
51091	Highland	Virginia	2,321	200	124.00	276.00	117,747.28	20,951.50	413,886.96	191,654.31	22,753.87	1,471,117.76	309,401.59	47,294.50	1,729,755.78
51093	Isle of Wight	Virginia	35,270	3,574	2,600.00	4,565.00	1,595,214.74	829,126.30	3,043,771.39	2,940,790.63	878,318.81	9,263,973.22	4,536,005.37	1,963,674.23	11,073,854.20
51095	James City	Virginia	67,009	6,892	4,955.00	8,877.50	3,428,140.41	2,078,808.85	5,432,985.62	6,536,419.70	2,304,994.84	16,825,131.70	9,964,560.11	4,824,557.81	21,096,606.23
51097	King and Queen	Virginia	6,945	710	517.50	898.50	334,395.44	109,810.09	874,407.74	637,646.72	129,596.35	3,202,579.39	972,042.16	263,726.07	3,711,931.03
51099	King George	Virginia	23,584	2,486	1,836.50	3,141.00	925,144.85	458,015.50	1,794,954.07	2,055,120.52	746,733.33	7,501,614.59	2,980,265.37	1,309,132.30	8,628,619.25
51101	King William	Virginia	15,935	1,661	1,198.00	2,113.50	676,981.83	303,645.54	1,504,729.74	1,194,338.76	281,813.68	5,804,969.10	1,871,320.58	619,289.15	6,558,633.06
51103	Lancaster	Virginia	11,391	1,127	797.00	1,446.50	723,668.28	280,632.01	1,642,643.43	1,449,218.52	249,401.67	5,424,285.73	2,172,886.80	614,945.54	6,460,046.39
51105	Lee	Virginia	25,587	1,874	1,133.50	2,618.50	853,808.93	375,363.84	1,736,514.19	1,398,523.53	302,135.95	5,561,759.92	2,252,332.47	735,511.34	6,740,435.05
51107	Loudoun	Virginia	312,311	33,233	25,311.50	41,101.00	10,515,530.02	7,539,021.30	14,738,267.61	19,422,921.78	10,818,337.40	34,913,539.96	29,938,451.80	19,076,955.33	47,789,312.63
51109	Louisa	Virginia	33,153	3,442	2,461.00	4,396.50	1,573,712.27	767,674.92	2,976,551.00	2,910,048.67	921,554.17	8,305,533.78	4,483,760.95	1,863,098.59	10,306,668.67
51111	Lunenburg	Virginia	12,914	1,216	818.00	1,602.00	579,953.97	219,482.66	1,328,843.60	1,008,243.90	188,455.83	4,538,947.76	1,588,197.87	458,590.34	5,294,920.76
51113	Madison	Virginia	13,308	1,335	941.50	1,725.50	636,818.41	262,896.68	1,340,038.69	1,031,617.44	214,503.35	4,324,959.85	1,668,435.85	554,282.71	5,156,416.14
51115	Mathews	Virginia	8,978	875	624.00	1,132.00	485,834.64	188,698.47	1,078,252.25	837,926.45	147,903.20	3,911,308.32	1,323,761.10	372,488.29	4,665,714.39
51117	Mecklenburg	Virginia	32,727	3,027	2,084.50	4,008.50	1,535,027.28	782,053.24	2,860,241.92	2,729,236.06	735,135.82	8,570,884.04	4,264,263.34	1,761,658.18	10,542,122.11
51119	Middlesex	Virginia	10,959	1,078	752.00	1,389.00	605,744.93	242,404.24	1,313,414.16	1,108,985.08	205,026.30	4,734,765.77	1,714,730.01	496,868.48	5,473,700.47
51121	Montgomery	Virginia	94,392	6,817	4,060.00	9,614.00	2,499,009.50	1,240,879.08	4,250,461.68	4,196,204.67	1,208,539.28	13,090,567.45	6,695,214.17	2,709,210.27	15,825,884.98
51125	Nelson	Virginia	15,020	1,368	926.00	1,789.50	713,863.74	292,459.93	1,579,371.09	1,252,490.53	234,708.87	4,744,337.59	1,966,354.26	597,230.10	5,738,334.81
51127	New Kent	Virginia	18,429	1,883	1,338.00	2,362.00	811,720.04	344,497.54	1,673,179.27	1,380,068.14	299,315.76	5,458,969.76	2,191,788.18	711,249.66	6,429,851.97
51131	Northampton	Virginia	12,389	1,230	853.00	1,611.50	652,020.12	267,292.42	1,374,610.54	1,167,641.71	247,985.49	4,518,309.89	1,819,661.83	594,638.89	5,427,571.87
51133	Northumberland	Virginia	12,330	1,212	860.00	1,549.50	752,197.41	329,694.96	1,575,979.00	1,616,873.78	261,369.50	6,863,360.11	2,266,071.19	668,807.53	7,868,375.29
51135	Nottoway	Virginia	15,853	1,555	1,063.00	2,047.50	733,829.56	303,337.32	1,559,344.32	1,242,770.78	260,911.47	4,723,419.07	1,976,600.34	622,092.16	5,670,374.74
51137	Orange	Virginia	33,481	3,495	2,506.00	4,454.50	1,626,110.69	870,334.44	3,123,045.42	2,975,450.19	851,835.48	10,063,683.99	4,601,560.88	1,924,935.78	11,617,943.41
51139	Page	Virginia	24,042	2,381	1,631.00	3,102.50	1,113,711.66	552,515.75	2,220,228.99	1,787,341.99	416,847.18	6,306,068.10	2,901,053.65	1,075,184.07	7,723,097.47
51141	Patrick	Virginia	18,490	1,389	868.50	1,913.50	721,184.29	265,033.22	1,534,101.94	1,285,390.16	202,342.77	4,968,144.83	2,006,574.45	549,582.34	5,752,696.23
51143	Pittsylvania	Virginia	63,506	5,222	3,484.50	7,034.00	2,512,784.50	1,310,171.90	4,380,343.72	4,499,903.50	1,350,769.13	14,744,422.24	7,012,688.00	2,928,138.62	17,477,944.15
51145	Powhatan	Virginia	28,046	2,825	2,029.00	3,661.00	1,154,975.95	571,996.91	2,232,155.86	1,942,649.20	524,803.56	6,218,276.50	3,097,625.15	1,227,517.88	7,601,045.72
51147	Prince Edward	Virginia	23,368	2,109	1,444.00	2,785.00	871,899.74	390,090.75	1,870,961.14	1,643,808.64	294,598.66	6,905,945.10	2,515,708.39	803,179.42	8,154,605.61
51149	Prince George	Virginia	35,725	3,657	2,727.00	4,594.50	1,419,374.82	765,920.46	2,655,600.39	2,579,514.44	863,854.37	8,157,544.27	3,998,889.26	1,861,885.92	9,818,478.77
51153	Prince William	Virginia	402,002	42,757	32,118.00	53,638.50	14,036,762.76	9,821,164.11	19,013,941.62	23,340,421.30	13,184,070.50	41,762,743.08	37,377,184.05	24,000,926.79	57,403,482.27
51155	Pulaski	Virginia	34,872	2,580	1,519.50	3,693.00	1,258,054.63	531,419.42	2,574,365.59	2,295,079.11	495,005.00	8,718,516.15	3,553,133.74	1,156,294.55	10,060,366.88
51157	Rappahannock	Virginia	7,373	729	495.50	956.00	376,369.02	129,984.44	1,027,463.43	731,616.10	141,201.82	3,447,490.67	1,107,985.12	291,227.40	3,842,605.10
51159	Richmond	Virginia	9,254	923	662.00	1,184.00	457,700.62	158,952.39	1,231,849.56	903,819.51	158,087.36	4,016,190.75	1,361,520.14	358,631.96	4,434,184.02
51161	Roanoke	Virginia	92,376	7,319	4,657.00	9,972.00	3,463,866.16	1,828,549.76	5,885,452.38	6,345,175.80	2,238,166.06	16,577,059.29	9,809,041.96	4,505,736.72	21,125,521.51
51163	Rockbridge	Virginia	22,307	1,928	1,184.50	2,621.00	1,006,663.59	407,201.04	1,961,119.87	1,805,215.22	379,840.23	6,448,285.86	2,811,878.81	898,689.07	7,662,466.31
51165	Rockingham	Virginia	76,314	7,364	5,127.00	9,810.00	3,258,925.53	1,906,990.56	5,227,326.56	5,889,962.94	2,112,149.16	15,504,383.08	9,148,888.47	4,402,924.47	19,901,188.48
51167	Russell	Virginia	28,897	2,066	1,132.00	2,919.50	983,000.06	362,212.21	2,086,953.82	1,716,243.32	324,282.05	6,222,269.65	2,699,243.38	753,463.86	7,269,358.46
51169	Scott	Virginia	23,177	1,668	939.50	2,345.50	828,438.53	316,059.16	1,777,587.02	1,585,987.44	256,601.35	6,940,448.48	2,414,425.97	650,951.17	7,827,594.43
51171	Shenandoah	Virginia	41,993	4,179	2,910.50	5,449.00	1,974,694.17	1,034,398.65	3,329,130.06	3,592,716.53	1,002,832.56	11,239,985.17	5,567,410.70	2,251,244.80	13,343,621.99
51173	Smyth	Virginia	32,208	2,348	1,344.50	3,309.50	1,125,619.86	453,131.13	2,216,842.56	2,047,642.72	421,648.29	7,098,777.90	3,173,262.58	937,099.93	8,579,203.28
51175	Southampton	Virginia	18,570	1,869	1,343.50	2,412.50	857,589.11	384,241.79	1,762,062.60	1,408,830.21	310,014.28	5,653,826.60	2,266,409.32	766,405.75	6,452,263.96
51177	Spotsylvania	Virginia	122,397	13,030	9,741.00	16,573.00	4,810,469.88	3,109,759.53	7,494,357.53	7,806,720.97	3,197,468.96	18,147,958.28	12,617,190.85	6,808,906.84	23,315,218.11
51179	Stafford	Virginia	128,961	13,505	9,850.00	17,134.50	4,548,086.30	2,807,878.43	7,085,417.88	7,552,688.84	3,518,405.83	17,928,714.00	12,100,775.14	6,723,620.65	22,413,366.41
51181	Surry	Virginia	7,058	722	515.50	922.50	339,735.72	110,255.38	901,615.97	693,755.94	228,254.48	2,859,824.14	1,033,491.66	365,429.04	3,220,689.26
51183	Sussex	Virginia	12,087	1,179	845.00	1,528.50	525,043.81	191,615.17	1,229,131.52	945,343.50	181,911.30	4,551,380.26	1,470,387.30	428,236.84	5,372,866.73

51185	Tazewell	Virginia	45,078	3,259	1,903.00	4,625.50	1,556,366.23	674,872.34	3,028,001.20	2,862,663.57	627,970.48	9,669,532.82	4,419,029.80	1,545,937.32	11,658,470.79
51187	Warren	Virginia	37,575	3,818	2,723.50	5,002.50	1,567,067.47	763,037.50	2,861,391.53	2,708,224.14	687,980.39	8,887,109.47	4,275,291.61	1,672,426.13	11,212,139.67
51191	Washington	Virginia	54,876	3,886	2,263.00	5,429.00	1,869,633.26	865,275.41	3,377,221.50	3,335,459.71	828,358.40	10,122,217.47	5,205,092.97	1,904,747.02	13,018,337.34
51193	Westmoreland	Virginia	17,454	1,780	1,320.00	2,234.00	918,685.18	456,543.97	1,911,052.25	1,536,461.00	326,490.42	5,750,359.51	2,455,146.18	871,436.66	6,896,778.03
51195	Wise	Virginia	41,452	2,990	1,781.50	4,203.00	1,285,707.83	577,056.69	2,552,090.85	2,283,509.59	533,688.01	7,805,177.16	3,569,217.42	1,250,674.72	9,209,976.34
51197	Wythe	Virginia	29,235	2,139	1,222.50	3,033.50	1,006,493.78	410,229.80	1,980,230.54	1,794,464.73	336,059.49	7,195,899.85	2,800,958.52	846,518.40	8,251,280.82
51199	York	Virginia	65,464	6,610	4,713.50	8,563.00	2,649,369.15	1,511,494.44	4,451,193.18	4,704,284.90	1,530,032.68	12,872,373.62	7,353,654.05	3,361,418.16	16,349,458.83
51510	Alexandria	Virginia	139,966	13,727	10,491.00	17,010.00	5,193,629.83	3,408,799.24	7,870,927.71	10,817,434.85	5,157,846.14	24,020,831.13	16,011,064.68	9,011,630.04	29,839,288.85
51515	Bedford City	Virginia	6,222	541	342.50	752.00	265,430.68	69,770.79	677,710.30	545,340.87	77,104.54	2,744,941.88	810,771.55	174,755.78	3,175,378.90
51520	Bristol	Virginia	17,835	1,311	735.00	1,882.00	632,476.58	199,693.54	1,558,556.01	1,088,392.83	218,294.43	4,436,811.67	1,720,869.41	491,441.01	5,466,353.17
51530	Buena Vista	Virginia	6,650	591	372.50	800.50	257,057.22	71,284.11	726,302.21	466,689.09	72,120.49	2,304,342.97	723,746.31	160,054.23	2,698,263.55
51540	Charlottesville	Virginia	43,475	4,012	2,850.00	5,236.50	1,464,201.23	751,557.11	2,657,391.48	2,547,567.02	776,649.37	8,246,829.51	4,011,768.24	1,726,997.72	10,028,682.49
51550	Chesapeake	Virginia	222,209	22,333	16,027.50	28,787.50	8,495,186.80	5,484,923.47	12,221,629.07	14,563,507.26	6,850,632.14	33,126,849.40	23,058,694.06	13,159,790.87	42,651,585.32
51560	Clifton Forge	Virginia	0	0	0.00	0.00	0.00	0.00	0.00	0.00	0.00	0.00	0.00	0.00	0.00
51570	Colonial Heights	Virginia	17,411	1,827	1,355.50	2,353.00	854,192.48	419,503.00	1,699,189.49	1,617,705.16	361,970.53	6,667,052.80	2,471,897.64	871,406.41	7,542,868.77
51580	Covington	Virginia	5,961	491	295.00	683.00	219,919.71	54,845.51	624,098.01	441,079.87	71,166.53	2,454,366.60	660,999.59	132,793.46	2,817,396.21
51590	Danville	Virginia	43,055	3,552	2,338.00	4,749.00	1,711,185.12	865,634.10	3,134,168.74	3,160,487.70	807,634.00	9,773,608.98	4,871,672.82	1,869,992.87	11,683,990.23
51595	Emporia	Virginia	5,927	604	413.50	798.00	271,325.74	77,956.41	782,688.00	428,041.42	78,053.19	2,230,876.76	699,367.16	168,358.73	2,679,629.39
51600	Fairfax City	Virginia	22,565	2,275	1,722.00	2,849.50	972,961.62	467,627.31	1,930,186.01	2,226,587.77	816,362.23	6,718,079.54	3,199,549.39	1,411,884.85	8,051,693.72
51610	Falls Chruch	Virginia	12,332	1,243	914.00	1,538.00	516,271.45	185,928.47	1,238,225.57	1,108,453.03	375,838.30	3,811,332.66	1,624,724.49	601,277.27	4,509,165.90
51620	Franklin City	Virginia	8,582	900	632.00	1,175.00	422,930.57	143,598.80	1,035,258.44	684,498.56	131,160.36	3,217,242.34	1,107,429.13	309,758.88	4,155,558.41
51630	Fredericksburg	Virginia	24,286	2,480	1,800.50	3,149.00	916,226.24	423,841.78	1,852,435.56	1,565,045.84	407,455.44	5,482,337.61	2,481,272.09	952,493.31	6,838,249.16
51640	Galax	Virginia	7,042	524	300.00	747.50	244,192.96	64,314.62	693,421.64	412,922.90	59,478.27	2,145,261.06	657,115.86	136,966.18	2,582,831.39
51650	Hampton	Virginia	137,436	13,671	9,875.50	17,447.00	5,468,454.32	3,417,200.81	7,961,556.08	10,214,988.00	4,597,174.67	23,664,394.40	15,683,442.32	8,507,063.03	29,890,061.01
51660	Harrisonburg	Virginia	48,914	4,426	3,052.00	5,815.00	1,491,640.57	785,844.83	2,839,736.72	2,342,926.93	648,802.13	8,687,126.25	3,834,567.50	1,565,498.46	10,470,097.40
51670	Hopewell	Virginia	22,591	2,449	1,787.50	3,120.50	1,037,836.27	510,631.79	2,128,324.48	2,118,364.25	626,113.96	6,591,028.60	3,156,200.52	1,269,871.28	8,210,222.16
51678	Lexington	Virginia	7,042	560	372.00	756.00	233,893.23	70,837.31	639,596.00	437,884.57	68,986.37	2,414,344.51	671,777.80	150,556.13	2,747,833.37
51680	Lynchburg	Virginia	75,568	6,616	4,388.50	9,023.50	2,712,003.74	1,511,482.89	4,565,460.75	4,968,146.56	1,678,548.89	14,580,076.60	7,680,150.30	3,469,374.02	17,415,124.85
51683	Manassas City	Virginia	37,821	4,014	3,006.00	5,052.50	1,347,174.99	676,845.99	2,778,980.78	2,169,297.10	767,706.19	6,903,366.71	3,516,472.10	1,579,677.12	9,006,345.80
51685	Manassas Park City	Virginia	14,273	1,520	1,101.50	1,921.00	452,840.09	193,106.07	937,411.84	736,477.27	239,734.35	3,354,320.93	1,189,317.36	460,978.45	4,067,413.89
51690	Martinsville	Virginia	13,821	1,102	674.00	1,518.50	528,305.38	175,658.81	1,232,227.47	1,146,332.46	159,656.10	5,209,657.67	1,674,637.85	375,277.09	5,805,956.88
51700	Newport News	Virginia	180,719	18,494	13,248.50	23,626.00	6,852,647.39	4,451,660.98	10,005,684.18	12,152,362.68	5,868,841.97	25,609,904.32	19,005,010.08	11,029,383.02	33,732,168.44
51710	Norfolk	Virginia	242,803	23,735	17,365.50	30,211.50	8,602,359.66	5,629,445.14	11,993,435.82	15,911,818.73	7,943,233.53	30,333,734.48	24,514,178.39	14,005,573.33	40,233,766.32
51720	Norton	Virginia	3,958	291	164.00	418.00	122,268.55	23,951.02	395,498.44	250,422.47	36,359.10	1,544,271.51	372,691.02	64,321.38	1,731,076.38
51730	Petersburg	Virginia	32,420	3,389	2,434.50	4,316.50	1,478,211.43	755,260.51	2,693,554.81	2,614,446.91	674,562.42	8,542,006.84	4,092,658.34	1,619,987.33	10,456,315.27
51735	Poquoson City	Virginia	12,150	1,208	871.50	1,550.50	555,971.08	215,895.07	1,429,928.02	935,261.53	206,437.92	4,239,417.62	1,491,232.61	489,767.48	4,930,963.40
51740	Portsmouth	Virginia	95,535	9,749	6,987.00	12,501.00	3,942,106.84	2,388,888.11	5,929,007.30	7,519,065.08	3,258,396.98	18,524,092.09	11,461,171.92	6,129,185.19	23,452,253.17
51750	Radford	Virginia	16,408	1,139	664.50	1,619.50	388,826.77	136,825.71	941,567.17	614,287.33	127,875.96	2,675,694.08	1,003,114.11	278,314.85	3,238,496.18
51760	Richmond City	Virginia	204,214	20,994	15,421.50	26,775.00	8,293,624.60	5,468,962.98	11,917,213.44	16,115,168.74	8,158,163.83	31,235,115.08	24,408,793.35	14,348,552.52	41,736,222.25
51770	Roanoke City	Virginia	97,032	7,862	5,020.50	10,989.50	3,403,083.17	1,770,093.92	5,564,451.77	5,877,931.87	2,034,634.79	15,135,870.52	9,281,015.03	4,144,877.73	19,147,327.98
51775	Salem	Virginia	24,802	1,934	1,184.00	2,707.00	890,844.55	342,880.28	1,816,298.51	1,757,292.80	397,802.06	6,565,182.23	2,648,137.34	883,618.37	7,619,594.09
51780	South Boston	Virginia	0	0	0.00	0.00	0.00	0.00	0.00	0.00	0.00	0.00	0.00	0.00	0.00
51790	Staunton	Virginia	23,746	2,225	1,488.50	2,996.50	1,090,644.26	479,686.58	2,164,773.51	2,101,823.49	470,614.63	7,995,482.81	3,192,467.75	1,076,160.04	9,478,644.24
51800	Suffolk	Virginia	84,585	8,664	6,213.00	11,175.00	3,351,305.81	1,961,153.77	5,527,575.76	6,162,420.73	2,174,740.51	16,781,230.78	9,513,726.54	4,523,342.93	20,289,055.46
51810	Virginia Beach	Virginia	437,994	42,709	29,372.00	55,195.00	16,182,804.40	10,599,404.06	22,220,185.71	27,734,418.03	14,473,283.97	52,169,211.02	43,917,222.43	26,768,753.43	70,207,626.70
51820	Waynesboro	Virginia	21,006	2,025	1,399.00	2,734.50	885,024.57	414,190.63	1,754,136.38	1,561,891.83	372,028.09	5,448,313.04	2,446,916.41	892,580.34	6,696,884.90
51830	Williamsburg	Virginia	14,068	1,314	948.50	1,690.50	550,003.77	222,174.37	1,237,509.27	1,082,214.22	206,189.44	5,257,019.20	1,632,217.98	463,717.94	6,312,290.11
51840	Winchester	Virginia	26,203	2,615	1,824.50	3,412.50	1,089,247.17	531,107.14	2,164,327.75	1,993,524.59	549,618.00	7,435,952.15	3,082,771.76	1,213,220.29	8,778,993.21
53001	Adams	Washington	18,728	1,552	845.50	2,265.00	534,976.11	180,533.47	1,233,927.33	799,812.52	167,349.77	3,518,517.93	1,334,788.62	387,594.33	4,301,915.46
53003	Asotin	Washington	21,623	1,574	833.00	2,286.00	769,788.13	263,313.50	1,667,081.16	1,285,099.56	206,503.38	4,817,000.11	2,054,887.69	520,712.86	5,925,491.95
53005	Benton	Washington	175,177	13,734	7,850.00	19,810.50	5,388,434.36	2,766,118.87	8,807,420.80	10,183,051.65	4,028,733.57	23,162,753.80	15,571,486.01	7,069,712.44	30,052,853.34
53007	Chelan	Washington	72,453	5,787	3,373.00	8,154.50	2,548,391.17	1,213,952.08	4,608,458.70	4,329,203.10	1,227,811.70	12,300,921.87	6,877,594.27	2,690,683.61	15,768,818.91
53009	Clallam	Washington	71,404	5,631	3,182.50	7,940.50	3,077,459.73	1,623,136.05	5,207,678.30	5,987,725.96	1,792,186.51	16,159,962.79	9,065,185.69	3,654,319.21	19,288,126.36
53011	Clark	Washington	425,363	33,090	19,231.50	45,474.00	12,986,620.41	7,158,336.69	18,865,220.92	23,131,498.65	11,015,362.35	44,431,703.15	36,118,119.07	19,160,621.33	60,398,764.30
53013	Columbia	Washington	4,078	306	157.00	439.50	160,131.20	32,791.39	490,414.95	308,453.36	41,192.10	2,023,878.61	468,584.56	83,581.57	2,246,527.25
53015	Cowlitz	Washington	102,410	8,016	4,860.00	11,215.00	3,545,113.16	1,847,950.71	5,895,887.58	6,525,149.15	2,248,215.09	17,935,823.02	10,070,262.31	4,453,799.61	21,975,634.75
53017	Douglas	Washington	38,431	3,026	1,594.00	4,308.00	1,203,758.40	511,239.79	2,281,037.48	2,107,215.10	404,565.32	7,686,093.10	3,310,973.50	1,004,219.78	9,235,023.87
53019	Ferry	Washington	7,551	565	316.00	830.00	281,852.50	70,409.62	824,178.14	466,406.01	76,211.78	2,381,345.53	748,258.51	157,475.61	2,770,245.69
53021	Franklin	Washington	78,163	6,507	3,742.50	9,211.00	2,060,346.42	962,278.57	3,558,278.93	3,138,758.73	827,421.74	9,793,266.56	5,199,105.15	1,882,515.23	12,354,378.80
53023	Garfield	Washington	2,266	161	89.50	242.50	85,276.02	10,080.40	311,471.31	142,799.32	20,851.53	828,059.07	228,075.34	34,437.87	1,128,060.78
53025	Grant	Washington	89,120	7,314	4,355.00	10,369.00	2,733,126.49	1,313,882.62	4,691,196.20	4,616,504.93	1,372,314.58	13,713,481.05	7,349,631.42	2,976,657.97	16,529,383.85
53027	Grays Harbor	Washington	72,797	5,744	3,353.00	7,956.50	2,653,460.14	1,311,897.93	4,605,587.61	4,949,874.37	1,400,856.68	15,401,258.20	7,603,334.50	2,986,015.28	18,463,146.37
53029	Island	Washington	78,506	6,241	3,853.00	8,512.50	3,009,586.12	1,558,512.23	5,303,434.12	5,667,047.16	1,796,050.40	14,578,475.18	8,676,633.29	3,612,720.07	17,893,193.03
53031	Jefferson	Washington	29,872	2,302	1,401.50	3,203.00	1,412,582.65	630,508.67	2,868,191.59	2,569,301.76	570,491.16	8,416,331.11	3,981,884.41	1,337,574.13	10,405,169.01
53033	King	Washington	1,931,249	152,409	97,842.00	208,156.00	60,114,831.55	38,888,816.97	82,489,276.18	119,915,765.60	70,624,650.18	180,872,953.10	180,030,597.10	110,828,351.10	259,963,888.30
53035	Kitsap	Washington	251,133	19,725	12,750.50	26,797.50	8,429,811.40	4,784,382.81	12,959,256.46	15,558,897.22	6,830,359.13	32,173,415.38	23,988,708.62	12,190,450.04	42,359,844.71

53037	Kittitas	Washington	40,915	3,109	1,834.00	4,368.00	1,314,437.43	607,404.52	2,805,096.14	2,223,785.01	482,859.89	7,856,178.78	3,538,222.45	1,197,064.42	9,866,473.13
53039	Klickitat	Washington	20,318	1,549	917.50	2,207.00	789,405.01	286,450.76	1,732,798.08	1,561,120.85	273,014.43	6,097,139.62	2,350,525.86	632,543.23	7,305,612.76
53041	Lewis	Washington	75,455	5,872	3,488.00	8,213.00	2,719,143.64	1,439,079.83	4,767,999.53	4,869,661.55	1,440,244.75	13,486,782.85	7,588,805.18	3,145,765.88	16,765,049.16
53043	Lincoln	Washington	10,570	802	447.00	1,162.50	402,942.69	117,549.44	1,043,849.48	823,635.57	105,601.35	4,048,415.56	1,226,578.26	247,915.32	4,799,470.46
53045	Mason	Washington	60,699	4,756	3,073.00	6,662.00	2,338,231.79	1,126,110.05	4,114,844.01	4,261,647.13	1,109,503.91	11,980,029.64	6,599,878.92	2,510,330.14	14,980,817.22
53047	Okanogan	Washington	41,120	3,202	1,662.00	4,697.00	1,496,546.25	616,335.10	2,956,511.19	2,711,868.25	512,047.69	10,662,505.61	4,208,414.50	1,334,634.58	12,143,858.75
53049	Pacific	Washington	20,920	1,609	947.50	2,272.00	910,687.21	357,755.43	1,912,182.21	1,679,336.09	293,409.58	6,745,441.91	2,590,023.30	753,557.34	7,659,245.68
53051	Pend Oreille	Washington	13,001	928	444.00	1,409.00	470,652.17	110,638.56	1,145,509.18	888,244.02	114,779.83	4,055,426.23	1,358,896.19	260,470.30	4,662,980.83
53053	Pierce	Washington	795,225	63,372	39,382.00	86,596.00	24,597,810.60	14,736,587.83	36,078,951.73	43,605,060.75	22,413,142.45	73,193,197.98	68,202,871.35	38,832,635.24	102,873,059.90
53055	San Juan	Washington	15,769	1,222	749.00	1,733.00	716,732.88	270,694.41	1,604,928.54	1,324,898.23	234,636.50	5,333,970.58	2,041,631.11	568,234.47	6,740,014.13
53057	Skagit	Washington	116,901	9,396	5,365.50	13,618.50	4,210,709.88	2,043,248.70	7,019,835.31	7,687,747.88	2,558,053.14	17,568,722.50	11,898,457.76	5,124,152.60	23,025,851.37
53059	Skamania	Washington	11,066	842	496.00	1,204.00	405,045.08	113,121.38	1,035,730.60	668,529.67	105,502.66	3,519,424.51	1,073,574.75	241,731.33	4,220,046.19
53061	Snohomish	Washington	713,335	56,216	33,455.00	78,162.00	21,740,151.41	12,448,836.14	31,406,853.64	38,844,790.75	19,551,034.47	67,128,193.94	60,584,942.17	32,631,602.71	95,210,098.75
53063	Spokane	Washington	471,221	34,424	20,608.00	48,962.50	14,193,753.36	8,230,794.54	21,317,941.50	25,511,308.01	11,817,828.03	46,854,083.22	39,705,061.36	20,794,353.33	65,724,263.08
53065	Stevens	Washington	43,531	3,132	1,603.50	4,734.50	1,527,902.20	558,363.54	3,126,779.87	2,665,691.62	509,521.22	9,212,566.30	4,193,593.82	1,253,306.35	11,252,895.13
53067	Thurston	Washington	252,264	19,498	11,658.50	27,981.50	8,209,317.85	4,520,831.39	12,669,155.29	14,684,165.55	6,363,451.40	30,791,604.05	22,893,483.40	11,782,178.92	42,498,761.86
53069	Wahkiakum	Washington	3,978	304	172.00	432.50	170,671.15	35,215.78	533,264.92	315,431.81	40,300.39	1,739,780.20	486,102.96	82,222.62	1,969,903.59
53071	Walla Walla	Washington	58,781	4,415	2,287.50	6,404.50	1,909,319.89	781,379.80	3,652,483.85	3,426,379.70	820,688.60	9,689,398.92	5,335,699.58	1,790,364.57	12,519,187.32
53073	Whatcom	Washington	201,140	15,817	8,741.00	22,888.50	6,668,982.43	3,168,588.26	10,549,347.32	12,085,860.04	4,423,719.44	29,041,090.79	18,754,842.47	8,129,657.78	37,151,695.85
53075	Whitman	Washington	44,776	3,034	1,658.50	4,466.50	1,106,107.27	434,225.95	2,195,152.31	1,864,221.75	357,678.35	7,659,190.98	2,970,329.02	813,846.85	8,808,410.19
53077	Yakima	Washington	243,231	19,949	11,531.50	28,087.50	7,395,417.60	3,764,904.20	11,551,314.06	12,346,898.71	4,584,652.11	28,679,100.16	19,742,316.31	8,951,965.51	37,538,907.25
54001	Barbour	West Virginia	16,589	1,484	970.50	1,994.00	699,961.75	269,708.39	1,641,750.75	1,218,743.10	197,948.39	5,800,096.73	1,918,704.85	534,876.71	6,908,550.63
54003	Berkeley	West Virginia	104,169	10,735	7,812.00	13,841.00	4,249,228.12	2,628,103.12	6,683,925.80	7,195,358.80	2,941,319.78	18,888,500.05	11,444,586.92	6,068,610.12	24,182,494.14
54005	Boone	West Virginia	24,629	1,824	1,037.00	2,597.00	819,621.38	309,073.27	1,989,071.65	1,598,857.49	415,043.42	6,281,028.38	2,418,478.87	784,425.90	7,230,151.90
54007	Braxton	West Virginia	14,523	1,158	703.00	1,628.00	583,065.16	206,169.66	1,418,211.89	1,054,267.73	156,053.79	5,281,642.51	1,637,332.89	412,300.96	6,125,060.23
54009	Brooke	West Virginia	24,069	2,075	1,358.00	2,807.00	1,034,349.46	456,172.72	2,042,541.03	2,008,759.84	418,332.20	7,121,044.02	3,043,109.30	1,015,452.27	8,188,957.54
54011	Cabell	West Virginia	96,319	6,839	3,801.00	9,887.50	3,070,043.28	1,377,384.24	5,406,321.35	5,613,833.36	1,543,501.34	16,565,015.18	8,683,876.64	3,240,609.68	19,910,428.92
54013	Calhoun	West Virginia	7,627	594	361.00	827.00	275,986.54	79,268.51	749,417.30	470,069.67	74,202.16	2,278,041.92	746,056.21	160,783.94	2,872,270.11
54015	Clay	West Virginia	9,386	733	415.50	1,040.00	330,104.43	90,459.04	1,060,299.91	644,455.67	101,520.18	3,196,869.76	974,560.11	211,254.34	3,579,292.72
54017	Doddridge	West Virginia	8,202	663	402.00	930.00	309,977.33	81,742.68	901,916.91	478,525.63	70,062.30	2,753,226.52	788,502.96	163,522.80	3,271,204.78
54019	Fayette	West Virginia	46,039	3,473	2,010.50	4,873.00	1,657,052.50	678,318.00	3,059,349.08	2,926,566.41	636,844.53	9,922,105.60	4,583,618.91	1,502,869.78	11,960,641.87
54021	Gilmer	West Virginia	8,693	653	389.50	903.50	291,418.61	75,341.60	897,299.54	587,115.58	74,368.61	3,233,628.60	878,534.19	166,233.39	3,507,490.58
54023	Grant	West Virginia	11,937	1,139	779.00	1,508.00	570,546.63	211,712.08	1,343,187.92	1,043,289.49	229,209.69	4,399,377.05	1,613,836.13	507,469.86	5,395,701.84
54025	Greenbrier	West Virginia	35,480	2,843	1,734.00	3,894.50	1,403,672.40	669,283.94	2,642,301.59	2,504,847.40	547,605.90	8,509,512.49	3,908,519.80	1,381,150.78	10,020,483.99
54027	Hampshire	West Virginia	23,964	2,334	1,628.00	3,057.50	1,074,895.73	509,180.50	2,123,544.41	1,805,058.19	377,020.47	6,461,339.58	2,879,953.92	999,155.04	7,715,544.05
54029	Hancock	West Virginia	30,676	2,663	1,768.50	3,495.50	1,323,244.74	626,255.27	2,532,811.08	2,345,925.03	548,875.40	7,671,328.39	3,669,169.77	1,402,810.03	9,120,200.81
54031	Hardy	West Virginia	14,025	1,346	938.00	1,794.00	608,992.73	235,904.02	1,315,326.10	1,081,183.04	193,115.53	4,439,830.41	1,690,175.77	487,396.81	5,123,871.91
54033	Harrison	West Virginia	69,099	5,898	3,758.00	8,092.00	2,698,616.56	1,352,539.19	4,564,840.08	4,860,946.33	1,590,082.25	13,201,385.72	7,559,562.89	3,329,163.07	16,782,314.54
54035	Jackson	West Virginia	29,211	2,203	1,260.50	3,114.00	1,020,720.72	412,248.48	2,062,142.59	1,748,175.72	358,555.82	6,285,638.31	2,768,896.44	876,182.48	7,457,843.91
54037	Jefferson	West Virginia	53,498	5,464	3,976.00	6,955.00	2,210,284.56	1,234,450.90	3,977,643.17	3,620,692.48	1,061,067.52	10,285,855.63	5,830,977.04	2,523,152.18	12,616,265.43
54039	Kanawha	West Virginia	193,063	14,101	8,370.50	20,168.00	6,654,303.58	3,465,216.68	10,909,571.31	12,567,468.32	4,855,893.80	27,309,252.93	19,221,771.90	9,434,503.86	35,312,585.04
54041	Lewis	West Virginia	16,372	1,380	857.00	1,910.50	669,810.39	268,855.76	1,455,960.55	1,195,209.22	226,236.64	4,549,150.99	1,865,019.61	531,515.14	5,530,119.48
54043	Lincoln	West Virginia	21,720	1,568	839.50	2,237.00	693,824.31	218,154.57	1,593,996.96	1,204,269.83	213,453.95	4,953,623.06	1,898,094.14	472,887.06	5,862,908.44
54045	Logan	West Virginia	36,743	2,617	1,404.50	3,769.00	1,226,924.85	485,088.69	2,506,135.70	2,154,712.58	439,463.04	7,744,271.65	3,381,637.43	1,071,906.06	9,075,699.61
54047	McDowell	West Virginia	22,113	1,615	917.50	2,307.00	774,124.17	278,798.28	1,671,986.34	1,432,197.93	282,291.47	6,354,731.12	2,206,322.10	644,037.57	7,345,830.94
54049	Marion	West Virginia	56,418	4,965	3,211.50	6,728.50	2,359,218.02	1,185,559.39	3,994,623.30	4,025,434.33	1,272,806.46	10,573,241.94	6,384,652.34	2,787,787.82	13,525,742.31
54051	Marshall	West Virginia	33,107	2,791	1,773.50	3,821.00	1,332,079.65	593,466.17	2,518,576.09	2,722,517.99	686,820.08	8,746,310.98	4,054,597.63	1,418,101.36	10,305,805.41
54053	Mason	West Virginia	27,324	1,995	1,178.50	2,876.00	916,656.05	361,791.81	1,917,614.13	1,808,501.80	377,129.68	7,544,575.79	2,725,157.85	854,534.81	8,897,870.72
54055	Mercer	West Virginia	62,264	4,569	2,679.50	6,495.00	2,172,054.66	1,012,369.78	3,874,603.39	3,797,283.13	904,510.06	11,496,444.08	5,969,337.79	2,162,517.68	14,562,438.51
54057	Mineral	West Virginia	28,212	2,700	1,857.00	3,505.50	1,284,583.23	624,360.71	2,645,848.02	2,336,998.16	567,246.13	9,520,980.51	3,621,581.39	1,341,878.77	11,202,072.09
54059	Mingo	West Virginia	26,839	1,933	1,158.00	2,799.00	841,577.01	322,680.93	1,894,694.89	1,543,050.35	372,668.99	5,666,628.49	2,384,627.36	733,960.22	6,743,480.60
54061	Monongalia	West Virginia	96,189	8,243	5,536.50	10,857.00	3,171,906.15	1,765,942.31	5,079,563.64	5,320,124.20	1,796,455.29	14,345,249.17	8,492,030.35	3,790,123.22	17,539,197.26
54063	Monroe	West Virginia	13,502	1,057	665.50	1,474.00	520,568.39	185,354.28	1,242,296.61	919,168.06	154,535.13	3,801,655.69	1,439,736.44	390,802.81	4,565,110.48
54065	Morgan	West Virginia	17,541	1,704	1,169.50	2,230.00	865,485.70	353,463.91	1,971,448.85	1,517,694.19	270,049.92	6,190,310.00	2,383,179.89	725,630.89	7,120,600.60
54067	Nicholas	West Virginia	26,233	2,055	1,302.00	2,899.50	965,362.27	410,432.74	1,955,504.40	1,604,398.57	365,588.01	5,605,900.93	2,569,760.84	902,123.98	7,175,735.26
54069	Ohio	West Virginia	44,443	3,826	2,516.50	5,104.50	1,904,245.11	938,342.93	3,379,218.32	3,366,337.89	800,161.01	11,048,060.25	5,270,583.00	2,048,270.74	13,397,456.54
54071	Pendleton	West Virginia	7,695	706	465.00	947.00	372,517.22	116,551.23	978,212.75	691,926.24	110,523.98	3,628,053.35	1,064,443.46	248,894.50	4,270,720.78
54073	Pleasants	West Virginia	7,605	590	353.50	836.00	273,796.10	69,049.71	814,267.14	532,638.99	87,010.80	2,721,822.79	806,435.08	166,064.78	3,247,139.42
54075	Pocahontas	West Virginia	8,719	723	447.00	980.50	392,379.42	112,921.23	1,165,523.41	687,552.41	87,340.25	3,263,531.77	1,079,931.83	216,129.50	4,087,207.72
54077	Preston	West Virginia	33,520	3,103	2,106.00	4,094.50	1,461,800.75	743,416.94	2,756,457.24	2,534,086.66	590,212.49	8,512,357.11	3,995,887.41	1,567,327.88	10,139,380.12
54079	Putnam	West Virginia	55,486	4,038	2,335.00	5,715.00	1,731,332.87	783,841.65	3,111,017.92	3,161,582.31	820,445.57	9,376,953.96	4,892,915.19	1,841,731.10	11,444,705.66
54081	Raleigh	West Virginia	78,859	5,790	3,241.00	8,226.00	2,684,368.32	1,221,507.47	4,907,064.75	4,761,633.98	1,331,915.61	12,810,914.23	7,446,002.30	2,797,073.32	15,975,832.87
54083	Randolph	West Virginia	29,405	2,567	1,634.50	3,515.50	1,221,284.97	535,714.53	2,397,282.17	2,110,970.05	428,273.28	7,193,612.36	3,332,255.02	1,072,282.97	8,645,250.12
54085	Ritchie	West Virginia	10,449	826	483.50	1,152.50	400,241.74	120,033.12	1,125,520.99	610,596.31	110,061.29	2,803,606.51	1,010,838.06	258,604.85	3,345,927.72
54087	Roane	West Virginia	14,926	1,128	656.00	1,633.50	549,954.46	186,960.78	1,288,762.64	848,491.53	142,948.06	3,640,666.44	1,398,445.99	363,386.65	4,872,802.16
54089	Summers	West Virginia	13,927	1,039	620.50	1,425.50	536,028.84	187,920.12	1,340,930.94	941,820.74	140,202.05	4,244,491.76	1,477,849.59	362,876.61	4,956,239.69

54091	Taylor	West Virginia	16,895	1,514	991.50	2,027.00	695,297.69	283,686.67	1,486,890.28	1,206,382.17	228,414.64	5,233,962.84	1,901,679.86	568,966.31	6,166,671.23
54093	Tucker	West Virginia	7,141	654	415.00	863.50	334,269.30	102,148.64	904,821.79	553,156.72	77,011.99	3,098,589.38	887,426.01	201,724.40	3,645,587.37
54095	Tyler	West Virginia	9,208	738	447.00	1,008.50	361,853.54	107,562.78	1,020,506.04	633,283.86	116,528.74	3,141,789.37	995,137.40	235,072.32	3,547,500.60
54097	Upshur	West Virginia	24,254	2,066	1,298.50	2,817.50	939,308.02	411,775.64	1,824,921.55	1,531,518.52	335,395.63	5,569,366.32	2,470,826.54	872,330.89	6,850,490.17
54099	Wayne	West Virginia	42,481	3,100	1,675.00	4,386.50	1,389,266.87	603,227.76	2,741,497.20	2,677,459.41	641,391.69	9,092,764.86	4,066,726.28	1,407,000.15	10,563,352.26
54101	Webster	West Virginia	9,154	759	469.50	1,044.50	359,975.96	102,861.06	863,301.42	678,566.66	106,318.44	3,834,010.39	1,038,542.61	233,914.34	4,384,300.12
54103	Wetzel	West Virginia	16,583	1,392	862.00	1,926.50	673,311.39	272,753.76	1,448,902.09	1,284,631.70	188,815.45	5,274,369.74	1,957,943.09	516,698.70	6,111,537.66
54105	Wirt	West Virginia	5,717	426	239.50	610.50	186,394.29	44,441.35	534,330.19	360,388.16	38,798.94	2,210,491.18	546,782.45	90,911.98	2,519,238.23
54107	Wood	West Virginia	86,956	6,576	3,695.50	9,194.50	3,052,396.50	1,473,493.44	5,252,113.76	5,692,980.61	1,619,765.51	14,968,918.81	8,745,377.11	3,454,302.91	19,215,941.43
54109	Wyoming	West Virginia	23,796	1,731	1,021.00	2,455.00	804,460.05	313,587.60	1,776,404.19	1,398,638.06	275,884.88	5,599,843.96	2,203,098.11	657,877.88	6,855,024.58
55001	Adams	Wisconsin	20,875	1,430	783.00	2,098.00	816,452.22	281,039.75	1,914,581.94	1,432,861.82	226,096.72	5,149,101.04	2,249,314.04	586,373.72	6,293,290.01
55003	Ashland	Wisconsin	16,157	1,152	518.00	1,802.00	525,453.33	143,963.80	1,299,211.14	920,122.91	127,380.19	4,624,828.11	1,445,576.24	301,426.45	5,171,555.45
55005	Barron	Wisconsin	45,870	3,217	1,777.50	4,749.50	1,555,650.12	625,537.36	3,154,020.32	2,675,084.11	577,187.79	8,745,290.11	4,230,734.23	1,389,959.67	10,734,267.74
55007	Bayfield	Wisconsin	15,014	1,046	431.50	1,612.00	586,078.34	122,057.84	1,409,103.67	998,661.84	87,974.31	4,605,697.90	1,584,740.19	235,813.43	5,470,537.10
55009	Brown	Wisconsin	248,007	17,964	9,689.50	26,724.00	7,038,531.58	3,451,532.94	11,466,349.85	12,772,727.58	4,784,209.71	28,106,039.18	19,811,259.16	8,439,925.31	37,558,297.14
55011	Buffalo	Wisconsin	13,587	928	511.50	1,378.00	441,987.87	130,108.47	1,148,584.56	807,555.54	118,676.12	4,085,661.55	1,249,543.40	269,631.37	4,697,182.85
55013	Burnett	Wisconsin	15,457	1,068	553.00	1,605.00	589,128.14	192,357.04	1,326,371.02	1,130,183.04	149,996.66	5,329,755.80	1,719,311.17	404,974.27	5,924,465.56
55015	Calumet	Wisconsin	48,971	3,502	1,918.50	5,018.50	1,344,398.86	572,247.99	2,597,423.92	2,238,934.73	468,803.99	8,843,103.90	3,583,333.59	1,188,996.59	10,418,316.21
55017	Chippewa	Wisconsin	62,415	4,483	2,372.50	6,581.50	1,917,434.51	793,842.77	3,501,533.04	3,357,351.94	777,231.53	12,032,538.76	5,274,786.44	1,869,298.78	14,169,644.89
55019	Clark	Wisconsin	34,690	2,591	1,333.50	3,772.00	1,079,010.02	417,942.22	2,081,288.14	1,944,681.32	361,974.06	7,114,850.20	3,023,691.34	917,345.28	8,254,425.40
55021	Columbia	Wisconsin	56,833	4,024	2,408.50	5,680.00	1,786,164.97	801,733.42	3,516,250.62	3,026,609.10	700,331.27	9,738,705.72	4,812,774.07	1,684,964.81	11,711,484.39
55023	Crawford	Wisconsin	16,644	1,116	539.00	1,639.00	567,173.43	164,412.75	1,354,702.11	930,555.74	121,857.84	4,266,981.94	1,497,729.17	332,860.75	5,027,368.52
55025	Dane	Wisconsin	488,073	34,064	20,437.00	47,903.00	13,154,586.86	7,557,471.25	19,966,335.11	22,908,039.05	10,338,382.78	43,505,184.64	36,062,625.91	18,887,869.03	60,586,741.14
55027	Dodge	Wisconsin	88,759	6,163	3,646.00	8,550.50	2,695,425.54	1,336,027.62	4,669,679.98	5,230,713.04	1,520,930.94	14,971,572.48	7,926,138.58	3,054,377.25	17,750,152.74
55029	Door	Wisconsin	27,785	1,956	1,007.00	2,891.00	1,062,595.81	393,900.41	2,306,931.26	1,973,457.72	320,249.06	6,678,686.61	3,036,053.53	812,478.43	8,079,916.58
55031	Douglas	Wisconsin	44,159	3,081	1,512.50	4,635.50	1,348,301.58	447,347.49	2,734,786.13	2,298,705.63	490,795.81	7,739,666.10	3,647,007.21	1,090,146.42	9,999,736.50
55033	Dunn	Wisconsin	43,857	2,984	1,419.50	4,376.50	1,224,230.83	451,490.83	2,422,607.48	2,155,160.33	391,025.81	8,647,486.16	3,379,391.16	929,979.14	10,244,948.95
55035	Eau Claire	Wisconsin	98,736	6,848	3,601.00	10,192.50	2,788,979.29	1,186,630.68	4,941,686.56	4,874,921.57	1,336,764.93	13,102,010.36	7,663,900.86	2,795,267.92	16,746,968.86
55037	Florence	Wisconsin	4,423	308	148.00	473.50	170,744.61	25,983.25	583,921.04	284,541.38	22,721.66	1,932,291.50	455,285.99	52,373.46	2,139,353.96
55039	Fond Du Lac	Wisconsin	101,633	7,068	3,968.50	10,195.00	3,147,311.11	1,517,763.81	5,585,332.47	5,518,515.22	1,635,575.37	14,703,803.88	8,665,826.32	3,491,538.59	19,804,737.20
55041	Forest	Wisconsin	9,304	661	343.00	1,012.50	326,864.64	85,886.98	794,937.40	592,451.89	68,870.02	3,404,654.19	919,316.53	180,379.42	3,949,961.49
55043	Grant	Wisconsin	51,208	3,356	1,682.50	4,876.50	1,468,742.92	554,489.34	3,041,168.00	2,494,115.03	526,213.06	8,914,647.93	3,962,857.95	1,229,378.08	10,666,256.49
55045	Green	Wisconsin	36,842	2,571	1,494.50	3,663.00	1,127,918.25	471,096.57	2,200,021.83	2,018,947.74	396,772.77	7,285,963.23	3,146,865.99	1,040,752.22	8,567,344.25
55047	Green Lake	Wisconsin	19,051	1,356	763.00	1,956.50	651,070.30	226,762.92	1,528,684.32	1,127,653.42	202,197.20	4,922,565.73	1,778,723.72	511,787.31	5,854,030.85
55049	Iowa	Wisconsin	23,687	1,646	898.50	2,440.00	721,426.22	248,702.33	1,651,538.55	1,272,557.16	217,966.12	5,657,888.44	1,993,983.38	538,044.23	6,442,269.52
55051	Iron	Wisconsin	5,916	410	193.00	637.50	240,313.49	43,572.97	789,534.65	403,415.55	40,449.66	2,524,013.93	643,729.04	96,072.50	2,846,597.21
55053	Jackson	Wisconsin	20,449	1,437	792.00	2,130.50	648,258.98	224,689.49	1,460,066.94	1,117,938.14	205,804.37	4,935,899.91	1,766,197.13	475,756.05	6,012,778.78
55055	Jefferson	Wisconsin	83,686	5,898	3,449.50	8,481.50	2,472,837.24	1,170,167.53	4,512,704.08	4,247,693.76	1,167,749.83	12,000,290.47	6,720,531.00	2,502,056.68	15,157,875.36
55057	Juneau	Wisconsin	26,664	1,864	1,054.00	2,723.00	897,928.48	338,759.95	1,936,162.50	1,656,070.35	300,621.37	7,151,730.29	2,553,998.83	735,465.07	8,331,854.62
55059	Kenosha	Wisconsin	166,426	11,626	6,884.00	16,329.00	4,477,178.53	2,350,658.37	7,553,838.77	7,924,669.27	2,674,761.99	17,069,502.00	12,401,847.80	5,504,692.08	22,406,670.49
55061	Kewaunee	Wisconsin	20,574	1,459	786.00	2,169.50	632,979.88	218,633.55	1,392,737.16	1,120,769.46	221,756.12	4,773,141.53	1,753,749.34	484,466.32	5,587,756.47
55063	La Crosse	Wisconsin	114,638	7,659	4,456.00	11,289.50	3,172,366.41	1,549,475.76	5,662,729.04	5,863,266.27	1,718,783.69	16,475,554.36	9,035,632.68	3,588,894.20	19,690,127.78
55065	Lafayette	Wisconsin	16,836	1,159	625.00	1,698.50	508,020.20	159,547.77	1,220,425.83	826,922.67	128,718.97	3,867,219.09	1,334,942.87	322,720.89	4,528,084.93
55067	Langlade	Wisconsin	19,977	1,399	686.50	2,101.50	714,557.16	227,119.39	1,714,263.42	1,233,653.78	199,940.82	4,938,632.25	1,948,210.94	529,737.54	5,918,370.35
55069	Lincoln	Wisconsin	28,743	2,034	1,021.00	2,998.50	997,109.93	307,177.32	2,265,177.38	1,764,801.35	294,789.36	7,195,682.63	2,761,911.28	666,609.51	8,527,094.62
55071	Manitowoc	Wisconsin	81,442	5,671	3,060.00	8,383.50	2,615,472.32	1,172,518.52	4,647,365.03	4,854,741.69	1,348,511.07	13,053,871.52	7,470,214.01	2,625,517.26	16,532,101.88
55073	Marathon	Wisconsin	134,063	9,575	4,765.50	14,246.50	4,120,231.57	1,761,887.77	6,957,667.15	7,391,082.23	2,321,721.35	18,491,630.31	11,511,313.80	4,408,680.39	23,305,654.62
55075	Marinette	Wisconsin	41,749	2,953	1,449.00	4,545.00	1,491,358.13	514,500.65	3,067,975.60	2,572,691.33	503,991.21	8,116,696.05	4,064,049.47	1,203,823.74	10,006,042.32
55077	Marquette	Wisconsin	15,404	1,088	640.50	1,510.00	566,602.52	197,029.02	1,295,376.43	964,206.29	143,724.10	4,150,351.97	1,530,808.81	384,688.50	5,011,643.28
55078	Menominee	Wisconsin	4,232	321	155.50	488.50	122,485.95	17,992.05	414,161.39	181,442.40	23,659.13	1,343,249.32	303,928.34	43,974.30	1,720,621.97
55079	Milwaukee	Wisconsin	947,735	66,051	37,925.50	93,263.50	25,392,439.93	14,461,756.17	36,217,232.00	46,667,545.57	23,512,524.16	83,046,385.21	72,059,985.51	40,123,756.30	115,516,766.20
55081	Monroe	Wisconsin	44,673	3,166	1,708.50	4,582.00	1,325,277.98	540,288.05	2,774,009.52	2,458,093.40	503,517.25	8,763,445.64	3,783,371.38	1,095,488.83	10,301,116.60
55083	Oconto	Wisconsin	37,660	2,642	1,331.00	3,944.50	1,233,807.37	460,503.38	2,532,325.93	2,130,109.29	344,440.76	7,837,905.65	3,363,916.65	931,690.35	10,339,279.42
55085	Oneida	Wisconsin	35,998	2,506	1,241.50	3,921.00	1,326,897.48	465,342.56	2,746,782.99	2,519,999.04	444,986.15	8,129,354.28	3,846,896.52	1,069,478.80	10,203,294.41
55087	Outagamie	Wisconsin	176,695	12,543	6,661.50	18,463.50	4,943,889.22	2,427,535.12	8,138,659.04	8,593,987.22	2,965,371.77	20,309,616.45	13,537,876.44	5,813,409.58	26,276,504.71
55089	Ozaukee	Wisconsin	86,395	5,937	3,411.00	8,508.00	2,675,174.15	1,232,628.22	4,595,687.64	4,847,919.48	1,376,556.40	13,182,731.50	7,523,093.64	3,012,501.67	16,634,128.18
55091	Pepin	Wisconsin	7,469	520	291.00	776.50	260,350.67	58,901.12	809,835.44	452,066.49	56,940.26	2,451,309.34	712,417.16	127,518.94	2,777,957.31
55093	Pierce	Wisconsin	41,019	2,776	1,472.50	3,996.50	1,087,111.34	399,626.71	2,083,559.83	1,674,460.80	346,168.09	6,365,050.78	2,761,572.14	839,492.46	7,469,175.20
55095	Polk	Wisconsin	44,205	3,097	1,687.50	4,503.00	1,407,066.39	555,223.24	2,855,422.78	2,451,448.75	468,914.37	8,378,374.81	3,858,515.14	1,173,099.99	10,309,221.49
55097	Portage	Wisconsin	70,019	4,828	2,425.00	6,972.50	2,027,599.71	856,331.78	3,618,536.07	3,451,018.26	846,909.75	10,300,410.64	5,478,617.97	1,937,698.56	12,775,335.13
55099	Price	Wisconsin	14,159	997	515.00	1,504.00	538,256.27	132,233.52	1,339,718.10	950,954.76	118,686.07	4,961,741.71	1,489,211.03	290,035.08	5,818,742.35
55101	Racine	Wisconsin	195,408	13,627	8,000.00	19,066.00	5,628,560.02	2,973,934.94	8,942,166.70	10,086,226.90	3,780,432.71	23,706,887.33	15,714,786.92	7,302,991.60	30,444,975.91
55103	Richland	Wisconsin	18,021	1,243	650.00	1,850.00	586,224.17	204,880.36	1,317,360.74	1,084,946.79	171,806.92	4,649,773.37	1,671,170.96	420,221.21	5,430,486.79
55105	Rock	Wisconsin	160,331	11,322	6,906.50	15,747.50	4,668,049.08	2,555,556.56	7,568,826.88	8,493,652.17	2,968,217.88	20,514,325.25	13,161,701.24	6,232,570.27	25,773,657.68
55107	Rusk	Wisconsin	14,755	1,047	501.50	1,562.50	526,050.78	147,283.04	1,310,857.36	939,061.72	113,603.57	4,310,185.72	1,465,112.50	296,072.93	4,943,329.87
55109	St. Croix	Wisconsin	84,345	5,987	3,383.00	8,629.50	2,242,779.79	956,268.74	4,097,221.28	3,611,083.44	904,001.44	10,637,055.16	5,853,863.23	2,138,173.43	13,474,392.57

55111	Sauk	Wisconsin	61,976	4,365	2,487.00	6,325.00	1,887,378.47	810,667.38	3,474,366.71	3,398,223.05	750,993.11	12,055,852.09	5,285,601.52	1,754,191.51	14,301,409.14
55113	Sawyer	Wisconsin	16,557	1,175	560.50	1,776.50	591,625.86	167,592.78	1,368,047.89	1,090,691.37	134,792.71	4,731,208.74	1,682,317.23	319,603.70	5,438,116.53
55115	Shawano	Wisconsin	41,949	2,994	1,573.00	4,481.50	1,386,258.11	545,319.55	2,814,627.65	2,401,629.43	437,274.61	9,175,533.71	3,787,887.54	1,152,335.89	10,702,055.97
55117	Sheboygan	Wisconsin	115,507	8,150	4,560.00	11,888.00	3,490,848.89	1,652,153.94	5,858,555.56	6,530,444.26	1,869,492.40	16,802,932.30	10,021,293.15	3,725,549.38	21,409,562.30
55119	Taylor	Wisconsin	20,689	1,489	721.00	2,189.00	679,760.64	215,019.36	1,529,891.90	1,139,737.06	178,943.29	5,300,939.09	1,819,497.70	457,587.75	6,276,954.78
55121	Trempealeau	Wisconsin	28,816	2,007	1,032.50	2,951.00	895,385.97	316,541.60	2,124,598.66	1,581,347.53	283,041.13	5,433,153.77	2,476,733.50	672,127.41	6,821,660.90
55123	Vernon	Wisconsin	29,773	2,100	1,045.00	3,093.00	947,932.50	305,214.40	2,057,139.74	1,708,415.37	262,031.48	7,060,257.07	2,656,347.87	642,403.97	8,263,684.89
55125	Vilas	Wisconsin	21,430	1,526	695.50	2,370.00	871,055.10	268,488.47	1,979,309.60	1,504,345.41	206,140.66	5,740,097.43	2,375,400.52	584,530.38	6,784,740.67
55127	Walworth	Wisconsin	102,228	7,219	4,603.00	10,014.50	2,997,545.90	1,520,930.04	5,084,064.11	5,255,351.79	1,598,795.14	14,503,987.85	8,252,897.69	3,514,880.32	18,926,838.78
55129	Washburn	Wisconsin	15,911	1,121	558.50	1,724.50	592,346.72	168,634.22	1,472,505.43	959,171.32	129,078.77	4,099,803.48	1,551,518.04	307,885.81	4,860,970.78
55131	Washington	Wisconsin	131,887	9,210	5,280.00	12,953.00	3,873,189.68	1,905,337.90	6,403,153.80	6,797,362.65	2,235,057.26	17,594,697.93	10,670,552.33	4,440,932.39	22,333,231.81
55133	Waukesha	Wisconsin	389,891	26,747	15,987.50	37,571.00	11,607,757.25	6,612,461.40	17,700,777.99	22,116,172.99	10,085,715.61	41,937,047.93	33,723,930.24	17,098,709.28	56,043,153.79
55135	Waupaca	Wisconsin	52,410	3,720	1,903.50	5,560.00	1,768,375.74	756,003.14	3,525,860.27	3,102,375.60	673,449.38	10,331,670.69	4,870,751.35	1,654,700.87	13,319,400.91
55137	Waushara	Wisconsin	24,496	1,711	944.00	2,477.50	875,080.37	311,429.84	1,908,668.06	1,557,491.53	242,335.37	5,859,386.26	2,432,571.89	637,175.50	6,840,107.89
55139	Winnebago	Wisconsin	166,994	11,659	6,584.50	16,828.50	4,840,870.73	2,381,367.54	7,714,932.03	8,790,136.48	3,075,379.75	20,795,039.04	13,631,007.21	6,174,482.12	26,237,121.41
55141	Wood	Wisconsin	74,749	5,320	2,813.00	7,760.50	2,507,254.65	1,082,118.78	4,572,825.71	4,773,929.02	1,227,013.83	13,808,218.80	7,281,183.67	2,525,182.22	16,649,726.45
56001	Albany	Wyoming	36,299	1,435	219.00	2,804.50	519,476.56	44,372.75	1,355,531.28	842,785.39	38,502.80	3,591,400.89	1,362,261.95	86,492.04	4,797,150.75
56003	Big Horn	Wyoming	11,668	516	70.00	1,053.50	243,323.85	12,543.30	836,693.21	438,854.88	16,516.81	2,549,907.03	682,178.73	32,501.62	3,137,788.82
56005	Campbell	Wyoming	46,133	1,972	158.00	3,964.00	649,347.38	23,647.48	1,790,556.53	1,141,043.94	38,852.99	4,363,399.99	1,790,391.32	60,903.12	5,933,588.05
56007	Carbon	Wyoming	15,885	680	97.00	1,287.00	285,590.20	20,239.67	846,958.37	465,939.97	24,035.57	2,534,714.56	751,530.17	46,924.35	3,165,230.14
56009	Converse	Wyoming	13,833	581	54.00	1,192.50	245,611.98	9,314.44	731,127.08	475,433.52	15,299.72	2,893,781.48	721,045.50	26,041.48	3,356,793.30
56011	Crook	Wyoming	7,083	362	41.50	697.50	163,469.87	5,048.03	550,369.65	288,735.46	8,220.40	2,008,075.26	452,205.33	14,911.42	2,207,102.53
56013	Fremont	Wyoming	40,123	1,815	318.50	3,338.50	781,844.23	77,914.58	1,919,533.22	1,411,820.88	74,491.58	6,420,757.38	2,193,665.10	158,979.96	7,617,811.49
56015	Goshen	Wyoming	13,249	577	69.50	1,132.00	289,178.61	16,118.39	842,375.58	518,335.06	15,597.58	2,550,340.19	807,513.67	33,870.95	3,234,258.82
56017	Hot Springs	Wyoming	4,812	202	17.00	393.50	117,421.41	1,760.83	469,215.99	170,940.66	3,741.48	1,140,586.56	288,362.06	5,475.19	1,444,350.15
56019	Johnson	Wyoming	8,569	366	31.50	760.00	169,835.95	3,956.01	569,691.70	318,974.66	6,559.25	1,935,564.45	488,810.61	12,051.16	2,236,346.80
56021	Laramie	Wyoming	91,738	4,144	671.50	7,702.00	1,677,314.07	272,781.56	3,633,160.43	2,983,434.67	240,478.01	10,168,576.54	4,660,748.75	564,599.70	13,303,877.35
56023	Lincoln	Wyoming	18,106	884	191.00	1,583.00	357,788.03	35,871.30	921,667.56	650,609.66	46,912.54	3,341,913.16	1,008,397.69	83,490.42	4,069,271.70
56025	Natrona	Wyoming	75,450	3,129	299.00	6,197.50	1,296,112.77	70,032.35	3,224,353.19	2,399,569.19	79,853.79	9,029,791.38	3,695,681.96	154,487.30	11,384,025.56
56027	Niobrara	Wyoming	2,484	102	8.50	214.00	51,508.11	423.50	232,709.56	73,591.00	1,932.61	425,100.56	125,099.11	2,563.11	643,425.23
56029	Park	Wyoming	28,205	1,305	179.50	2,574.50	618,096.12	41,035.11	1,587,255.74	1,181,431.87	49,684.82	5,351,100.89	1,799,527.99	104,443.31	6,519,352.35
56031	Platte	Wyoming	8,667	361	51.50	709.00	186,293.20	8,520.51	621,938.65	319,627.91	12,210.16	1,723,962.09	505,921.10	21,697.05	2,095,555.37
56033	Sheridan	Wyoming	29,116	1,234	94.00	2,564.00	557,800.79	19,625.44	1,509,281.27	1,077,022.45	24,940.93	4,512,020.44	1,634,823.24	46,551.17	5,354,733.65
56035	Sublette	Wyoming	10,247	469	74.50	855.00	182,393.98	9,632.07	598,063.15	363,825.01	22,712.89	2,122,573.79	546,218.99	33,746.56	2,528,658.55
56037	Sweetwater	Wyoming	43,806	2,014	389.50	3,648.50	723,198.65	105,706.29	1,786,966.04	1,179,926.00	117,246.63	4,526,071.34	1,903,124.64	244,661.11	5,831,332.76
56039	Teton	Wyoming	21,294	1,013	145.00	1,921.00	407,669.57	33,685.89	1,072,044.00	736,051.84	34,538.39	4,255,036.53	1,143,721.42	66,884.24	5,006,627.18
56041	Uinta	Wyoming	21,118	1,019	231.00	1,756.50	368,209.43	43,181.37	1,027,343.09	644,712.79	53,975.82	2,877,815.66	1,012,922.21	100,772.08	3,661,488.30
56043	Washakie	Wyoming	8,533	371	32.50	768.00	164,815.25	3,995.48	555,287.53	288,835.12	7,059.94	1,735,483.00	453,650.37	13,930.62	2,184,912.97
56045	Weston	Wyoming	7,208	325	29.00	645.50	148,609.15	2,685.64	539,102.05	237,326.77	6,238.14	1,356,884.08	385,935.91	9,087.04	1,592,159.08
